# 3,4,5‐Trifluorophenyldiazonium–A Unique Radical Source for the Visible Light Induced, Catalyst‐Free Arylation of Tyrosine Residues in Peptides

**DOI:** 10.1002/chem.202501160

**Published:** 2025-06-18

**Authors:** Meret K. Kuschow, Daniel R. Troeger, Luca‐Sophie Schuster, Leonard Bock, Leon A. Zähnle, Markus R. Heinrich

**Affiliations:** ^1^ Department of Chemistry and Pharmacy Pharmaceutical Chemistry Friedrich‐Alexander‐Universität Erlangen‐Nürnberg Nikolaus‐Fiebiger‐Str. 10 91058 Erlangen Germany

**Keywords:** arylation, diazonium, peptides, radical reactions, tyrosine

## Abstract

The direct radical arylation of tyrosine residues in peptides has been achieved under highly attractive conditions, requiring only the peptide substrate, a 3,4,5‐trifluorophenyldiazonium salt, and irradiation with visible light. Being well‐balanced in its reactivity to form strong charge‐transfer complexes with the phenolic side chain, which are required for initiation and aryl radical formation, the trifluoro substitution still shows sufficient stability toward nucleophilic aromatic substitution in aqueous solution. Besides allowing selective arylation, the introduction of the 3,4,5‐trifluorophenyl unit enables further functionalization as well as potential applications in NMR and MRT studies.

## Graphical abstract

By exploiting the unique properties of 3,4,5‐trifluorophenyldiazonium salts in combination with phenols, radical arylations of tyrosine units in peptide substrates could for the first time be achieved under highly attractive conditions requiring only visible light irradiation and neither photocatalysts nor other additives, pre‐functionalization, or protecting groups.

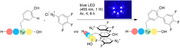

## 1 Introduction

**Scheme 1:**
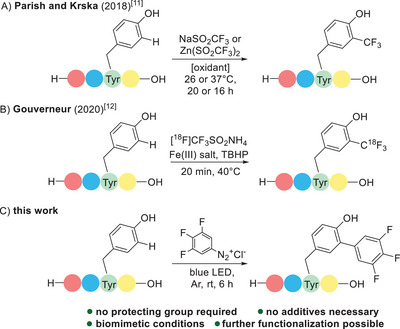
Trifluormethylation and trifluoroarylation of tyrosine in peptides.

The selective chemical functionalization of native, nonprotected peptides represents a highly challenging field of research. While a broad range of methods is known for the modification of cysteine units at their thiol group^[^ ^1^, ^2^, ^3^ ^]^ and lysines at their amino functionality,^[^ ^1^, ^4^ ^]^ reactions at the aromatic side chains of phenylalanine,^[^ ^5^ ^]^ tyrosine,^[^ ^6^, ^7^ ^]^ histidine^[^ ^8^ ^]^, or tryptophan^[^ ^9^ ^]^ are much less developed.^[^ ^10^ ^]^ Regarding tyrosine in particular, especially radical functionalizations have recently been described, whereat the trifluoromethyl radical plays a pivotal role (Scheme 1A,B).^[^ ^11^, ^12^ ^]^ Such trifluoromethylations can either be achieved by stoichiometric reductants or under photoredox catalysis. Attractive radical arylations of tyrosine residues, on the other hand, are yet unknown.^[^ ^7^ ^]^

From a general perspective, a virtually ideal arylation reaction setup would only require the aryl radical precursor and the nonmodified peptide substrate with visible light being the trigger for initiation. Based on seminal work by Kochi,^[^ ^13^ ^]^ such initiation can basically be achieved by exploiting photo‐excitable charge transfer (CT) complexes between an aromatic substrate and a diazonium salt, which leads to the formation of aryl radicals.^[^ ^14^, ^15^ ^]^ With regard to an arylation of tyrosine residues in peptides under biomimetic conditions, the applied diazonium salt would however need to be sufficiently electron‐deficient to form excitable CT complexes, but should also be sufficiently stable in water to avoid competing nucleophilic substitution in case of too strong activation.^[^ ^16^, ^17^ ^]^

## 2 Results and Discussion

In this work, we now demonstrate that such ideal reaction conditions, requiring only the peptide substrate, a diazonium salt, and visible light can indeed be put into practice to achieve a highly convenient arylation of tyrosine residues (Scheme 1C).

We started our investigations with readily water‐soluble tyramine hydrochloride (**1a**) and 3,4,5‐trifluorophenyldiazonium chloride (**2a**), whereat the diazonium salt was chosen so that a CT interaction could be expected due to trifluoro substitution, but the salt would still be sufficiently stable towards nucleophilic aromatic substitution in aqueous solution. Besides irradiation with visible light from a blue LED to achieve initiation, we used a novel biphasic reaction mixture to continuously extract strongly light‐absorbing by‐products into the organic phase. The organic solvent *t*BuOMe (MTBE) was thereby chosen due to comparably low hydrogen abstraction by aryl radicals. In this way, and as shown by preliminary experiments, initiation and aryl radical formation are strongly improved, especially in later stages of the reaction course, where azo compounds (e.g., resulting from homocoupling of the diazonium salt) typically slow down product formation. Selected results from the study aimed at optimization are summarized in Table 1 (see Supporting Information Table S1 for further data).

**Table 1:** Optimization of reaction conditions.^[^ ^a^ ^]^

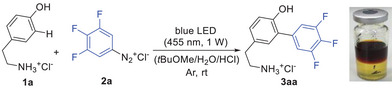		
Entry	Equivalents 2a and reaction time^[^ ^b^ ^]^	Yield 3aa [%]^[^ ^c^ ^]^
1	0.75 (1 h)	28
2	0.75 (2 h)	38
3	0.75 (3 h)	31
4	1.1 (2 h)	38
5	1.5 (2 h)	39
6	1.1 + 0.75 (2 + 2 h)	50
7	1.1 + 0.75 + 0.75 (2 + 2 + 2 h)	56
8	1.1 + 0.75 + 0.75 (3 + 3 + 3 h)	59

^[a]^ 3,4,5‐Trifluorophenyldiazonium chloride (**2a**) previously prepared from 3,4,5‐trifluoroaniline using sodium nitrite in aqueous hydrochloric acid.

^[b]^ Concentration of HCl (remaining from diazotization) in the reaction mixture: 0.20.3 M.

^[c]^ Yields determined by ^1^H NMR spectroscopy using 1,3,5‐trimethoxybenzene as internal standard after aqueous work‐up.

While a reaction time of 2 h turned out to be superior to shorter (1 h) or longer durations (3 h) (entries 1–3), no further increase in yield could be achieved by raising the amount of diazonium salt **2a** from initially 0.75 to 1.1 or 1.5 equivalents (entries 2, 4, 5). On this basis, the addition of a second or even third batch of **2a** appeared reasonable, which then indeed led to yields of up to 59% of **3aa** under attractive reaction conditions and without the formation of major tyramine‐derived side products (entries 7, 8). Irradiation of **2a** in the absence of tyramine **1a** did not lead to any conversion, as no excitable CT complex can be formed.

**Scheme 2:**
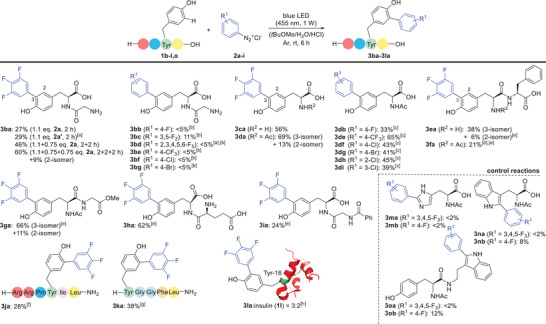
Trifluoroarylation of tyrosine residues in peptides **1b–k** (1 mmol) and **2a–g** (1.1 + 0.75 + 0.75 mmol, 3.8 + 2.5 + 2.5 mL of a previously prepared 0.3 M diazonium chloride solution) irradiated with a blue LED (455 nm, 1W) for 2 + 2 + 2 h at rt. Yields determined by 1H NMR spectroscopy using maleic acid or 1,3,5‐trimethoxybenzene as an internal standard. [a] Tetrafluoroborate (1.5 equiv.) was used instead of diazonium chloride. [b] Performed with 1.5 equiv. of diazonium salt **2b‐2g** at a reaction time of 2 h. [c] **1d** (1 mmol) and **2b‐I** (1.0 + 1.0 mmol) irradiated with a blue LED (455 nm, 1W) for 3 + 3 h at rt. [d] Complicated by low solubility of the peptidic substrate (reaction time 2 + 2 h). [e] 0.5 mmol of **1e‐i** was used. [f] 0.1 mmol of **1j** was used. [g] 0.09 mmol of **1k** was used. [h] Insulin (**1l**) (5.00 µmol).

Due to the slightly more convenient protocol, the conditions shown in entry 7 (Table 1) were finally selected for an investigation of scope and limitations (Scheme 2). Using the arylation of H‐Gly‐Tyr‐OH (**1b**) with 3,4,5‐trifluorophenyl‐diazonium **2a** to give **3ba**, we could at first confirm the superiority of the batchwise addition of a total of 2.6 equivalents of **2a** (1.1 + 0.75 + 0.75), and the storable 3,4,5‐trifluorophenyldiazonium tetrafluoroborate turned out to be equally well suited at a short reaction time of 2 h. In a second series of experiments, H‐Gly‐Tyr‐OH (**1b**) was employed to reinvestigate the effect of the degree of fluoro substitution on the diazonium salt, whereat the 3,4,5‐trifluoro pattern was shown to be ideally suited compared to lower and higher fluoro substitution (c.f. products **3bb-3bd**) as well as to 4‐trifluoromethyl‐, 4‐chloro‐, or 4‐bromo‐substituted diazonium salts (c.f. products **3be–3bg**).

Inspired by the good yield then obtained for **3ca** [56% from H‐Tyr‐OH (**1c**)] and the even better yield for **3** **da** [69% from Ac‐Tyr‐OH (**1d**)], we turned our attention again to differently substituted diazonium salts (R^1^ = 4‐F, 4‐CF_3_, 4‐Cl, 4‐Br, 2‐Cl, 3‐Cl), which now gave useful yields of **3db–3di** ranging from 33% to 65%.

Comparing the two substrates H‐Gly‐Tyr‐OH (**1b**) and Ac‐Tyr‐OH (**1d**), of which the former is protonated under the acidic reaction conditions while the latter remains uncharged, points to a strong influence of the positive charge on CT complex formation. Due to its unique properties (see also below for further evidence), the 3,4,5‐trifluorophenyldiazonium ion **2a** is able to overcome the repulsive effect of the positive charge while other diazonium ions such as **2b** (R^1^ = 4‐F) or **2f** (R^1^ = 4‐Cl) require an uncharged substrate such as Ac‐Tyr‐OH (**1d**) for sufficiently strong CT complex formation and successful radical initiation.

Most of the further substrates H‐Tyr‐Phe‐OH (**1e**), Ac‐Tyr‐Phe‐OH (**1f**), Ac‐Tyr‐Gly‐OMe (**1** **g**), H‐Glu‐Tyr‐OH (**1** **h**), and Bz‐Gly‐Tyr‐OH (**1i**) gave useful to good yields of the desired arylation products, whereat difficulties were only encountered in one case due to low solubility (dipeptide **1f**).

The formation of 2‐isomers,^[^ ^18^ ^]^ which was observed for substrates **1b**, **1d**, **1e,** and **1 g**, points to a certain influence of the polarity of the peptide structure, especially when compared to the closely related substrates **1c** and **1f**, which did not lead to significant regioisomers. Generally, we assume that more or less small amounts of the 2‐isomer are formed in all reactions, but the quantities may occasionally be too small to allow detection or isolation. For all cases, in which the 2‐isomer could be isolated (see above), a ratio better than 5:1 in favor of the 3‐isomer was determined. Competing arylation on additional phenyl rings, as it is possible for peptide substrates **1e**, **1f**, **1i,** and **1k,** could be detected by LC‐MS analysis, but the trace amounts of the related regioisomers only allowed for their isolation in one case (**3ea’’**).

The synthetic power of the newly developed methodology becomes obvious from the successful arylations of the hexapeptide NT(8–13) (**1j**), Leu‐enkephalin (**1k**), and insulin (**1l**), whereat related products have hitherto not been accessible in one single step under such simple reaction conditions. Insulin (**1l**), which contains four tyrosine units in its A and B chains, was arylated with good conversion (ca. 60%) and high selectivity for tyrosine‐16 in the B chain, as shown by subsequent enzymatic cleavage and fragment analysis by LC‐MS. Notably, this result differs from the selectivity observed in trifluoromethylations,^[^ ^11^, ^12^ ^]^ where tyrosine‐19 in the A chain was preferably attacked.

Control reactions with Ac‐His‐OH (**1 m**) revealed that this amino acid shows almost no conversion with **2a** (R^1^ = 3,4,5‐F_3_) and **2b** (R^1^ = 4‐F) under the typical reaction conditions, which is most probably due to protonation of the imidazole ring and insufficient CT complex formation (Scheme 2, bottom right). The observation that experiments with Ac‐Trp‐OH (**1n**) are strongly hindered by competing azo coupling is in full agreement with a recent study by Santos^[^ ^15^ ^]^ and the related arylation products **3na** and **3** **nb** were only obtained in traces or minor amounts, respectively (see Supporting Information for representative azo compounds). Competing azo coupling also hindered the arylation of tyrosine tryptamide **1o** leading to trace amounts of **3oa** and a low yield of **3ob** (12%). In turn, the presence of a tryptophan unit represents a limitation of the arylation described herein, not only due to competing azo coupling, but also due to preferred arylation of the tryptophan's indole over the tyrosine's phenol (c.f. **3ob**). Histidine residues, in contrast, are not likely to be detrimental, as it was earlier proven by the successful arylation of insulin (**3l**).

**Scheme 3:**
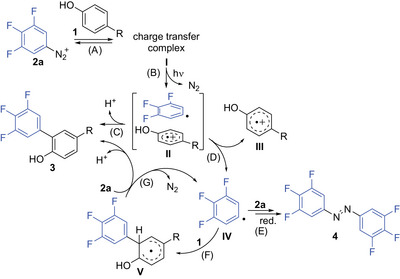
Plausible mechanistic pathways.

Taking into account all results summarized in Scheme 2, we then turned to the question why the 3,4,5‐trifluorophenyldiazonium displays such unique properties and even enables arylations of positively charged substrates with presumably unfavorable CT complex formation (c.f. reactions from **1b** and **1d**, Scheme 2). Regarding the plausible mechanistic steps depicted in Scheme 3, the strength and optical excitability of the CT complex **I** formed from **2a** and phenol **1** (step (A)) assumingly plays a major role.

**Figure 1:**
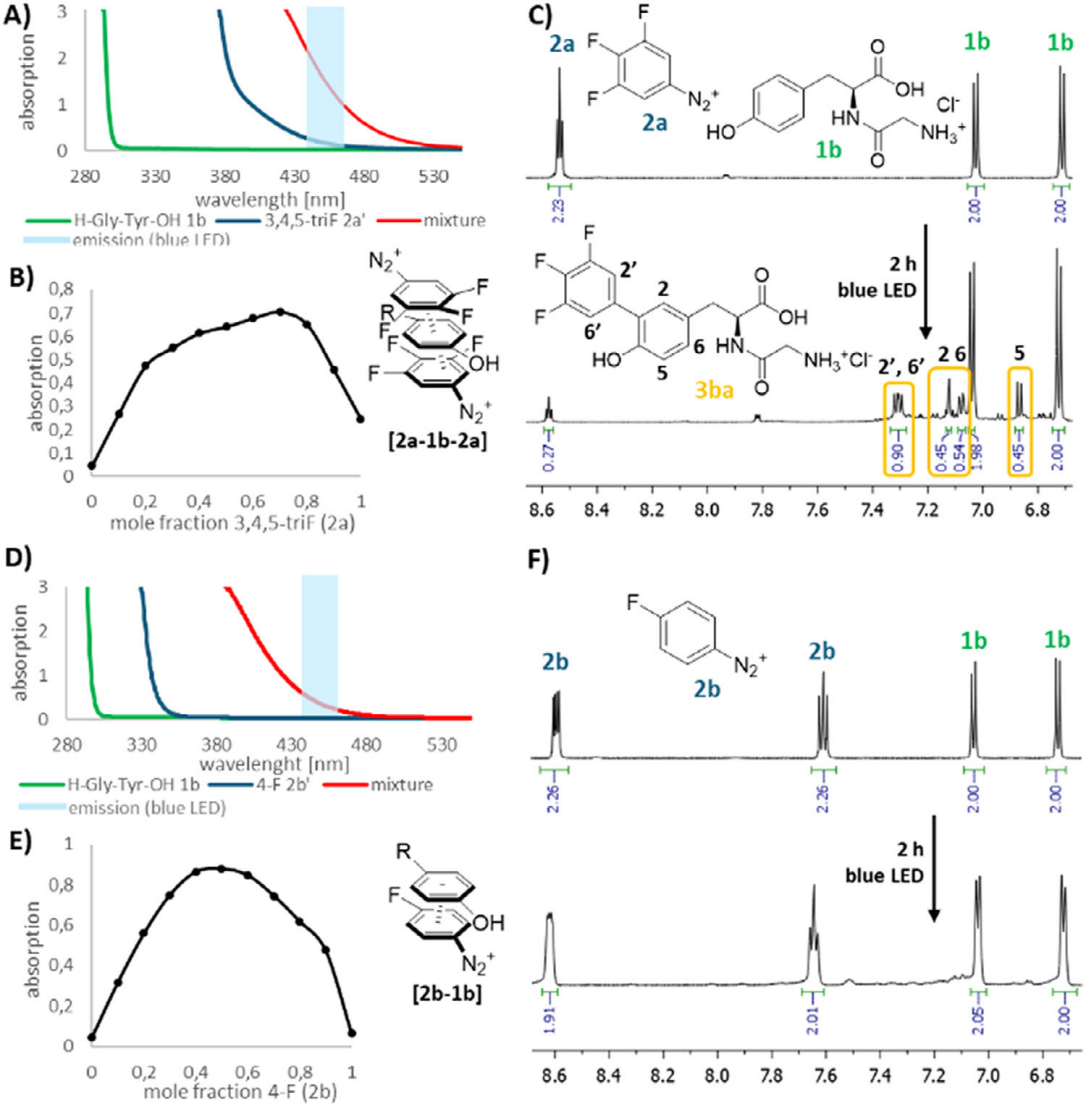
Comparison of CT complex formation and conversions of 3,4,5‐trifluorophenyldiazonium **2a** and 4‐fluorophenyldiazonium **2b** with the positively charged substrate H‐Gly‐Tyr‐OH (**1b**) under visible light irradiation.

A comparison of the CT arising from the positively charged substrate H‐Gly‐Tyr‐OH (**1b**) and 3,4,5‐trifluorophenyldiazonium **2a**, and **1b** and 4‐fluorophenyldiazonium **2b** confirmed a much stronger absorption of the triflluorophenyldiazonium complex in the emission range of the blue LED (455 ± 10 nm) (Figures 1A,D).

This can nicely be rationalized by the preferred 2:1 stoichiometry of CT complex **[2a‐1b‐2a]** compared to **[2b‐1b]**, which became apparent upon Job's plot analysis (Figures 1B,E).^[^ ^19^, ^20^ ^]^ As a result, more or less clean formation of **3ba** takes place under blue LED irradiation while the monofluorinated biaryl **3bb** only occurs in traces after 2‐hour reaction time (Figures 1C,F). Besides the good optical excitability of the ternary CT complex **I** to give the radical‐radical cation pair **II** (Scheme 3, step B), we reasoned that a further advantage of **2a** might arise from the 3,4,5‐trifluoro substitution of the related aryl radical **IV** and the concomitant properties.^[^ ^21^, ^22^ ^]^ In particular, the trifluoro substitution of **IV** could lead to a comparably high stability of the radical–radical ion pair **II** and formation of biaryl **3** via a direct radical combination in the solvent shell (step (C)).^[^ ^23^ ^]^ Although the effect of 3,4,5‐trifluoro substitution on the radical–radical ion pair **II** is difficult to determine experimentally, the related diazonium salt **2a** showed surprisingly high R_f_ values in TLC experiments (see Figure S13, Supporting Information), which provides indirect support for the assumption that the particular water repellency^[^ ^24^ ^]^ of the 3,4,5‐trifluorophenyl unit may lead to a longer lifetime of **II**, especially in an aqueous environment.

That direct radical combination from complex **II** is, however, not the only pathway, and dissociation to radical cation **III** and aryl radical **IV** does occur (step (D)), could be shown in a competition experiment, in which non‐CT‐complex forming nitrobenzene was added to the reaction mixture containing Ac‐Tyr‐OH (**1d**) and **2a** (see Scheme S1, Supporting Information). In this experiment, the tyrosine arylation product **3** **da** was formed in 20% yield and 3,4,5‐trifluoro‐4′‐nitrobiphenyl in 4% yield after a short reaction time of 2 hours, which demonstrates at least some dissociation of the radical–radical cation pair **II**. The assumption that step (C) is of importance compared to the alternative pathway via steps (D), (F), and (G), is further supported by the unexpectedly low extent of homocoupling to give hexafluoroazobenzene **4** (step (E)),^[^ ^24^ ^]^ where **4** could only be detected in trace amounts although comparably high concentrations of **2a** were generally present in the reaction mixture. Homocoupling to **4** thereby proceeds via the attack of aryl radical **IV** onto **2a** and a further reductive step.^[^ ^25^ ^]^

Regarding step (F), aryl radical addition to phenols **1** can be considered as 5–10 times faster than the addition to unsubstituted benzene (*k* ∼ 5 × 10^5^ M^−1^s^−1^),^[^ ^26^ ^]^ which is in agreement with the selectivity observed for the phenolic tyrosine side chain over other phenyl moieties present in some of the peptide substrates (Scheme 2). Rearomatization of adduct **V** (step (G)) is possible by hydrogen atom transfer from **V** to oxygen, if the arylation is conducted under air,^[^ ^27^ ^]^ but since argon was used in the present case, mainly rearomatization by single electron transfer to **2a** combined with chain propagation appears plausible.^[^ ^13^, ^28^ ^]^ In this context, a comparison of the oxidation potentials of 3,4,5‐trifluorophenyldiazonium **2a,** and 4‐fluorophenyldiazonium **2b** by differential pulse voltammetry (DPV) revealed values of + 0.7 V and + 0.4 V,^[^ ^29^ ^]^ respectively, whereat the higher potential of **2a** certainly facilitates step (G) and can as well be beneficial for step (B).

**Scheme 4:**
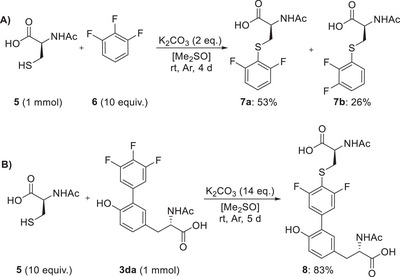
Nucleophilic substitution of the 3,4,5‐trifluorophenyl moiety.

Having investigated the synthetic potential and the unique mechanistic background of tyrosine arylation with 3,4,5‐trifluorophenyldiazonium, we finally turned to the question whether further functionalization based on nucleophilic substitution of the trifluorophenyl moiety might be possible. Related reactions with decafluorobiphenyl have recently become highly popular,^[^ ^1^, ^30^ ^]^ but due to absence in literature,^[^ ^31^ ^]^ we assumed that the 3,4,5‐trifluorophenyl subunit might not be sufficiently activated to undergo substitutions under mild conditions. A preliminary experiment then however revealed that a substitution of 1,2,3‐trifluorobenzene (**6**) by Ac‐Cys‐OH (**5**) can indeed be achieved under mild conditions (Scheme 4A), where a 2:1 mixture of regioisomers **7a** and **7b** was obtained.

Transferring the reaction conditions to the previously obtained trifluorophenyl tyrosine **3** **da**, with an inverted excess of Ac‐Cys‐OH (**5**) over **3** **da**, then furnished the only regioisomer **8** in a good yield of 83% (Scheme 4B). These findings point to the fact that 3,4,5‐trifluorophenylated tyrosines are likely to show sufficient reactivity to undergo coupling reactions to cysteine residues, which appears even more favored in an intramolecular peptide‐like setup than in the above shown intermolecular substitution.

## 3 Conclusion

In summary, it has been shown that a well‐balanced substitution on a phenyldiazonium ion–regarding strong CT complex formation and stability in water–can enable a direct functionalization of tyrosine residues in peptides. Further taking advantage of a biphasic setup and the beneficial formation of a ternary, sandwich‐like CT complex between 3,4,5‐trifluorophenyldiazonium **2a** and the phenolic side chain of tyrosine, the arylation of various peptides–up to such complex substrates as insulin –could be realized under visible light irradiation, exceptionally mild conditions and without any additional additives or catalysts. In combination with the option of further functionalization via nucleophilic substitution, these results reveal a much greater potential of radical arylations in peptide functionalization than hitherto assumed. Experiments toward the extension to additional diazonium salts and medicinal as well as applications in MRT imaging^[^ ^32^ ^]^ are currently underway in our laboratory.

## 4 Supporting Information

Detailed experimental procedures and characterization of newly obtained compounds. The authors have cited additional references within the Supporting Information.^[^ ^33^, ^34^, ^35^, ^36^, ^37^, ^38^, ^39^ ^]^

## 6 Conflict of Interest

The authors declare no conflict of interest.

## Supporting information



Supporting Information

## Data Availability

The data that support the findings of this study are available in the supplementary material of this article.
