# Supplementary material for: 3,4,5‐Trifluorophenyldiazonium–A Unique Radical Source for the Visible Light Induced, Catalyst‐Free Arylation of Tyrosine Residues in Peptides
Source: Chemistry. 2025 Jun 18;31(38):e202501160. doi: 10.1002/chem.202501160 (PMC12238915; doi:10.1002/chem.202501160)
Supplement: Supplementary file 1 — Supporting Information [file CHEM-31-e202501160-s001.pdf]

Supporting Information

3,4,5-Trifluorophenyldiazonium – a unique radical source for the visible light induced, catalyst-free arylation of tyrosine residues in peptides

Meret K. Kuschow, Daniel R. Troeger, Luca Sophie Schuster, Leonard Bock, Leon A. Zähnle, Markus R. Heinrich*

Department of Chemistry and Pharmacy, Pharmaceutical Chemistry
Friedrich-Alexander-Universität Erlangen-Nürnberg
Nikolaus-Fiebiger-Str. 10, 91058 Erlangen, Germany
E-mail: markus.heinrich@fau.de

Contents

1. Materials and methods 3
2. Synthesis of starting material 5
   2.1 Synthesis of substrates 5
   2.2 Synthesis of diazonium salts 13
3. Optimization experiments with tyramine hydrochloride 20
4. Scope and limitations 23
   4.1 Assignment of regioisomers 23
   4.2 Variation of reaction time and equivalents 24
   4.3 Variation of the substitution pattern of the phenyldiazonium salt 26
   4.4 Variation of peptide 33
   4.5 Arylation of insulin 48
   4.6 Control reactions with other aromatic amino acids 53
5. Additional experiments on mechanistic background 59
   5.1 Analysis of CT complex formation 59
   5.2 Job plot measurements 62
   5.3 TLC experiments with different diazonium salts 64
   5.4 Competition experiment with nitrobenzene 64
   5.5 Differential pulse voltammetry 66
6. Functionalization by nucleophilic substitution 68
7. ^{1}H NMR and DEPTQ spectra of synthesized compounds 71
8. References 107

1. Materials and methods

Solvents and reagents were obtained from commercial sources and used as received. The peptides **1f**, **1g**, **1i**, **1j**, **1k** and **1l** were synthesized using the respective methods. Starting material **1d** was purchased from Chempur and peptides **1b**, **1e** and **1h** were purchased from Bachem. Insulin was purchased from Merck. ^{1}H NMR and ^{13}C NMR spectra were recorded on a Bruker Avance 600 (^{1}H: 600 MHz; ^{13}C: 151 MHz) or a Bruker Avance 400 (^{1}H: 400 MHz; ^{13}C: 101 MHz; ^{19}F: 377 MHz) spectrometer. For ^{1}H NMR spectra $CDCl_3$, CD_3OD, D_2O, CD_3CN and $(CD_3)_2SO$ were used as solvents referenced to $CDCl_3$ (7.26 ppm), CD_3OD (3.31 ppm), D_2O (4.79 ppm), CD_3CN (1.94 ppm) and $(CD_3)_2SO$ (2.50 ppm). Chemical shifts are reported in parts per million (ppm). Coupling constants are in Hertz (Hz). The following abbreviations are used for the description of signals: s (singlet), d (doublet), t (triplet), q (quartet) and m (multiplet). ^{13}C NMR spectra were recorded in DEPTQ mode (Distorsionless Enhancement by Polarization Transfer with retention of Quaternaries) using CD_3OD or $(CD_3)_2SO$ as solvents with CD_3OD (49.00 ppm) and $(CD_3)_2SO$ (39.52 ppm) as reference. C_q and CH_2 signals are negative and CH and CH_3 signals positive. For ^{19}F NMR CCl_3F (0.00 ppm) was used as standard. High resolution mass spectra were recorded on Bruker micrOTOF Dottonik using electron spray ionization (ESI), Atmospheric Pressure Photoionization (APPI) and a sector field mass analyzer or time of flight (TOF). Analytical thin-layer chromatography (TLC) was carried out on Merck silica gel plates using short wave (254 nm) UV light to visualize components. Silica gel (Kieselgel 60, 40−63 mm, Merck) was used for flash column chromatography. IR spectra were recorded on JASCO FT/IR-4100 as KBr pellets and are expressed in cm^{-1}. Optical rotation was measured on a JASCO P-2000 polarimeter. For preparative HPLC an Agilent 1100 Preparative Series (system A), equipped with a VWD-detector and a column from Macherey-Nagel Varioprep VP 250/32 Nucleodur C18 HTec with a flow rate of 30 ml/min or a Knauer Azura (system B), equipped with a MWD-detector and a Phenomenex Kinetex C18 column, 100 × 21.2 nm with a flow rate of 30 ml/min were applied. For analytical measurement of the enzymatic cleavages an Agilent Infinity II, equipped with a MWD-detector and an Agilent Kinetex C18 column 50 × 2.1 mm with a flow rate of 0.4 mL/min and an Agilent SingleQuad ESI-MS were applied. UV-Vis spectra were measured with a Specord 200 Plus device. Irradiation experiments were carried out in a Photoreactor TAK120 MK1 LC by HK Testsysteme GmbH with blue LEDs (455 nm, 1 W) at room temperature.

a.

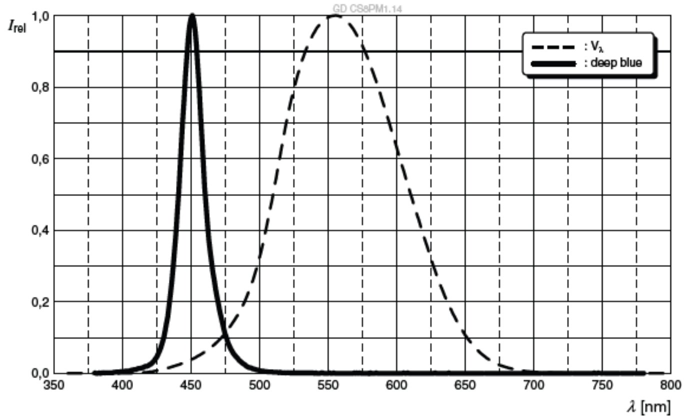

b.

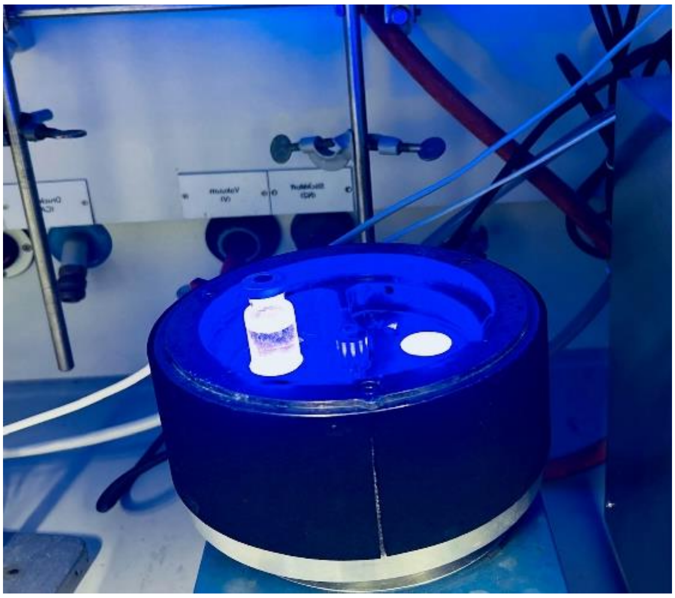

Figure S1. a. Emission spectrum of blue LED light source used in the photoreactor TAK120 MK1 LC, provided by the company. **b.** Irradition experiment setup of the photoreactor TAK120 MK1 LC.

2. Synthesis of starting material

2.1 Synthesis of substrates

General procedure for liquid phase peptide synthesis (GP1)

The corresponding acid (1.0 equiv.) and amine (0.85 – 1.0 equiv.) were dissolved in dichloromethane and/or *N*,*N*-dimethylformamide under nitrogen. 1-Hydroxy-1*H*-benzotriazole (1.1 equiv.), 2-(1*H*-benzotriazole-1-yl)-1,1,3,3-tetramethylaminium hexafluorophosphate (1.1 equiv.) and triethylamine were added and the mixture was stirred over night at room temperature. Ethyl acetate was added and the organic phase was washed with 1 M HCl and saturated Na_2CO_3 solution. The combined organic layers were dried over Na_2SO_4 and the solvent was removed under reduced pressure. Liquid phase peptide synthesis was performed according to a procedure from literature.[2]

General procedure for solid phase peptide synthesis (GP2)

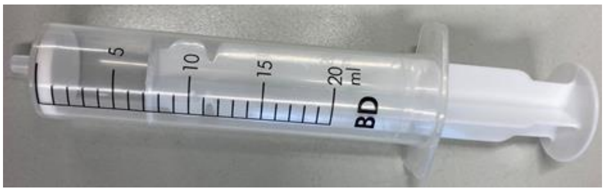

Figure S2. 20 mL reactor used for solid phase peptide synthesis.

Peptides were synthesized on TentaGel SRAM resin (2.00 g with a loading capacity of 0.23 mmol/g, 0.46 mmol, 1.0 equiv.) as C-terminal amides using Fmoc/*t*Bu-based solid phase synthesis in 20 mL reactors (**Figure S2**). All used amino acids had the *L*-configuration. For each amino acid, a coupling cycle consisting of Fmoc-deprotection, amino acid coupling and capping was performed. Before starting with the first Fmoc-deprotection, the resin was swelled with 10 mL of CH_2Cl_2 for 2 min. Before cleaving the peptide from the resin, an additional Fmoc-deprotection step was performed to receive the free amine. The solid phase peptide synthesis was performed according to procedures from literature.[3]

<u>Fmoc-deprotection:</u>

After incubating the resin for 5 min with 10 mL of 20% (*v/v*) piperidine solution in *N*,*N*-dimethylformamide, the reactor was washed with *N*,*N*-dimethylformamide (3 × 10 mL).

Amino acid coupling:

The corresponding Fmoc-protected amino acid (2.30 mmol, 5 equiv.) was dissolved in a solution of 2-(6-chloro-1*H*-benzotriazol-1-yl)-1,1,3,3-tetramethylaminium-hexafluoro-phosphate (933 mg, 2.30 mmol, 4.9 equiv.) in 10 mL of *N*,*N*-dimethylformamide. For activation, *N*-methylmorpholine (1.0 mL, 931 mg, 9.20 mmol, 20 equiv.) was added to the amino acid solution and the resin was incubated with this mixture for 10 min. For all amino acids these steps were repeated 3 times, except Fmoc-Arg(Pbf)-OH was only coupled twice. After finished coupling, the resin was washed with *N,N*-dimethylformamide (3 × 10 mL).

Capping:

The resin was incubated with a mixture of acetic anhydride (1.7 mL), pyridine (3.3 mL) and *N*,*N*-dimethylformamide (5 mL) for 2 min and washed with *N*,*N*-dimethylformamide (5 × 10 mL).

Cleavage:

Peptides were cleaved from the resin with 3 × 10 mL of a mixture of trifluoroacetic acid (8 mL), water (0.5 mL), phenol (0.5 g), thioanisole (0.5 mL), triisopropylsilane (0.5 mL), incubating each time for 2 h. The combined reactions mixtures (30 mL), from which the resin was separated by filtration, were added to 150 mL of cold methyl *tert*-butyl ether (-20 °C) and 30 mL of water. After mixing, the biphasic mixture was cooled to -20 °C over night. The supernatant methyl *tert*-butyl ether phase was decanted and the frozen water phase was lyophilized. The crude peptides were purified by preparative HPLC.

***N*-Acetyl-*L*-tyrosyl-*L*-phenylalanine (1f) (Ac-Tyr-Phe-OH)**

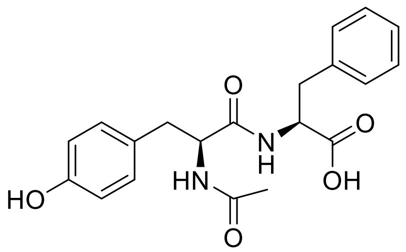

$C_{20}H_{22}N_2O_5$
370.41 g/mol

Compound **1f** was synthesized from (*S*)-phenylalanine *tert*-butyl ester hydrochloride (885 mg, 3.40 mmol, 0.85 equiv.) and *N*-acetyl-*L* tyrosine (**1d**) (893 mg, 4.00 mmol, 1 equiv.) with triethylamine (1.70 mL, 1.21 g, 12.0 mmol, 3 equiv.) following **GP1**. 10 mL of dichloromethane and 9 mL of *N*,*N*-dimethylformamide were used as solvents. Afterwards, the crude mixture was stirred with triisopropyl silane (2.1 mL, 1.58 g, 10.0 mmol, 2.5 equiv.), trifluoroacetic acid (15 mL, 23 g, 0.2 mol, 50 equiv.), phenol (1.88 g, 20.0 mmol, 5 equiv.) and water (0.55 mL, 541 mg, 30.0 mmol, 7.5 equiv.) for 5 h at room temperature. Water (150 mL) was added to the reaction mixture and washed with hexane (3 × 60 mL). The crude mixture was dried via lyophilization and purified by preparative HPLC (system B, gradient: 20% → 31% acetonitrile in water, 0.1% trifluoroacetic acid) to give **1f** as colorless powder.

t_R 5.12 min (system B, 20% → 31% acetonitrile in water, 0.1% trifluoroacetic acid).

^{1}H NMR (400 MHz, CD_3OD) δ (ppm) = 7.30 – 7.23 (m, 2H), 7.22 – 7.16 (m, 3H), 7.05 – 6.99 (m, 2H), 6.69 – 6.63 (m, 2H), 4.61 (dd, *J* = 7.9, 5.2 Hz, 1H), 4.53 (dd, *J* = 9.4, 5.2 Hz, 1H), 3.20 (dd, *J* = 13.9, 5.2 Hz, 1H), 3.06 – 2.93 (m, 2H), 2.69 (dd, *J* = 14.1, 9.5 Hz, 1H), 1.85 (s, 3H).

^{13}C NMR (101 MHz, CD_3OD) δ (ppm) = 174.2 (C_q), 173.6 (C_q), 173.0 (C_q), 157.2 (C_q), 138.2 (C_q), 131.2 (2 × CH), 130.4 (2 × CH), 129.4 (2 × CH), 129.1 (C_q), 127.8 (CH), 116.1 (2 × CH), 56.1 (CH), 55.0 (CH_3), 38.3 (CH_2), 37.9 (CH_2), 22.3 (CH_3).

HRMS (ESI) (*m/z*) calcd. for $C_{20}H_{23}N_2O_5$ [M+H$^+$]: 371.1602, found: 371.1614.

Methyl *N*-acetyl-*L*-tyrosylglycinate (1g) (Ac-Tyr-Gly-Me)

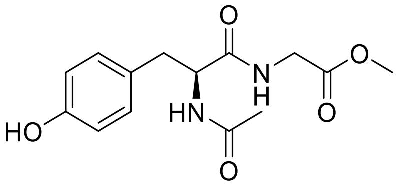

$C_{14}H_{18}N_2O_5$
294.31 g/mol

Compound **1g** was synthesized from glycine methyl ester hydrochloride (1.26 g, 10.0 mmol, 1 equiv.) and *N*-acetyl-*L* tyrosine (2.23 g, 10.0 mmol, 1 equiv.) with triethylamine (4.2 mL, 3.04 g, 30.0 mmol, 3 equiv.) following **GP1**. 25 mL of dichloromethane and 10 mL of *N,N*-dimethylformamide were used as solvents. The crude mixture was purified by preparative HPLC (system A, gradient: 12% → 27% acetonitrile in water, 0.1% formic acid) to give **1g** as colorless powder.

t_R 5.65 min (system A, 12% → 27% acetonitrile in water, 0.1% formic acid).

^{1}H NMR (400 MHz, CD_3OD) δ (ppm) = 7.13 – 6.98 (m, 2H), 6.77 – 6.62 (m, 2H), 4.58 (dd, *J* = 9.2, 5.5 Hz, 1H), 3.92 (s, 2H), 3.71 (s, 3H), 3.07 (dd, *J* = 14.0, 5.5 Hz, 1H), 2.77 (dd, *J* = 14.0, 9.2 Hz, 1H), 1.90 (s, 3H).

^{13}C NMR (101 MHz, CD_3OD) δ (ppm) = 174.4 (C_q), 173.2 (C_q), 171.6 (C_q), 157.3 (C_q), 131.3 (2 × CH), 129.2 (2 × CH), 116.2 (2 × CH), 56.3 (CH), 52.6 (CH_3), 41.9 (CH_2), 38.2 (CH_2), 22.5 (CH_3).

HRMS (ESI) (*m/z*) calcd. for $C_{14}H_{19}N_2NaO_5$ [M+Na$^+$]: 317.1108, found: 317.1107.

Benzoylglycyl-*L*-tyrosine (1i) (Bz-Gly-Tyr-OH)

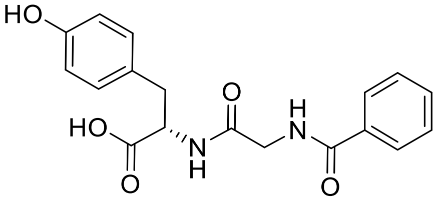

$C_{18}H_{18}N_2O_5$
342.35 g/mol

Compound **1i** was synthesized from *L*-tyrosine *tert*-butyl ester (950 mg, 4.00 mmol, 1 equiv.) and hippuric acid (717 mg, 4.00 mmol, 1 equiv.) with triethylamine (1.1 mL, 810 mg, 8.00 mmol, 2 equiv.) following the **GP1**. 10 mL of dichloromethane was used as solvent. Afterwards, the crude mixture was stirred with triisopropylsilane (2.1 mL, 1.58 g, 10.0 mmol, 2.5 equiv.), trifluoroacetic acid (15 mL, 22.8 g, 200 mmol, 50 equiv.), phenol (1.9 g, 20.0 mmol, 5 equiv.) and water (0.55 mL, 541 mg, 30.0 mmol, 7.5 equiv.) for 5 h at room temperature. Water (150 mL) was added to the reaction mixture and washed with hexane (3 × 600 mL). The crude mixture was dried via lyophilization and purified by preparative HPLC (system B, gradient: 15% → 30% acetonitrile in water, 0.1% trifluoroacetic acid) to give **1i** as colorless powder.

t_R 5.03 min (system B, 15% → 30% acetonitrile in water, 0.1% trifluoroacetic acid).

^{1}H NMR (400 MHz, CD_3OD) δ (ppm) = 7.90 – 7.80 (m, 2H), 7.59 – 7.52 (m, 1H), 7.51 – 7.44 (m, 2H), 7.04 – 6.98 (m, 2H), 6.66 – 6.61 (m, 2H), 4.65 (dd, *J* = 7.7, 5.2 Hz, 1H), 4.12 – 3.96 (m, 2H), 3.08 (dd, *J* = 14.0, 5.2 Hz, 1H), 2.94 (dd, *J* = 14.0, 7.7 Hz, 1H).

^{13}C NMR (101 MHz, CD_3OD) δ (ppm) = 174.5 (C_q), 171.4 (C_q), 170.5 (C_q), 157.4 (C_q), 135.0 (C_q), 133.0 (CH), 131.4 (2 × CH), 129.6 (2 × CH), 128.6 (C_q), 128.5 (2 × CH), 116.2 (2 × CH), 55.1 (CH), 43.9 (CH_2), 37.6 (CH_2).

HRMS (ESI) (*m/z*) calcd. for $C_{18}H_{19}N_2O_5$ [M+H$^+$]: 343.1289, found: 343.1286.

(*S*)-1-(*L*-Arginyl-*L*-arginyl)-*N*-((*S*)-1-{[(2*S*,3*S*)-1-{[(*S*)-1-amino-4-methyl-1-oxopentan-2-yl]amino}-3-methyl-1-oxopentan-2-yl]amino}-3-(4-hydroxyphenyl)-1-oxopropan-2-yl)pyrrolidine-2-carboxamide (NT(8-13)) (1j)

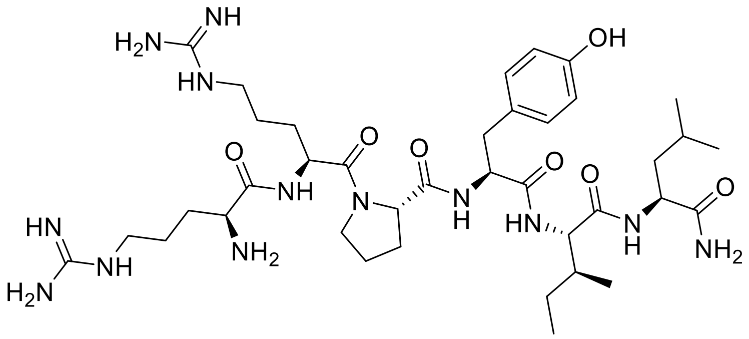

$C_{38}H_{65}N_{13}O_7$
816.02 g/mol

Compound **1j** was prepared according to **GP2**. For amino acid coupling the following amino acids were used in this order: Fmoc-Leu-OH (813 mg, 2.30 mmol, 5 equiv.), Fmoc-Ile-OH (813 mg, 2.30 mmol, 5 equiv.), Fmoc-Tyr(*t*Bu)-OH (1.06 g, 2.30 mmol, 5 equiv.), Fmoc-Pro-OH*H_2O (817 mg, 2.30 mmol, 5 equiv.), Fmoc-Arg(Pbf)-OH (1.49 g, 2.30 mmol, 5 equiv.) Fmoc-Arg(Pbf)-OH (1.49 g, 2.30 mmol, 5 equiv.). The crude peptide was purified by preparative HPLC (system B, gradient: 5% → 35% acetonitrile in water, 0.1% trifluoroacetic acid) to give **1j** as colorless powder.

***t*R** 5.94 min (system B, 5% → 35% acetonitrile in water, 0.1% trifluoroacetic acid).

^{1}H NMR (600 MHz, CD_3OD) δ (ppm) = 7.13 – 7.00 (m, 2H), 6.70 (dd, *J* = 8.9, 2.5 Hz, 2H), 4.61 (dd, *J* = 7.3, 4.7 Hz, 1H), 4.54 (dd, *J* = 7.8, 6.1 Hz, 1H), 4.40 (td, *J* = 9.7, 9.1, 5.2 Hz, 2H), 4.19 (d, *J* = 7.7 Hz, 1H), 3.99 (t, *J* = 6.1 Hz, 1H), 3.84 (dt, *J* = 9.9, 6.9 Hz, 1H), 3.64 (dt, *J* = 9.7, 6.9 Hz, 1H), 3.20 (t, *J* = 7.1 Hz, 4H), 3.06 (dd, *J* = 14.2, 5.8 Hz, 1H), 2.88 (dd, *J* = 14.2, 8.0 Hz, 1H), 2.17 (ddd, *J* = 15.5, 12.1, 7.7 Hz, 1H), 2.06 – 1.46 (m, 16H), 1.21 – 1.10 (m, 1H), 0.97 (d, *J* = 6.5 Hz, 3H), 0.92 (dd, *J* = 6.6, 1.9 Hz, 6H), 0.89 (t, *J* = 7.5 Hz, 3H).

^{13}C NMR (101 MHz, CD_3OD) δ (ppm) = 177.1 (C_q), 174.0 (C_q), 173.5 (C_q), 173.3 (C_q), 172.3 (C_q), 170.0 (C_q), 158.73 (C_q), 158.67 (C_q), 157.3 (C_q), 131.4 (2 × CH), 128.7 (C_q), 116.3 (2 × CH), 61.7 (CH), 59.3 (CH), 56.3 (CH), 53.6 (CH), 52.8 (CH), 52.7 (CH), 42.11 (CH_2), 42.05 (CH_2), 41.8 (CH_2), 38.2 (CH), 37.7 (CH_2), 30.5 (CH_2), 29.6 (CH_2), 29.0 (CH_2), 26.0 (CH_2), 25.8 (CH_2), 24.9 (2 × CH_2), 23.5 (CH), 22.0 (2 × CH_3), 15.9 (CH_3), 11.3 (CH_3). One carbon signal missing.

HRMS (ESI) (*m/z*) calcd. for $C_{38}H_{67}N_{13}O_7$ [M+2H$^+$]: 408.7638, found: 408.7641.

(*S*)-2-[(*S*)-2-(2-{2-[(*S*)-2-Amino-3-(4-hydroxyphenyl)propanamido]acetamido}acetamido)-3-phenylpropanamido]-4-methylpentanamide (1k) (Leu-enkephalin amide)

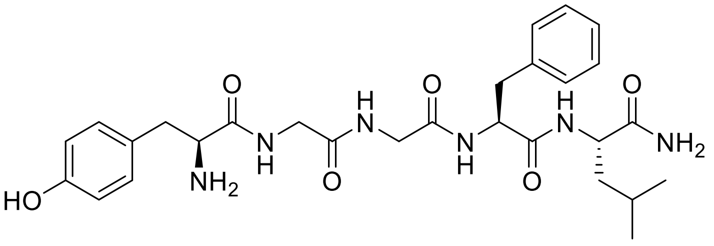

$C_{28}H_{38}N_6O_6$
554.65 g/mol

Compound **1k** was prepared according to **GP2**. For amino acid coupling the following amino acids were used in this order: Fmoc-Leu-OH (813 mg, 2.30 mmol, 5 equiv.), Fmoc-Phe-OH (891 mg, 2.30 mmol, 5 equiv.), Fmoc-Gly-OH (684 mg, 2.30 mmol, 5 equiv.), Fmoc-Gly-OH (684 mg, 2.30 mmol, 5 equiv.), Fmoc-Tyr(*t*Bu)-OH (1.06 g, 2.30 mmol, 5 equiv.). The crude peptide was purified by preparative HPLC (system B, gradient: 5% → 30% acetonitrile in water, 0.1% trifluoroacetic acid) to give **1k** as colorless powder.

***t*R** 7.47 min (system B, 5% → 30% acetonitrile in water, 0.1% trifluoroacetic acid).

^{1}H NMR (400 MHz, CD_3OD) δ (ppm) = 7.34 – 7.24 (m, 4H), 7.26 – 7.18 (m, 1H), 7.14 – 7.09 (m, 2H), 6.82 – 6.76 (m, 2H), 4.64 (dd, *J* = 8.9, 5.8 Hz, 1H), 4.32 (dd, *J* = 9.5, 5.3 Hz, 1H), 4.07 (dd, *J* = 8.3, 6.3 Hz, 1H), 4.03 – 3.70 (m, 4H), 3.17 (dd, *J* = 14.0, 6.0 Hz, 2H), 2.98 (dt, *J* = 14.2, 8.5 Hz, 2H), 1.72 – 1.50 (m, 3H), 0.91 (dd, *J* = 18.2, 6.2 Hz, 6H).

^{13}C NMR (101 MHz, CD_3OD) δ (ppm) = 177.2 (C_q), 173.6 (C_q), 171.6 (C_q), 171.5 (C_q), 170.9 (C_q), 158.3 (C_q), 138.2 (C_q), 131.5 (2 × CH), 130.4 (2 × CH), 129.6 (2 × CH), 127.9 (CH), 126.0 (C_q), 116.9 (2 × CH), 56.24 (CH), 56.15 (CH), 53.1 (CH), 43.7 (CH_2), 43.3 (CH_2), 41.7 (CH_2), 38.6 (CH), 37.7 (CH_2), 25.8 (CH), 23.5 (CH_3), 21.8 (CH_3).

HRMS (ESI) (*m/z*) calcd. for $C_{28}H_{39}N_6O_6$ [M+H$^+$]: 555.2926, found: 555.2927.

(*S*)-*N*-[2-(1*H*-Indol-3-yl)ethyl]-2-acetamido-3-(4-hydroxyphenyl)propanamide (1l)

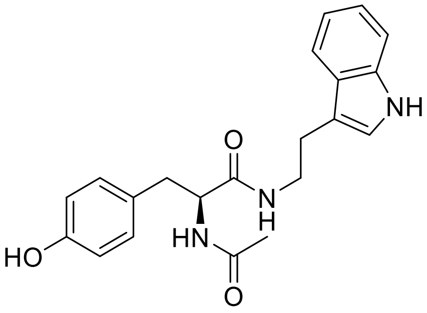

$C_{21}H_{23}N_3O_3$
365.43 g/mol

Compound **1l** was synthesized from *N*-acetyl-*L* tyrosine (2.23 g, 10.0 mmol, 1 equiv.) and Tryptophan (1.60 g, 10.0 mmol, 1 equiv.) with triethylamine (2.79 mL, 2.02 g, 20.0 mmol, 2 equiv.) following **GP1**. 25 mL of dichloromethane and 10 mL of *N*,*N*-dimethylformamide were used as solvents. The crude mixture was purified by preparative HPLC (system A, gradient: 21% → 36 % acetonitrile in water, 0.1 % formic acid) to give **1l** as colorless powder.

t_R 10.19 min (system A, 21% → 36% acetonitrile in water, 0.1% formic acid).

^{1}H NMR (400 MHz, CD_3OD) δ (ppm) = 7.53 (dt, *J* = 7.9, 1.0 Hz, 1H), 7.32 (dt, *J* = 8.1, 0.9 Hz, 1H), 7.08 (ddd, *J* = 8.1, 7.1, 1.1 Hz, 2H), 7.06 – 6.95 (m, 4H), 6.73 – 6.65 (m, 2H), 4.46 (dd, *J* = 8.4, 6.6 Hz, 1H), 3.42 (dddd, *J* = 36.2, 13.1, 7.9, 6.7 Hz, 2H), 2.94 (dd, *J* = 13.8, 6.6 Hz, 1H), 2.89 – 2.78 (m, 2H), 2.74 (dd, *J* = 13.8, 8.4 Hz, 1H), 1.89 (s, 3H).

^{13}C NMR (101 MHz, CD_3OD) δ (ppm) = 173.6 (C_q), 173.1 (C_q), 157.2 (C_q), 138.1 (C_q), 130.4 (CH), 129.1 (C_q), 128.6 (C_q), 123.5 (CH), 122.3 (CH), 119.6 (CH), 119.2 (CH), 116.2 (2 × CH), 113.0 (C_q), 112.2 (CH) , 56.7 (CH), 41.2 (CH_2), 38.3 (CH_2), 26.0 (CH_2) , 22.4 (CH_3).

IR (KBr) $\tilde{\nu}$ (cm^{-1}): 3291 (s, br), 2927 (m), 1647 (s), 1515 (s), 1457 (s), 1371 (m), 1230 (s), 1102 (m), 831 (m), 745 (s).

HRMS (ESI) (*m/z*) calcd. for $C_{21}H_{23}N_3O_3$ [M+H$^+$]: 366.1812, found: 366.1811.

$[\alpha]_D^{20}$ = + 6.0 (c = 1.00 in CH_3OH).

2.2 Synthesis of diazonium salts

General procedure for the preparation of diazonium tetrafluoroborate salts (GP3)

The corresponding aniline (10.0 mmol, 1 equiv.) was dissolved in water (3.5 mL) and aqueous tetrafluoroboric acid (3.5 mL of a 48% (*w/w*) solution, 2.28 g, 26.0 mmol, 2.6 equiv.) and cooled to -7 °C using an ice-salt bath. A solution of sodium nitrite (690 mg, 10.0 mmol, 1 equiv.) in water (2.5 mL) was added dropwise over 1 h using a syringe pump and the mixture was stirred an additional hour below 1 °C. The precipitate was filtered off, washed with cold diethyl ether (-20 °C) and dissolved in a minimal amount of cold acetone (-20 °C). After a second precipitation with cold diethyl ether (-20 °C), the solid was collected by filtration. Diazonium tetrafluoroborate salts were synthesized according to a procedure from literature.[4]

General procedure for the preparation of fully aqueous diazonium chloride solutions (GP4)

The corresponding aniline (5.00 mmol, 1 equiv.) was dissolved in HCl (10 mL of 1.5 M solution), cooled to 0 °C using an ice bath and degassed. A degassed solution of sodium nitrite (345 mg, 5.00 mmol, 1 equiv.) in water (2.5 mL) was added dropwise over 10 minutes using a syringe pump. After stirring for additional 20 minutes at 0 °C, the freshly prepared 0.4 M diazonium chloride solution was directly used for the arylation reactions. Diazonium chloride solutions were synthesized according to a procedure from literature.[1]

General procedure for the preparation of aqueous diazonium chloride solutions containing acetonitrile (GP4’)

The corresponding aniline (5.00 mmol, 1 equiv.) was dissolved in HCl (10 mL of 1.5 M solution) and acetonitrile (2 mL), cooled to 0 °C using an ice bath and degassed. A degassed solution of sodium nitrite (345 mg, 5.00 mmol, 1 equiv.) in water (2.5 mL) was added dropwise over 10 minutes using a syringe pump. After stirring for additional 20 minutes at 0 °C, the freshly prepared 0.345 M diazonium chloride solution was directly used for the arylation reactions. Diazonium chloride solutions were synthesized according to a procedure from literature.[1]

3,4,5-Trifluorophenyldiazonium chloride (2a)

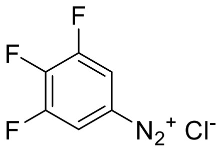

$C_6H_2ClF_3N_2$
194.54 g/mol

Solution **2a** was synthesized according to **GP4**, using 3,4,5-trifluoroaniline (736 mg, 5.00 mmol, 1 equiv.) as starting material. Unlike described in **GP4**, the determination of the concentration (see underneath) resulted in a 0.3 M 3,4,5-trifluorophenyldiazonium chloride solution.

<u>For determining the concentration of 3,4,5-trifluorophenyldiazonium chloride in the solution the reaction was performed in deuterated solvents:</u>

To an ice-cooled degassed solution of the 3,4,5-trifluoroaniline (147 mg, 1.00 mmol, 1 equiv.) in D_2O (1.75 mL) and DCl (0.25 mL of 12 M solution) a degassed solution of sodium nitrite (69 mg, 1.00 mmol, 1 equiv.) in D_2O (0.5 mL) was added dropwise over 10 minutes using a syringe pump. After stirring for additional 20 minutes at 0°C the solution was analyzed by ^1H NMR with maleic acid as internal standard giving 3,4,5-trifluorophenyldiazonium chloride in 75% yield (0.3 M diazonium chloride solution).

3,4,5-Trifluorophenyldiazonium tetrafluoroborate (2a’)

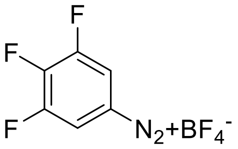

$C_6H_2ClBF_7N_2$
245.89 g/mol

Compound **2a’** was synthesized according to **GP3**, using 3,4,5-trifluoroaniline (1.47 g, 10.0 mmol, 1 equiv.) as starting material. Pure **2a’** was obtained as yellowish crystals.

^{1}H NMR (400 MHz, CD_3CN) δ (ppm) = 8.52 – 8.38 (m, 2H).

The NMR data is in accordance with data from literature.[5]

4-Fluorophenyldiazonium chloride (2b)

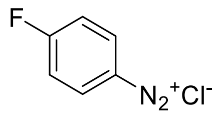

$C_6H_4ClFN_2$
158.56 g/mol

Solution **2b** was synthesized according to **GP4**, using 4-fluoroaniline (475 µl, 556 mg, 5.00 mmol, 1 equiv.) as starting material.

4-Fluorophenyldiazonium tetrafluoroborate (2b')

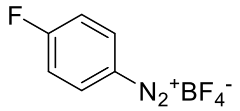

$C_6H_4BF_5N_2$
209.91 g/mol

Compound **2b'** was synthesized according to **GP3**, using 4-fluoroaniline (947 µl, 1.11 g, 10.0 mmol, 1 equiv.) as starting material. Pure **2b'** was obtained as colorless crystals.

^{1}H NMR (400 MHz, CD_3CN) δ (ppm) = 8.62 – 8.50 (m, 2H), 7.71 – 7.55 (m, 2H).

The NMR data is in accordance with data from literature.[6]

3,5-Difluorophenyldiazonium chloride (2c)

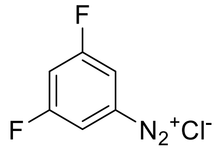

$C_6H_3ClF_2N_2$
176.55 g/mol

Solution **2c** was synthesized according to **GP4'**, using 3,5-difluoroaniline (646 mg, 5.00 mmol, 1 equiv.) as starting material.

2,3,4,5,6-Pentafluorophenyldiazonium tetrafluoroborate (2d')

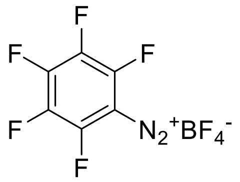

$C_6BF_9N_2$
281.88 g/mol

Nitrosonium tetrafluoroborate (584 mg, 5.00 mmol, 1 equiv.) was dissolved in dry acetonitrile (2 mL) under argon atmosphere and cooled to -30 °C. 2,3,4,5,6-pentafluoroaniline (915 mg, 5.00 mmol, 1 equiv.) was dissolved in dry acetonitrile (2 mL) under argon atmosphere and added to the nitrosonium tetrafluoroborate solution with a syringe pump over 30 min at -30 °C. After stirring for an additional hour, the mixture was treated with dry CH_2Cl_2 (10 mL) and the precipitate was filtered off, washed with dry CH_2Cl_2 and dissolved in a minimal amount of dry acetonitrile. Precipitation with dry CH_2Cl_2 and filtration led to pure **2d'** as yellowish crystals. The synthesis was performed according to a procedure from literature.[7]

^{19}F NMR (376 MHz, CD_3CN) δ (ppm) = -118.7 (m), -122.4 (m), -150.8 (m), -151.6 (m).

The NMR data is in accordance with data from literature.[8]

4-(Trifluoromethyl)phenyldiazonium chloride (2e)

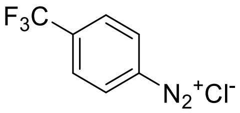

$C_7H_4ClF_3N_2$
208.57 g/mol

Solution **2e** was synthesized according to **GP4'**, using 4-(trifluoromethyl)aniline (628 µl, 805 mg, 5.00 mmol, 1 equiv.) as starting material.

4-Chlorophenyldiazonium chloride (2f)

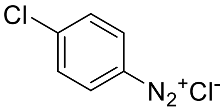

$C_6H_4Cl_2N_2$
175.01 g/mol

Solution **2f** was synthesized according to **GP4**, using 4-chloroaniline (638 mg, 5.00 mmol, 1 equiv.) as starting material.

4-Clorophenyldiazonium tetrafluoroborate (2f')

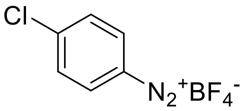

$C_6H_4BClF_4N_2$
226.37 g/mol

Compound **2f'** was synthesized according to **GP3**, using 4-chloroaniline (1.28 g, 10.0 mmol, 1 equiv.) as starting material. Pure **2f'** was obtained as colorless crystals.

^{1}H NMR (400 MHz, CD_3CN) δ (ppm) = 8.49 – 8.43 (m, 2H), 7.97 – 7.91 (m, 2H).

The NMR data is in accordance with data from literature.[9]

4-Bromophenyldiazonium chloride (2g)

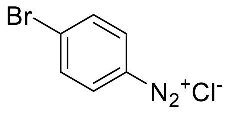

$C_6H_4BrClN_2$
219.47 g/mol

Solution **2g** was synthesized according to **GP4**, using 4-bromoaniline (860 mg, 5.00 mmol, 1 equiv.) as starting material.

4-Bromophenyldiazonium tetrafluoroborate (2g')

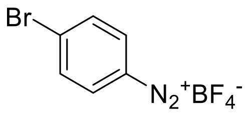

$C_6H_4BBrF_4N_2$
270.82 g/mol

Compound **2g'** was synthesized according to **GP3**, using 4-bromoaniline (1.72 g, 10.0 mmol, 1 equiv.) as starting material. Pure **2g'** was obtained as colorless crystals.

^{1}H NMR (400 MHz, CD_3CN) δ (ppm) = 8.39 – 8.32 (m, 2H), 8.15 – 8.07 (m, 2H).

The NMR data is in accordance with data from literature.[6]

2-Chlorophenyldiazonium chloride (2h)

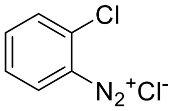

$C_6H_4Cl_2N_2$
175.01 g/mol

Solution **2h** was synthesized according to **GP4**, using 2-chloroaniline (638 mg, 5.00 mmol, 527 µL, 1 equiv.) as starting material.

3-Chlorophenyldiazonium chloride (2i)

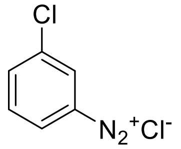

$C_6H_4Cl_2N_2$
175.01 g/mol

Solution **2i** was synthesized according to **GP4**, using 3-chloroaniline (638 mg, 5.00 mmol, 523 µL, 1 equiv.) as starting material.

4-Methoxyphenyldiazonium tetrafluoroborate (2j)

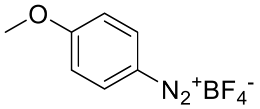

$C_7H_7BF_4N_2O$
221.95g/mol

Compound **2j** was synthesized according to **GP3**, using 4-methoxyaniline (1.23 g, 10.0 mmol, 1 equiv.) as starting material. Pure **2j** was obtained as colorless crystals.

^{1}H NMR (400 MHz, CD_3CN) δ (ppm) = 8.43 – 8.36 (m, 2H), 7.37 – 7.32 (m, 2H), 4.06 (s, 3H).

The NMR data is in accordance with data from literature.[6]

4-Cyanophenyldiazonium tetrafluoroborate (2k)

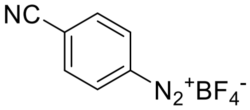

$C_7H_4BF_4N_3$
216.93 g/mol

Compound **2k** was synthesized according to **GP3**, using 4-aminobenzonitrile (1.18 g, 10.0 mmol, 1 equiv.) as starting material. Pure **2k** was obtained as yellowish crystals.

^{1}H NMR (400 MHz, CD_3CN) δ (ppm) = 8.65 – 8.59 (m, 2H), 8.29 – 8.22 (m, 2H).

The NMR data is in accordance with data from literature.[4]

3. Optimization experiments with tyramine hydrochloride

5-(2-Aminoethyl)-3',4',5'-trifluoro-[1,1'-biphenyl]-2-ol (3aa)

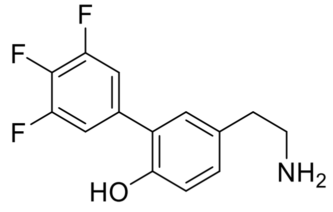

$C_{14}H_{12}F_3NO$
267.25 g/mol

The optimization experiments were carried out under irradiation in the photoreactor described above. Tyramine hydrochloride (**1a**) (174 mg, 1.00 mmol, 1 equiv.) and 2.5 mL (0.75 mmol, 0.75 equiv.) of a 0.3 M solution of 3,4,5-trifluorophenyldiazonium chloride (**2a**) (preparation see above) were added to 3 mL of water under argon atmosphere and the mixture was overlayered with 5.5 mL of methyl *tert*-butyl ether. Reaction times and equivalents (2.5 mL of solution of **2a** correspond to 0.75 equiv.) are given in the table below. If adding additional diazonium salt **2a** after irradiation for 2 h, the ether phase was replaced by the same volume of fresh methyl *tert*-butyl ether. After the full reaction time, the reaction mixture was extracted with ethyl acetate (3 × 20 mL) and the solvent of the combined organic phases (including the methyl *tert*-butyl ether separated earlier) was removed under reduced pressure. Yields were determined by ^{1}H NMR using 1,3,5-trimethoxybenzene as internal standard (see Table S1 below). Purification of a representative sample by preparative HPLC (system A, gradient: 10% → 23% acetonitrile in water, 0.1% formic acid) gave **3aa** as formiate salt as colorless powder.

***R*f** 0.1 (CH_2Cl_2/MeOH = 1:1) [UV].

***t*R** 9.34 min (system A, 10% → 23% acetonitrile in water, 0.1% formic acid).

^{1}H NMR (400 MHz, CD_3OD) δ (ppm) = 7.42 – 7.31 (m, 2H), 7.20 (d, *J* = 2.2 Hz, 1H), 7.13 (dd, *J* = 8.3, 2.2 Hz, 1H), 6.90 (d, *J* = 8.3 Hz, 1H), 3.15 (t, *J* = 7.6 Hz, 2H), 2.90 (t, *J* = 7.6 Hz, 2H).

^{13}C NMR (101 MHz, CD_3OD) δ (ppm) = 154.9 (C_q), 151.9 (ddd, J_{CF} = 246.3, 9.8, 4.2 Hz, 2 × C_q), 139.7 (dt, J_{CF} = 249.0, 15.6 Hz, C_q), 136.6 (td, J_{CF} = 8.4, 4.8 Hz, C_q), 131.6 (CH), 131.0 (CH), 129.3 (C_q), 126.7 (C_q), 117.7 (CH), 114.5 (d, J_{CF} = 21.7 Hz, 2 × CH), 42.1 (CH_2), 33.9 (CH_2).

^{19}F NMR (376 MHz, CD_3OD) δ (ppm) = -137.0 (m, 2F), -165.2 (m, 1F).

HRMS (ESI) (*m/z*) calcd. for $C_{14}H_{13}F_3NO$ [M+H$^+$]: 268.0944, found: 268.0945.

The NMR data is in accordance with data from literature.[11]

Table S1. Optimization of reaction conditions

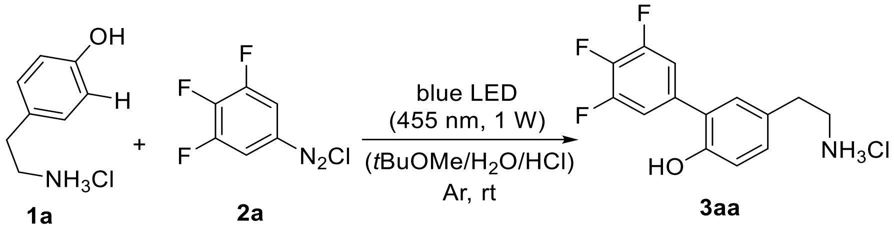

Entry	Equivalents **2a** and reaction time	Yield **3aa** (%)
1	0.75 (1 h)	28
2	0.75 (2 h)	38
3	0.75 (3 h)	31
4	0.75 (4 h)	29
5	0.75 (6 h)	31
6	1.1 (1 h)	31
7	1.5 (1 h)	19
8	1.1 (2 h)	38
9	1.5 (2 h)	39
10	0.75 + 0.75 (2 + 2 h)	47
11	1.1 + 0.75 (2 + 2 h)	50
12	1.1 + 0.75 + 0.75 (2 + 2 + 2 h)	56
13	1.1 + 0.75 + 0.75 (3 + 3 + 3 h)	59

3,4,5-Trifluorophenyldiazonium chloride (**2a**) previously prepared from 3,4,5-trifluoroaniline using sodium nitrite in aqueous hydrochloric acid. Yields determined by ^1H NMR spectroscopy using 1,3,5-trimethoxybenzene as internal standard after aqueous work-up.

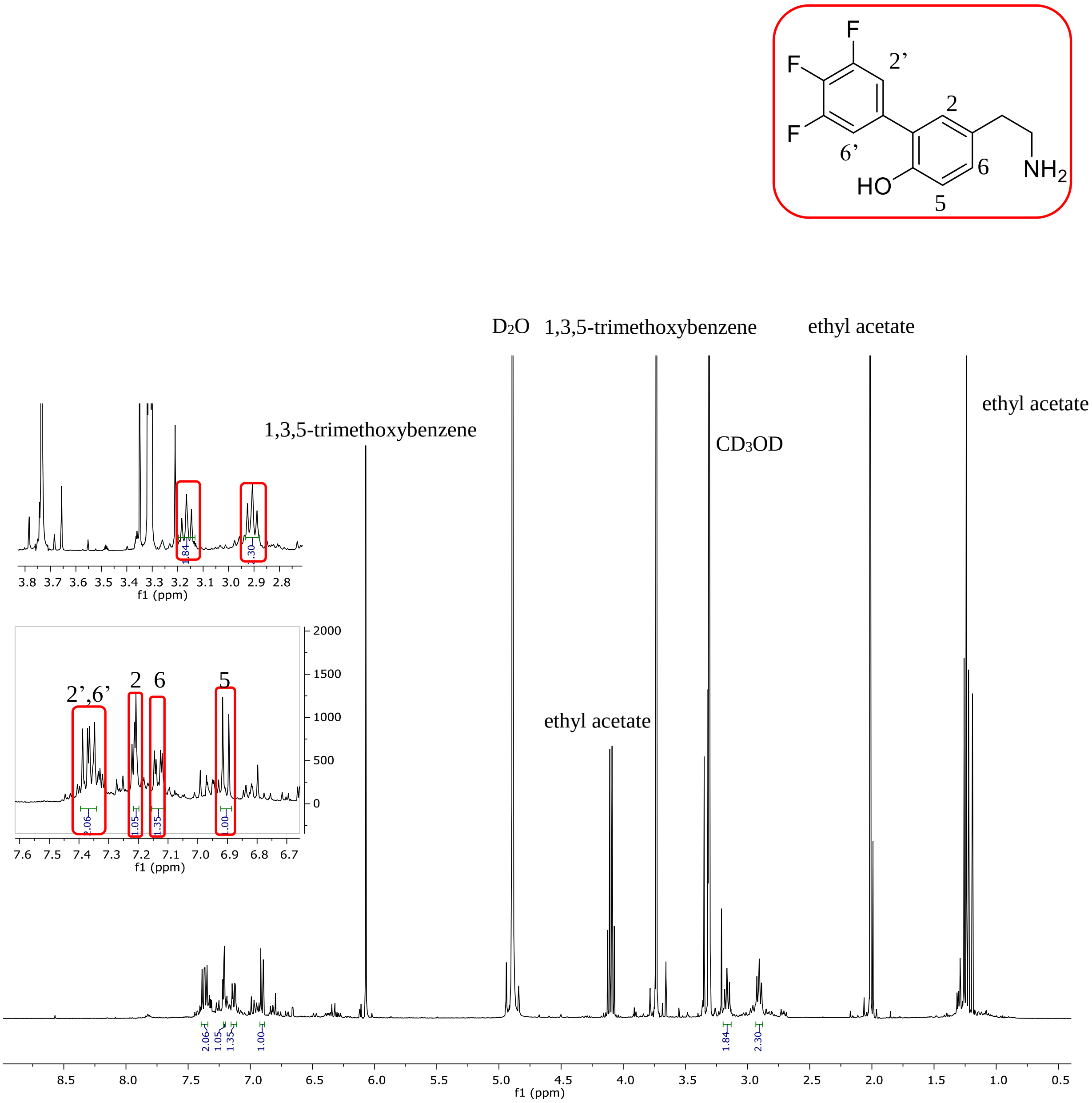

Figure S3. Crude ^{1}H NMR spectra of **3aa** after irradiation for 3 + 3 + 3 h with 1.1 + 0.75 + 0.75 equiv. of 3,4,5-triflourophenyldiazonium chloride. No major tyramine-derived side products occurred.

4. Scope and limitations

4.1 Assignment of regioisomers

The regioisomers arising from arylation of the tyrosine residues in 2- and 3-position of the phenolic subunit were assigned by ^{1}H NMR spectroscopy. A representative example with cutouts of the respective aromatic regions is shown below.

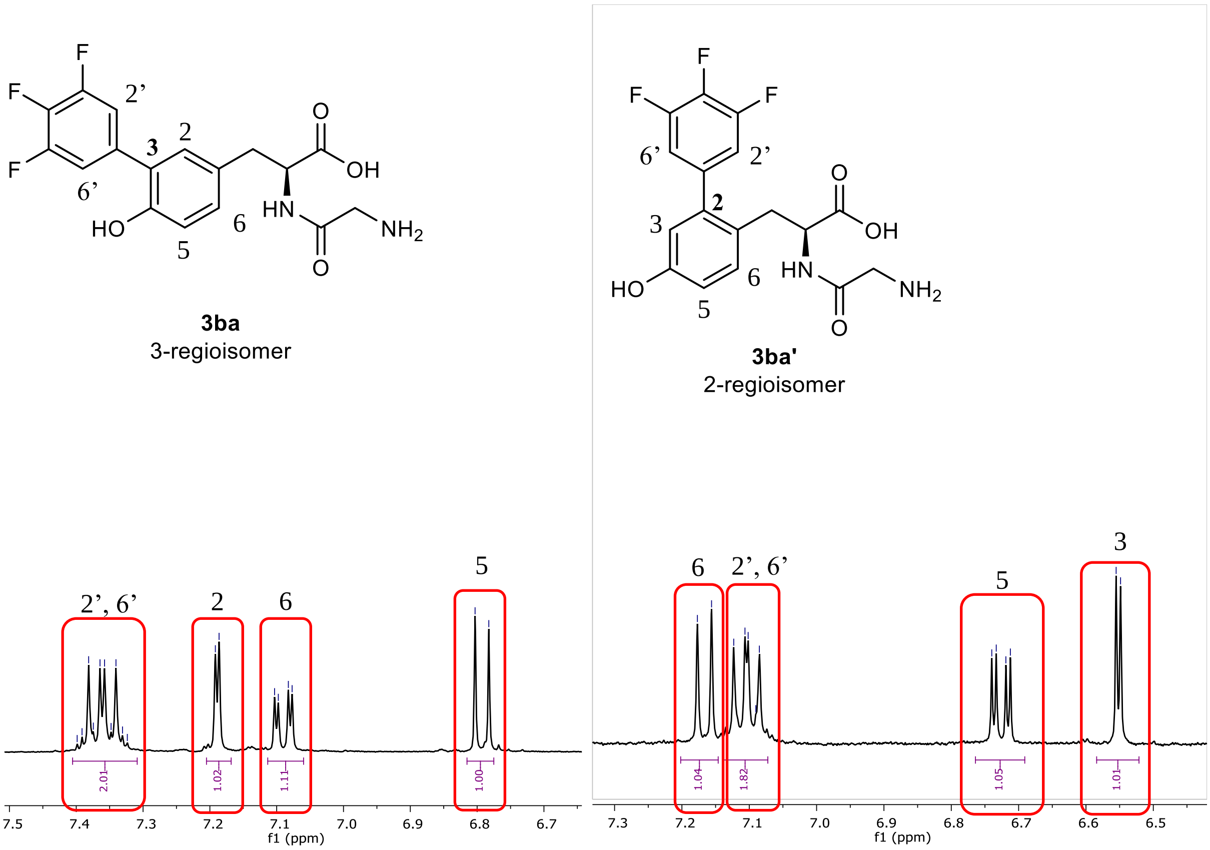

Figure S4. Cutouts of ^{1}H NMR spectra of **3ba** and its regioisomer **3ba'** in the aromatic regions. Due to the chemical shifts of the typical substitution patterns, the two regioisomers can be assigned unambigiously.

4.2 Variation of reaction time and equivalents

(*S*)-2-(2-Aminoacetamido)-3-(3',4',5'-trifluoro-6-hydroxy-[1,1'-biphenyl]-3-yl)propanoic acid (3ba) and its regioisomer (*S*)-2-(2-Aminoacetamido)-3-(3',4',5'-trifluoro-5-hydroxy-[1,1'-biphenyl]-2-yl)propanoic acid (**3ba'**)

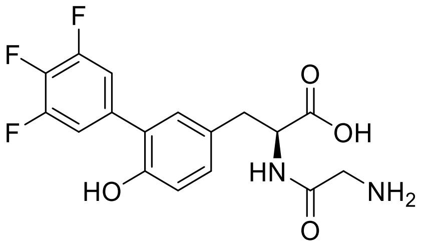

$C_{17}H_{15}F_3N_2O_4$
368.31g/mol

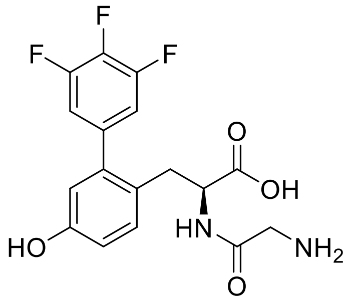

$C_{17}H_{15}F_3N_2O_4$
368.31g/mol

H-Gly-Tyr-OH (**1b)** **(**238 mg, 1.00 mmol, 1 equiv.) and **2a** (3.8 mL of 0.3 M solution, 1.1 mmol, 1.1 equiv.) were added to 1 mL of water and 2 mL of 1 M HCl under argon atmosphere and the mixture was overlayered with 5,5 mL of methyl *tert*-butyl ether. After irradiation for 2 h, the ether phase was replaced and additional **2a** (2.5 mL of 0.3 M solution, 0.75 mmol, 0.75 equiv.) was added. After irradiation for 2 h, replacement of the organic phase and addition of the diazonium solution was repeated once more. After a total irradiation time of 6 h and adding 2 times diazonium solution **2a**, the reaction mixture was extracted with ethyl acetate (3 × 20 mL) and the combined organic phases (including the methyl *tert*-butyl ether separated earlier) were concentrated to 1/10 of the original volume and 10 mL of methanol and maleic acid as standard were added. Analysis by ^{1}H NMR gave **3ba** in 60% and **3ba'** (minor isomer) in 9%. After removing the solvent under reduced pressure, purification by preparative HPLC (system A, gradient: 37% → 46% acetonitrile in water, 0.1% formic acid) gave **3ba** and **3ba'** as colorless powders. The preparation of **3ba** was also carried out under different reaction conditions, regarding reaction times and equivalents of **2a**, but otherwise in analogy to the procedure described above. For individual results, see Scheme 2 in the manuscript.

3ba

***R*f** 0.2 (CH_2Cl_2/MeOH = 1:1) [UV].

***t*R** 7.78 min (system A, 37% → 46% acetonitrile in water, 0.1% formic acid).

^{1}H NMR (400 MHz, CD_3OD) δ (ppm) = 7.36 (dd, *J* = 9.6, 6.8 Hz, 2H), 7.19 (d, *J* = 2.2 Hz, 1H), 7.09 (dd, *J* = 8.3, 2.2 Hz, 1H), 6.79 (d, *J* = 8.2 Hz, 1H), 4.53 (dd, *J* = 8.6,

4.5 Hz, 1H), 3.65 (d, J = 15.8 Hz, 1H), 3.49 (d, J = 15.8 Hz, 1H), 3.19 (dd, J = 14.0, 4.5 Hz, 1H), 2.87 (dd, J = 14.0, 8.6 Hz, 1H).

^{13}C NMR (101 MHz, CD_3OD) δ (ppm) = 177.8 (C_q), 166.7 (C_q), 154.2 (C_q), 151.9 (ddd, J_{CF} = 246.1, 9.7, 4.2 Hz, 2 × C_q), 139.5 (dt, J_{CF} = 248.7, 15.7 Hz, C_q), 136.9 (td, J_{CF} = 8.5, 4.9 Hz, C_q), 132.1 (CH), 131.6 (CH), 130.9 (C_q), 126.0 (C_q), 117.1 (CH), 115.1 – 114.0 (m, 2 × CH), 57.8 (CH), 41.6 (CH_2), 38.5 (CH_2). One quaternary carbon signal missing.

^{19}F NMR (376 MHz, CD_3OD) δ (ppm) = -137.2 (m, 2F), -165.7 (m, 1F).

IR (KBr) $\tilde{\nu}$ (cm^{-1}): 3276 (w, br), 3078 (w), 2926 (w), 1684 (m), 1616 (s), 1531 (s), 1509 (s), 1409 (m), 1253 (m), 1129 (w), 1041 (m), 862 (w), 650 (w).

HRMS (ESI) (*m/z*) calcd. for $C_{17}H_{16}F_3N_2O_4$ [$M+H^+$]: 369.1057, found: 369.1059.

$[\alpha]_D^{20}$ = + 38.9 (c = 1.00 in CH_3OH).

3ba'

***R*f** 0.2 (CH_2Cl_2/MeOH = 1:1) [UV].

***t*R** 5.08 min (system A, 37% → 46% acetonitrile in water, 0.1% formic acid).

^{1}H NMR (400 MHz, CD_3OD) δ (ppm) = 7.17 (d, J = 8.4 Hz, 1H), 7.10 (dd, J = 8.6, 6.7 Hz, 2H), 6.73 (dd, J = 8.4, 2.7 Hz, 1H), 6.55 (d, J = 2.6 Hz, 1H), 4.36 (dd, J = 9.7, 5.1 Hz, 1H), 3.59 (d, J = 15.7 Hz, 1H), 3.41 (d, J = 15.7 Hz, 1H), 3.17 (dd, J = 14.5, 5.2 Hz, 1H), 2.70 (dd, J = 14.5, 9.7 Hz, 1H).

^{13}C NMR (101 MHz, CD_3OD) δ (ppm) = 177.4 (C_q), 166.3 (C_q), 157.0 (C_q), 152.00 (ddd, J_{CF} = 248.5, 9.9, 4.2 Hz, 2 × C_q), 142.5 – 138.8 (m, C_q), 139.8 – 139.3 (m, C_q) 132.6 (CH), 127.3 (C_q), 117.5 (CH), 116.1 (CH), 115.3 – 114.5 (m, 2 × CH), 56.5 (CH), 41.5 (CH_2), 36.1 (CH_2). One carbon signal missing.

^{19}F NMR (376 MHz, CD_3OD) δ (ppm) = -135.9 (m, 2F), -165.0 (m, 1F).

HRMS (ESI) (*m/z*) calcd. for $C_{17}H_{16}F_3N_2O_4$ [$M+H^+$]: 369.1057, found: 369.1060.

4.3 Variation of the substitution pattern of the phenyldiazonium salt

(*S*)-2-(2-Aminoacetamido)-3-(3',5'-difluoro-6-hydroxy-[1,1'-biphenyl]-3-yl)propanoic acid (3bc)

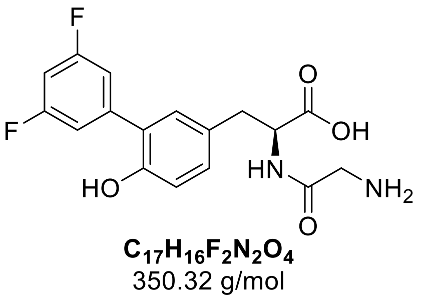

$C_{17}H_{16}F_2N_2O_4$
350.32 g/mol

H-Gly-Tyr-OH (**1b**) **(**238 mg, 1.00 mmol, 1 equiv.) and **2c** (4.4 mL of 0.4 M solution, 1.50 mmol, 1.5 equiv.) were added to 1 mL of water and 2 mL of 1 M HCl under argon atmosphere and the mixture was overlayered with 5.5 mL of methyl *tert*-butyl ether. After irradiation for 2 h, the ether phase was taken off and the reaction mixture was extracted with ethyl acetate (3 × 20 mL). The combined organic phases (including the methyl *tert*-butyl ether separated earlier) were concentrated to 1/10 of the original volume and 10 mL of methanol and maleic acid as internal standard were added. All organic phases were analyzed by ^{1}H NMR, yielding **3bc** in 11%. After removing the solvent under reduced pressure, purification by preparative HPLC (system A, gradient: 8% → 23% acetonitrile in water, 0.1% formic acid) gave **3bc** as colorless powder.

***R*f** 0.2 (CH_2Cl_2/MeOH = 1:1) [UV].

***t*R** 10.52 min (system A, 8% → 23% acetonitrile in water, 0.1% formic acid).

^{1}H NMR (400 MHz, CD_3OD) δ (ppm) = 7.24 – 7.17 (m, 3H), 7.10 (dd, *J* = 8.3, 2.3 Hz, 1H), 6.87 – 6.78 (m, 2H), 4.54 (dd, *J* = 8.7, 4.5 Hz, 1H), 3.66 (d, *J* = 15.8 Hz, 1H), 3.50 (d, *J* = 15.9 Hz, 1H), 3.20 (dd, J = 14.0, 4.5 Hz, 1H), 2.87 (dd, *J* = 14.0, 8.8 Hz, 1H).

^{13}C NMR (101 MHz, CD_3OD) δ (ppm) = 177.8 (C_q), 166.6 (C_q), 164.1 (dd, J_{CF} = 245.2, 13.3, 2 × C_q), 154.2 (C_q), 143.9 (t, J_{CF} = 10.1 Hz, C_q), 132.2 (CH), 131.5 (CH), 130.9 (C_q), 126.8 (C_q), 117.1 (CH), 113.6 – 112.5 (m, 2 × CH), 102.4 (t, J_{CF} = 25.8 Hz, CH), 57.8 (CH), 41.6 (CH_2), 38.5 (CH_2).

^{19}F NMR (376 MHz, CD_3OD) δ (ppm) = -111.6 (m, 2F).

IR (KBr) $\tilde{\nu}$ (cm^{-1}): 3234 (m, br), 3085 (m), 2934 (m), 1683 (s), 1624 (s), 1596 (s), 1513 (m), 1410 (m), 1344 (m), 1182 (s), 1120 (m), 1025 (m), 986 (s), 863 (m), 721 (w).

HRMS (ESI) (*m/z*) calcd. for $C_{17}H_{17}F_2N_2O_4$ [M+H$^+$]: 351.1151, found: 351.1148.

$[\alpha]_D^{20}$ = + 10.5 (c = 1.00 in CH_3OH).

Further experiments using diazonium salts **2b**, **2d-2g** were carried out under similar conditions, but yielded only traces of arylation products.

General procedure for irradiation experiments with *N*-acetyl-*L*-tyrosine and various substituted diazonium salts (GP5)

N-Acetyl-*L*-tyrosine (**1d**) (223 mg, 1.00 mmol, 1.0 equiv.) and a 0.4 M solution of the respective diazonium salt (2.5 mL, 1.00 mmol, 1.0 equiv.) were added to water (3 mL) under argon atmosphere and the mixture was overlayered with di-*iso*propyl ether (5.5 mL). After irradiation for 3 h, the ether phase was replaced and additional diazonium chloride solution (1.00 mmol, 1.0 equiv.) was added. After additional irradiation for 3 h, the reaction mixture was extracted with ethyl acetate (3 × 20 mL). The solvent of an aliquot of the combined organic phases (including the di-*iso*propyl ether separated earlier) was removed under reduced pressure and analyzed by ^{1}H NMR. Representative samples of the product were purified by column chromatography.

(*S*)-2-Acetamido-3-(4'-fluoro-6-hydroxy-[1,1'-biphenyl]-3-yl)propanoic acid (3db)

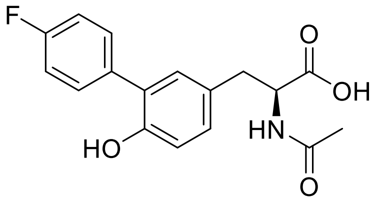

$C_{17}H_{16}FNO_4$
317.32 g/mol

Compound **3db** was prepared according **GP5**, using **2b** (0.4 M solution, 2 × 1.00 mmol, 2 × 1.0 equiv.). Analysis of the crude reaction mixture by ^{1}H NMR led to a yield of **3db** of 33%. Purification of a representative sample by column chromatography (CH_2Cl_2 / MeOH = 100:1 + 0.1% formic acid → 20:1 + 0,1% formic acid) yielded pure **3db** as a colorless oil (74.4 mg, 225 µmol, 22%).

***R*f** 0.2 (CH_2Cl_2/MeOH = 10:1 + 0.1 % FA) [UV].

^{1}H NMR (400 MHz, CD_3OD) δ (ppm) = 8.08 (s, 1H), 7.58 – 7.51 (m, 2H), 7.13 – 7.05 (m, 3H), 7.02 (dd, *J* = 8.2, 2.3 Hz, 1H), 6.81 (d, *J* = 8.2 Hz, 1H), 4.64 (dd, *J* =

8.7, 5.2 Hz, 1H), 3.14 (dd, J = 14.0, 5.2 Hz, 1H), 2.90 (dd, J = 14.0, 8.7 Hz, 1H), 1.92 (s, 3H). (signal at 8.08 ppm resulting from formiate)

^{13}C NMR (101 MHz, CD_3OD) δ (ppm) = 174.9 (C_q), 173.1 (C_q), 163.2 (d, J_{CF} = 243.9 Hz, C_q), 154.3 (C_q), 136.3 (d, J_{CF} = 3.3 Hz, C_q), 132.4 (CH), 132.1 (d, J_{CF} = 7.9 Hz, 2 × CH), 130.3 (CH), 129.5 (C_q), 128.6 (C_q), 116.9 (CH), 115.5 (d, J_{CF} = 21.5 Hz, 2 × CH), 55.4 (CH), 37.7 (CH_2), 22.3 (CH_3).

^{19}F NMR **(**376 MHz, CD_3OD) δ (ppm) = -117.0 (m, 1F).

IR (KBr) $\tilde{\nu}$ (cm^{-1}): 3297 (m, br), 3082 (m), 2926 (m), 1718 (s), 1653 (m), 1607 (m), 1503 (m), 1435 (m), 1221 (s), 1133 (m), 839 (m), 812 (m).

HRMS (ESI) (*m/z*) calcd. for $C_{17}H_{16}FNNaO_4$ [M+Na$^+$]: 340.0956, found: 340.0953.

$[\alpha]_D^{20}$ = + 37.1 (c = 1.00 in CH_3OH).

(*S*)-2-Acetamido-3-[6-hydroxy-4'-(trifluoromethyl)-[1,1'-biphenyl]-3-yl]propanoic acid (3de)

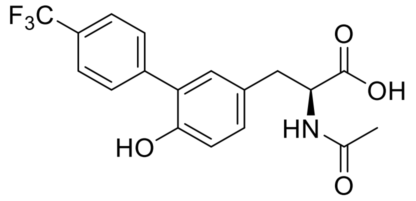

$C_{18}H_{16}F_3NO_4$
367.32 g/mol

Compound **3de** was prepared according **GP5**, using **2e** (0.345 M solution, 2 × 1.00 mmol, 2 × 1.0 equiv.). Analysis of the crude reaction mixture by ^{1}H NMR led to a yield of **3de** of 65%. Purification of a representative sample by column chromatography (CH_2Cl_2 / MeOH = 100:1 + 0.1% formic acid → 30:1 + 0,1% formic acid) yielded pure **3de** as brown oil (133 mg, 361 µmol, 36%).

***R*f** 0.2 (CH_2Cl_2/MeOH = 10:1 + 0.1% FA) [UV].

^{1}H NMR (400 MHz, CD_3OD) δ (ppm) = 8.08 (s, 1H), 7.78 – 7.72 (m, 2H), 7.69 – 7.63 (m, 2H), 7.17 (d, J = 2.2 Hz, 1H), 7.08 (dd, J = 8.3, 2.3 Hz, 1H), 6.85 (d, J = 8.2 Hz, 1H), 4.65 (dd, J = 8.8, 5.2 Hz, 1H), 3.16 (dd, J = 14.0, 5.2 Hz, 1H), 2.91 (dd, J = 14.0, 8.8 Hz, 1H), 1.92 (s, 3H). (signal at 8.08 ppm resulting from formiate)

^{13}C NMR (101 MHz, CD_3OD) δ (ppm) = 174.8 (C_q), 173.1 (C_q), 154.5 (C_q), 144.3 (C_q), 132.4 (CH), 131.2 (CH), 130.9 (2 × CH), 129.7 (C_q), 129.5 (q, J_{CF} = 32.1 Hz, C_q), 128.1 (C_q), 126.0 (q, J_{CF} = 270.9 Hz, C_q), 125.7 (q, J_{CF} = 3.7 Hz, 2 × CH), 117.1 (CH), 55.3 (CH), 37.7 (CH_2), 22.3 (CH_3).

^{19}F NMR (376 MHz, CD_3OD) δ (ppm) = -62.2 (m, 3F).

IR (KBr) $\tilde{\nu}$ (cm^{-1}): 3296 (m, br), 3089 (m), 2935 (m), 1733 (m), 1652 (m), 1616 (m), 1538 (m), 1446 (m), 1404 (m), 1326 (s), 1165 (m), 1123 (m), 1067 (m), 1016 (m), 899 (w), 847 (m).

HRMS (ESI) (*m/z*) calcd. for $C_{18}H_{17}F_3NO_4$ [M+H$^+$]: 368.1104, found: 368.1103.

$[\alpha]_D^{20}$ = + 2.9 (c = 1.00 in CH_3OH).

(*S*)-2-Acetamido-3-(4'-chloro-6-hydroxy-[1,1'-biphenyl]-3-yl)propanoic acid (3df)

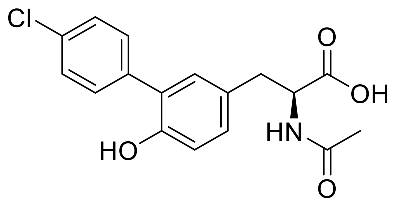

$C_{17}H_{16}ClNO_4$
333.77 g/mol

Compound **3df** was prepared according **GP5**, using **2f** (0.4 M solution, 2 × 1.00 mmol, 2 × 1.0 equiv.). Analysis of the crude reaction mixture by ^{1}H NMR led to a yield of **3df** of 43%. Purification of a representative sample by column chromatography (CH_2Cl_2 / MeOH = 100:1 + 0.1% formic acid → 20:1 + 0,1% formic acid) yielded pure **3df** as red brown oil (93,0 mg, 279 µmol, 28%).

***R*f** 0.3 (CH_2Cl_2/MeOH = 10:1 + 0.1% FA) [UV].

^{1}H NMR (400 MHz, CD_3OD) δ (ppm) = 8.09 (s, 1H), 7.57 – 7.51 (m, 2H), 7.40 – 7.34 (m, 2H), 7.12 (d, *J* = 2.3 Hz, 1H), 7.03 (dd, *J* = 8.2, 2.3 Hz, 1H), 6.81 (d, *J* = 8.2 Hz, 1H), 4.63 (dd, *J* = 8.8, 5.2 Hz, 1H), 3.14 (dd, *J* = 14.0, 5.2 Hz, 1H), 2.90 (dd, *J* = 14.0, 8.8 Hz, 1H), 1.91 (s, 3H). (signal at 8.09 ppm resulting from formiate)

^{13}C NMR (101 MHz, CD_3OD) δ (ppm) = 175.0 (C_q), 173.1 (C_q), 154.3 (C_q), 138.9 (C_q), 133.5 (C_q), 132.3 (CH), 131.9 (2 × CH), 130.6 (CH), 129.6 (C_q), 129.0 (2 × CH), 128.3 (C_q), 117.0 (CH), 55.4 (CH), 37.7 (CH_2), 22.3 (CH_3).

IR (KBr) $\tilde{\nu}$ (cm^{-1}): 3297 (m, br), 3084 (m), 2926 (m), 1722 (s), 1652 (s), 1610 (s), 1488 (s), 1430 (m), 1375 (m), 1265 (m), 1223 (m), 1133 (m), 1090 (m), 1012 (m), 832 (m).

HRMS (ESI) (*m/z*) calcd. for $C_{17}H_{16}ClNO_4$ [M+Na$^+$]: 356.0660, found: 356.0663.

$[\alpha]_D^{20}$ = + 26.2 (c = 1.00 in CH_3OH).

(*S*)-2-Acetamido-3-(4'-bromo-6-hydroxy-[1,1'-biphenyl]-3-yl)propanoic acid (3dg)

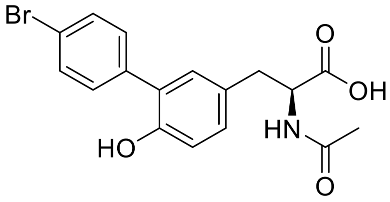

$C_{17}H_{16}BrNO_4$
378.22 g/mol

Compound **3dg** was prepared according **GP5**, using **2g** (0.4 M solution, 2 × 1.00 mmol, 2 × 1.0 equiv.). Analysis of the crude reaction mixture by ^{1}H NMR led to a yield of **3dg** of 41%. Purification of a representative sample by column chromatography (CH_2Cl_2 / MeOH = 100:1 + 0.1% formic acid → 20:1 + 0,1% formic acid) yielded pure **3dg** as red brown oil (113 mg, 230 µmol, 30%).

***R*f** 0.2 (CH_2Cl_2/MeOH = 10:1 + 0.1% FA) [UV].

^{1}H NMR (400 MHz, CD_3OD) δ (ppm) = 8.08 (s, 1H), 7.54 – 7.45 (m, 4H), 7.12 (d, *J* = 2.3 Hz, 1H), 7.04 (dd, *J* = 8.3, 2.3 Hz, 1H), 6.81 (d, *J* = 8.3 Hz, 1H), 4.63 (dd, *J* = 8.8, 5.2 Hz, 1H), 3.14 (dd, *J* = 14.0, 5.2 Hz, 1H), 2.90 (dd, *J* = 14.0, 8.8 Hz, 1H), 1.91 (s, 3H). (signal at 8.08 ppm resulting from formiate)

^{13}C NMR (101 MHz, CD_3OD) δ (ppm) = 174.9 (C_q), 173.1 (C_q), 164.6 (CH), 154.3 (C_q), 139.3 (C_q), 132.2 (2 × CH), 132.0 (2 × CH), 130.6 (CH), 129.6 (C_q), 128.3 (C_q), 121.5 (C_q), 117.0 (CH), 55.3 (CH), 37.7 (CH_2), 22.3 (CH_3).

IR (KBr) $\tilde{\nu}$ (cm^{-1}): 3307 (m, br), 3085 (m), 2929 (m), 1723 (s), 1653 (s), 1611 (m), 1485 (s), 1429 (s), 1374 (m), 1285 (m), 1218 (s), 1133 (m), 1072 (m), 1009 (s), 830 (s), 546 (m).

HRMS (ESI) (*m/z*) calcd. for $C_{17}H_{16}BrNO_4$ [M+Na$^+$]: 400.0155, found: 400.0150.

$[\alpha]_D^{20}$ = + 26.2 (c = 1.00 in CH_3OH).

(*S*)-2-Acetamido-3-(2'-chloro-6-hydroxy-[1,1'-biphenyl]-3-yl)propanoic acid (3dh)

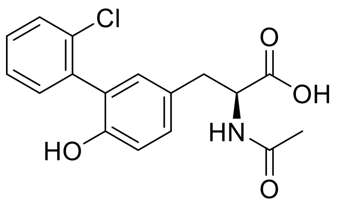

$C_{17}H_{16}ClNO_4$
333.77 g/mol

Compound **3dh** was prepared according **GP5**, using **2h** (0.4 M solution, 2 × 1.00 mmol, 2 × 1.0 equiv.). Analysis of the crude reaction mixture by ^{1}H NMR led to a yield of **3dh** of 45%. Purification of a representative sample by column chromatography (CH_2Cl_2 / MeOH = 100:1 + 0.1% formic acid → 30:1 + 0,1% formic acid) yielded pure **3dh** as yellow brown oil (105 mg, 315 µmol, 32%).

***R*f** 0.2 (CH_2Cl_2/MeOH = 10:1 + 0.1% FA) [UV].

^{1}H NMR (400 MHz, CD_3OD) δ (ppm) = 8.09 (s, 1H), 7.46 – 7.42 (m, 1H), 7.33 – 7.27 (m, 3H), 7.09 (dd, *J* = 8.3, 2.3 Hz, 1H), 6.96 (d, *J* = 2.2 Hz, 1H), 6.81 (d, *J* = 8.3 Hz, 1H), 4.62 (dd, *J* = 9.0, 5.0 Hz, 1H), 3.15 (dd, *J* = 14.0, 5.0 Hz, 1H), 2.89 (dd, *J* = 14.0, 9.0 Hz, 1H), 1.92 (s, 3H). (signal at 8.09 ppm resulting from formiate)

^{13}C NMR (101 MHz, CD_3OD) δ (ppm) = 174.9 (C_q), 173.1 (C_q), 154.5 (C_q), 139.3 (C_q), 135.0 (C_q), 133.1 (CH), 132.9 (CH), 130.8 (CH), 130.3 (CH), 129.6 (CH), 129.0 (C_q), 128.0 (C_q), 127.5 (CH), 116.7 (CH), 55.4 (CH), 37.6 (CH_2), 22.4 (CH_3).

IR (KBr) $\tilde{\nu}$ (cm^{-1}): 3327 (m, br), 3061 (m), 2930 (m), 1723 (s), 1652 (s), 1613 (s), 1538 (m), 1510 (s), 1474 (m), 1422 (s), 1375 (m), 1213 (s), 1134 (m), 1070 (m), 1034 (m), 823 (m), 758 (s).

HRMS (ESI) (*m/z*) calcd. for $C_{17}H_{17}ClNO_4$ [M+H$^+$]: 334.0841, found 334.0840.

$[\alpha]_D^{20}$ = + 17.3 (c = 1.00 in CH_3OH).

(*S*)-2-Acetamido-3-(3'-chloro-6-hydroxy-[1,1'-biphenyl]-3-yl)propanoic acid (3di)

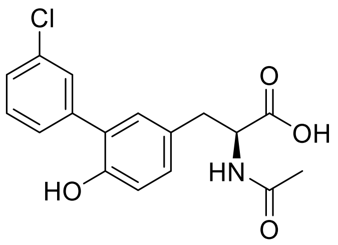

$C_{17}H_{16}ClNO_4$
333.77 g/mol

Compound **3di** was prepared according **GP5**, using **2i** (0.4 M solution, 2 × 1.00 mmol, 2 × 1.0 equiv.). Analysis of the crude reaction mixture by ^{1}H NMR led to a yield of **3di** of 39%. Purification of a representative sample by column chromatography (CH_2Cl_2 / MeOH = 100:1 + 0.1% formic acid → 20:1 + 0,1% formic acid) yielded pure **3di** as yellow brown solid (95.0 mg, 285 µmol, 28%).

***R*f** 0.3 (CH_2Cl_2/MeOH = 10:1 + 0.1% FA) [UV].

^{1}H NMR (400 MHz, CD_3OD) δ (ppm) = 8.08 (s, 1H), 7.58 (t, *J* = 1.8 Hz, 1H), 7.47 (dt, *J* = 7.8, 1.5 Hz, 1H), 7.35 (t, *J* = 7.8 Hz, 1H), 7.27 (ddd, *J* = 8.0, 2.1, 1.1 Hz, 1H), 7.12 (d, *J* = 2.2 Hz, 1H), 7.05 (dd, *J* = 8.2, 2.3 Hz, 1H), 6.83 (d, *J* = 8.2 Hz, 1H), 4.64 (dd, *J* = 8.7, 5.2 Hz, 1H), 3.15 (dd, *J* = 14.0, 5.2 Hz, 1H), 2.91 (dd, *J* = 14.0, 8.7 Hz, 1H), 1.92 (s, 3H). (signal at 8.08 ppm resulting from formiate)

^{13}C NMR (101 MHz, CD_3OD) δ (ppm) = 174.9, 173.1, 154.4, 142.2, 134.7, 132.3, 130.8, 130.4, 130.3, 129.7, 128.7, 128.1, 127.5, 117.1, 55.4, 37.7, 22.4.

IR (KBr) $\tilde{\nu}$ (cm^{-1}): 3410 (m, br), 3068 (w), 2929 (m), 2855 (m), 1706 (m), 1599 (m), 1505 (s), 1472 (s), 1399 (m), 1324 (s), 1151 (s), 1114 (s), 962 (w), 821 (m), 706 (s), 506 (m).

HRMS (ESI) (*m/z*) calcd. for $C_{17}H_{17}ClNO_4$ [M+H$^+$]: 334.0841; found: 334.0846.

$[\alpha]_D^{20}$ = - 6.9 (c = 0.50 in CH_3OH).

4.4 Variation of peptide

General procedure for irradiation experiments with various peptides (GP6)

The respective peptide **(**1 equiv.) and a 0.3 M solution of 3,4,5-trifluorophenyldiazonium chloride (**2a) (**1.1 equiv.) were added to water (2 mL per 1 mmol substrate) and 1 M HCl (1.0 mL per 1.0 mmol substrate) under argon atmosphere and the mixture was overlayered with methyl *tert*-butyl ether (5.5 mL per 1.0 mmol substrate). After irradiation for 2 h and 4 h, the ether phase was replaced and additional 3,4,5-trifluorophenyldiazonium chloride (**2a) (**0.3 M solution, 0.75 equiv.) was added. After a total irradiation time of 6 h adding 2 times diazonium solution **2a**, the reaction mixture was extracted with ethyl acetate (3 × 20 mL). The combined organic phases (including the methyl *tert*-butyl ether separated earlier) were concentrated to 1/10 of the original volume and 10 mL of methanol and maleic acid as internal standard were added. Yields were determined by ^{1}H NMR.

(*S*)-2-Amino-3-(3',4',5'-trifluoro-6-hydroxy-[1,1'-biphenyl]-3-yl)propanoic acid (3ca)

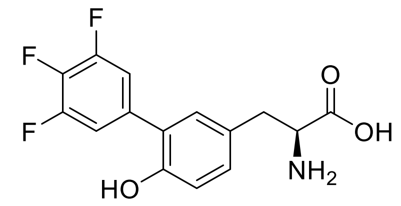

$C_{15}H_{12}F_3NO_3$
311.26 g/mol

Compound **3ca** was prepared according to **GP6**, using *L*-tyrosine (**1c**) **(**181 mg, 1.00 mmol, 1 equiv.) and **2a** (0.3 M solution, 1.1 equiv. and 2 × 0.75 equiv.). Analysis by standard ^{1}H NMR gave **3ca** in 56%. Purification by preparative HPLC (system A, gradient: 22% → 37% acetonitrile in water, 0.1% formic acid) gave **3ca** as colorless powder.

***R*f** 0.2 (CH_2Cl_2/MeOH = 1:1) [UV].

***t*R** 6.34 min (system A, 22% → 37% acetonitrile in water, 0.1% formic acid).

^{1}H NMR (400 MHz, CD_3OD) δ (ppm) = 7.44 – 7.35 (m, 2H), 7.23 (d, *J* = 2.3 Hz, 1H), 7.16 (dd, *J* = 8.3, 2.3 Hz, 1H), 6.89 (d, *J* = 8.2 Hz, 1H), 3.76 (dd, *J* = 8.1, 4.5 Hz, 1H), 3.22 (dd, *J* = 14.6, 4.5 Hz, 1H), 3.01 (dd, *J* = 14.6, 8.2 Hz, 1H).

^{13}C NMR (101 MHz, CD_3OD) δ (ppm) = 173.7 (C_q), 155.0 (C_q), 151.9 (ddd, J_{CF} = 246.1, 9.7, 4.1 Hz, 2 × C_q), 139.6 (dt, J_{CF} = 248.8, 15.4 Hz, C_q), 137.0 – 136.3 (m, CH), 132.3 (CH), 131.7 (CH), 128.3 (C_q), 126.6 (C_q), 117.7 (CH), 114.9 – 114.1 (m, 2 × CH), 57.5 (CH), 37.3 (CH_2).

^{19}F NMR (376 MHz, CD_3OD) δ (ppm) = -137.1 (m, 2F), -165.4 (m, 1F).

IR (KBr) $\tilde{\nu}$ (cm^{-1}): 3245 (m), 1616 (s), 1589 (s), 1532 (s), 1510 (s), 1408 (s), 1330 (s), 1280 (m), 1133 (m), 1041 (s), 863 (m), 837 (m), 771 (m), 734 (m), 653 (m), 548 (m).

HRMS (ESI) (*m/z*) calcd. for $C_{15}H_{13}F_3NO_3$ [M+H$^+$]: 312.0842, found: 312.0842.

$[\alpha]_D^{20}$ = - 14.6 (c = 1.00 in CH_3OH).

(*S*)-2-Acetamido-3-(3',4',5'-trifluoro-6-hydroxy-[1,1'-biphenyl]-3-yl)propanoic acid (3da) and its regioisomer (*S*)-2-Acetamido-3-(3',4',5'-trifluoro-5-hydroxy-[1,1'-biphenyl]-2-yl)propanoic acid (**3da'**)

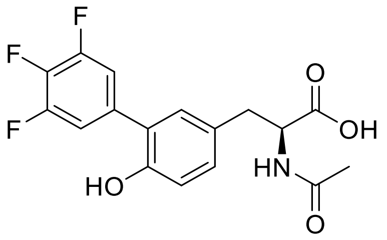

$C_{17}H_{14}F_3NO_4$
353.30 g/mol

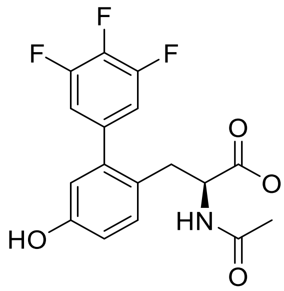

$C_{17}H_{14}F_3NO_4$
353.30 g/mol

Compound **3da** and **3da'** were prepared according to **GP6**, using *N*-acetyl-*L*-tyrosine (**1d**) (223 mg, 1.00 mmol, 1 equiv.) and **2a** (0.3 M solution, 1.1 equiv. and 2 × 0.75 equiv.). Analysis by standard ^{1}H NMR gave **3da** in 69% and **3da'** (minor isomer) in 13%. Purification by preparative HPLC (system A, gradient: 24% → 39% acetonitrile in water, 0.1% formic acid) gave **3da** and **3da'**as colorless powders.

3da

***R*f** 0.2 (CH_2Cl_2/MeOH = 1:1) [UV].

***t*R** 11.14 min (system A, 24% → 39% acetonitrile in water, 0.1% formic acid).

^{1}H NMR (400 MHz, CD_3OD) δ (ppm) = 7.38 – 7.29 (m, 2H), 7.14 (d, *J* = 2.2 Hz, 1H), 7.07 (dd, *J* = 8.3, 2.3 Hz, 1H), 6.83 (d, *J* = 8.2 Hz, 1H), 4.64 (dd, *J* = 8.8, 5.3 Hz, 1H), 3.14 (dd, *J* = 14.0, 5.3 Hz, 1H), 2.90 (dd, *J* = 14.0, 8.8 Hz, 1H), 1.91 (s, 3H).

^{13}C NMR (101 MHz, CD_3OD) δ (ppm) = 174.9 (C_q), 173.1 (C_q), 154.4 (C_q), 151.9 (ddd, J_{CF} = 246.2, 9.6, 4.2 Hz, C_q), 139.6 (dt, J_{CF} = 248.6, 15.3 Hz, C_q), 137.1 – 136.6 (m, C_q), 132.0 (CH), 131.5 (CH), 129.8 (C_q), 126.2 (C_q), 117.2 (CH), 115.1 – 113.8 (m, 2 × CH), 55.3 (CH), 37.7 (CH_2), 22.3 (CH_2).

^{19}F NMR (376 MHz, CD_3OD) δ (ppm) = -137.1 (m, 2F), -165.5 (m, 1F).

IR (KBr) $\tilde{\nu}$ (cm^{-1}): 3341 (m, br), 1727 (m), 1617 (s), 1530 (s), 1500 (m), 1424 (m), 1375 (m), 1234 (m), 1129 (m), 1044 (s), 861 (m), 746 (m).

HRMS (ESI) (*m/z*) calcd. for $C_{17}H_{15}F_3NO_4$ [M+H$^+$]: 354.0948, found: 354.0948.

$[\alpha]_D^{20}$ = + 21.0 (c = 1.00 in CH_3OH).

3da’

***R*f**	0.2 (CH_2Cl_2/MeOH = 1:1) [UV].
***t*R**	9.37 min (system A, 24% → 39% acetonitrile in water, 0.1% formic acid).
**^{1}H NMR**	(400 MHz, CD_3OD) δ (ppm) = 7.17 – 7.04 (m, 3H), 6.74 (dd, *J* = 8.4, 2.6 Hz, 1H), 6.59 (d, *J* = 2.6 Hz, 1H), 4.35 (dd, *J* = 9.2, 5.5 Hz, 1H), 3.16 (dd, *J* = 14.3, 5.5 Hz, 1H), 2.76 (dd, *J* = 14.3, 9.2 Hz, 1H), 1.84 (s, 3H).
**^{13}C NMR**	(101 MHz, CD_3OD) δ (ppm) = 174.6 (C_q), 172.9 (C_q), 157.4 (C_q), 152.0 (ddd, J_{CF} = 248.8, 9.8, 3.9 Hz, 2 × C_q), 142.0 (C_q), 141.2 – 139.3 (m, C_q), 139.6 – 139.2 (m, CH), 132.8 (CH), 126.3 (CH), 117.6 (CH), 116.2 (CH), 115.3 – 114.5 (m, 2 × CH), 54.3 (CH), 35.3 (CH_2), 22.3 (CH_3).
**^{19}F NMR**	(376 MHz, CD_3OD) δ (ppm) = -135.7 (m, 2F), -164.7 (m, 1F).
**IR (KBr)**	$\tilde{\nu}$ (cm^{-1}): 3343 (m, br), 3072 (m), 2943 (m), 1726 (m), 1617 (s), 1531 (s), 1510 (m), 1410 (m), 1374 (m), 1251 (m), 1128 (m), 1042 (m), 861 (m), 737 (w).
**HRMS (ESI)**	(*m/z*) calcd. for $C_{17}H_{15}F_3NO_4$ [M+H$^+$]: 354.0948, found: 354.0946.

$[\alpha]_D^{20}$ = + 22.1 (c = 1.00 in CH_3OH).

[(*S*)-2-Amino-3-(3',4',5'-trifluoro-6-hydroxy-[1,1'-biphenyl]-3-yl)propanoyl]-*L*-phenylalanine (3ea) and its regioisomers [(*S*)-2-Amino-3-(3',4',5'-trifluoro-5-hydroxy-[1,1'-biphenyl]-2-yl)propanoyl]-*L*-phenylalanine (**3ea'**) **and** (***S***)**-2-[(*S*)-2-Amino-3-(4-hydroxyphenyl)propanamido]-3-(3',4',5'-trifluoro-[1,1'-biphenyl]-2-yl)propanoic acid** (**3ea''**)

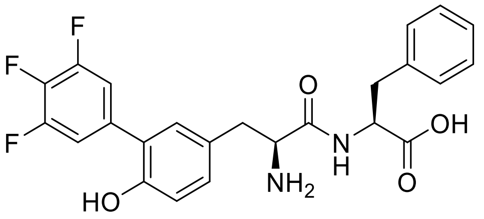

$C_{24}H_{21}F_3N_2O_4$
458.44 g/mol

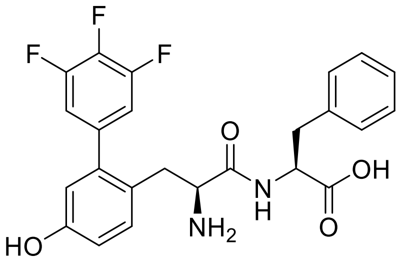

$C_{24}H_{21}F_3N_2O_4$
458.44 gmol

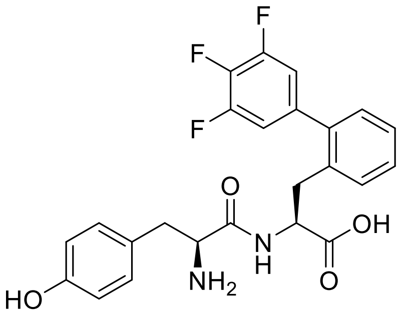

$C_{24}H_{21}F_3N_2O_4$
458.44 g/mol

Compound **3ea, 3ea'** and **3ea''** were prepared according to **GP6**, using H-Tyr-Phe-OH **(**164 mg, 0.50 mmol, 1 equiv.) and **2a** (0.3 M solution, 1.1 equiv. and 2 × 0.75 equiv.). 1 mL of 1 M HCl, 0.2 mL of water and 0.7 mL of acetonitrile were used as solvents. Analysis by ^{1}H NMR gave **3ea** in 38%, **3ea'** (minor isomer) in 6.4% and **3ea''** (minor isomer) in traces. Purification by preparative HPLC (system A, gradient: 24% → 36% acetonitrile in water, 0.1% formic acid) gave **3ea** and **3ea'** as colorless powders.

3ea

***R*f** 0.5 (CH_2Cl_2/MeOH = 17:3) [UV].

***t*R** 6.43 min (system A, 24% → 36% acetonitrile in water, 0.1% formic acid).

^{1}H NMR (400 MHz, CD_3OD) δ (ppm) = 7.41 – 7.31 (m, 2H), 7.27 – 7.19 (m, 5H), 7.20 – 7.10 (m, 1H), 7.13 (dd, *J* = 8.4, 2.3 Hz, 1H), 6.88 (d, *J* = 8.3 Hz, 1H), 4.52 (dd, *J* = 8.2, 4.8 Hz, 1H), 4.01 (dd, *J* = 8.6, 4.8 Hz, 1H), 3.21 (ddd, *J* = 16.2,

14.3, 4.8 Hz, 2H), 3.00 (dd, J = 13.9, 8.2 Hz, 1H), 2.92 (dd, J = 14.6, 8.6 Hz, 1H).

^{13}C NMR (101 MHz, CD_3OD) δ (ppm) = 176.8 (C_q), 169.5 (C_q), 155.2 (C_q), 151.9 (ddd, J_{CF} = 246.3, 9.8, 4.2 Hz, 2 × C_q), 139.6 (dt, J_{CF} = 249.0, 15.6 Hz, C_q), 139.4 (C_q), 136.7 – 136.3 (m, C_q), 132.4 (CH), 131.9 (CH), 130.5 (2 × CH), 129.3 (2 × CH), 127.5 (CH), 127.0 (C_q),126.7 (C_q), 117.8 (CH), 115.3 – 113.7 (m, 2 × CH), 57.5 (CH), 55.8 (CH), 39.0 (CH_2), 37.8 (CH_2).

^{19}F NMR (376 MHz, CD_3OD) δ (ppm) = -135.7 (m, 2F), -164.8 (m, 1F).

IR (KBr) $\tilde{\nu}$ (cm^{-1}): 3382 (m, br), 1675 (m), 1617 (m), 1532 (s), 1410 (m), 1253 (w), 1042 (m), 701 (w).

HRMS (ESI) (*m/z*) calcd. for $C_{24}H_{22}F_3N_2O_4$ [$M+H^+$]: 459.1526, found: 459.1525.

$[\alpha]_D^{20}$ = + 9.5 (c = 0.50 in CH_3OH).

3ea’

***R*f** 0.5 (CH_2Cl_2/MeOH = 17:3) [UV].

***t*R** 5.00 min (system A, 24% → 36% acetonitrile in water, 0.1% formic acid).

^{1}H NMR (400 MHz, CD_3OD) δ (ppm) = 7.26 – 7.15 (m, 6H), 7.11 – 7.04 (m, 2H), 6.80 (dd, J = 8.4, 2.7 Hz, 1H), 6.62 (d, J = 2.6 Hz, 1H), 4.36 (dd, J = 7.6, 5.1 Hz, 1H), 3.69 (dd, J = 8.3, 6.7 Hz, 1H), 3.18 (dd, J = 13.8, 5.1 Hz, 1H), 3.11 (dd, J = 14.6, 6.7 Hz, 1H), 2.98 (dd, J = 13.8, 7.6 Hz, 1H), 2.84 (dd, J = 14.6, 8.3 Hz, 1H).

^{13}C NMR (101 MHz, CD_3OD) δ (ppm) = 176.4 (C_q), 169.4 (C_q), 157.9 (C_q), 154.1 – 150.5 (m, 2 × C_q), 142.2 (C_q), 139.3 (C_q), 142.3 – 136.4 (m, C_q), 132.4 (CH), 130.4 (2 × CH), 129.2 (2 × CH), 127.4 (CH), 123.6 (C_q), 118.3 (CH), 117.1 (CH), 115.3 – 114.7 (m, 2 × CH), 57.6 (CH), 55.5 (CH), 38.8 (CH_2), 34.9 (CH_2). One carbon signal missing.

^{19}F NMR (376 MHz, CD_3OD) δ (ppm) = -135.0 (m, 2F), -164.1 (m, 1F).

HRMS (ESI) (*m/z*) calcd. for $C_{24}H_{22}F_3N_2O_4$ [$M+H^+$]: 459.1526, found: 459.1526.

3ea’’

***R*f** 0.5 (CH_2Cl_2/MeOH = 17:3) [UV].

***t*R** 6.33 min (system A, 24% → 36% acetonitrile in water, 0.1% formic acid).

^{1}H NMR (400 MHz, CD_3OD) δ (ppm) = 7.38 (dd, J = 7.7, 1.4 Hz, 1H), 7.31 (td, J = 7.5, 1.6 Hz, 1H), 7.25 (td, J = 7.5, 1.5 Hz, 1H), 7.17 – 7.09 (m, 3H), 7.09 – 7.02 (m, 2H), 6.76 – 6.71 (m, 2H), 4.37 (dd, J = 9.1, 5.9 Hz, 1H), 3.85 (dd, J = 8.7, 4.8 Hz, 1H), 3.22 (dd, J = 14.4, 6.0 Hz, 1H), 3.11 (dd, J = 14.6, 4.9 Hz, 1H), 2.92 (dd, J = 14.4, 9.1 Hz, 1H), 2.81 (dd, J = 14.5, 8.7 Hz, 1H).

^{13}C NMR (101 MHz, CD_3OD) δ (ppm) = 177.1 (C_q), 169.6 (C_q), 158.1 (C_q), 152.0 (ddd, J_{CF} = 248.8, 10.0, 4.1 Hz, 2 × C_q), 141.0 (C_q), 140.2 (dt, J_{CF} = 249.1, 15.2 Hz, C_q), 139.6 – 139.3 (m, C_q), 136.8 (C_q), 131.6 (2 × CH), 131.3 (CH), 130.8 (CH), 129.3 (CH), 127.6 (CH), 126.4 (C_q), 116.8 (2 × CH), 115.3 – 114.8 (m, 2 × CH), 56.7 (CH), 56.0 (CH), 37.9 (CH_2), 36.7 (CH_2).

^{19}F NMR (376 MHz, CD_3OD) δ (ppm) = -135.7 (m, 2F), -164.8 (m, 1F).

HRMS (ESI) (*m/z*) calcd. for $C_{24}H_{22}F_3N_2O_4$ [M+H$^+$]: 459.1526, found: 459.1525.

[(*S*)-2-Acetamido-3-(3',4',5'-trifluoro-6-hydroxy-[1,1'-biphenyl]-3-yl)propanoyl]-*L*-phenylalanine (3fa)

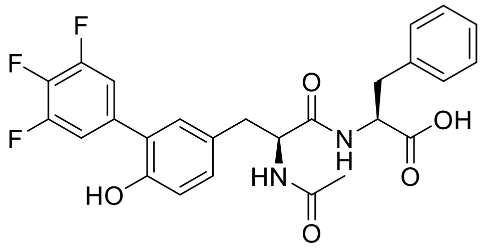

$C_{26}H_{23}F_3N_2O_5$
500.47 g/mol

Compound **3fa** was prepared according to **GP6**, using **1f (**185 mg, 0.50 mmol, 1 equiv.) applying **2a (**0.3 M solution, 1.1 equiv. and 0.75 equiv.). 1.5 mL of 1 M HCl and 5 mL of water were used as solvents and the reaction mixture was overall only irradiated for 2 h + 2 h with only adding 0.75 equiv. of the diazonium solution **2a**. Analysis by standard ^{1}H NMR gave **3fa** in 21%. Purification by preparative HPLC (system A, gradient: 32% → 47% acetonitrile in water, 0.1% formic acid) gave **3fa** as colorless powder.

t_R 11.87 min (system A, 32% → 47% acetonitrile in water, 0.1% formic acid).

^{1}H NMR (400 MHz, CD_3OD) δ (ppm) = 7.38 – 7.30 (m, 2H), 7.28 – 7.17 (m, 5H), 7.17 (d, *J* = 2.2 Hz, 1H), 7.05 (dd, *J* = 8.3, 2.3 Hz, 1H), 6.80 (d, *J* = 8.3 Hz, 1H), 4.70 – 4.52 (m, 2H), 3.20 (dd, *J* = 13.9, 5.1 Hz, 1H), 3.04 (dd, *J* = 14.0, 5.5 Hz, 1H), 2.99 (dd, *J* = 13.9, 7.9 Hz, 1H), 2.74 (dd, *J* = 14.0, 9.2 Hz, 1H), 1.86 (s, 3H).

^{13}C NMR (101 MHz, CD_3OD) δ (ppm) = 174.7 (C_q), 173.2 (C_q), 173.0 (C_q), 154.3 (Cq), 151.9 (ddd, J_{CF} = 246.1, 9.8, 4.1 Hz, 2 × C_q), 139.6 (dt, J_{CF} = 248.5, 15.6 Hz, C_q), 138.4 (C_q), 137.0 – 136.7 (m, C_q), 132.0 (CH), 131.5 (CH), 130.5 (2 × CH), 129.9 (C_q), 129.4 (2 × CH), 127.7 (CH), 126.2 (C_q), 117.2 (CH), 114.7 – 114.2 (m, 2 × CH), 56.0 (CH), 55.3 (CH_3), 38.6 (CH_2), 38.0 (CH_2), 22.4 (CH_3).

^{19}F NMR (376 MHz, CD_3OD) δ (ppm) = -137.2 (m, 2F), -165.6 (m, 1F).

IR (KBr) $\tilde{\nu}$ (cm^{-1}): 3342 (m), 1745 (s), 1617 (s), 1530 (s), 1509 (m), 1408 (m), 1374 (w), 1253 (w), 1170 (w), 1041 (m), 860 (w), 755 (w), 701 (m).

HRMS (ESI) (*m/z*) calcd. for $C_{26}H_{24}F_3N_2O_5$ [M+H$^+$]: 501.1631, found: 501.1638.

$[\alpha]_D^{20}$ = + 3.7 (c = 1.00 in CH_3OH).

Methyl (*S*)-[2-acetamido-3-(3',4',5'-trifluoro-6-hydroxy-[1,1'-biphenyl]-3-yl)propanoyl]glycinate (3ga) and its regioisomer methyl (*S*)-[2-acetamido-3-(3',4',5'-trifluoro-5-hydroxy-[1,1'-biphenyl]-2-yl)propanoyl]glycinate (**3ga'**)

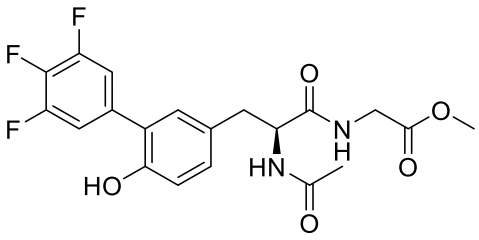

$C_{20}H_{19}F_3N_2O_5$
424.38 g/mol

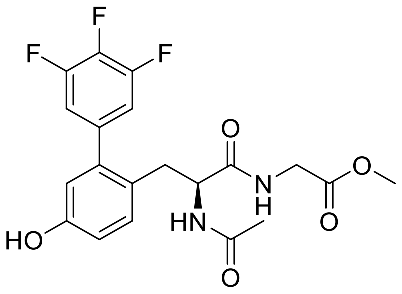

$C_{20}H_{19}F_3N_2O_5$
424.38 g/mol

Compound **3ga** and **3ga'** were prepared according to **GP6**, using **1g (**147 mg, 0.50 mmol, 1 equiv.) aapplying **2a** (0.3 M solution, 1.1 equiv. and 2 × 0.75 equiv.). Analysis by standard ^{1}H NMR gave **3ga** in 66% and **3ga'** (minor isomer) in 11%. Purification by preparative HPLC (system A, gradient: 24% → 39% acetonitrile in water, 0.1% formic acid) gave **3ga** and **3ga'**as colorless powders.

3ga

***R*f** 0.1 (CH_2Cl_2/MeOH = 1:1) [UV].

***t*R** 12.52 min (system A, 24% → 39% acetonitrile in water, 0.1% formic acid).

^{1}H NMR (400 MHz, CD_3OD) δ (ppm) = 7.41 – 7.32 (m, 2H), 7.20 (d, *J* = 2.2 Hz, 1H), 7.09 (dd, *J* = 8.3, 2.3 Hz, 1H), 6.82 (d, *J* = 8.3 Hz, 1H), 4.64 (dd, *J* = 8.9, 5.9 Hz, 1H), 4.01 – 3.85 (m, 2H), 3.70 (s, 3H), 3.12 (dd, *J* = 13.9, 5.8 Hz, 1H), 2.83 (dd, *J* = 14.0, 8.8 Hz, 1H), 1.91 (s, 3H).

^{13}C NMR (101 MHz, CD_3OD) δ (ppm) = 174.2 (C_q), 173.2 (C_q), 171.5 (C_q), 154.4 (Cq), 151.9 (ddd, J_{CF} = 246.1, 9.8, 4.2 Hz, 2 × C_q), 139.6 (dt, J_{CF} = 248.5, 15.6 Hz, C_q), 136.8 (dt, J_{CF} = 8.3, 4.0 Hz, C_q), 132.1 (CH), 131.5 (CH), 129.8 (C_q), 126.2 (C_q), 117.1 (CH), 115.0 – 114.0 (m, 2 × CH), 56.0 (CH), 52.6 (CH_3), 41.8 (CH_2), 38.1 (CH_2), 22.4 (CH_3).

^{19}F NMR (376 MHz, CD_3OD) δ (ppm) = -137.2 (m, 2F), -165.6 (m, 1F).

IR (KBr) $\tilde{\nu}$ (cm^{-1}): 3308 (s, br), 2959 (m), 1750 (s), 1659 (s), 1532 (s), 1403 (s), 1369 (m), 1217 (s), 1133 (m), 1038 (s), 863 (m).

HRMS (ESI) (*m/z*) calcd. for $C_{20}H_{20}F_3N_2O_5$ [M+H$^+$]: 425.1319, found: 425.1321.

$[\alpha]_D^{20}$ = + 6.7 (c = 1.00 in CH_3OH).

3ga'

***R*f** 0.1 (CH_2Cl_2/MeOH = 1:1) [UV].

***t*R** 10.70 (system A, 24% → 39% acetonitrile in water, 0.1% formic acid).

^{1}H NMR (400 MHz, CD_3OD) δ (ppm) = 7.14 (d, *J* = 8.4 Hz, 1H), 7.10 (dd, *J* = 8.6, 6.7 Hz, 2H), 6.75 (dd, *J* = 8.4, 2.7 Hz, 1H), 6.59 (d, *J* = 2.6 Hz, 1H), 4.42 (dd, *J* = 8.7, 6.3 Hz, 1H), 3.85 (d, *J* = 1.7 Hz, 2H), 3.69 (s, 3H), 3.12 (dd, *J* = 14.4, 6.3 Hz, 1H), 2.79 – 2.62 (m, 1H), 1.85 (s, 3H).

^{13}C NMR (101 MHz, CD_3OD) δ (ppm) = 173.9 (C_q), 173.0 (C_q), 171.4 (C_q), 157.3 (Cq), 153.6 – 150.7 (m, 2 × C_q), 142.0 (C_q), 139.5 – 139.1 (m, C_q), 132.7 (CH), 126.2 (C_q), 117.6 (CH), 116.2 (CH), 115.5 – 114.4 (m, 2 × CH), 55.0 (CH), 52.6 (CH_3), 41.8 (CH_2), 35.2 (CH_2), 22.4 (CH_3). One quaternary carbon signal missing.

^{19}F NMR (376 MHz, CD_3OD) δ (ppm) = -135.6 (m, 2F), -164.7 (m, 1F).

HRMS (ESI) (*m/z*) calcd. for $C_{20}H_{20}F_3N_2O_5$ [M+H$^+$]: 425.1319, found: 425.1321.

4-Amino-5-{[(*S*)-1-carboxy-2-(3',4',5'-trifluoro-6-hydroxy-[1,1'-biphenyl]-3-yl)ethyl]amino}-5-oxopentanoic acid (3ha)

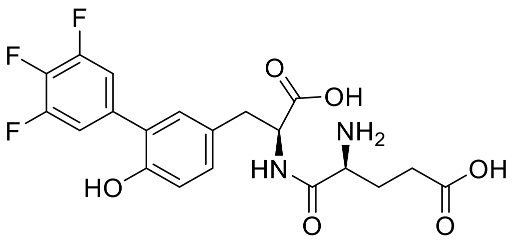

$C_{20}H_{19}F_3N_2O_6$
440.38 g/mol

Compound **3ha** was prepared according to **GP6**, using H-Glu-Tyr-OH (**1h**) **(**155 mg, 0.50 mmol, 1 equiv.) and **2a (**0.3 M solution, 1.1 equiv. and 2 × 0.75 equiv.). Analysis by standard ^{1}H NMR gave **3ha** in 62%. Purification by preparative HPLC (system A, gradient: 11% → 27% acetonitrile in water, 0.1% formic acid) gave **3ha** as colorless powder.

***R*f** 0.3 (ethyl acetate) [UV].

***t*R** 10.54 min (system A, 11% → 27% acetonitrile in water, 0.1% formic acid).

^{1}H NMR (400 MHz, CD_3OD) δ (ppm) = 7.41 (dd, *J* = 9.4, 6.9 Hz, 2H), 7.34 – 7.15 (m, 7H), 6.94 (d, *J* = 8.3 Hz, 1H), 4.64 (dd, *J* = 9.0, 5.7 Hz, 1H), 4.34 (dd, *J* = 9.9, 4.7 Hz, 1H), 4.20 (dd, *J* = 8.1, 6.1 Hz, 1H), 4.03 – 3.72 (m, 4H), 3.26 (dd, *J* = 14.2, 6.2 Hz, 1H), 3.18 (dd, *J* = 13.9, 5.8 Hz, 1H), 3.06 (dd, *J* = 14.2, 8.3 Hz, 1H), 3.00 (dd, *J* = 14.0, 9.2 Hz, 1H), 1.75 – 1.51 (m, 3H), 0.95 (d, *J* = 6.0 Hz, 3H), 0.90 (d, *J* = 6.1 Hz, 3H).

^{13}C NMR (101 MHz, CD_3OD) δ (ppm) = 178.2 (C_q), 176.2 (C_q), 169.8 (C_q), 154.3 (C_q), 151.9 (ddd, J_{CF} = 246.1, 9.8, 4.2 Hz, 2 × C_q), 139.6 (dt, J_{CF} = 248.8, 15.7 Hz, C_q), 137.0 – 136.6 (m, C_q), 132.0 (CH), 131.6 (CH), 130.5 (C_q), 126.1 (C_q), 117.2 (CH), 114.6 – 114.2 (m, 2 × CH), 57.3 (CH), 54.2 (CH), 37.9 (C_q), 32.6 (C_q), 28.5 (C_q).

^{19}F NMR (376 MHz, CD_3OD) δ (ppm) = -137.1 (m, 2F), -165.6 (m, 1F).

IR (KBr) $\tilde{\nu}$ (cm^{-1}): 3290 (m, br), 2919 (m), 2359 (m), 1699 (m), 1653 (s), 1558 (m), 1541 (m), 1457 (m), 1025 (m), 669 (w).

HRMS (ESI) (*m/z*) calcd. for $C_{20}H_{20}F_3N_2O_6$ [M+H$^+$]: 441.1268, found: 441.1269.

$[\alpha]_D^{20}$ = + 21.6 (c = 0.50 in CH_3OH).

(*S*)-2-(2-Benzamidoacetamido)-3-(3',4',5'-trifluoro-6-hydroxy-[1,1'-biphenyl]-3-yl)propanoic acid (3ia)

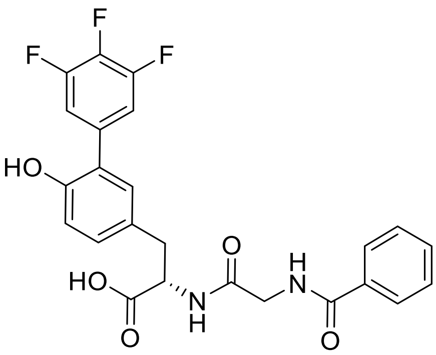

$C_{24}H_{19}F_3N_2O_5$
472.42 g/mol

Compound **3ia** was prepared according to **GP6**, using **1i** (171 mg, 0.50 mmol, 1 equiv.) applying **2a** (0.3 M solution, 1.1 equiv. and 2 × 0.75 equiv.). Analysis by standard ^{1}H NMR gave **3ia** in 24%. Purification by preparative HPLC (system A, gradient: 32% → 47% acetonitrile in water, 0.1% formic acid) gave **3ia** as colorless powder.

***t*R** 10.91 min (system A, 32% → 47% acetonitrile in water, 0.1% formic acid).

^{1}H NMR (400 MHz, CD_3OD) δ (ppm) = 7.84 – 7.78 (m, 2H), 7.57 – 7.50 (m, 1H), 7.48 – 7.41 (m, 2H), 7.32 (ddd, *J* = 9.5, 6.8, 3.4 Hz, 2H), 7.14 (d, *J* = 2.2 Hz, 1H), 7.06 (dd, *J* = 8.3, 2.3 Hz, 1H), 6.78 (d, *J* = 8.3 Hz, 1H), 4.70 (dd, *J* = 7.6, 5.2 Hz, 1H), 4.10 – 3.95 (m, 2H), 3.15 (dd, *J* = 14.0, 5.2 Hz, 1H), 3.00 (dd, *J* = 14.0, 7.6 Hz, 1H).

^{13}C NMR (101 MHz, CD_3OD) δ (ppm) = 174.7 (C_q), 171.6 (C_q), 170.5 (C_q), 154.6 (Cq), 151.9 (ddd, J_{CF} = 246.2, 9.8, 4.3 Hz, 2 × C_q), 139.6 (dt, J_{CF} = 248.4, 15.4 Hz, C_q), 136.9 – 136.6 (m, C_q), 135.0 (C_q), 133.0 (CH), 132.1 (CH), 131.7 (CH), 129.6 (2 × CH), 129.5 (C_q), 128.5 (2 × CH), 126.3 (C_q), 117.4 (CH), 114.9 – 114.2 (m, 2 × CH), 55.2 (CH), 44.0 (CH_2), 37.7 (CH_2).

^{19}F NMR (376 MHz, CD_3OD) δ (ppm) = -137.1 (m, 2F), -165.5 (m, 1F).

IR (KBr) $\tilde{\nu}$ (cm^{-1}): 3124 (s, br), 1745 (w), 1616 (m), 1529 (m), 1402 (s), 1252 (w), 1041 (m), 701 (w).

HRMS (ESI) (*m/z*) calcd. for $C_{24}H_{20}F_3N_2O_3$ [M+H$^+$]: 473.1319, found: 473.1321.

$[\alpha]_D^{20}$ = + 37.2 (c = 1.00 in CH_3OH).

(*S*)-1-(*L*-Arginyl-*L*-arginyl)-*N*-[(*S*)-1-{[(2*S*,3*S*)-1-{[(*S*)-1-amino-4-methyl-1-oxopentan-2-yl]amino}-3-methyl-1-oxopentan-2-yl]amino}-1-oxo-3-(3',4',5'-trifluoro-6-hydroxy-[1,1'-biphenyl]-3-yl)propan-2-yl]pyrrolidine-2-carboxamide (3ja)

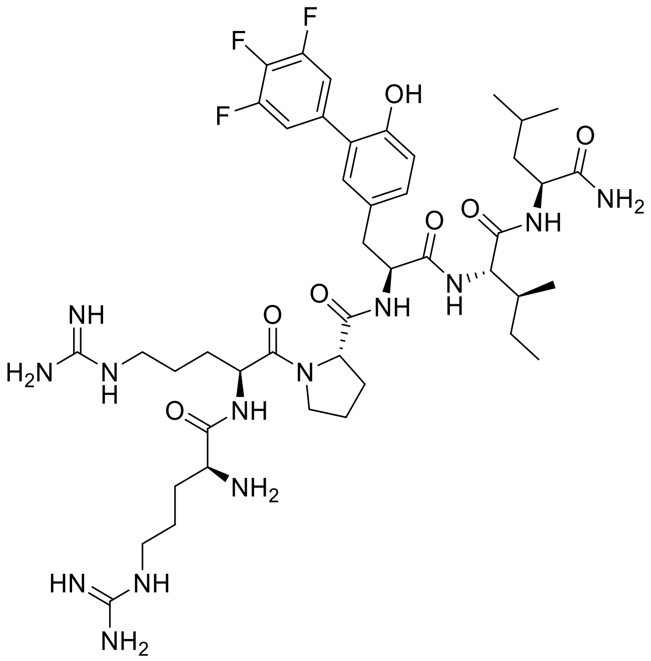

$C_{44}H_{66}F_3N_{13}O_7$
946.09 g/mol

Compound **3ja** was prepared according to **GP6**, using **1j (**81.6 mg, 0.10 mmol, 1 equiv.) and **2a** (0.3 M solution, 1.1 equiv. and 2 × 0.75 equiv.). Analysis of the ratio of **1j**/**3ja** by ^{1}H NMR led to a yield of **3ja** of 28%. Purification by preparative HPLC (system B, gradient: 5% → 30% acetonitrile in water, 0.1% trifluoroacetic acid) gave **3ja** as colorless powder.

***t*R** 7.00 min (system B, 5% → 30% acetonitrile in water, 0.1% trifluoroacetic acid).

^{1}H NMR (400 MHz, CD_3OD) δ (ppm) = 7.29 (dd, *J* = 9.1, 6.7 Hz, 2H), 7.18 – 7.09 (m, 2H), 6.92 (d, *J* = 8.2 Hz, 1H), 4.67 – 4.56 (m, 2H), 4.38 (dd, *J* = 8.3, 5.8 Hz, 1H), 4.17 (t, *J* = 7.3 Hz, 1H), 4.10 (d, *J* = 8.1 Hz, 1H), 4.02 (t, *J* = 6.4 Hz, 1H), 3.87 – 3.74 (m, 1H), 3.69 – 3.52 (m, 1H), 3.17 (t, *J* = 6.6 Hz, 2H), 3.11 (t, *J* = 6.9 Hz, 2H), 3.01 (d, *J* = 7.4 Hz, 2H), 2.31 – 2.15 (m, 1H), 2.03 – 1.92 (m, 2H), 1.91 – 1.46 (m, 13H), 1.43 – 1.26 (m, 1H), 1.14 – 1.01 (m, 1H), 0.90 – 0.68 (m, 12H).

^{13}C NMR (101 MHz, CD_3OD) δ (ppm) = 178.3 (C_q), 174.8 (C_q), 174.0 (C_q), 173.8 (C_q), 172.6 (C_q), 170.7 (C_q), 158.1 (C_q), 158.0 (C_q), 153.0 (C_q), 153.4 – 150.5 (m, 2 × C_q), 139.0 – 135.0 (m, C_q), 134.6 – 133.9 (m, C_q), 132.3 (CH), 131.8 (CH), 129.5 (C_q), 127.1 (C_q), 117.8 (CH), 115.1 – 114.6 (m, 2 × CH), 61.8 (CH), 59.2 (CH), 56.1 (CH), 53.8 (CH), 53.5 (CH), 53.0 (CH), 49.3 (CH_2), 41.9 (CH_2), 41.7 (CH_2), 41.0 (CH_2), 37.9 (CH), 37.3 (CH_2), 30.7 (CH_2), 29.4 (CH_2), 28.5 (CH_2), 26.0 (CH_2), 25.71 (CH), 25.65 (CH_2), 25.4 (CH_2), 24.6 (CH_2), 23.3 (CH_3), 22.2 (CH_3), 15.9 (CH_3), 11.3 (CH_3).

^{19}F NMR (376 MHz, CD_3OD) δ (ppm) = -134.8 (m, 2F), -162.2 (m, 1F).

IR (KBr) $\tilde{\nu}$ (cm^{-1}): 3351 (m, br), 2966 (w), 1675 (s), 1532 (m), 1436 (w), 1204 (s), 1136 (m), 1043 (m), 839 (w), 802 (m), 723 (m).

HRMS (ESI) (*m/z*) calcd. For $C_{44}H_{68}F_3N_{13}O_7$ [$M+2H^+$]: 473.7653, found: 473.7655.

$[\alpha]_D^{20}$ = -29.9 (c = 0.10 in CH_3OH).

(*S*)-2-[(*S*)-2-(2-{2-[(*S*)-2-Amino-3-(3',4',5'-trifluoro-6-hydroxy-[1,1'-biphenyl]-3-yl)propanamido]acetamido}acetamido)-3-phenylpropanamido]-4-methylpentanamide (3ka)

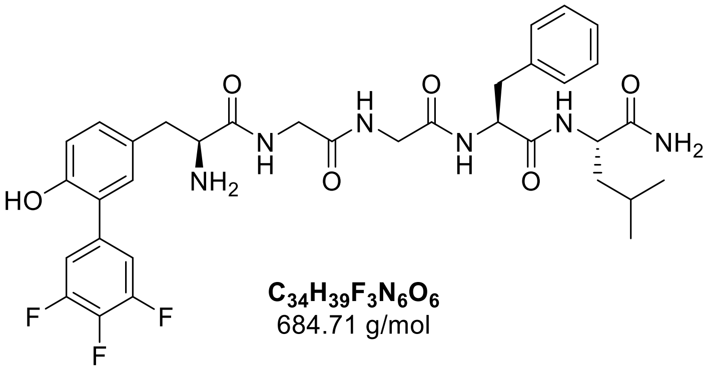

Compound **3ka** was prepared according to **GP6**, using **1k** (50.0 mg, 0.09 mmol, 1 equiv.) and **2a** (0.3 M solution, 1.1 equiv. and 2 × 0.75 equiv.). Analysis by standard ^{1}H NMR gave **3ia** in 38%. Purification by preparative HPLC (system B, gradient: 10% → 45% acetonitrile in water, 0.1% trifluoroacetic acid) gave **3ka** as colorless powder.

***t*R** 9.04 min (system B, 10% → 45% acetonitrile in water, 0.1% trifluoroacetic acid).

^{1}H NMR (400 MHz, CD_3OD) δ (ppm) = 7.39 (dd, *J* = 9.4, 6.9 Hz, 2H), 7.32 – 7.13 (m, 7H), 6.93 (d, *J* = 8.3 Hz, 1H), 4.62 (dd, *J* = 9.0, 5.7 Hz, 1H), 4.32 (dd, *J* = 9.9, 4.7 Hz, 1H), 4.18 (dd, *J* = 8.1, 6.1 Hz, 1H), 4.01 – 3.70 (m, 4H), 3.24 (dd, *J* = 14.2, 6.2 Hz, 1H), 3.16 (dd, *J* = 13.9, 5.8 Hz, 1H), 3.04 (dd, *J* = 14.2, 8.3 Hz, 1H), 2.98 (dd, *J* = 14.0, 9.2 Hz, 1H), 1.73 – 1.49 (m, 3H), 0.93 (d, *J* = 6.0 Hz, 3H), 0.88 (d, *J* = 6.1 Hz, 3H).

^{13}C NMR (101 MHz, CD_3OD) δ (ppm) = 177.2 (C_q), 173.6 (C_q), 171.5 (2 × C_q), 170.8 (C_q), 155.4 (C_q), 151.9 (ddd, J_{CF} = 246.4, 9.8, 4.0 Hz, 2 × C_q), 139.7 (dt, J_{CF} = 249.6, 15.8 Hz, C_q), 138.2 (C_q), 136.8 – 135.9 (m, C_q), 132.4 (CH), 131.7 (CH), 130.4 (2 × CH), 129.6 (2 × CH), 127.9 (CH), 126.9 (C_q), 126.7 (C_q), 117.8 (CH), 114.8 – 114.2 (m, 2 × C_q), 56.3 (CH), 56.0 (CH), 53.0 (CH), 43.7 (CH_2), 43.3 (CH_2), 41.7 (CH_2), 38.6 (CH_2), 37.6 (CH_2), 25.8 (CH), 23.5 (CH_3), 21.8 (CH_3).

^{19}F NMR (376 MHz, CD_3OD) δ (ppm) = -136.8 (m, 2F), -165.0 (m, 1F).

IR (KBr) $\tilde{\nu}$ (cm^{-1}): 3298 (m, br), 3071 (m), 2961 (m), 1671 (s), 1532 (m), 1412 (m), 1203 (m), 1137 (m), 1042 (m), 838 (w), 801 (w), 723 (w).

HRMS (ESI) (*m/z*) calcd. for $C_{34}H_{40}F_3N_6O_6$ $[M+H^+]$: 685.2956, found: 685.2953.

$[\boldsymbol{\alpha}]_{\boldsymbol{D}}^{\mathbf{20}}$ = + 33.4 (c = 0.50 in CH_3OH).

4.5 Arylation of insulin

Enzymatic cleavage of peptides (GP7)

The corresponding peptide (1.0 mg) was suspended in 0.1 M TRIS-HCl-buffer containing 10 mM $CaCl_2$ (50 µL) and 2,2,2-trifluoroethanol (50 µL). After the addition of 0.2 M *D,L*-dithiothreitol (4 µL) the mixture was incubated for 10 min at 60 °C. The mixture was left to cool down to room temperature and 0.2 M 2-iodoacetamide (16 µL) subsequently was added. After incubating for 60 min at room temperature under light protection, 0.2 M *D,L*-dithiothreitol (4 µL) was added and a further incubation step for 60 min at room temperature under light protection was started. Subsequently high purified water (600 µL) and 0.1 M TRIS-HCl-buffer containing 10 mM $CaCl_2$ (200 µL) were added and the pH value was checked (pH = 7.5 to 8.3). To start the enzymatic cleavage, a 1 mg/ml *α*-chymotrypsin stock solution (20 µL; corresponding a peptide to enzyme ratio (m/m) of 50:1) was added and the solution was mixed afterwards. The mixture was incubated at 37 ° C overnight and a 50 µL sample was collected after 0 min, 30 min, 120 min, 240 min and overnight. To stop the enzymatic cleavage after collecting the sample, formic acid (5 µL) was added and the sample was cooled to – 20 °C.

3',4',5'-Trifluorophenyl[B16]insulin (3la)

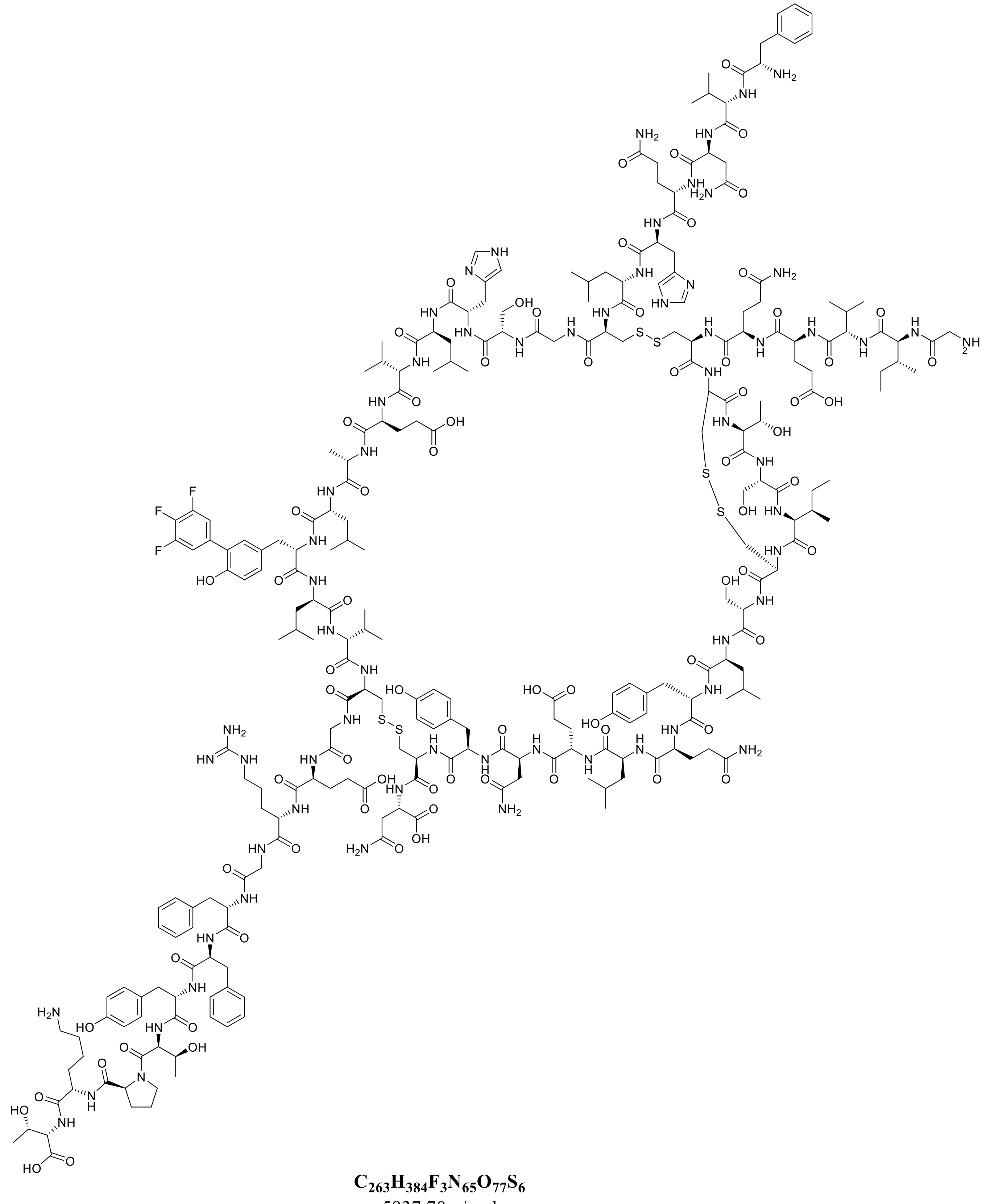

$C_{263}H_{384}F_3N_{65}O_{77}S_6$
5937.70 g/mol

Compound **3la** was prepared according to **GP6**, using insulin (29.0 mg, 5.00 µmol, 1.0 equiv.), **2a** (0.3 M solution, 1.1 equiv. and 2 × 0.75 equiv.) and acetonitrile (2.00 mL per 1 mmol substrate). Extraction was performed by using methyl *tert*-butyl ether. The product precipitated between the two phases and was filtered under vacuum. The precipitate was treated according to **GP7.** Analysis of the cleavage fragments was performed by LC-MS.

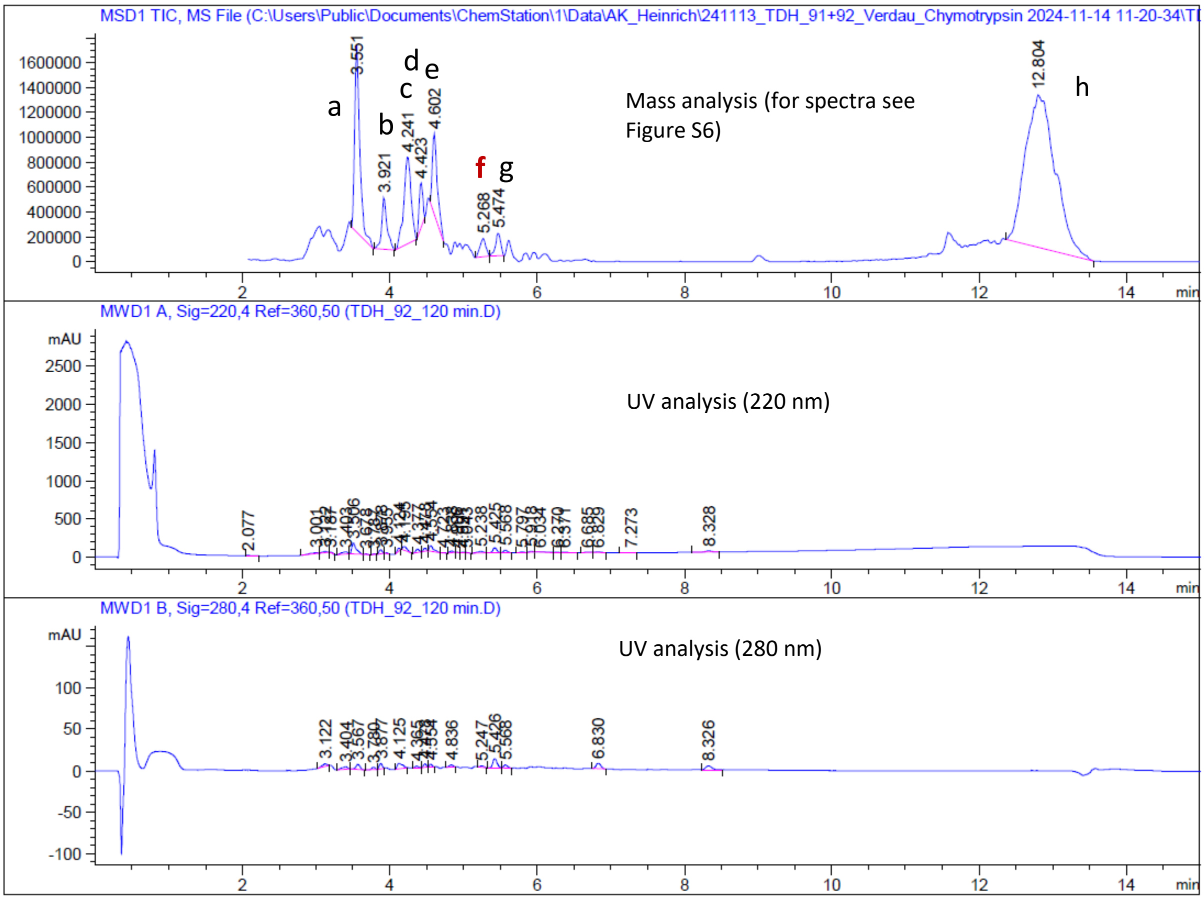

Figure S5. Example for a LC-MS run of enzymatic cleavage sample of **3la** collected after 120 min of incubation.

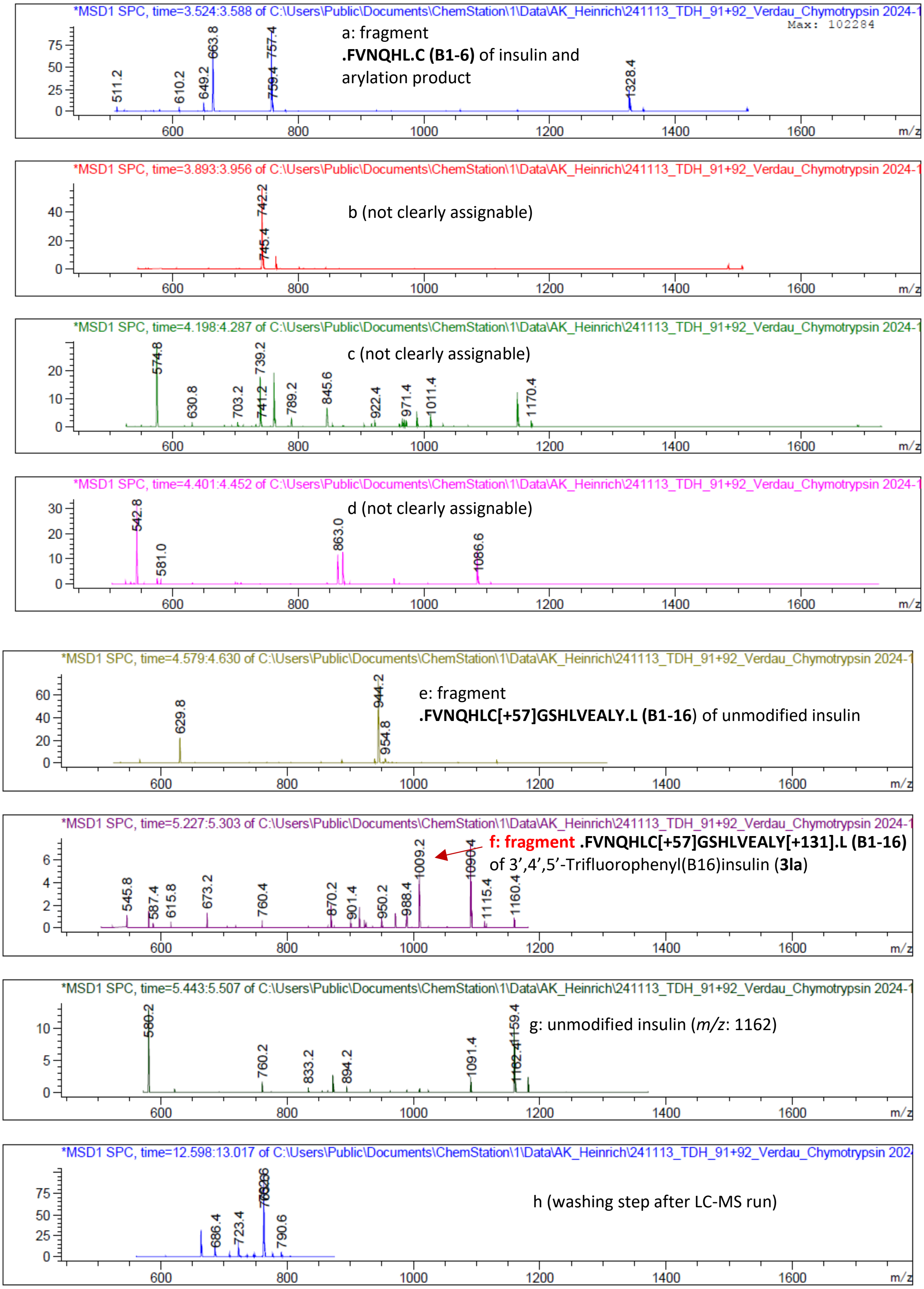

Figure S6. Exemplary mass spectra data of enzymatic cleavage sample of **3la** shown in **S5** collected after 120 min of incubation.

a.

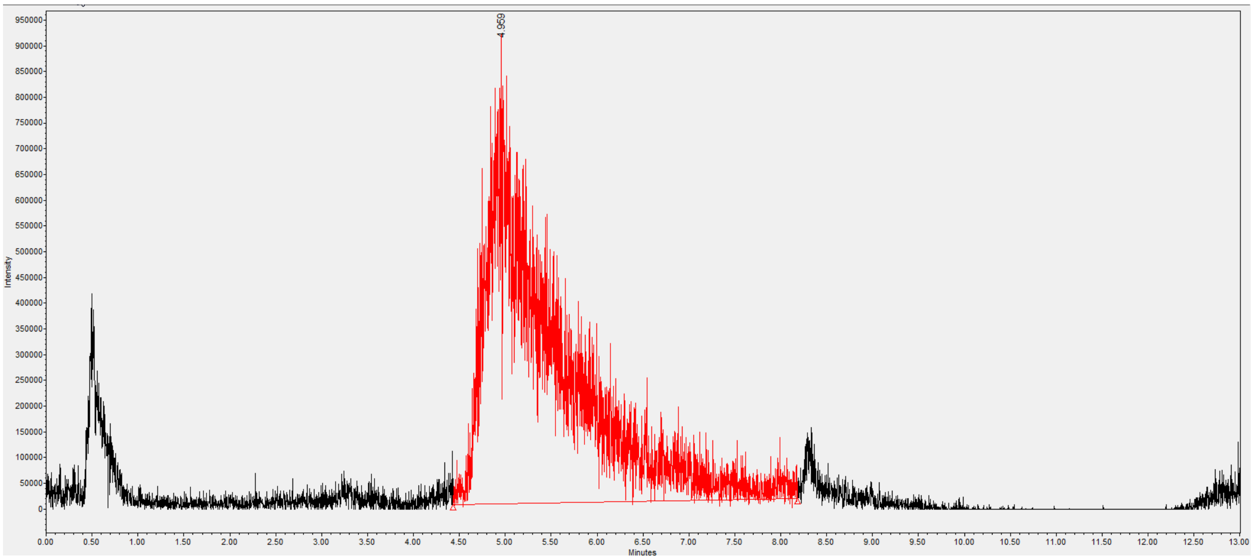

b.

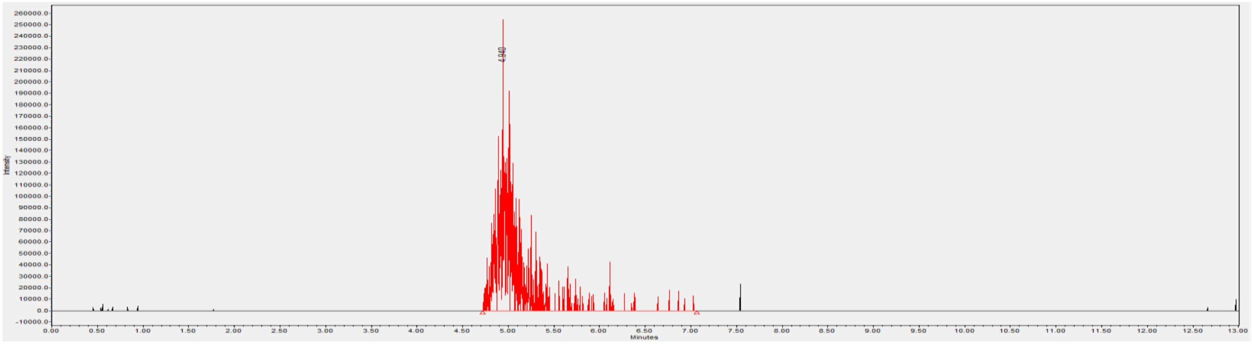

c.

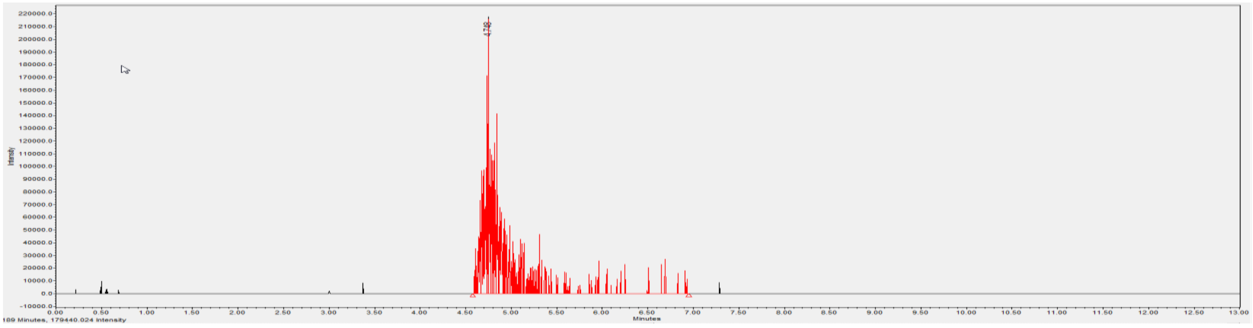

Figure S7. MS data of LC-MS analysis before enzymatic digestion of compound **3la**. **a.** TIC plot. **b.** *m/z* = 1188 (**3la**), AUC = 1590000. **c.** *m/z* = 1162 (insulin), AUC = 1060000.

4.6 Control reactions with other aromatic amino acids

General procedure for irradiation experiments with variated, not water soluble peptides (GP8)

The respective peptide **(**1 equiv.) and a 0.3 M solution of 3,4,5-trifluorophenyldiazonium chloride (**2a) (**1.1 equiv.) were added to acetonitrile (2 mL per 1 mmol substrate) and 1 M HCl (1.0 mL per 1.0 mmol substrate) under argon atmosphere. After irradiation for 2 h and 4 h, additional 3,4,5-trifluorophenyldiazonium chloride (**2a) (**0.3 M solution, 0.75 equiv.) was added. After a total irradiation time of 6 h adding 2 times diazonium solution **2a**, the reaction mixture was extracted with ethyl acetate (3 × 20 mL). The combined organic phases were concentrated to 1/10 of the original volume and 10 mL of methanol and maleic acid as internal standard were added. Yields were determined by ^{1}H NMR.

(*S*)-2-Acetamido-3-[2-(4-fluorophenyl)-1*H*-indol-3-yl]propanoic acid (3nb) and its by-product (*S*)-2-Acetamido-3-{2-[(4-fluorophenyl)diazenyl]-1*H*-indol-3-yl}propanoic acid (3nb')

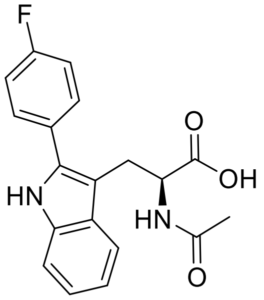

$C_{19}H_{17}FN_2O_3$
340.35 g/mol

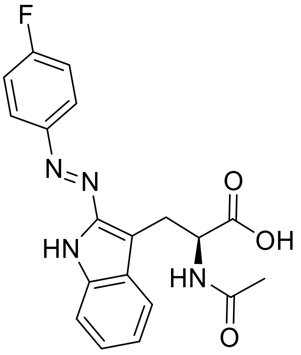

$C_{19}H_{17}FN_4O_3$
368.37 g/mol

Compounds **3nb** and **3nb'** were prepared according to **GP8**, using *N*-acetyl-*L* tryptophane (**1n**) **(**246 mg, 1.00 mmol, 1 equiv.) and **2b** (0.4 M solution, 1.1 equiv. and 2 × 0.75 equiv.). Analysis by standard ^{1}H NMR gave **3nb** in 8% and **3nb'** in 16% yield. Purification by preparative HPLC (system A, gradient: 38% → 63% acetonitrile in water, 0.1% formic acid) gave **3nb** as pink powder and **3nb'** as yellow powder.

3nb

***R*f** 0.24 (CH_2Cl_2/MeOH = 5:1) [UV].

***t*R** 7.57 min (system A, 38% → 63% acetonitrile in water, 0.1% formic acid).

^{1}H NMR (400 MHz, CD_3OD) δ (ppm) = 7.71 – 7.61 (m, 3H), 7.33 (dt, *J* = 8.1, 0.9 Hz, 1H), 7.25 – 7.18 (m, 2H), 7.10 (ddd, *J* = 8.1, 7.1, 1.2 Hz, 1H), 7.03 (ddd, *J* = 8.0, 7.0, 1.1 Hz, 1H), 4.69 (dd, *J* = 7.6, 6.0 Hz, 1H), 3.50 (dd, *J* = 14.6, 6.1 Hz, 1H), 3.28 (d, *J* = 7.7 Hz, 1H), 1.71 (s, 3H).

^{13}C NMR (101 MHz, CD_3OD) δ (ppm) = 175.3 (C_q), 172.8 (C_q), 163.7 (d, J_{CF} = 245.80, C_q), 137.6 (C_q), 136.3 (C_q), 131.4 (d, J_{CF} = 8.1 Hz, 2 x CH), 131.2 (d, J_{CF} = 3.2 Hz, C_q), 130.5 (C_q), 122.8 (CH), 120.1 (CH), 119.8 (CH), 116.5 (d, J_{CF} = 21.7, 2 x CH), 111.9 (CH), 108.4 (C_q), 54.9 (CH), 28.3 (CH_2), 22.4 (CH_3).

^{19}F NMR (377 MHz, CD_3OD) δ (ppm) = -116.7 (m, 1F).

HRMS (ESI) (*m/z*) calcd. for $C_{19}H_{17}FN_2O_3$ [M+H$^+$]: 341.1296, found: 341.1294.

$[\alpha]_D^{20}$ = + 4.3 (c = 0.25 in CH_3OH).

3nb'

***R*f** 0.33 (CH_2Cl_2/MeOH = 5:1) [UV].

***t*R** 10.17 min (system A, 38% → 63% acetonitrile in water, 0.1% formic acid).

^{1}H NMR (400 MHz, CD_3OD) δ 8.03 – 7.98 (m, 2H), 7.75 (dt, *J* = 8.1, 1.0 Hz, 1H), 7.37 (dt, *J* = 8.3, 1.0 Hz, 1H), 7.30 – 7.24 (m, 3H), 7.07 (ddd, *J* = 8.1, 7.0, 1.1 Hz, 1H), 4.86 (d, *J* = 7.1 Hz, 1H), 3.84 (dd, *J* = 14.0, 5.8 Hz, 1H), 3.62 (dd, *J* = 14.0, 7.0 Hz, 1H), 1.76 (s, 3H).

^{13}C NMR (101 MHz, CD_3OD) δ (ppm) = 173.3 (C_q), 171.5 (C_q), 163.9 (d, J_{CF} = 249.8 Hz, C_q), 149.85 (d, J_{CF} = 3.1 Hz, C_q), 146.6 (C_q), 136.4 (C_q), 128.0 (C_q), 126.0 (CH), 124.2 (d, J_{CF} = 8.7 Hz, 2 x CH), 120.2 (CH), 119.6 (CH), 118.0 (C_q), 115.6 (d, J_{CF} = 23.2 Hz, 2 x CH), 111.7 (CH), 53.5 (CH), 25.8 (CH_2), 21.1 (CH_3).

^{19}F NMR (377 MHz, CD_3OD) δ (ppm) = -112.7 (m, 1F).

HRMS (ESI) (*m/z*) calcd. for $C_{19}H_{17}FN_4O_3$ [M+H$^+$]: 369.1357, found: 369.1354.

$[\alpha]_D^{20}$ = + 11.2 (c = 0.50 in CH_3OH).

(*S*)-2-Acetamido-3-(4-hydroxyphenyl)-*N*-(2-{2-[(3,4,5-trifluorophenyl)diazenyl]-1*H*-indol-3-yl}ethyl)propanamide (3oa')

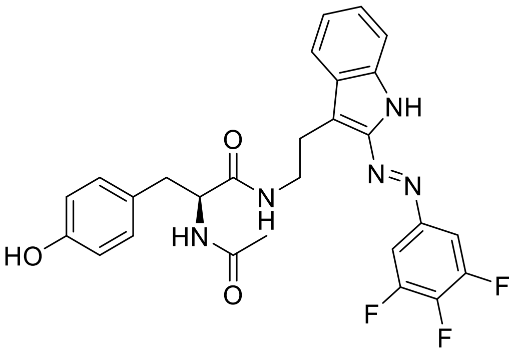

$C_{27}H_{24}F_3N_5O_3$
523.52 g/mol

Compound **3oa'** was prepared according to **GP8**, using **1l (**183 mg, 0.50 mmol, 1 equiv.) and **2a** (0.3 M solution, 1.1 equiv. and 2 × 0.75 equiv.). Analysis by standard ^{1}H NMR gave **3oa'** in 31%. Purification by preparative HPLC (system A, gradient: 52% → 77% acetonitrile in water, 0.1% formic acid) gave **3oa'** as yellow powder.

***R*f** 0.47 (CH_2Cl_2/MeOH = 10:1) [UV].

***t*R** 8.89 min (system A, 52% → 77% acetonitrile in water, 0.1% formic acid).

^{1}H NMR (400 MHz, CD_3OD) δ (ppm) = 7.75 (dt, *J* = 8.1, 1.0 Hz, 1H), 7.71 – 7.63 (m, 2H), 7.36 (dt, *J* = 8.3, 1.1 Hz, 1H), 7.32 (ddd, *J* = 8.3, 6.8, 1.0 Hz, 1H), 7.09 (ddd, *J* = 8.1, 6.8, 1.2 Hz, 1H), 6.96 – 6.87 (m, 2H), 6.68 – 6.60 (m, 2H), 4.38 (dd, *J* = 9.0, 5.9 Hz, 1H), 3.70 – 3.46 (m, 2H), 3.34 (t, *J* = 7.8 Hz, 2H), 2.82 (dd, *J* = 14.0, 6.0 Hz, 1H), 2.61 (dd, *J* = 13.9, 9.0 Hz, 1H), 1.82 (s, 3H).

^{13}C NMR (101 MHz, CD_3OD) δ (ppm) = 172.4 (C_q), 171.5 (C_q), 155.8 (C_q), 151.4 (ddd, J_{CF} = 249.1, 10.9, 4.0 Hz, 2 x C_q), 148.7 (td, J_{CF} = 6.7, 3.5 Hz, C_q), 145.9 (C_q), 140.3 (dt, J_{CF} = 254.0, 16.1 Hz, C_q), 137.2 (C_q), 129.7 (2 x CH) , 127.7 (C_q), 127.6 (C_q) , 127.1 (CH), 123.2 (C_q), 120.5 (CH), 119.8 (CH), 114.6 (2 x CH), 111.9 (CH), 106.24 (dd, J_{CF} = 18.4, 4.5 Hz, 2 x CH), 55.1 (CH), 40.1 (CH_2), 37.0 (CH_2), 23.6 (CH_2), 21.0 (CH_3).

^{19}F NMR (377 MHz, CD_3OD) δ (ppm) = -136.1 (m, 2F), -161.5 (m, 1F).

IR (KBr) $\tilde{\nu}$ (cm^{-1}): 3287 (m, br), 1644 (s), 1516 (s), 1458 (m), 1381 (m), 1230 (m), 1044 (m), 966 (w), 866 (w), 744 (m).

HRMS (ESI) (*m/z*) calcd. for $C_{27}H_{24}F_3N_5O_3$ [M+H$^+$]: 524.1904, found: 524.1906.

$[\boldsymbol{\alpha}]_{\boldsymbol{D}}^{\mathbf{20}}$ = + 25.1 (c = 0.50 in CH_3OH).

(*S*)-2-Acetamido-*N*-{2-[2-(4-fluorophenyl)-1*H*-indol-3-yl]ethyl}-3-(4-hydroxyphenyl)propanamide (3ob) and by-product (*S*)-2-acetamido-*N*-(2-{2-[(4-fluorophenyl)diazenyl]-1*H*-indol-3-yl}ethyl)-3-(4-hydroxyphenyl)propanamide (3ob')

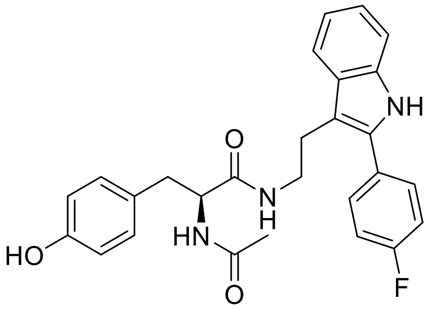

$C_{27}H_{26}FN_3O_3$
459.52 g/mol

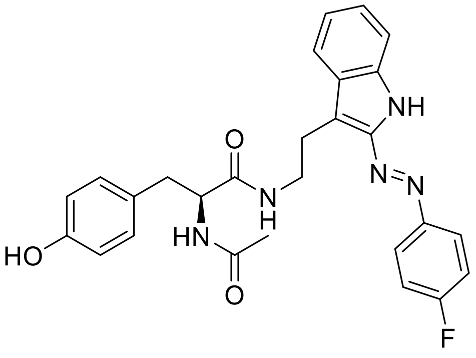

$C_{27}H_{26}FN_5O_3$
487.54 g/mol

Compounds **3ob** and **3ob'** were prepared according to **GP8**, using **1l** (183 mg, 0.50 mmol, 1 equiv.) and **2b** (0.4 M solution, 1.1 equiv. and 2 × 0.75 equiv.). Analysis by standard ^{1}H NMR gave **3ob** in 12% and **3ob'** in 40%. Purification by preparative HPLC (system A, gradient: 35% → 59% acetonitrile in water, 0.1% formic acid) gave **3ob** and **3ob'** as yellow powders.

3ob

***R*f** 0.48 (CH_2Cl_2/MeOH = 10:1) [UV].

***t*R** 7.44 min (system A, 35% → 59% acetonitrile in water, 0.1% formic acid).

^{1}H NMR (400 MHz, CD_3OD) δ (ppm) = 7.68 – 7.62 (m, 2H), 7.60 (dt, *J* = 7.8, 1.0 Hz, 1H), 7.35 (dt, *J* = 8.1, 0.9 Hz, 1H), 7.25 – 7.18 (m, 2H), 7.11 (ddd, *J* = 8.2, 7.0, 1.2 Hz, 1H), 7.06 – 6.99 (m, 3H), 6.71 – 6.66 (m, 2H), 4.43 (dd, *J* = 8.3, 6.7 Hz, 1H), 3.51 – 3.33 (m, 2H), 2.98 – 2.93 (m, 2H), 2.90 (dd, *J* = 13.9, 6.9 Hz, 1H), 2.73 (dd, *J* = 13.8, 8.3 Hz, 1H), 1.89 (s, 3H).

^{13}C NMR (101 MHz, CD_3OD) δ (ppm) = 173.6 (C_q), 173.0 (C_q), 163.6 (d, J_{CF} = 245.7 Hz, C_q), 157.3 (C_q), 137.8 (C_q), 135.3 (C_q), 131.3 (2 x CH), 131.0 (d, J_{CF} = 8.1 Hz, 2 x CH), 130.3 (C_q), 129.1 (C_q), 122.9 (CH), 120.1 (CH), 119.6 (CH), 116.6 (d, J_{CF} = 21.7 Hz, 2 x CH), 116.1 (2 x CH), 112.0 (CH), 109.9 (C_q), 56.7 (CH), 41.2 (CH_2), 38.4 (CH_2), 25.8 (CH_2), 22.4 (CH_3).One carbon signal missing.

^{19}F NMR (377 MHz, CD_3OD) δ (ppm) = -116.9 (m, 1F).

IR (KBr) $\tilde{\nu}$ (cm^{-1}): 3397 (m), 3294 (m, br), 3064 (m), 2930 (m), 1647 (s), 1515 (s), 1457 (m), 1372 (m), 1227 (s), 1157 (m), 1012 (w), 839 (m), 745 (m).

HRMS (ESI) (*m/z*) calcd. for $C_{27}H_{26}FN_3O_3$ [M+H$^+$]: 460.2031, found: 460.2032.

$[\alpha]_D^{20}$ = + 12.2 (c = 0.25 in CH_3OH).

3ob'

***R*f** 0.41 (CH_2Cl_2/MeOH = 10:1) [UV].

***t*R** 8.96 min (system A, 35% → 59% acetonitrile in water, 0.1% formic acid).

^{1}H NMR (600 MHz, CD_3OD) δ (ppm) = 8.01 – 7.94 (m, 2H), 7.74 (dt, *J* = 8.0, 1.0 Hz, 1H), 7.38 (dt, *J* = 8.1, 0.9 Hz, 1H), 7.29 (ddd, *J* = 8.3, 7.0, 1.2 Hz, 1H), 7.29 – 7.22 (m, 2H), 7.09 (ddd, *J* = 8.0, 7.0, 1.0 Hz, 1H), 6.95 – 6.89 (m, 2H), 6.67 – 6.62 (m, 2H), 4.40 (dd, *J* = 8.8, 6.1 Hz, 1H), 3.69 – 3.61 (m, 1H), 3.57 – 3.49 (m, 1H), 3.38 – 3.32 (m, 2H), 2.84 (dd, *J* = 14.0, 6.0 Hz, 1H), 2.66 – 2.63 (m, 1H), 1.82 (s, 3H).

^{13}C NMR (151 MHz, CD_3OD) δ (ppm) = 172.3 (C_q), 171.5 (C_q), 163.8 (d, J_{CF} = 249.9 Hz, C_q), 155.8 (C_q), 149.9 (d, J_{CF} = 3.1 Hz, C_q), 146.1 (C_q), 136.6 (C_q), 129.8 (2 x CH), 127.8 (C_q), 127.7 (C_q), 126.2 (CH), 124.1 (d, J_{CF} = 8.8 Hz, 2 x CH), 120.4 (C_q), 120.2 (CH), 119.6 (CH), 115.6 (d, J_{CF} = 23.2 Hz, 2 x CH), 114.7 (2 x CH), 111.8 (CH), 55.1 (CH), 40.2 (CH_2), 37.0 (CH_2), 23.5 (CH_2), 21.0 (CH_3).

^{19}F NMR (377 MHz, CD_3OD) δ (ppm) = -112.9 (m, 1F).

IR (KBr) $\tilde{\nu}$ (cm^{-1}): 3439 (w), 3287 (m), 3059 (w), 2928 (w), 1651 (s), 1606 (m), 1592 (m), 1550 (m), 1512 (m), 1496 (m), 1383 (m), 1226 (m), 1153 (w), 1075 (w), 843 (w), 739 (w).

HRMS (ESI) (*m/z*) calcd. for $C_{27}H_{26}FN_5O_3$ [M+H$^+$]: 488.2092, found: 488.2091.

$[\alpha]_D^{20}$ = + 18.9 (c = 0.50 in CH_3OH).

Further experiments for the synthesis of **3ma** and **3mb** were carried out under similar conditions, but yielded only traces of arylation products and no significant amounts of side products.

5. Additional experiments on mechanistic background

5.1 Analysis of CT complex formation

UV-Vis measurements were performed to investigate the CT complex according to a procedure from literature.[1] For all UV-Vis measurements a mixture of water and 2 M HCl (1/1) was used as solvent.

Table S2. CT complex measurement with **1b** and **2a’**

measurement	H-Gly-Tyr-OH (**1b**)	3,4,5-trifluorophenyldiazonium tetrafluoroborate (**2a’**)	solvent
blank	--	--	2 mL
**1b**	0.4 mmol	--	2 mL
**2a**		0.4 mmol	2 mL
complex	0.4 mmol	0.4 mmol	2 mL

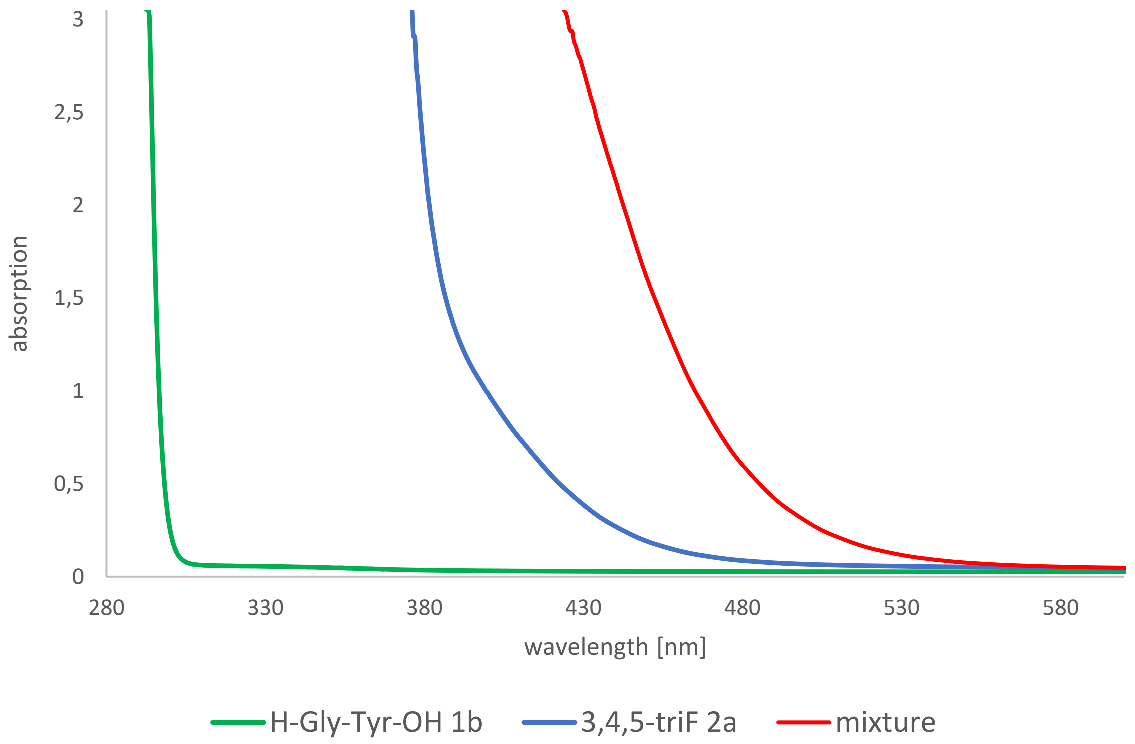

Figure S8. UV-Vis spectra of **1b**, **2a’** and the reaction mixture.

Table S3. CT complex measurement with **1b** and **2b'**

measurement	H-Gly-Tyr-OH (**1b**)	4-fluorophenyldiazonium tetrafluoroborate (**2b'**)	solvent
blank	--	--	2 mL
**1b**	0.4 mmol	--	2 mL
**2b**	--	0.4 mmol	2 mL
complex	0.4 mmol	0.4 mmol	2 mL

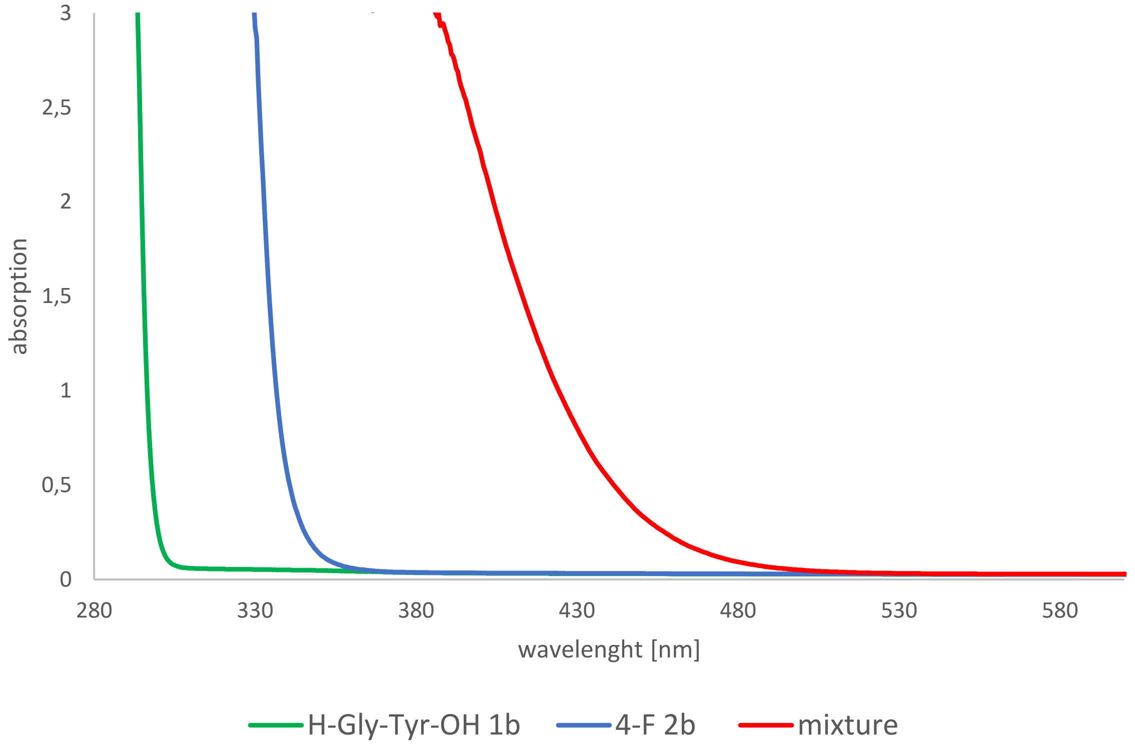

Figure S9. UV-Vis spectra of **1b**, **2b'** and the reaction mixture.

a.

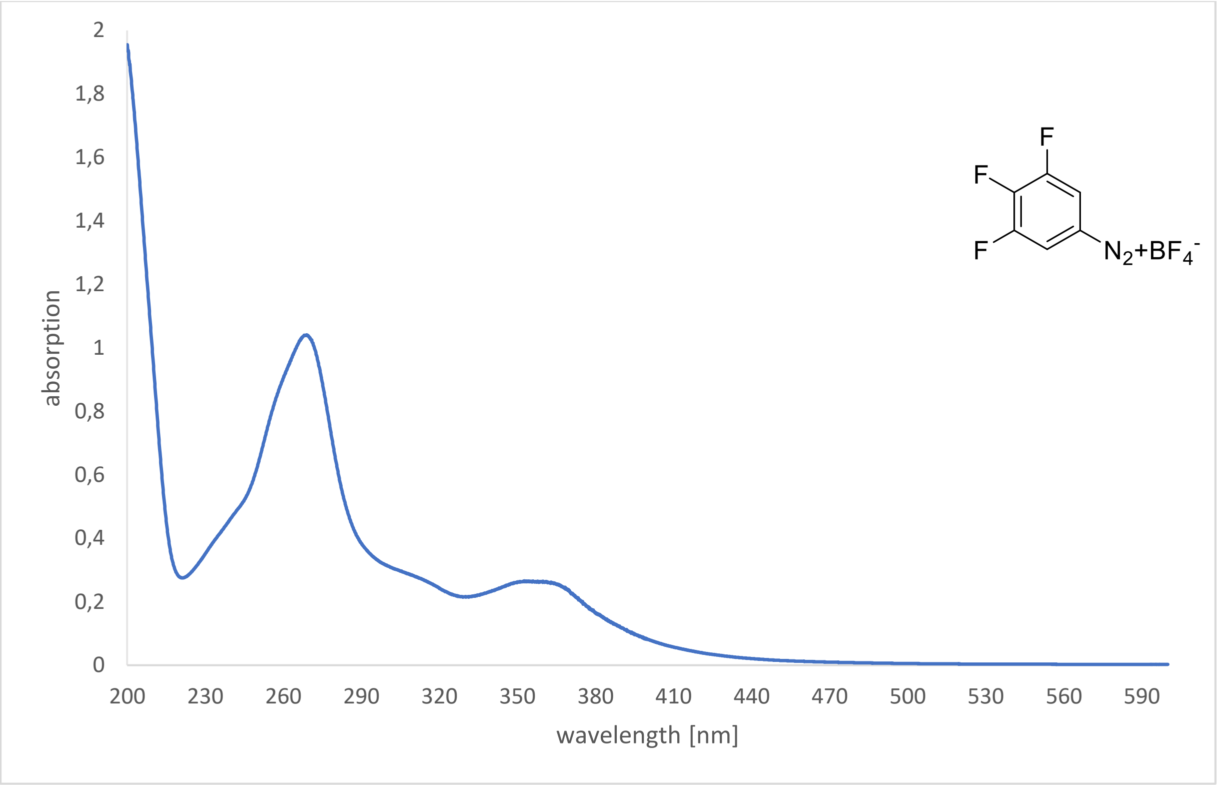

b.

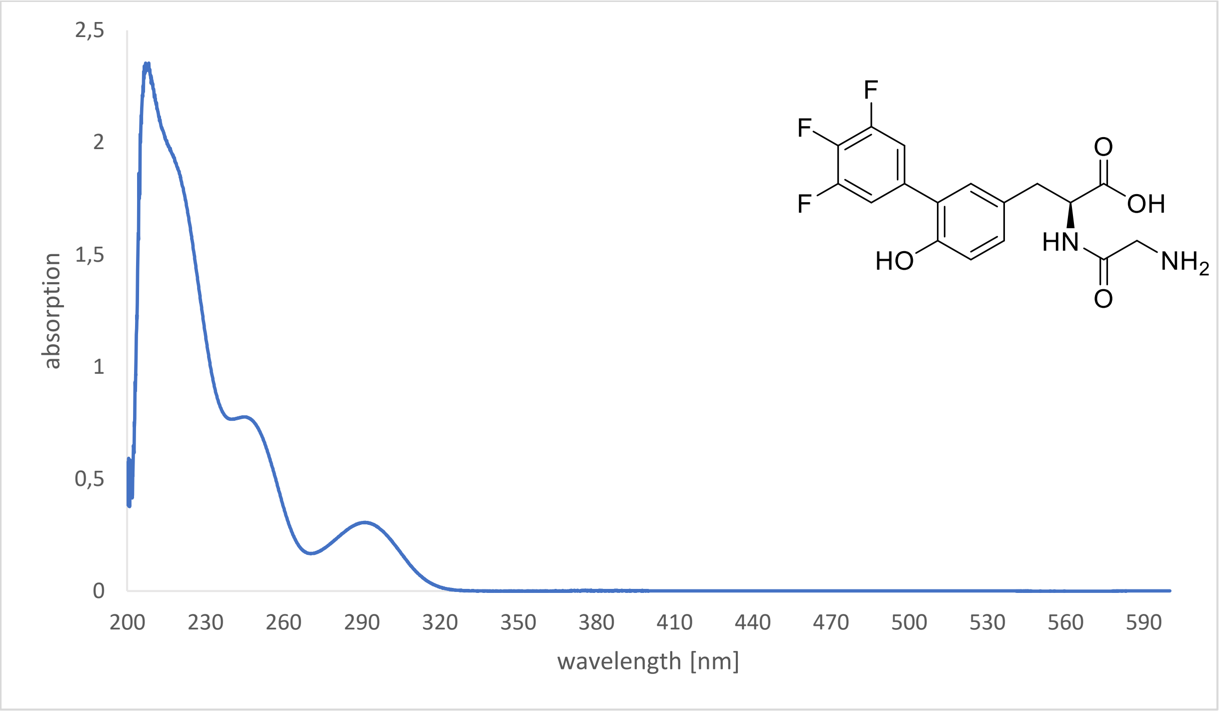

Figure S10. a. UV-Vis spectrum of **2a'** (c = 0.125 µmol/mL, CH_3CN) **b.** UV-Vis spectrum of **3ba** (c = 0.10 µmol/mL, 1 M HCl in water).

5.2 Job plot measurements

For job plot measurements a mixture of water and 2 M HCl (1/1) was used as solvent.

Table S4. Amounts of **1b** and **2a'** or **2b'** used for job plot measurements.

ratio	H-Gly-Tyr-OH (**1b**)	Diazonium tetrafluoroborate	solvent
blank	--	--	1 mL
10:0	0.40 mmol	--	1 mL
9:1	0.36 mmol	0.04 mmol	1 mL
8:2	0.32 mmol	0.08 mmol	1 mL
7:3	0.28 mmol	0.12 mmol	1 mL
6:4	0.24 mmol	0.16 mmol	1 mL
5:5	0.20 mmol	0.20 mmol	1 mL
4:6	0.16 mmol	0.24 mmol	1 mL
3:7	0.12 mmol	0.28 mmol	1 mL
2:8	0.08 mmol	0.32 mmol	1 mL
1:9	0.04 mmol	0.36 mmol	1 mL
0:10	--	0.40 mmol	1 mL

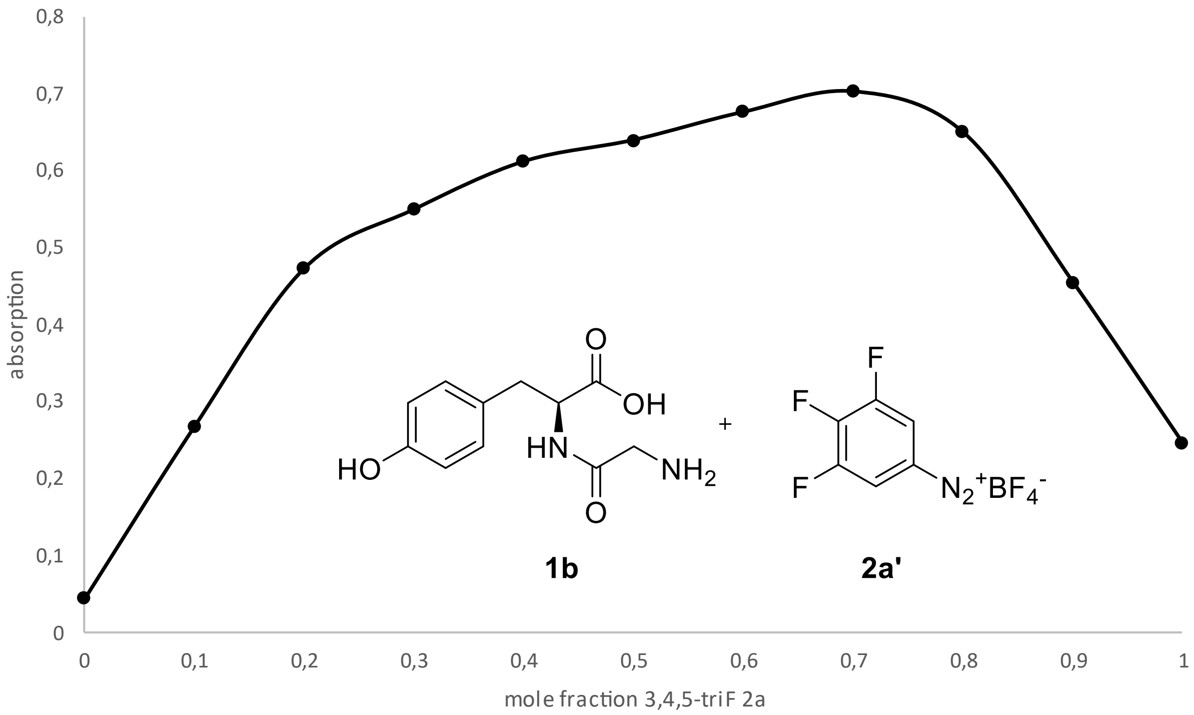

Figure S11. Job plot of **1b** and **2a'**.

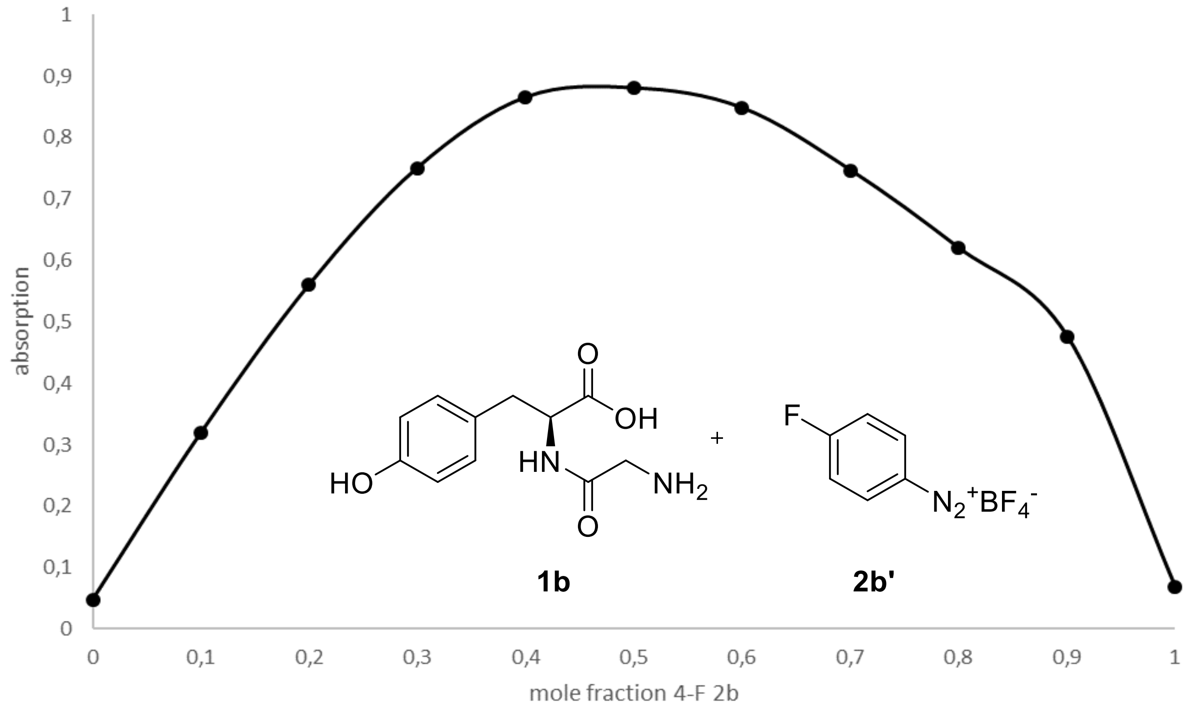

Figure S12. Job plot of **1b** and **2b’**.

5.3 TLC experiments with different diazonium salts

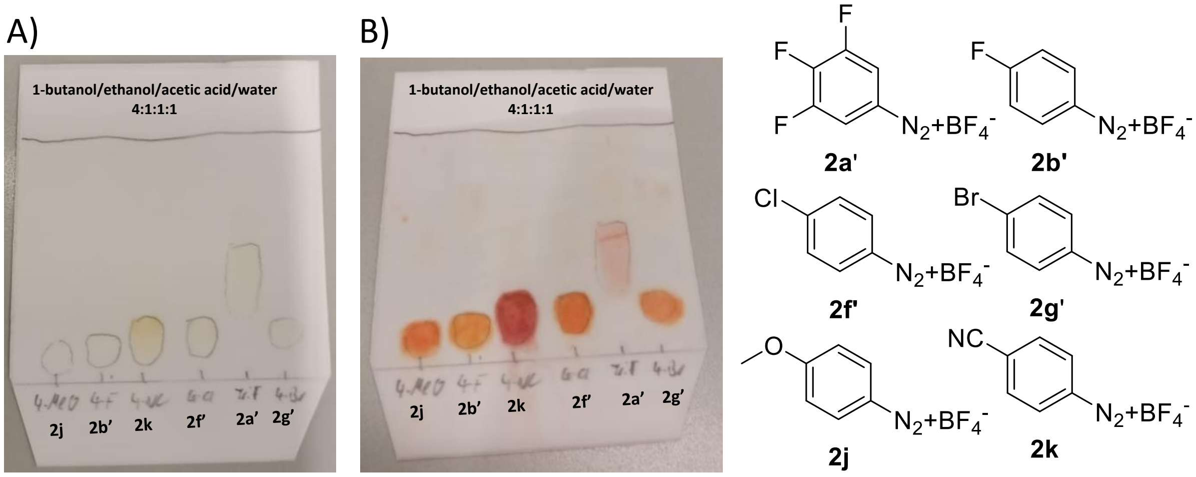

Figure S13. TLC with 6 different phenyldiazonium tetrafluoroborate salts using 1-butanol/ethanol/acetic acid/water (4/1/1/1) as mobile phase. A: TLC was analyzed under UV light. B: TLC was colored with ß-naphthol and sodium hydroxide solution.

R_f values for different phenyldiazonium salts using 1-butanol/ethanol/acetic acid/water (4/1/1/1) as mobile phase.

***R*f (**4-methoxyphenyldiazonium tetrafluoroborate (**2j**)) 0.11 [UV].

***R*f (**4-fluorophenyldiazonium tetrafluoroborate (**2b'**)) 0.14 [UV].

***R*f (**4-cyanophenyldiazonium tetrafluoroborate (**2k**)) 0.17 [UV].

***R*f (**4-chlorophenyldiazonium tetrafluoroborate (**2f'**)) 0.17 [UV].

***R*f (**3,4,5-trifluorophenyldiazonium tetrafluoroborate (**2a'**)) 0.44 [UV].

***R*f (**4-bromophenyldiazonium tetrafluoroborate (**2g'**)) 0.17 [UV].

5.4 Competition experiment with nitrobenzene

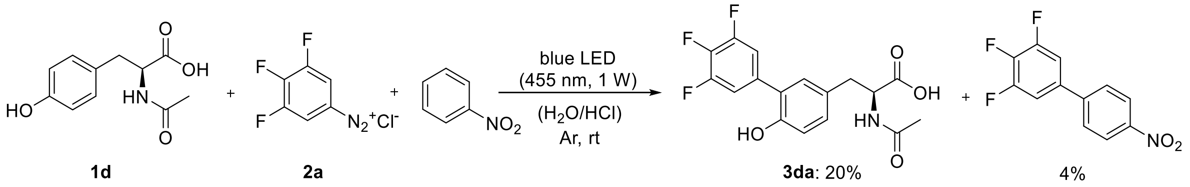

Scheme S1. Competition experiment with **1d**, **2a** and nitrobenzene.

N-acetyl-*L*-tyrosine (**1d**) **(**223 mg, 1.00 mmol, 1 equiv.) and nitrobenzene (1.00 mmol, 103 µL, 1 equiv.) were dissolved in 0.5 mL of 2 M HCl and 2.5 mL of acetonitrile and **2a** (2.5 mL of a 0.3 M solution, 0.75 mmol, 0.75 equiv.) was added under argon atmosphere. After irradiation

for 2 h under stirring the reaction mixture was extracted with ethyl acetate (3 × 20 mL). The combined organic phases were concentrated to 1/10 of the original volume and 10 mL of methanol and maleic acid as standard were added. Analysis of an aliquot by ^{1}H NMR spectroscopy gave **3da** in 20% and 3,4,5-trifluoro-4'-nitro-1,1'-biphenyl in 4% yield.

Preparation of reference compound 3,4,5-trifluoro-4'-nitro-1,1'-biphenyl

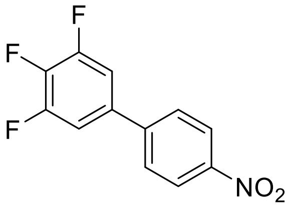

$C_{12}H_6F_3NO_2$
253.18 g/mol

Nitrobenzene (1.00 mmol, 103 µL, 1 equiv.) was dissolved in 0.5 mL of 2 M HCl and 2.5 mL of acetonitrile under argon atmosphere and **2a** (3.8 mL of a 0.3 M solution, 1.1 mmol, 1.1 equiv.) was added. After 2 h and 4 h of irradiation, additional **2a** (2.5 mL of 0.3 M solution, 0.75 mmol, 0.75 equiv.) was added. After a total irradiation time of 6 h, the reaction mixture was extracted with ethyl acetate (3 × 20 mL) and the solvent of the combined organic phases were removed under reduced pressure. Purification with column chromatography (*n*-hexane) produced pure 3,4,5-trifluoro-4'-nitro-1,1'-biphenyl along with minor amounts of other isomers.

***R*f** 0.5 (hexane/ethyl acetate = 24:1) [UV].

^{1}H NMR (400 MHz, CD_3OD) δ (ppm) = 8.37 – 8.31 (m, 2H), 7.92 – 7.86 (m, 2H), 7.63 – 7.53 (m, 2H).

^{13}C NMR (101 MHz, CD_3OD) δ (ppm) = 152.8 (ddd, J_{CF} = 248.8, 10.0, 4.0 Hz, 2 × C_q), 149.2 (C_q), 145.3 (C_q), 141.3 (dt, J_{CF} = 252.2, 15.5 Hz, C_q), 136.7 (td, J_{CF} = 8.1, 4.9 Hz, C_q), 129.2 (2 × CH), 125.2 (2× CH), 113.4 – 112.8 (m, 2 × CH).

HRMS (ESI) (*m/z*) calcd. for $C_{12}H_7F_3NO_2$ [M+H$^+$]: 254.0423, found: 254.0423.

5.5 Differential pulse voltammetry

Differential pulse voltammetry was performed in a classical three-electrode cell from Deutsche Metrohm GmbH & Co. KG with Metrohm Autolab pGSTAT 101 and controlled by NOVA 2.1 software. As working electrode, a gold electrode was used, combined with a platinum sheet (1.0 cm^2) as counter electrode. All potentials were measured relative to an Ag/AgCl (2 M lithium chloride in ethanol) reference electrode with a potential of 0.164 V vs. SHE at 21 ± 1 °C. The diazonium salt **2a'** or **2b'** (1 mM) were dissolved in acetonitrile (LC-MS grade) with NBu_4PF_6 (0.1 M) under nitrogen atmosphere.

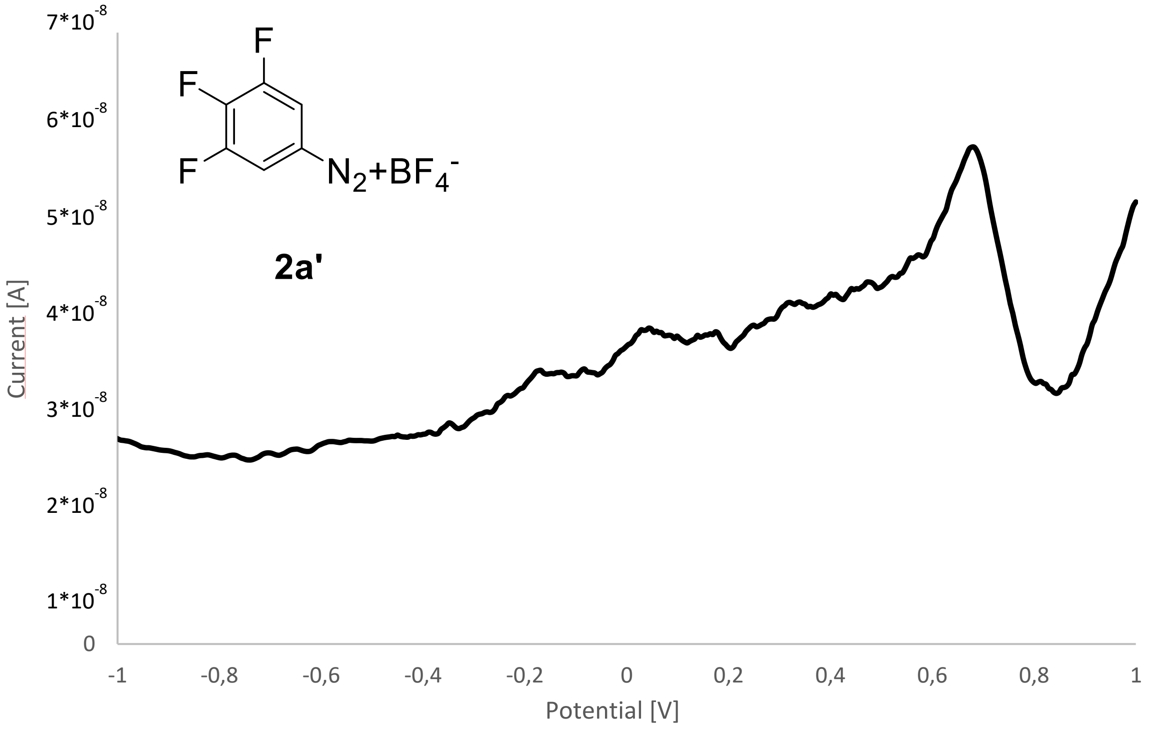

Figure S14. Differential pulse voltammetry of **2a'**.

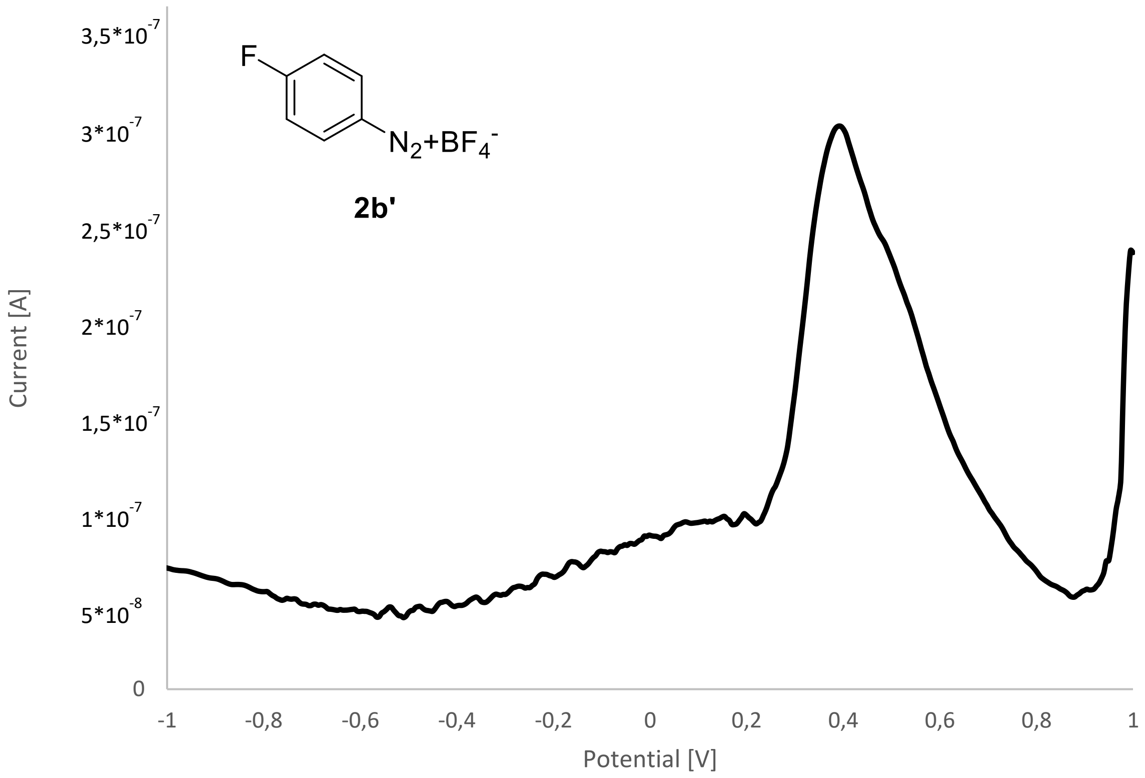

Figure S15. Differential pulse voltammetry of **2b'**.

6. Functionalization by nucleophilic substitution

Reaction of **5** with **6** (Scheme 4A in the manuscript)

Acetyl-*L*-cysteine (**5**) (163 mg, 1.00 mmol, 1.0 equiv.), potassium carbonate (276 mg, 2.00 mmol, 2.0 equiv.) and 1,2,3-trifluorobenzene (**6**) (1.32 g, 10 mmol, 1.0 ml, 10 equiv.) were dissolved in dimethyl sulfoxide (5 mL) under argon. After stirring the suspension for four days at room temperature, the mixture was acidified with 1 M HCl to pH = 1-3 and extracted with ethyl acetate (3 × 70 mL). The organic phase was analyzed by ^{1}H NMR, giving a ratio of product **7a**, product **7b** and acetyl-*L*-cysteine of 1:0.5:0.4. The crude mixture was purified by preparative HPLC (system A, gradient: 19% → 26% acetonitrile in water, 0.1% formic acid) to give **7a** and **7b** as white powder. The synthetic procedure was derived from literature.[10]

N-Acetyl-*S*-(2,6-difluorophenyl)-*L*-cysteine (7a)

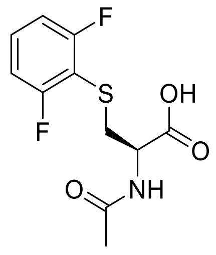

$C_{11}H_{11}F_2NO_3S$
275.27 g/mol

***R*f**	0.5 (CH_2Cl_2/MeOH = 9:1 +0.1% formic acid) [UV].
***t*R**	9.68 min (system A, 19% → 26% acetonitrile in water, 0.1% formic acid).
**^{1}H NMR**	(400 MHz, $(CD_3)_2SO$) δ (ppm) = 8.25 (d, *J* = 7.9 Hz, 1H), 7.46 (tt, *J* = 8.1, 6.5 Hz, 1H), 7.21 – 7.12 (m, 2H), 4.28 – 4.16 (m, 1H), 3.06 (dd, *J* = 13.5, 8.8 Hz, 1H), 1.77 (s, 3H). One aliphatic H is covered by the HDO signal.
**^{13}C NMR**	(101 MHz, $(CD_3)_2SO$) δ (ppm) = 171.7 (C_q), 169.3 (C_q), 162.6 (dd, J_{CF} = 246.3, 4.9 Hz, 2 × C_q), 131.2 (t, J_{CF} = 10.4 Hz, CH), 112.6 – 111.8 (m, 2 × CH), 109.5 (t, J_{CF} = 22.2 Hz, C_q), 52.3 (CH), 35.1 (CH_2), 22.3 (CH_3).
**^{19}F NMR**	(376 MHz, $(CD_3)_2SO$) δ (ppm) = -104.6 (m, 2F).

IR (KBr) $\tilde{\nu}$ (cm^{-1}): 3371 (m), 3083 (w), 2888 (w), 2500 (m, br), 1731 (s), 1616 (s), 1527 (s), 1462 (s), 1376 (m), 1350 (m), 1313 (m), 1275 (m), 1237 (s), 1218 (s), 1140 (w), 990 (s), 952 (m), 839 (s), 793 (m), 586 (m).

HRMS (ESI) (*m/z*) calcd. for $C_{11}H_{12}F_2NO_3S$ [$M+H^+$]: 276.0501, found: 276.0501.

$[\alpha]_D^{20}$ = + 1.2 (c = 1.00 in CH_3OH).

N-Acetyl-*S*-(2,6-difluorophenyl)-*L*-cysteine (7b)

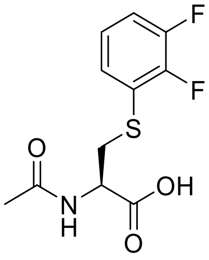

$C_{11}H_{11}F_2NO_3S$
275.27 g/mol

***R*f** 0.5 (CH_2Cl_2/MeOH = 9:1 +0.1% formic acid) [UV].

***t*R** 12.06 min (system A, 19% → 26% acetonitrile in water, 0.1% formic acid).

^{1}H NMR (400 MHz, $(CD_3)_2SO$) δ (ppm) = 8.35 (d, *J* = 8.0 Hz, 1H), 7.38 – 7.26 (m, 2H), 7.26 – 7.17 (m, 1H), 4.44 – 4.32 (m, 1H), 3.19 (dd, *J* = 13.7, 8.5 Hz, 1H), 1.80 (s, 3H). One aliphatic H is covered by the HDO signal.

^{13}C NMR (101 MHz, $(CD_3)_2SO$) δ (ppm) = 171.6 (C_q), 169.4 (C_q), 149.8 (dd, J_{CF} = 247.2, 13.1 Hz, C_q), 148.1 (dd, J_{CF} = 243.5, 13.4 Hz, C_q), 126.1 (d, J_{CF} = 3.3 Hz, CH), 125.2 (dd, J_{CF} = 7.5, 4.6 Hz, CH), 125.0 (d, J_{CF} = 13.7 Hz, C_q), 115.8 (d, J_{CF} = 17.1 Hz, CH), 51.6 (CH), 33.8 (CH_2), 22.3 (CH_3).

^{19}F NMR (376 MHz, $(CD_3)_2SO$) δ (ppm) = -135.5 (m, 1F), -137.5 (m, 1F).

HRMS (ESI) (*m/z*) calcd. for $C_{11}H_{12}F_2NO_3S$ [$M+H^+$]: 276.0501, found: 276.0501.

(*S*)-{5'-[(*R*)-2-Acetamido-2-carboxyethyl]-3,5-difluoro-2'-hydroxy-[1,1'-biphenyl]-4-yl}-*N*-acetyl-*L*-cysteine (8)

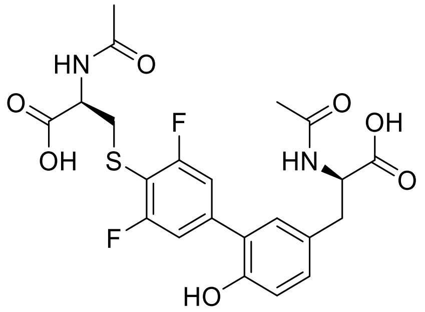

$C_{22}H_{22}F_2N_2O_7S$
496.48 g/mol

N-Acetyl-*L*-cysteine (**5**) (1.63 g, 10 mmol, 10 equiv.), potassium carbonate (1.93 g, 14 mmol, 14 equiv.) and **3da** (353 mg, 1.0 mmol, 1.0 equiv.) were dissolved in dimethyl sulfoxide (15 mL) under argon. After stirring the suspension for five days at room temperature, the mixture was acidified with 1 M HCl to pH = 1-3 and extracted with ethyl acetate (3 × 70 mL). The organic phase was analyzed by ^{1}H NMR, giving a ratio of product **8** and *N*-acetyl-*L*-cysteine of 1:0.2. The crude mixture was purified by preparative HPLC (system A, gradient: 15% → 30% acetonitrile in water, 0.1% formic acid) to give **8** as white powder.

***t*R** 12.12 min (system A, 15% → 30% acetonitrile in water, 0.1% formic acid).

^{1}H NMR (400 MHz, $(CD_3)_2SO$) δ (ppm) = 9.85 (s, 1H), 8.26 (d, *J* = 7.8 Hz, 1H), 8.17 (d, *J* = 8.0 Hz, 1H), 7.44 – 7.34 (m, 2H), 7.25 (d, *J* = 2.2 Hz, 1H), 7.07 (dd, *J* = 8.3, 2.2 Hz, 1H), 6.88 (d, *J* = 8.3 Hz, 1H), 4.38 (ddd, *J* = 9.4, 8.0, 5.0 Hz, 1H), 4.26 (td, *J* = 8.3, 4.8 Hz, 1H), 3.31 (dd, *J* = 13.8, 5.2 Hz, 1H), 3.09 (dd, *J* = 13.5, 8.8 Hz, 1H), 2.98 (dd, *J* = 13.8, 5.0 Hz, 1H), 2.77 (dd, *J* = 13.8, 9.4 Hz, 1H), 1.80 (s, 3H), 1.79 (s, 3H). Two carboxylic acid protons are missing.

^{13}C NMR (101 MHz, $(CD_3)_2SO$) δ (ppm) = 173.3 (C_q), 171.7 (C_q), 169.31 (C_q), 169.25 (C_q), 162.1 (dd, J_{CF} = 244.4, 6.0 Hz, C_q), 153.1 (C_q), 141.5 (t, J_{CF} = 10.4 Hz, C_q), 130.9 (CH), 130.5 (CH), 128.6 (C_q), 123.8 (C_q), 116.3 (CH), 112.5 – 111.7 (m, 2 × CH), 107.2 (t, J_{CF} = 22.6 Hz, C_q), 53.7 (CH), 52.4 (CH), 36.0 (CH_2), 35.3 (CH_2), 22.4 (CH_3), 22.3 (CH_3).

^{19}F NMR (376 MHz, $(CD_3)_2SO$) δ (ppm) = -105.8 (m, 1F).

HRMS (ESI) (*m/z*) calcd. for $C_{22}H_{23}F_2N_2O_7S$ [M+H$^+$]: 497.1189, found: 497.1187.

7. ^{1}H NMR and DEPTQ spectra of synthesized compounds

1f (Ac-Tyr-Phe-OH)

^{1}H NMR in CD_3OD, 400 MHz

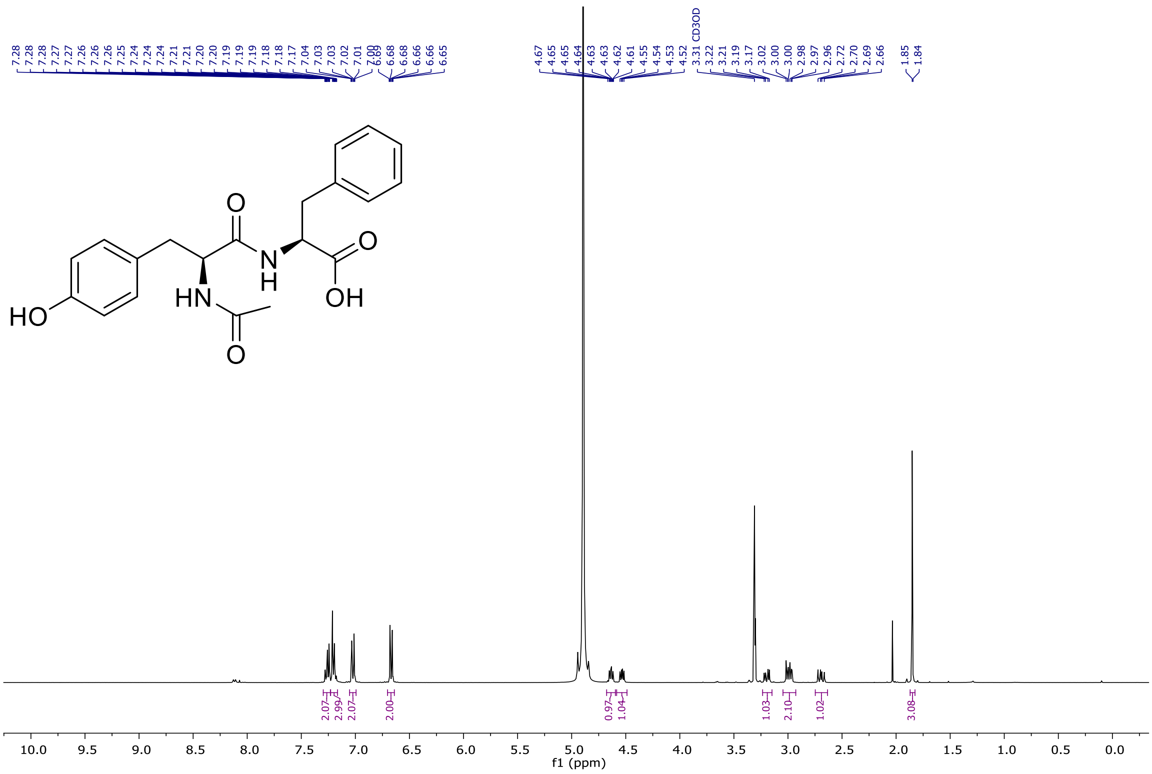

^{13}C NMR in CD_3OD, 101 MHz

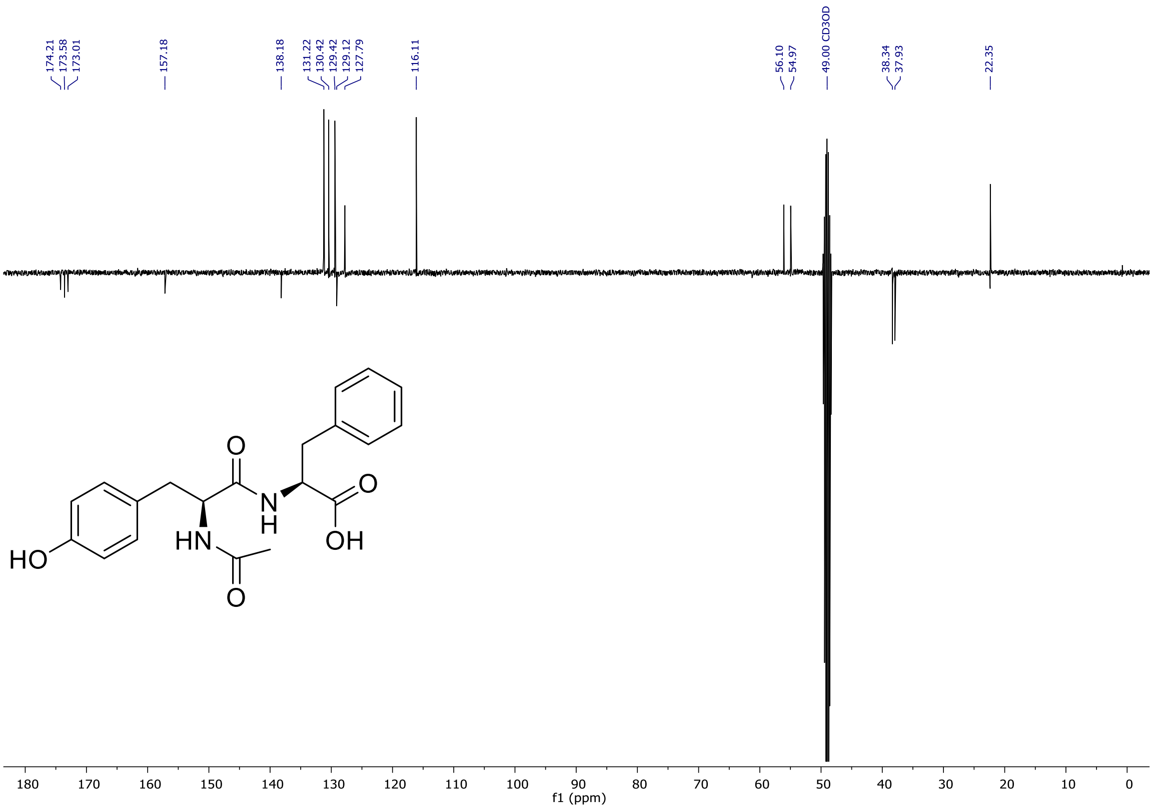

1g (Ac-Tyr-Gly-Me)

^{1}H NMR in CD_3OD, 400 MHz

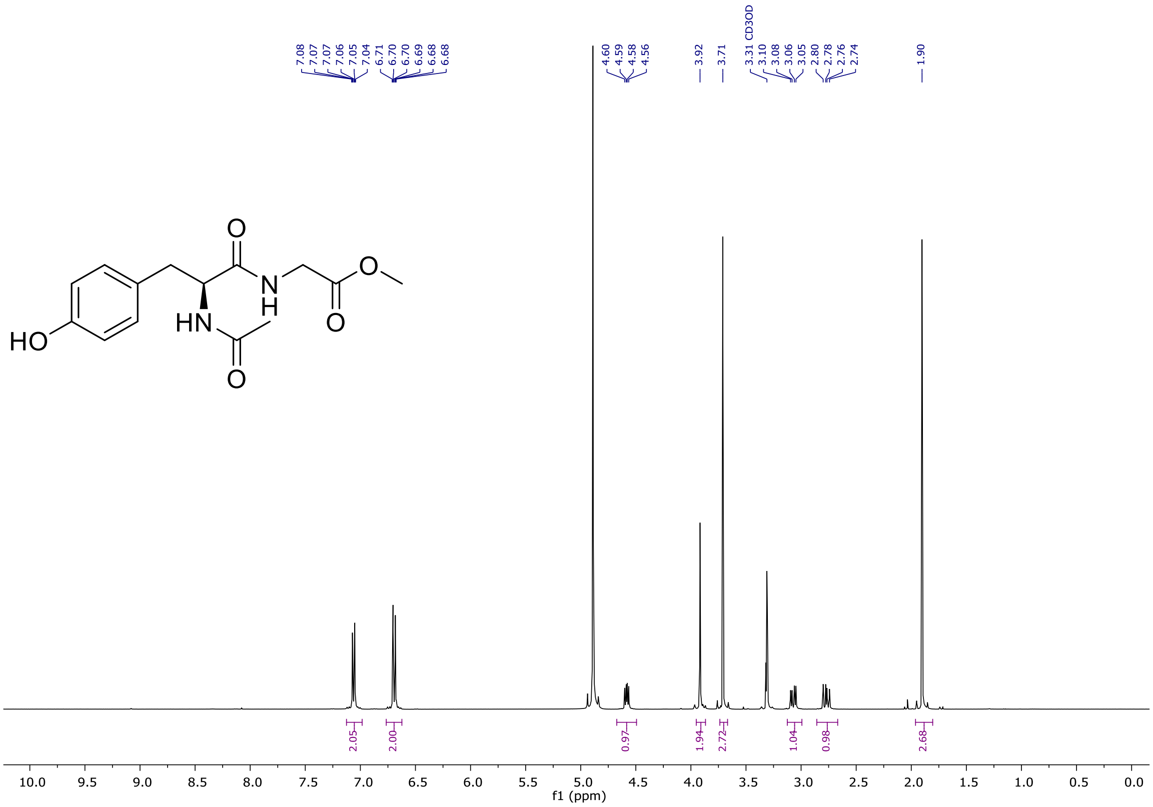

^{13}C NMR in CD_3OD, 101 MHz

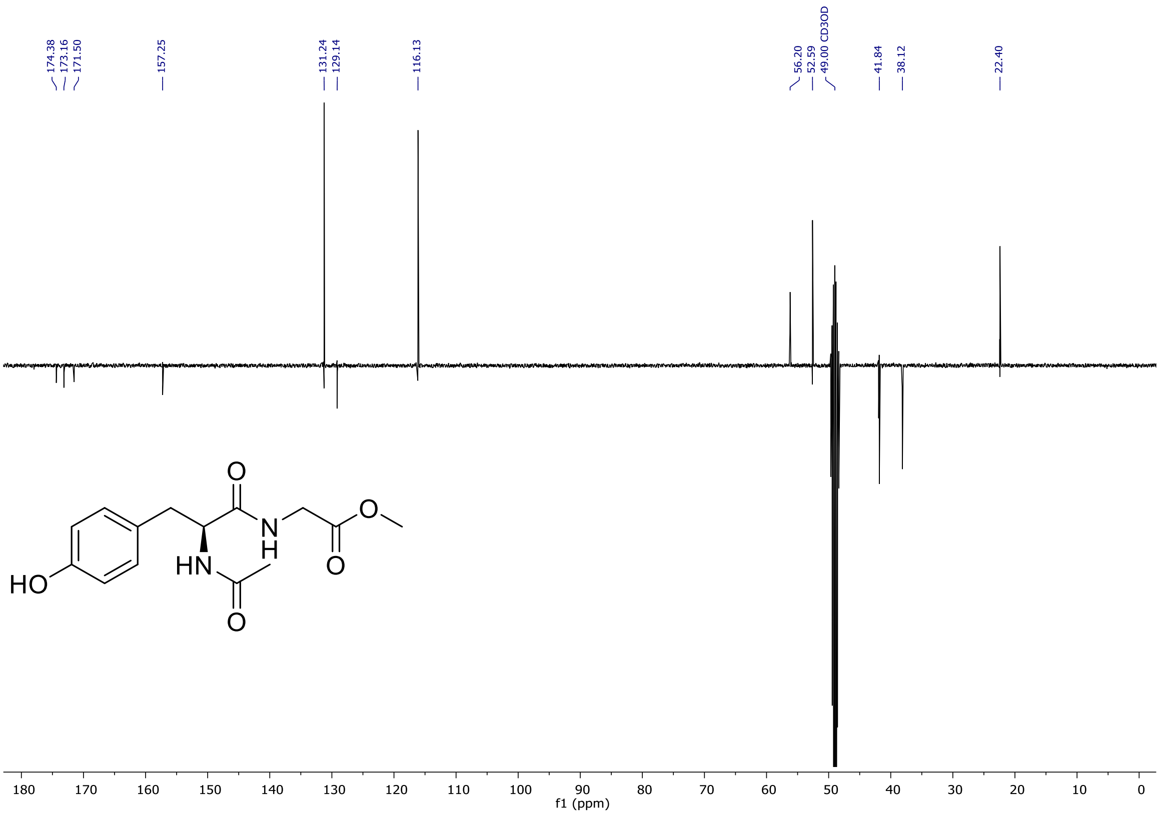

1i (Bz-Gly-Tyr-OH)

^{1}H NMR in CD_3OD, 400 MHz

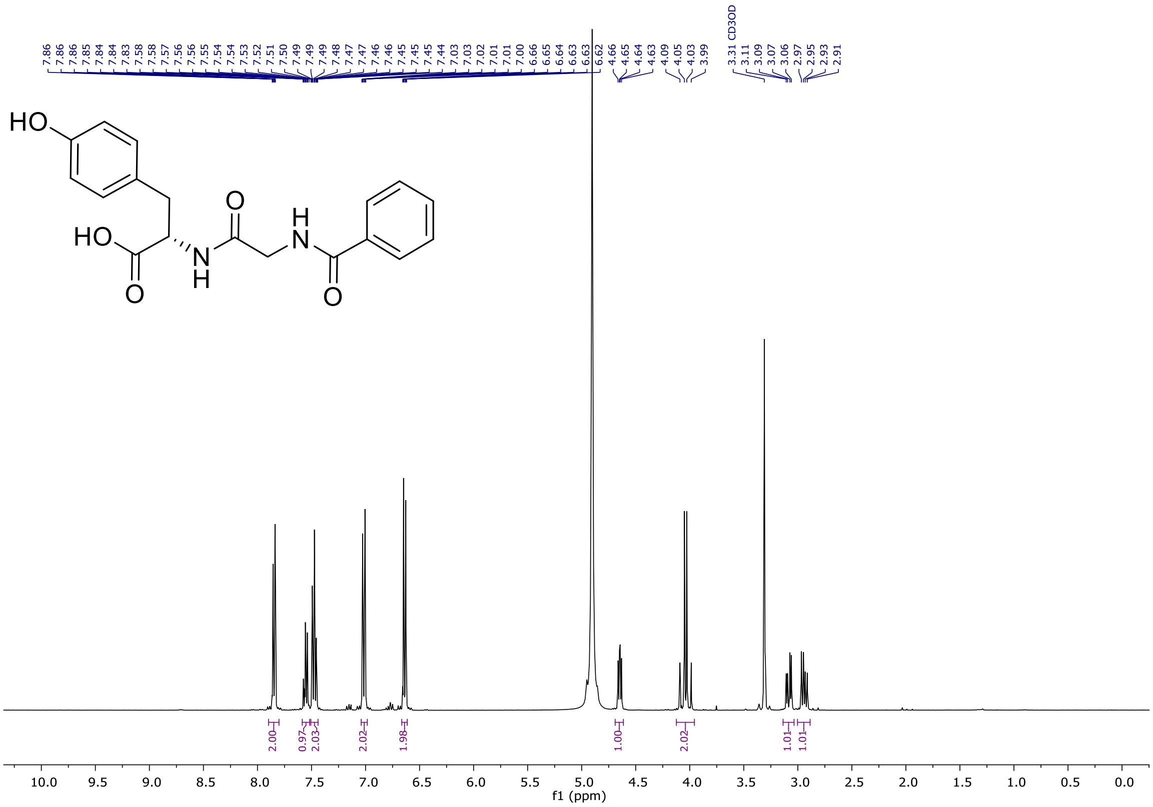

^{13}C NMR in CD_3OD, 101 MHz

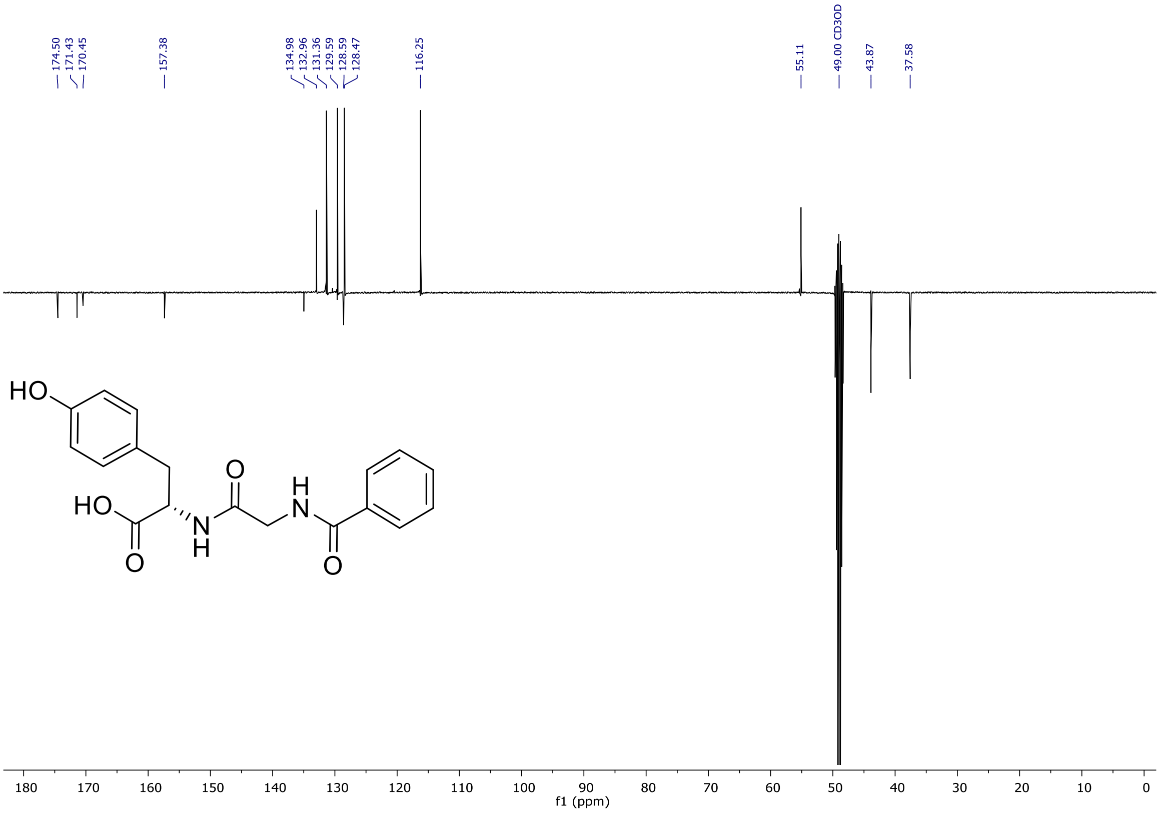

1j (NT(8-13))

^{1}H NMR in CD_3OD, 600 MHz

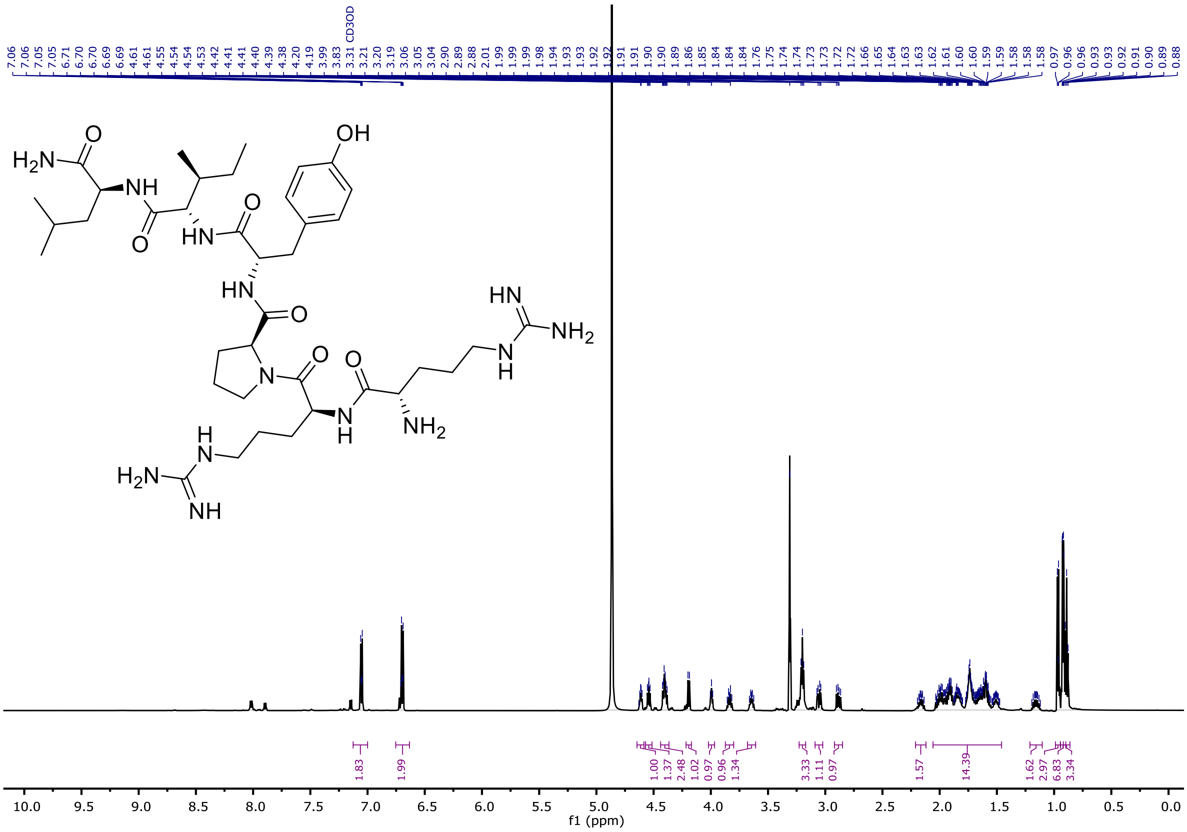

^{13}C NMR in CD_3OD, 101 MHz

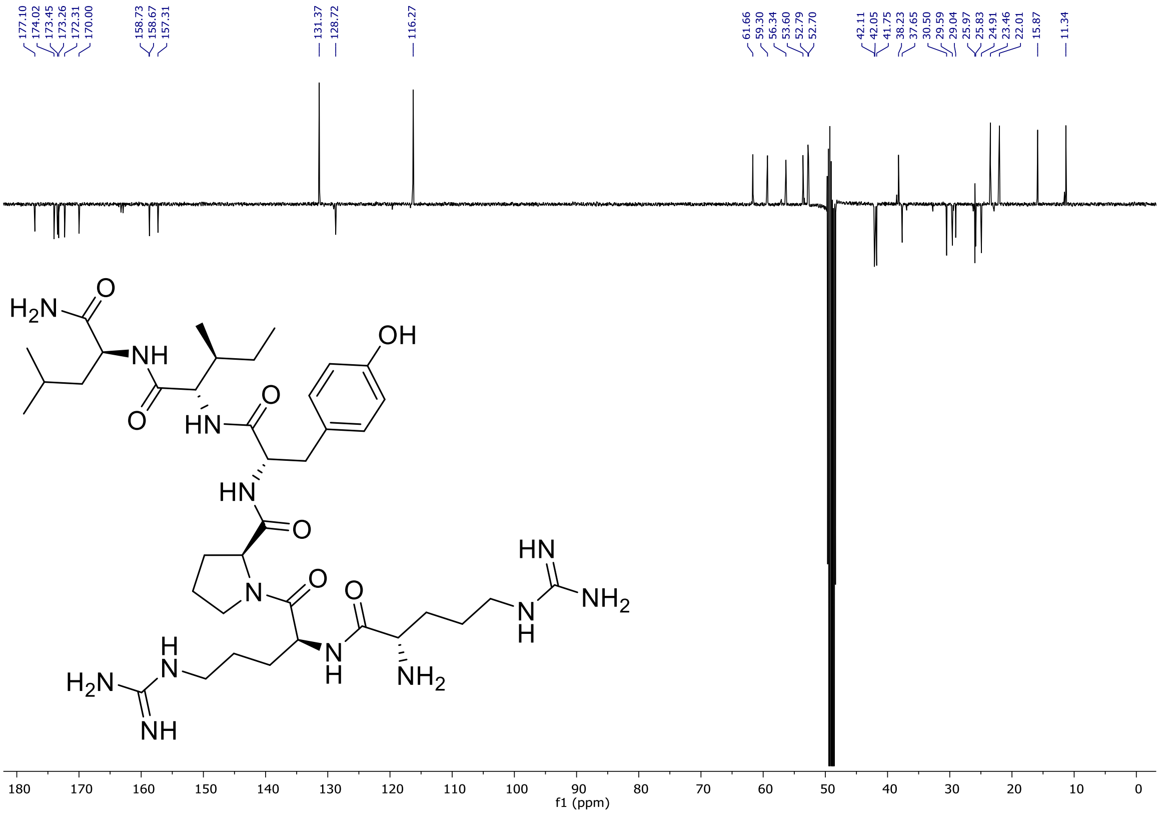

1k (Leu-enkephalin)

^{1}H NMR in CD_3OD, 400 MHz

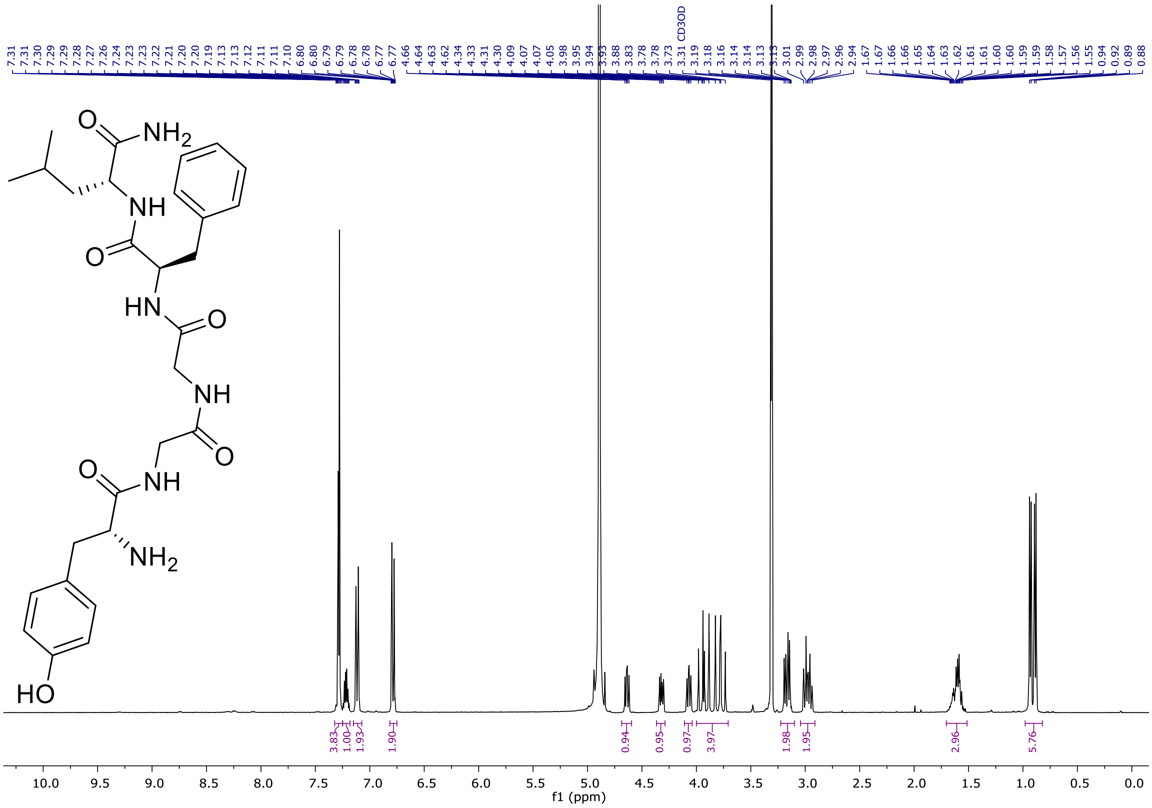

^{13}C NMR in CD_3OD, 101 MHz

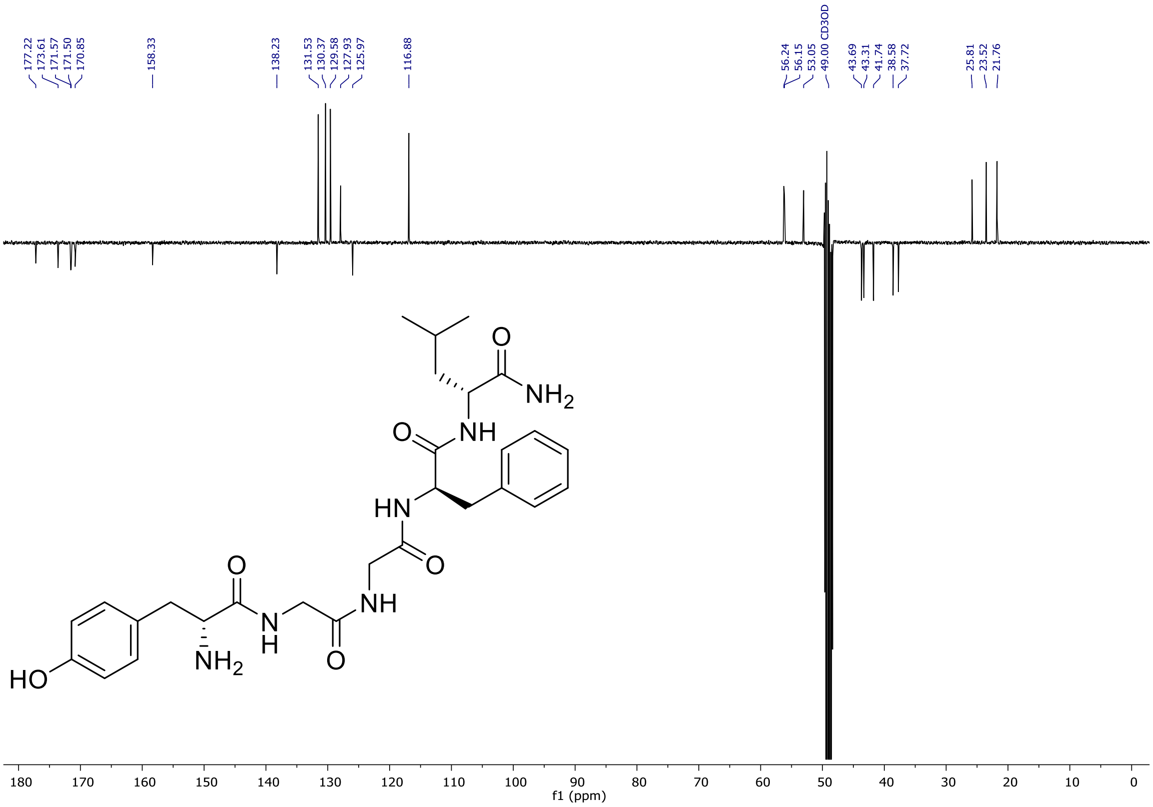

1l (Ac-Tyr-Tryptamin)

^{1}H NMR in CD_3OD, 400 MHz

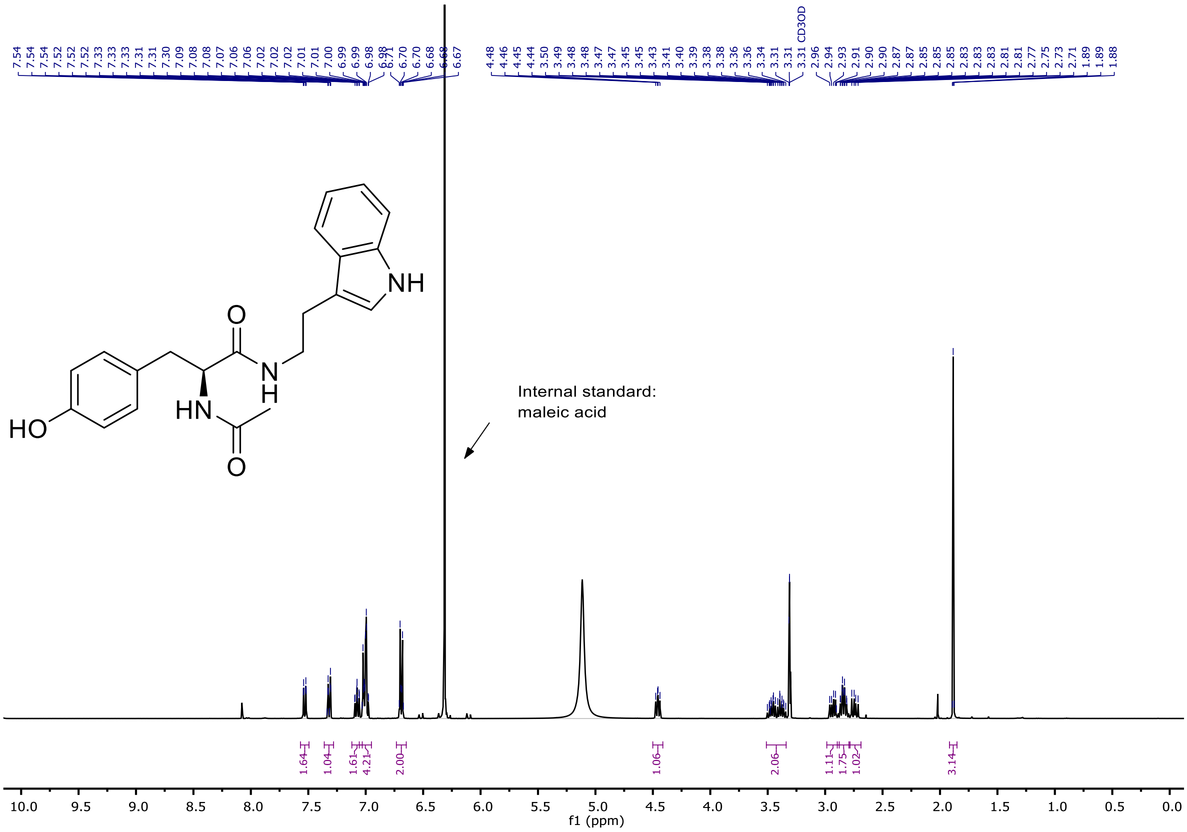

^{13}C NMR in CD_3OD, 101 MHz

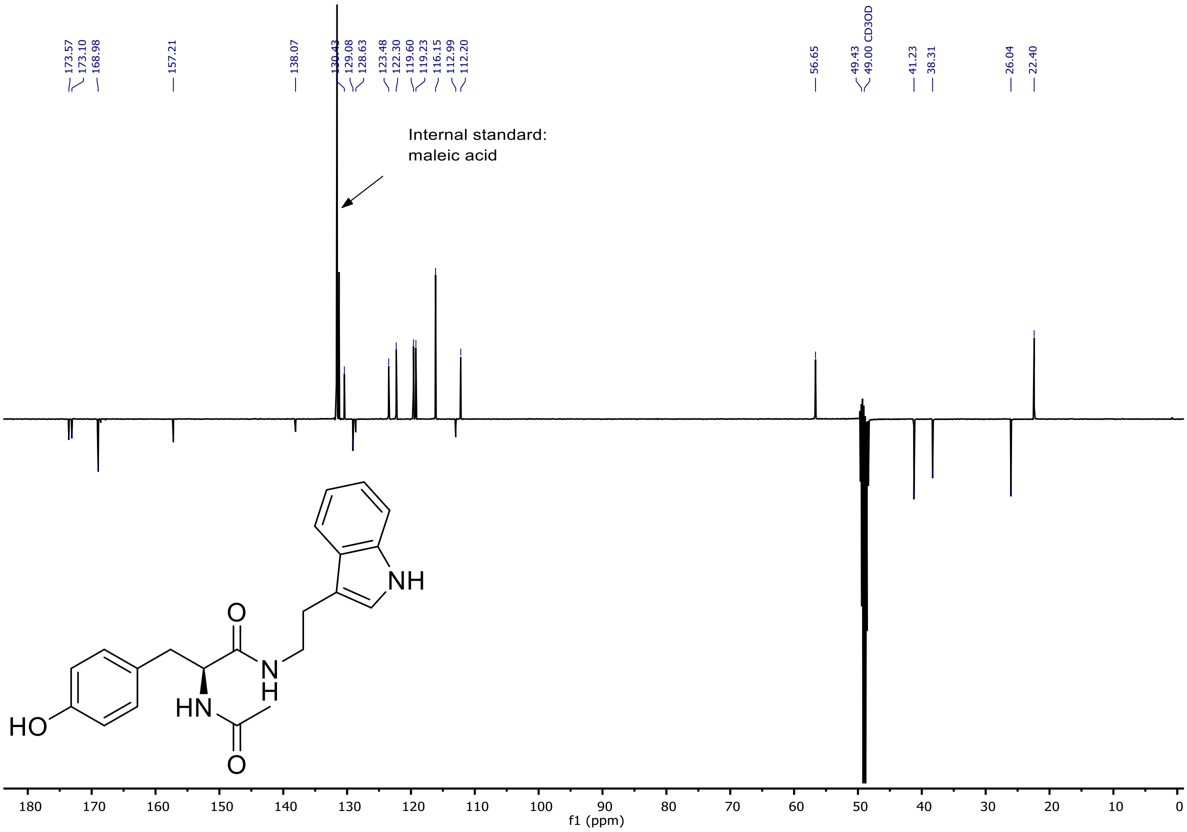

2a’

^{1}H NMR in CD_3CN, 400 MHz

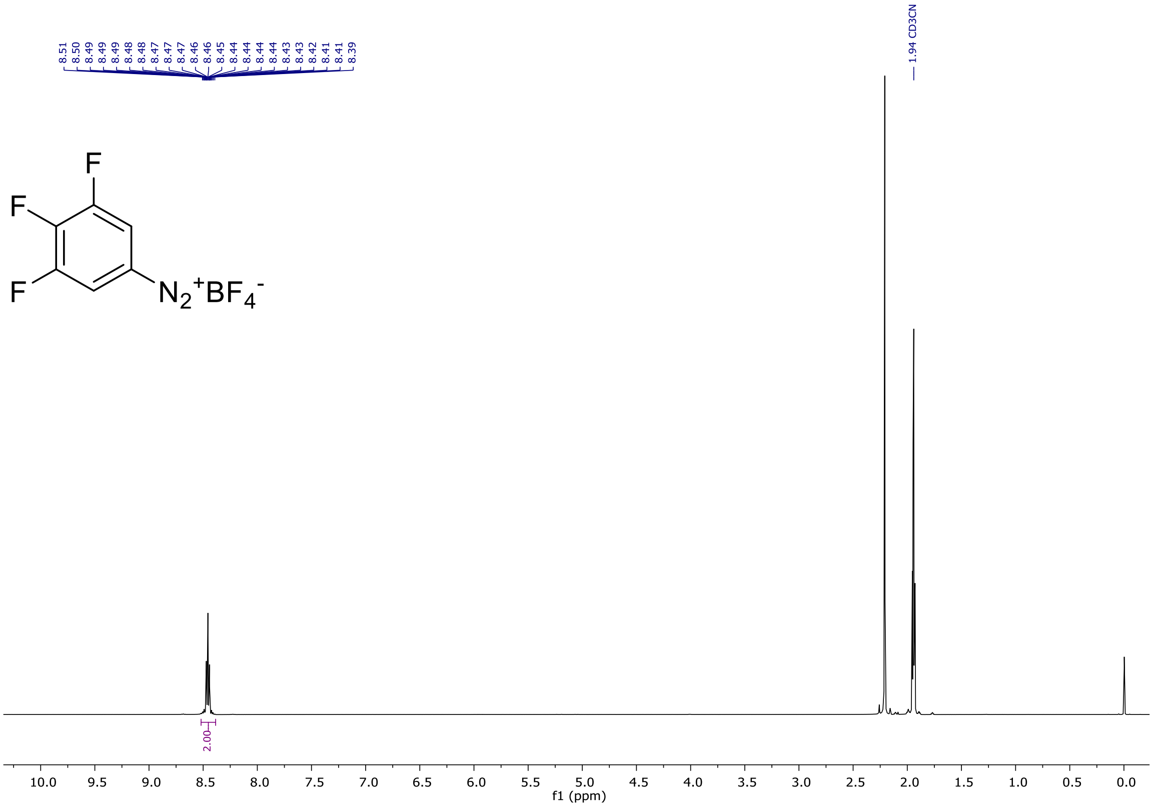

2b’

^{1}H NMR in CD_3CN, 400 MHz

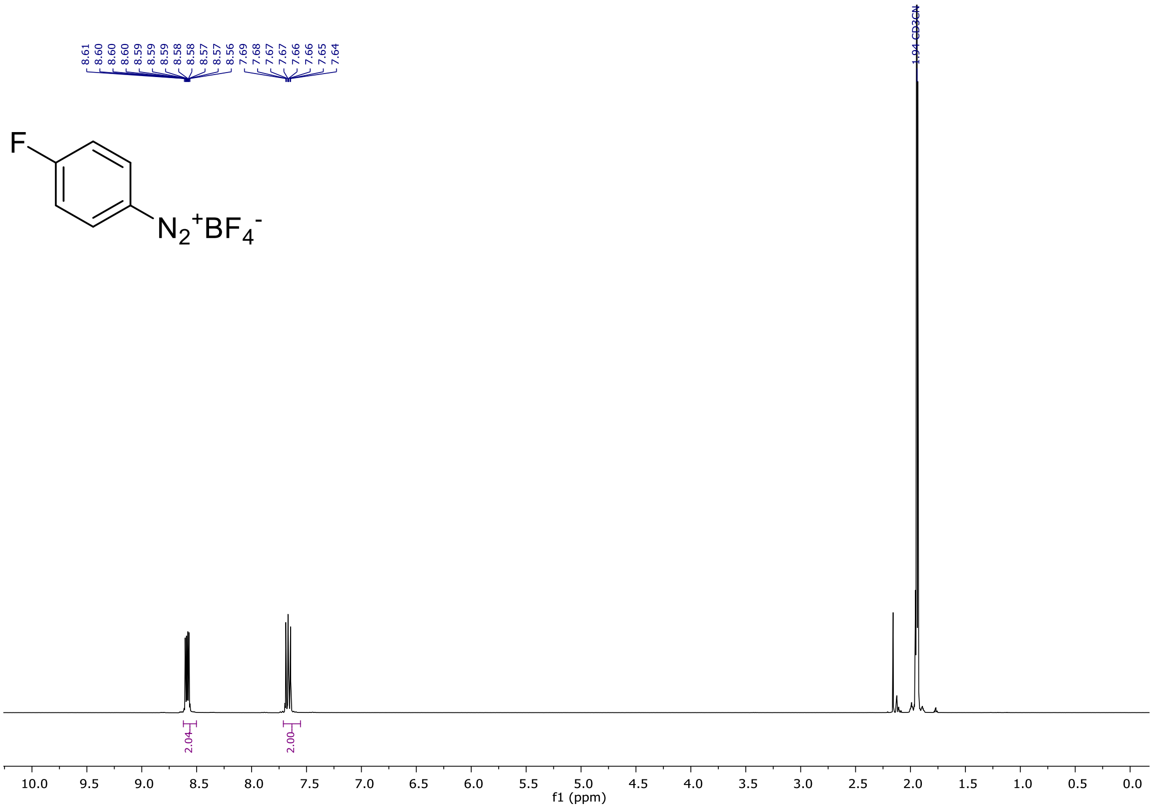

2d’

^{1}H NMR in CD_3CN, 400 MHz

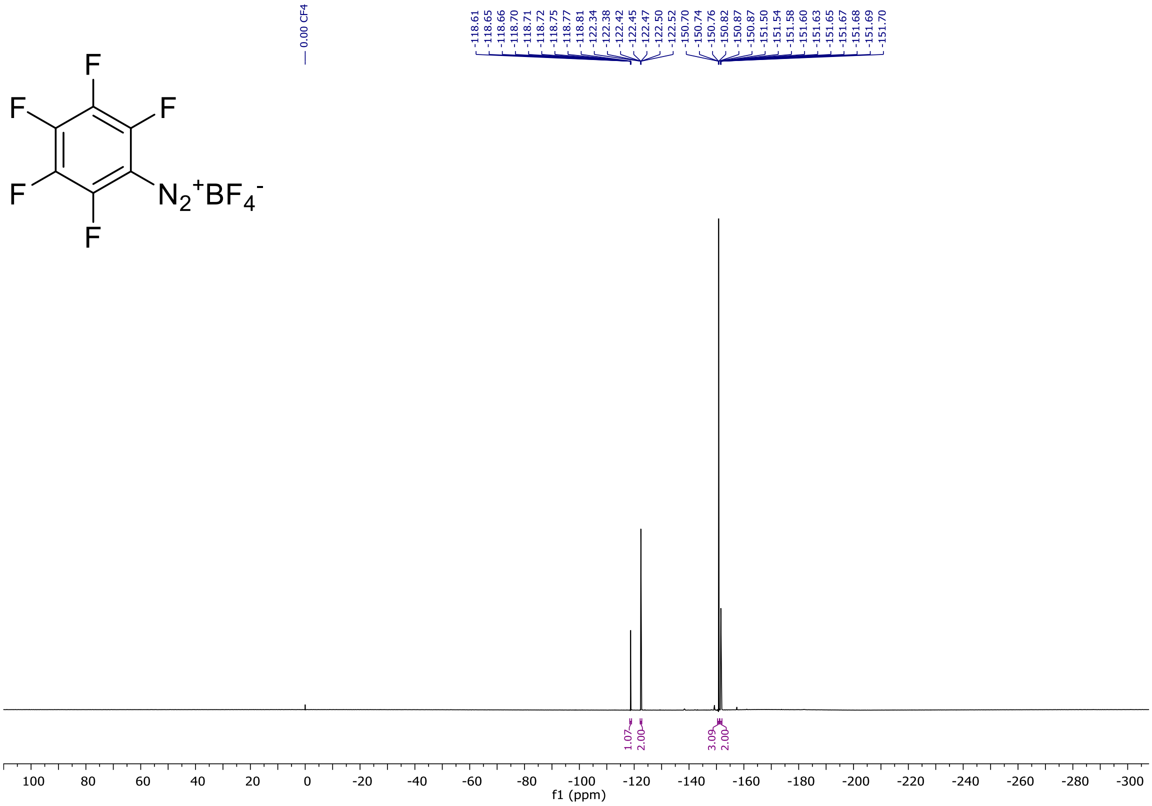

2f’

^{1}H NMR in CD_3CN, 400 MHz

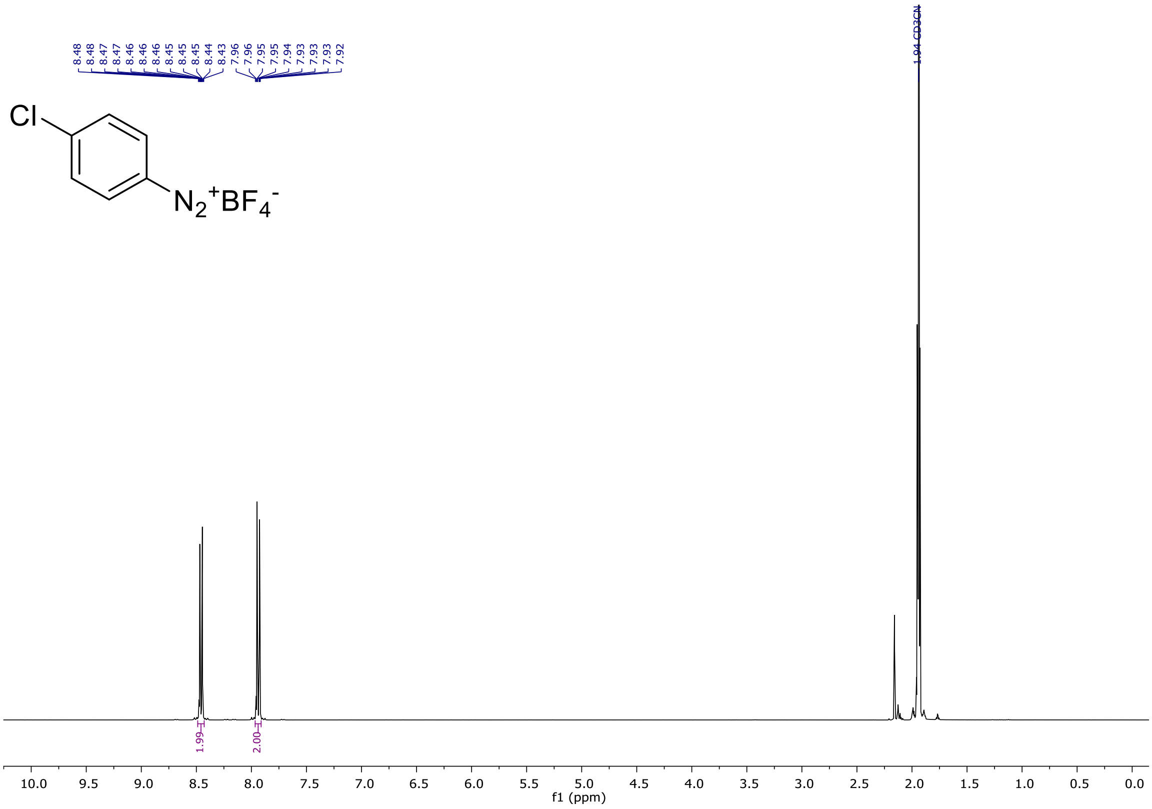

2g’

^{1}H NMR in CD_3CN, 400 MHz

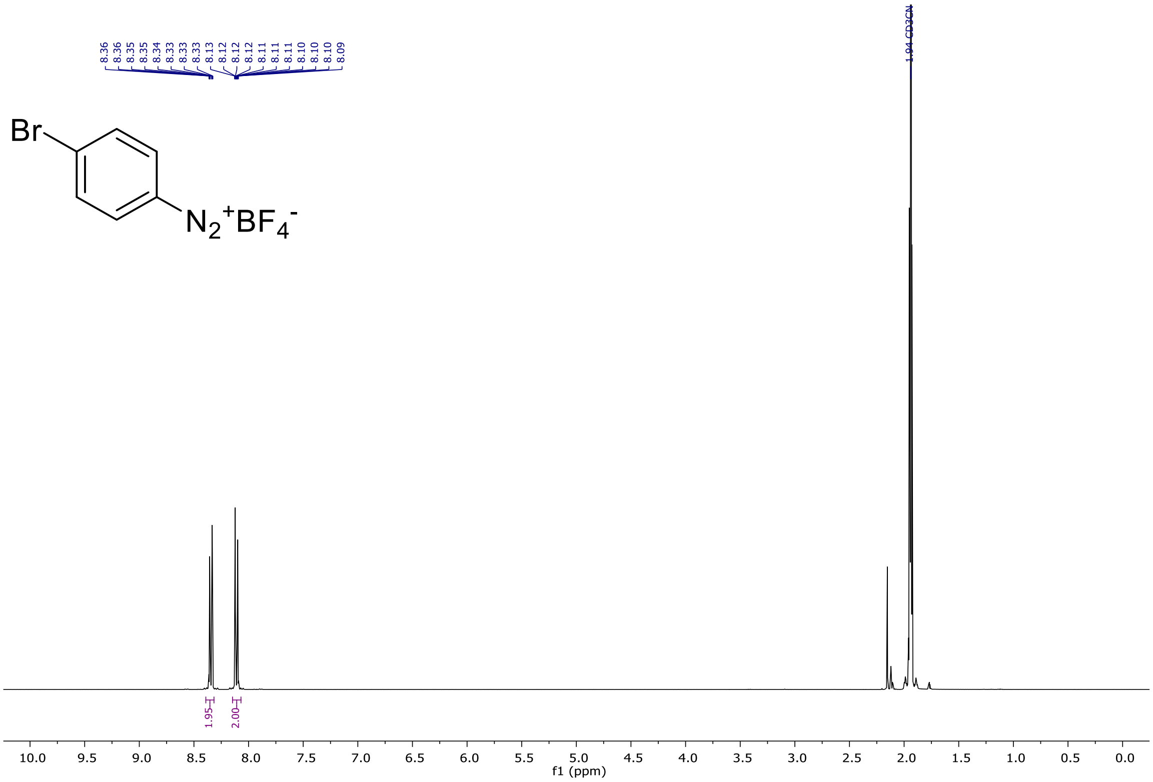

2j

^{1}H NMR in CD_3CN, 400 MHz

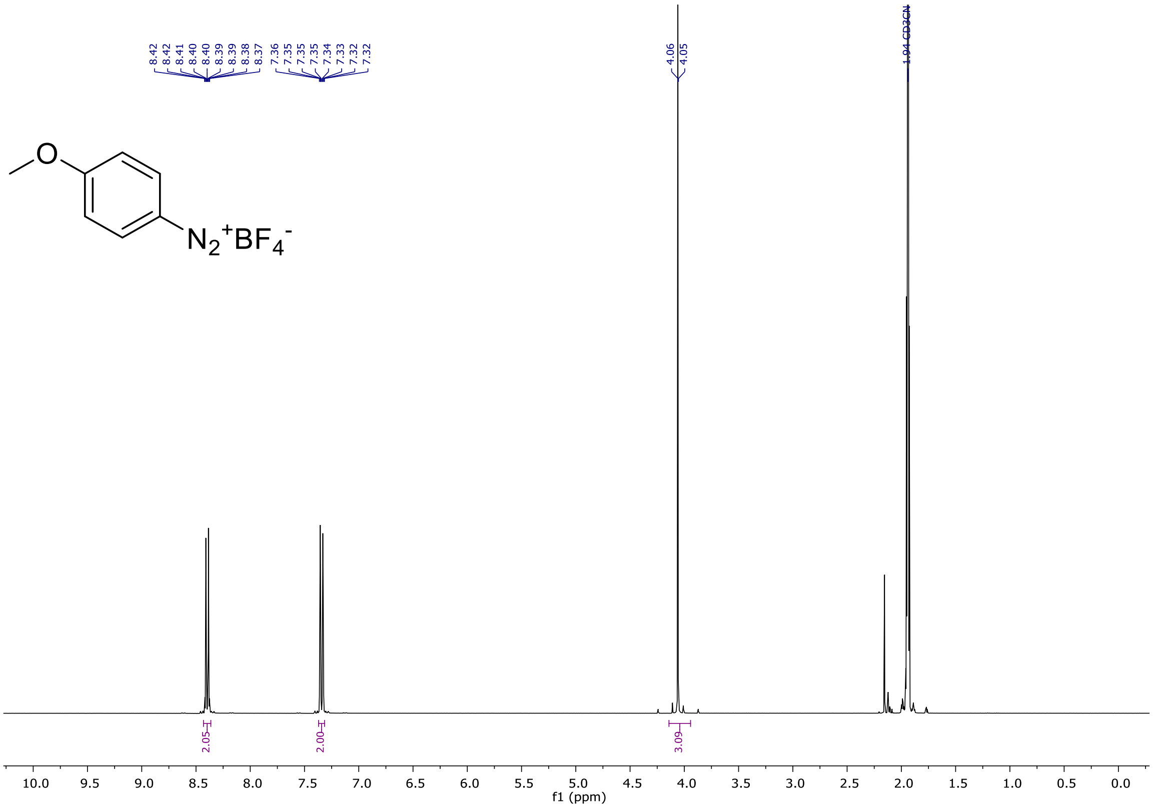

2k

^{1}H NMR in CD_3CN, 400 MHz

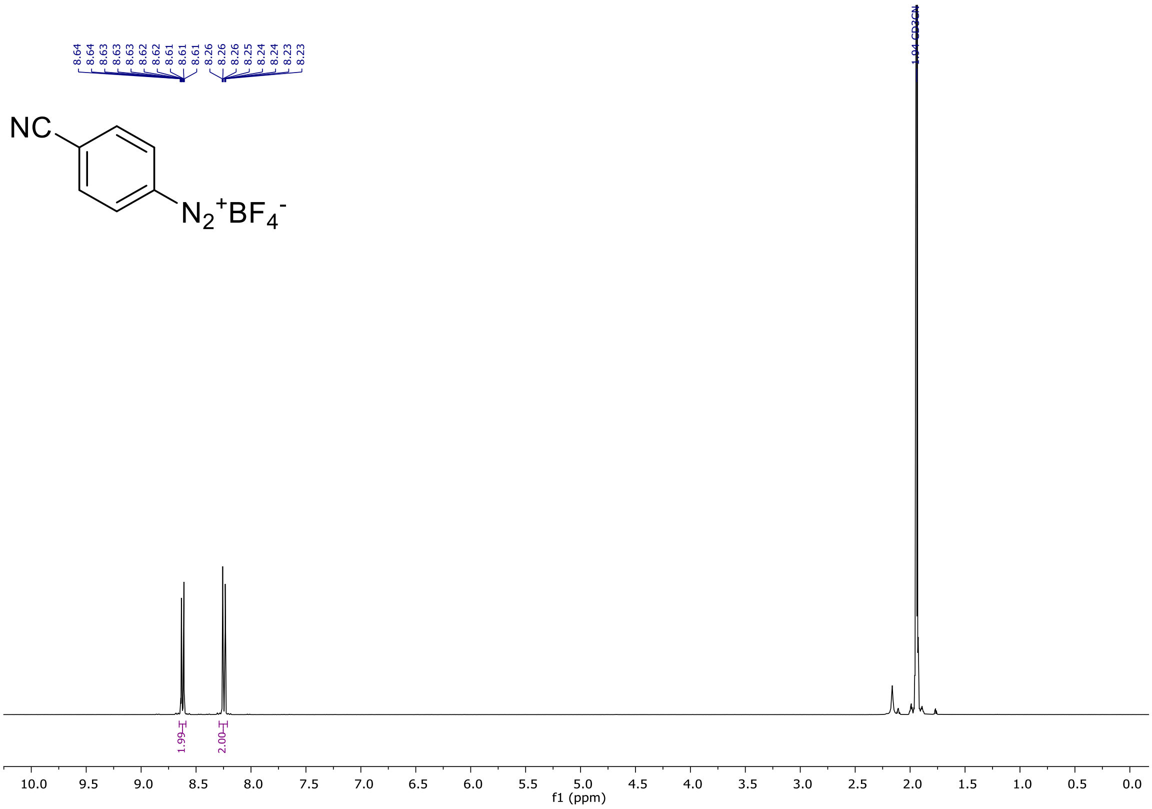

3aa

^{1}H NMR in CD_3OD, 400 MHz

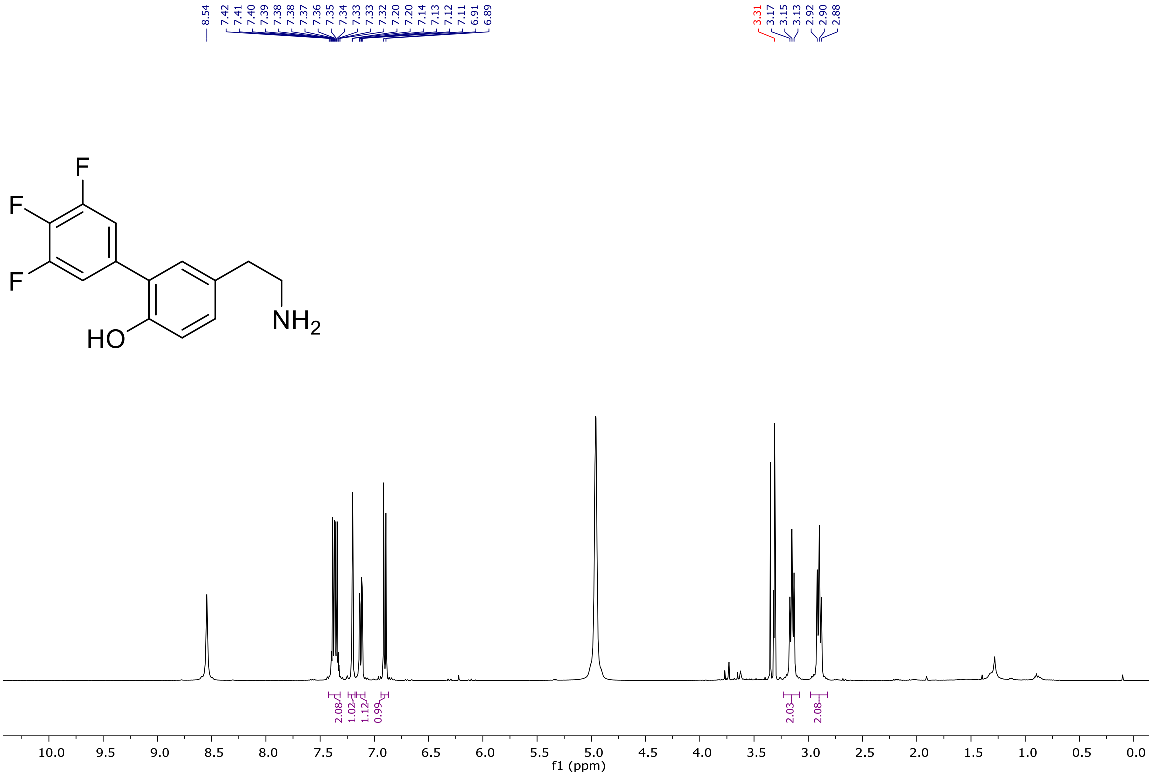

^{13}C NMR in CD_3OD, 101 MHz

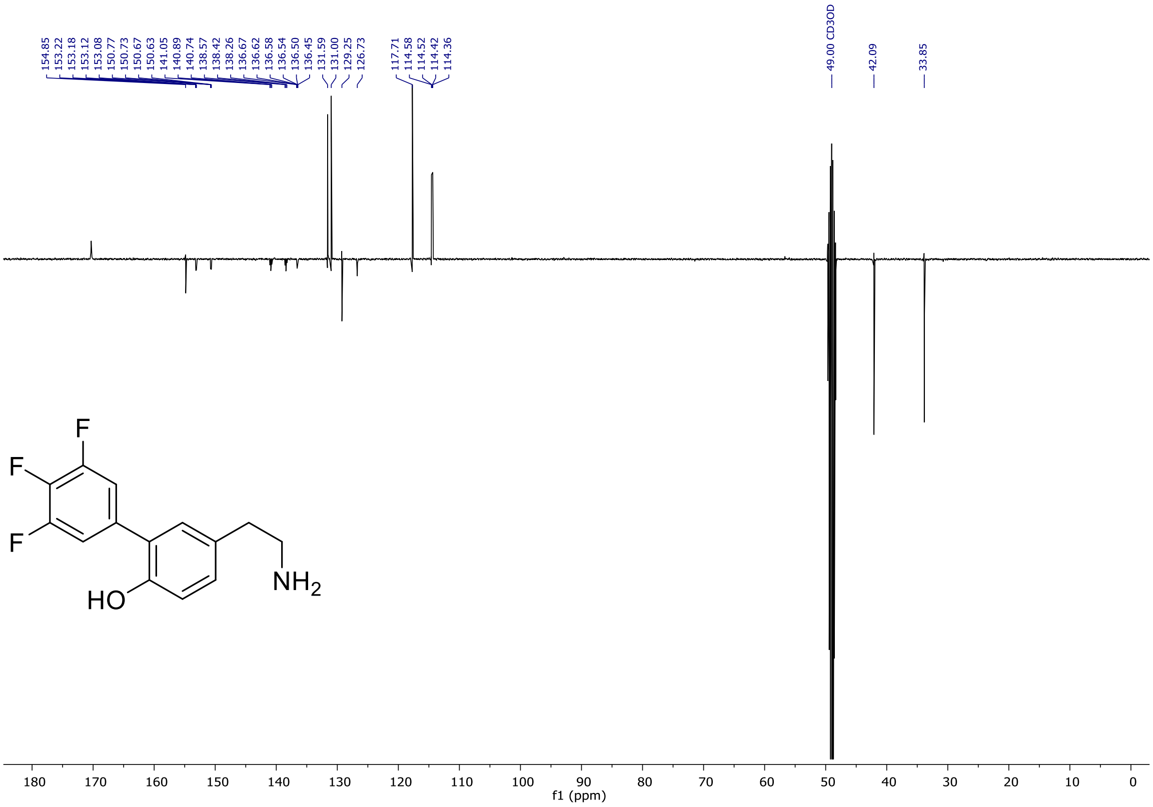

3ba

^{1}H NMR in CD_3OD, 400 MHz

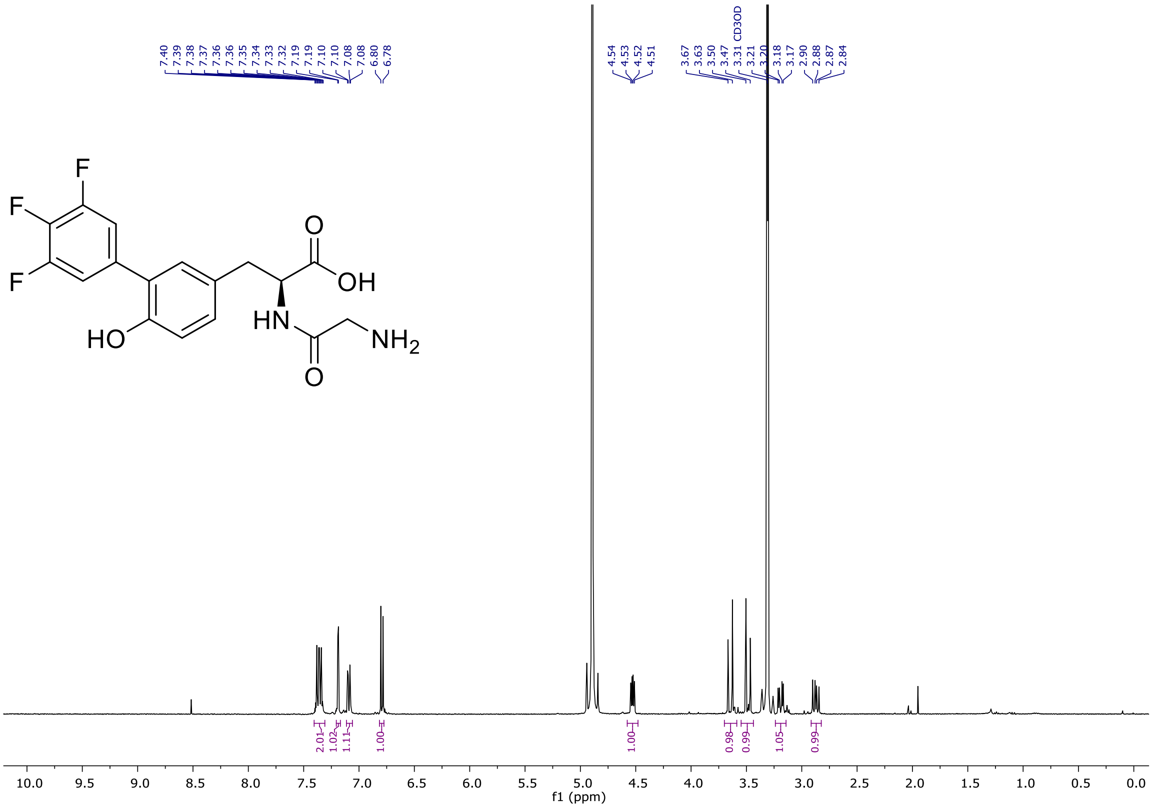

^{13}C-NMR in CD_3OD, 101 MHz

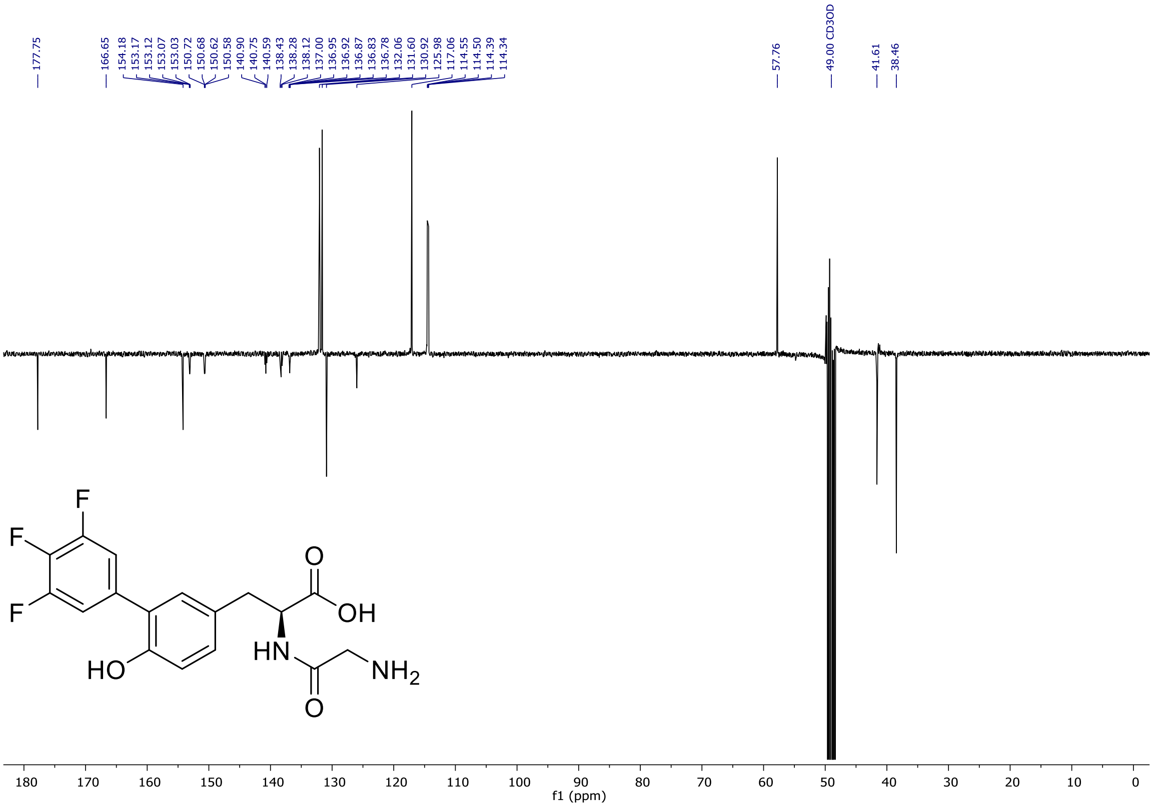

3ba'

^{1}H NMR in CD_3OD, 400 MHz

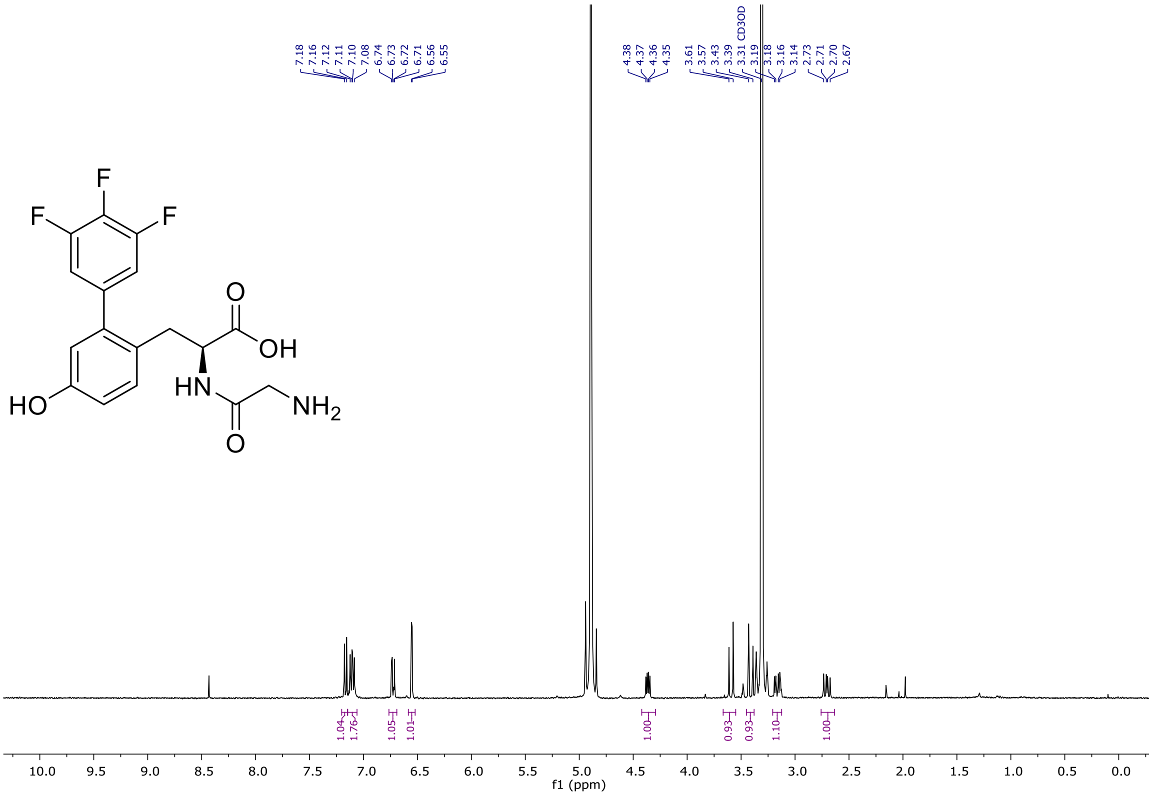

^{13}C-NMR in CD_3OD, 101 MHz

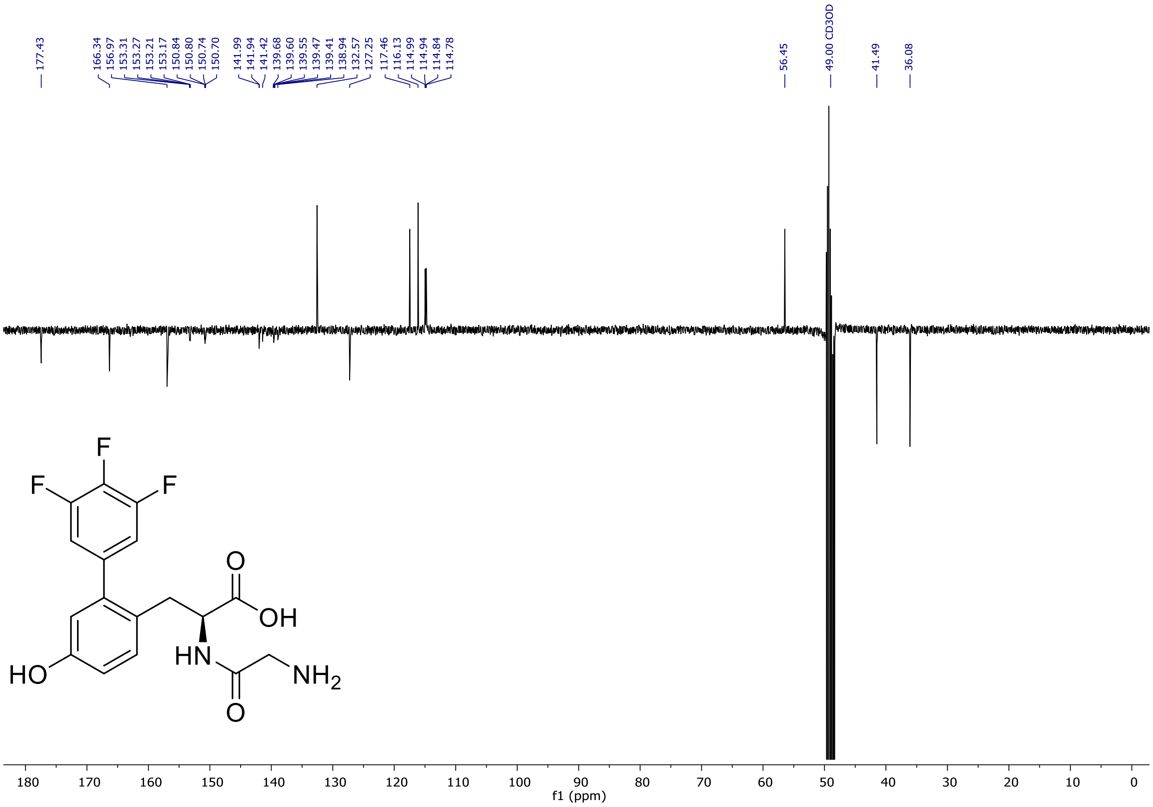

3bc

^{1}H NMR in CD_3OD, 400 MHz

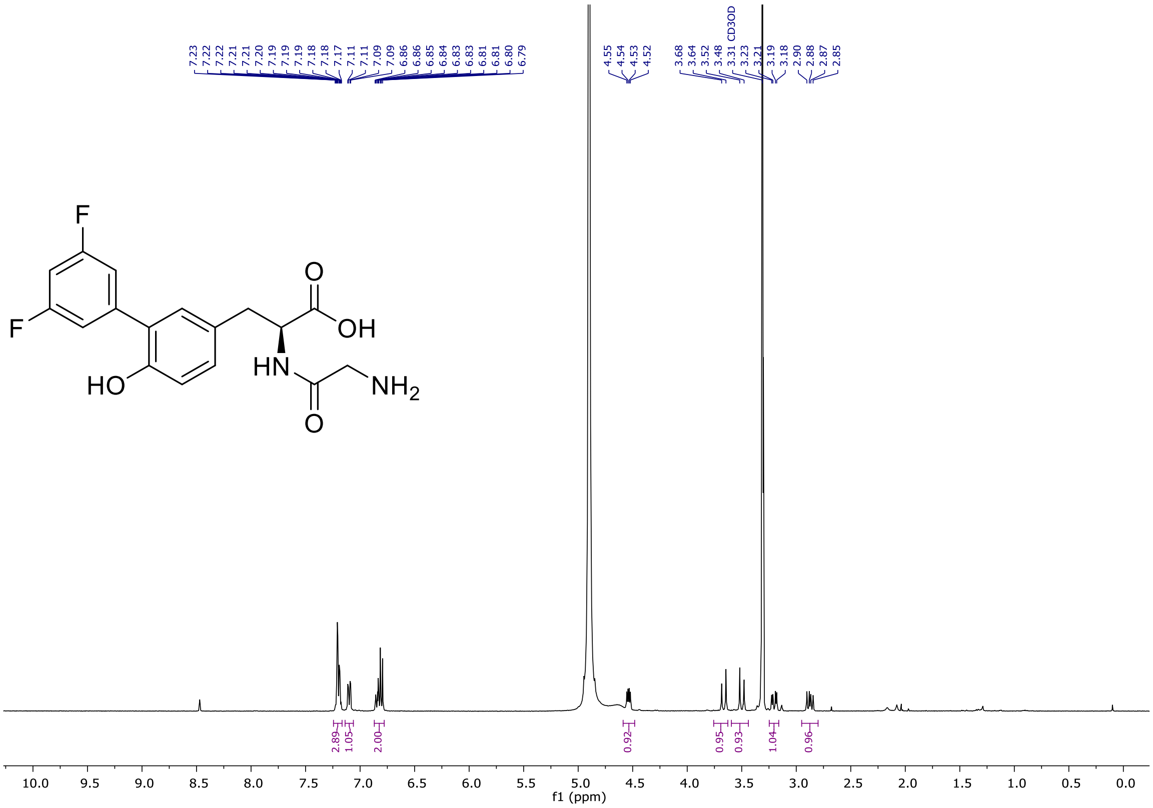

^{13}C-NMR in CD_3OD, 101 MHz

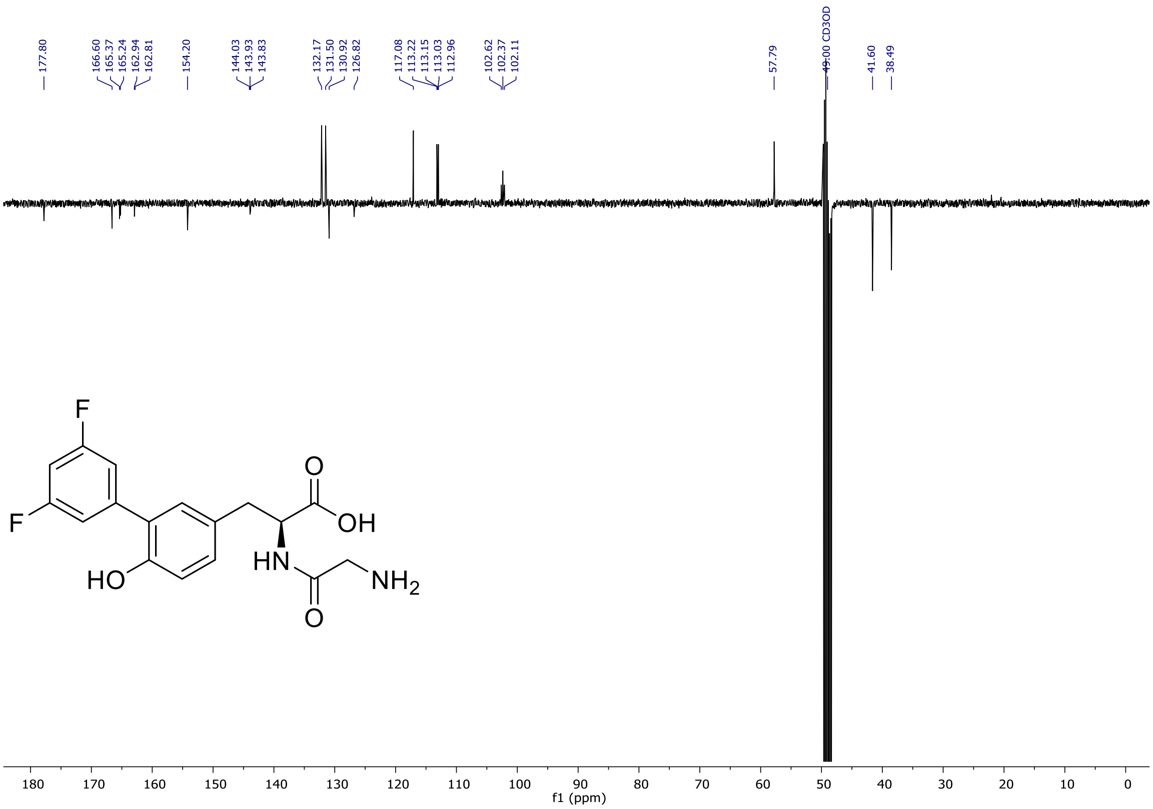

3ca

^{1}H NMR in CD_3OD, 400 MHz

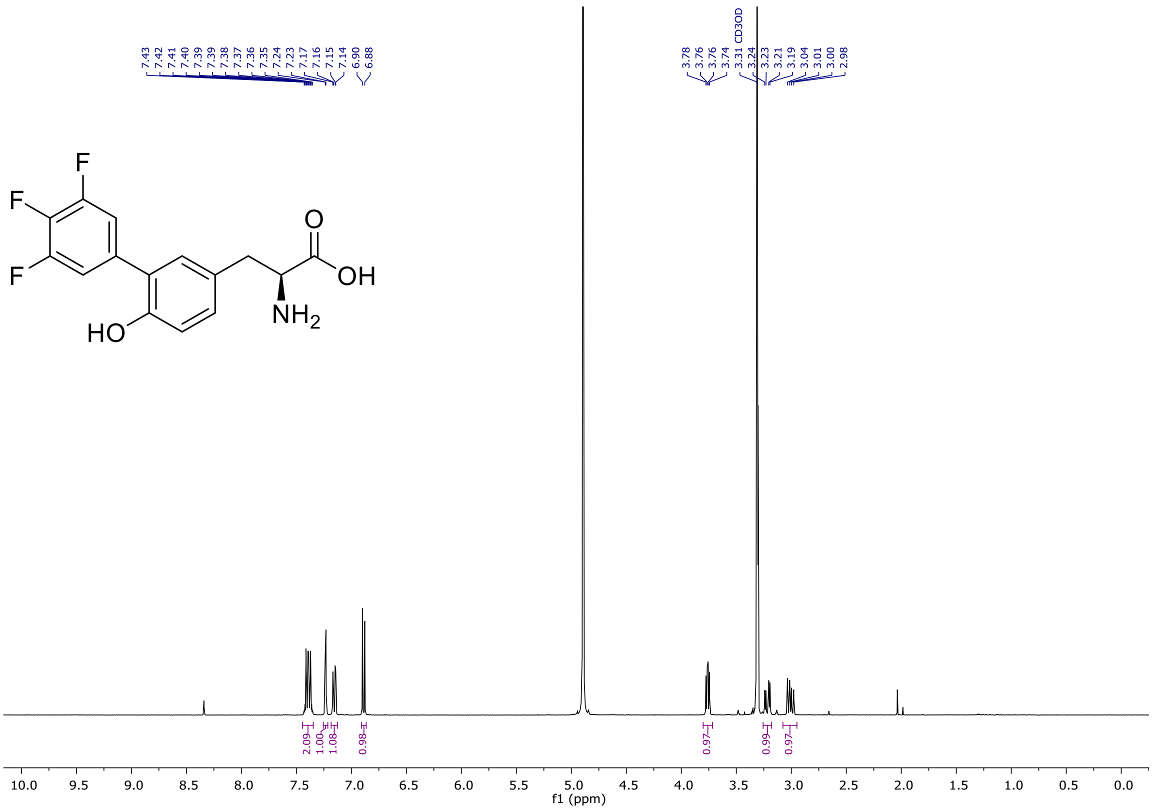

^{13}C-NMR in CD_3OD, 101 MHz

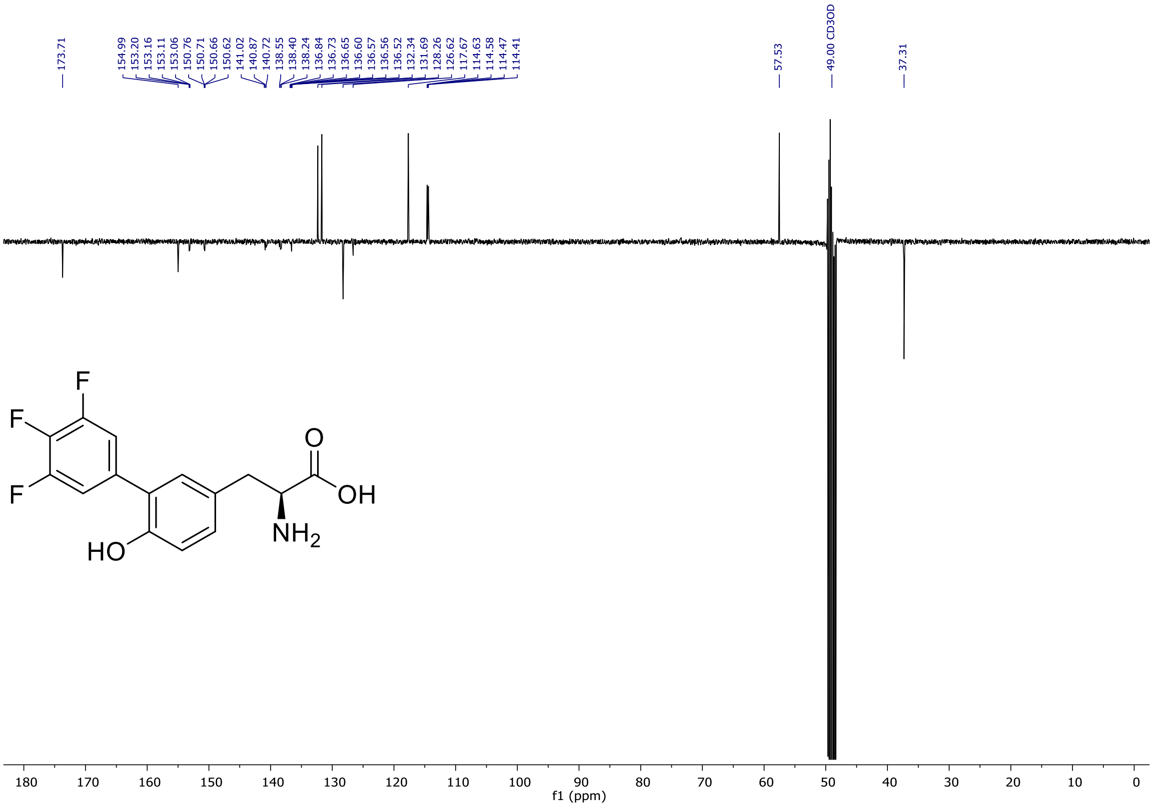

3da

^{1}H NMR in CD_3OD, 400 MHz

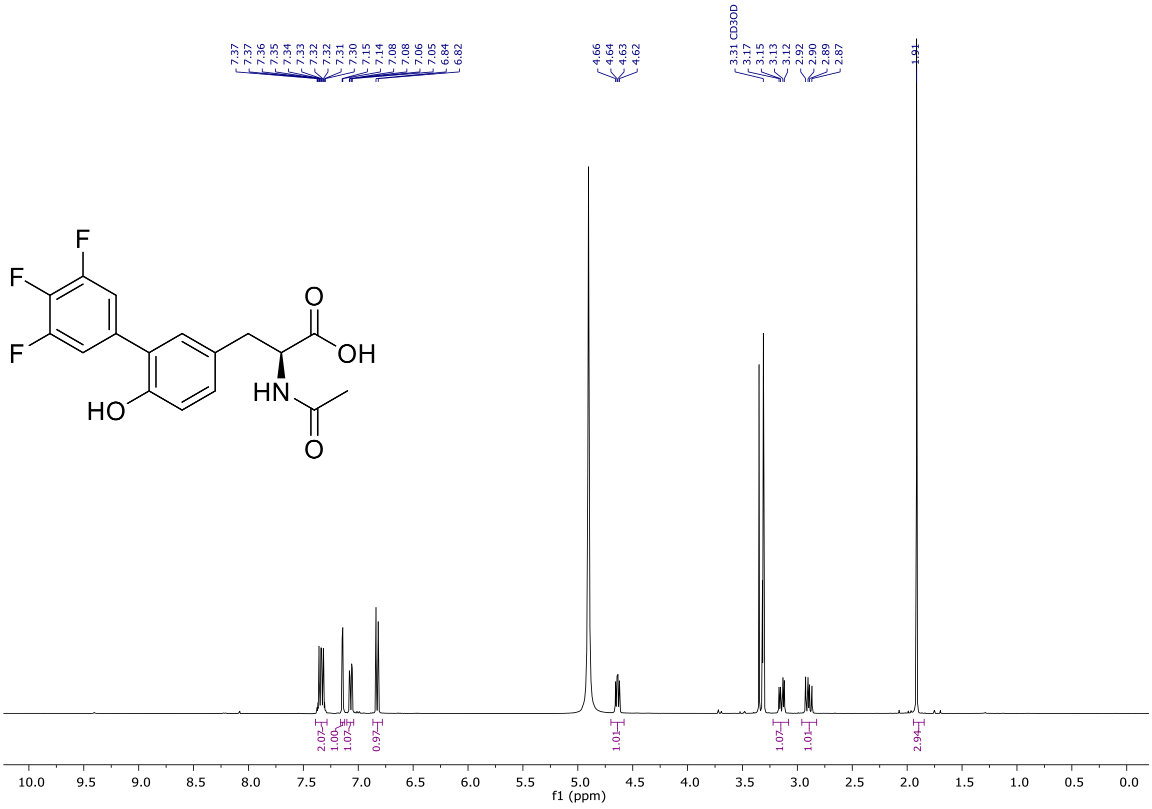

^{13}C-NMR in CD_3OD, 101 MHz

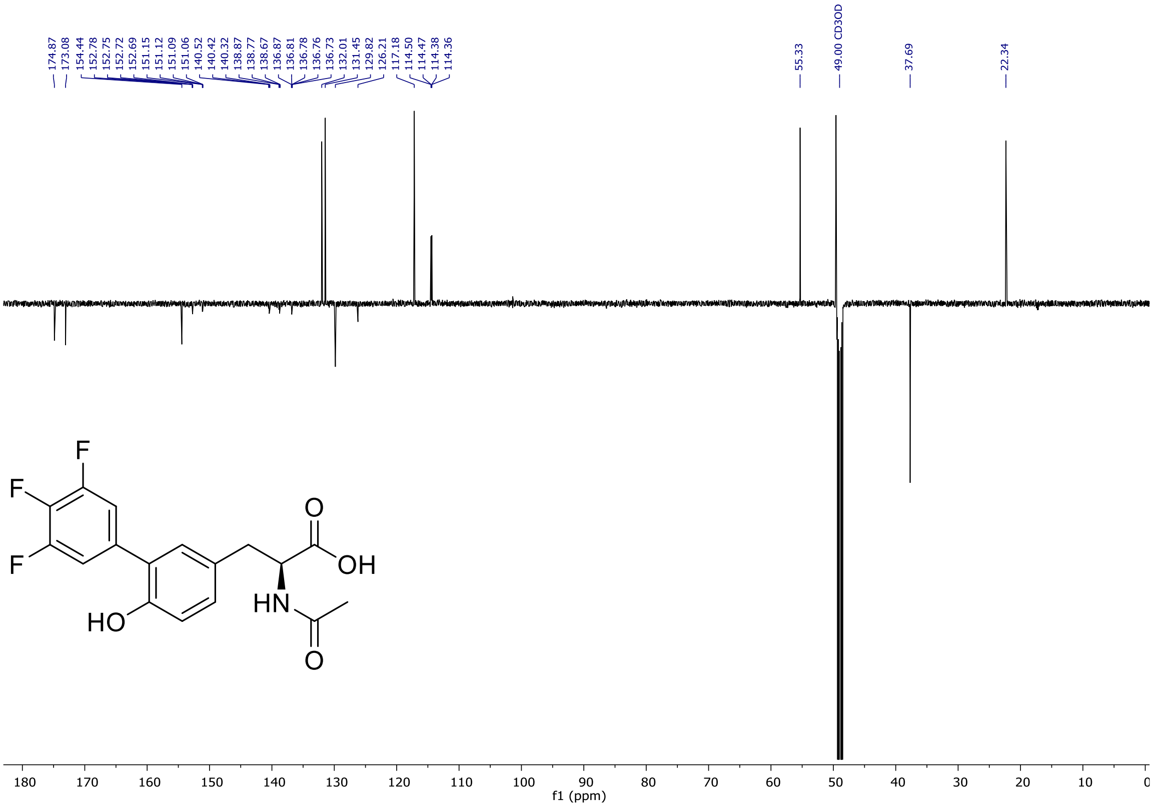

3da‘

^{1}H NMR in CD_3OD, 400 MHz

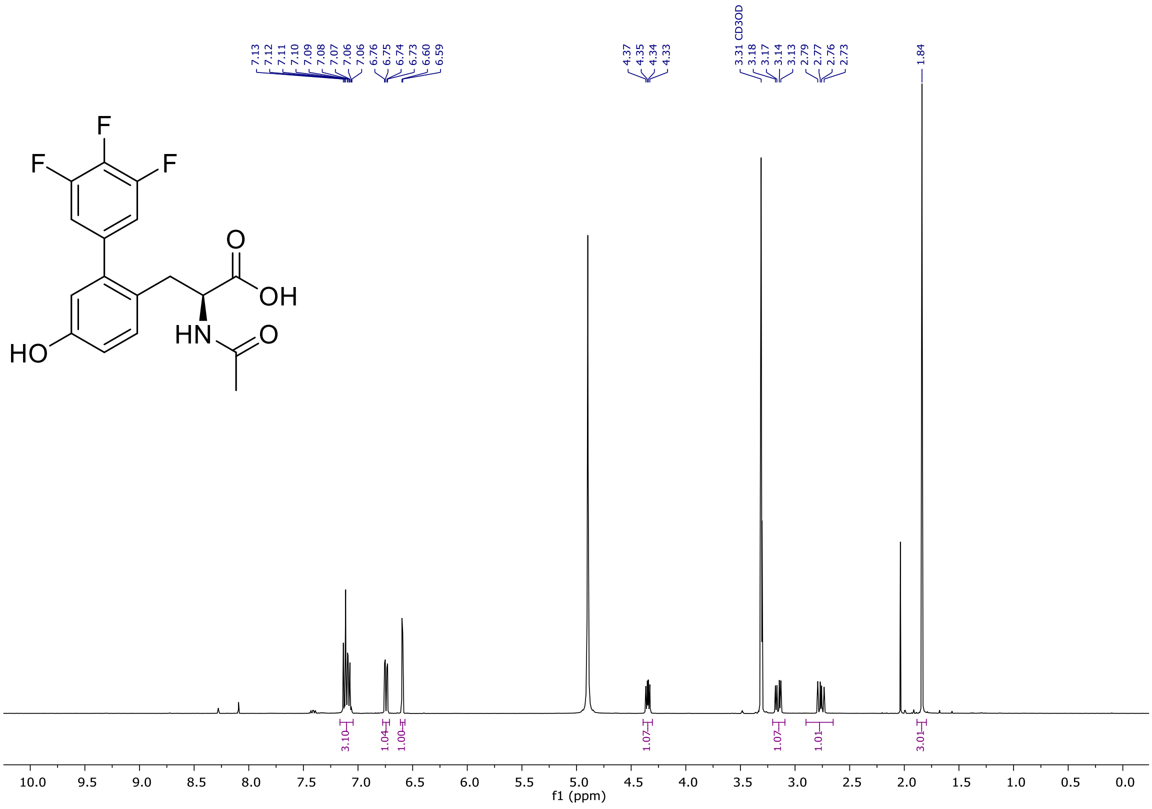

^{13}C-NMR in CD_3OD, 101 MHz

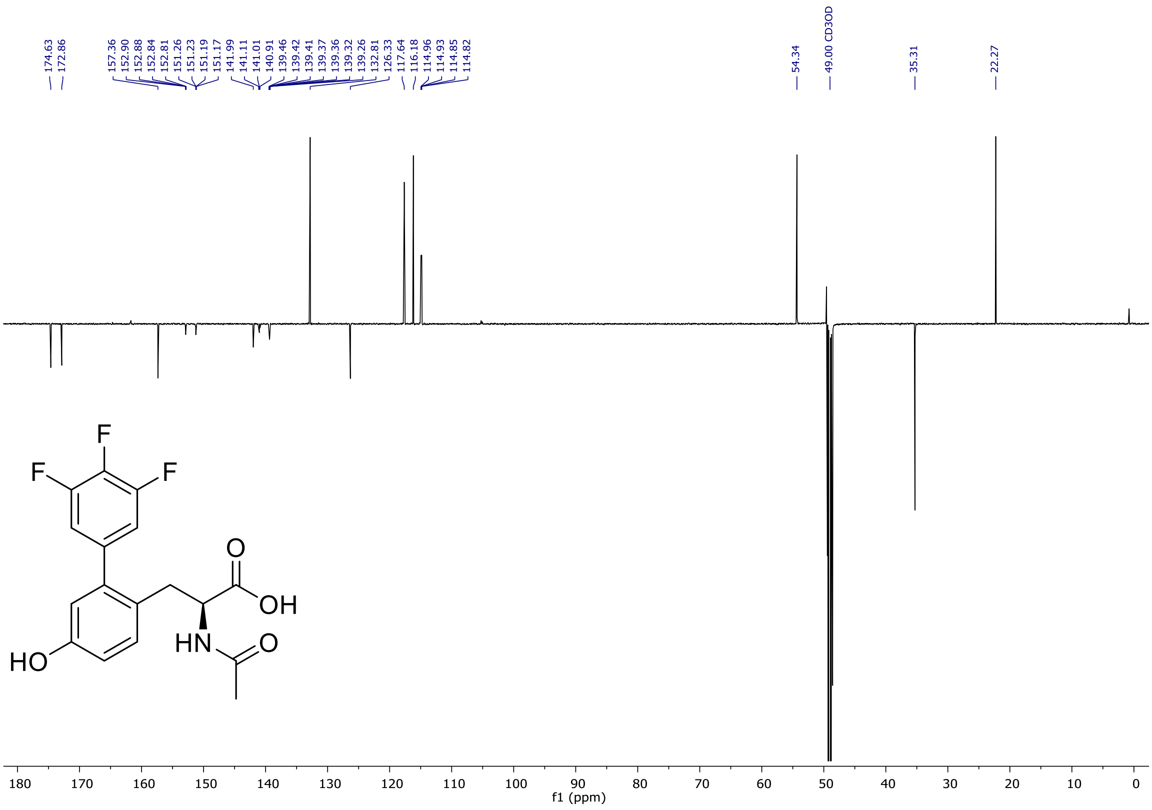

3ea

^{1}H NMR in CD_3OD, 400 MHz

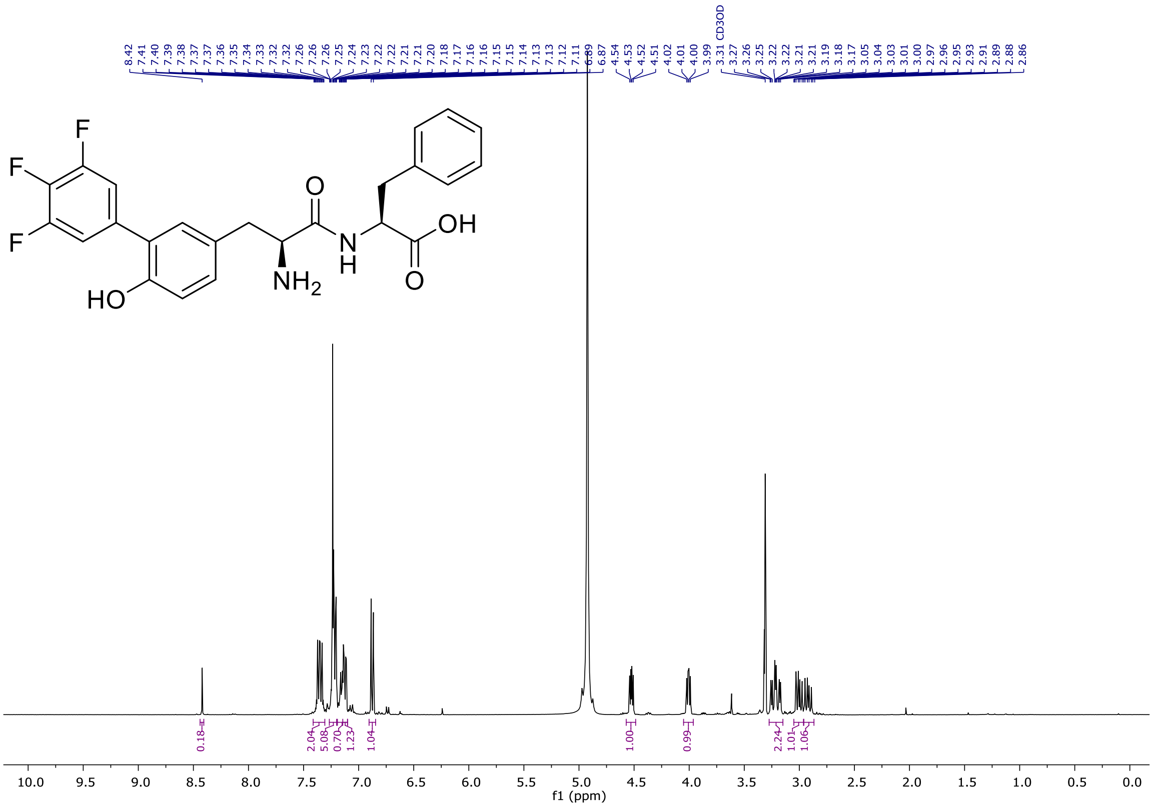

^{13}C-NMR in CD_3OD, 101 MHz

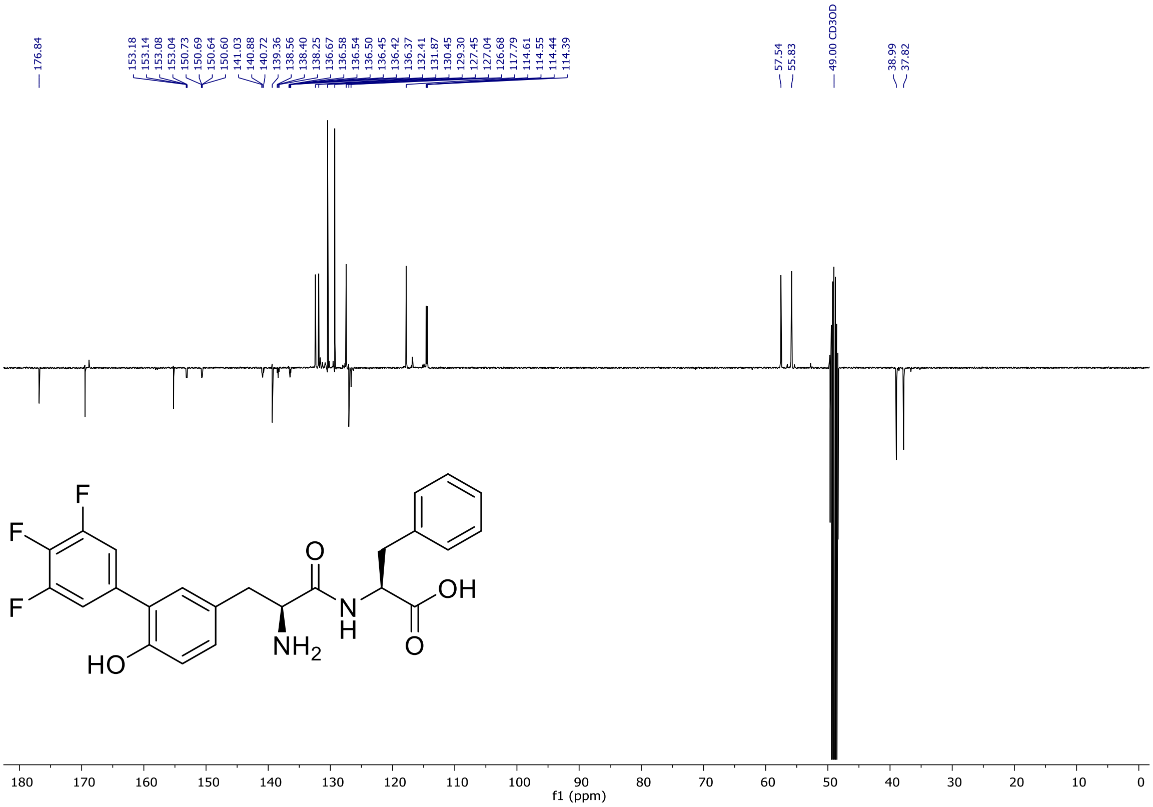

3ea‘

^{1}H NMR in CD_3OD, 400 MHz

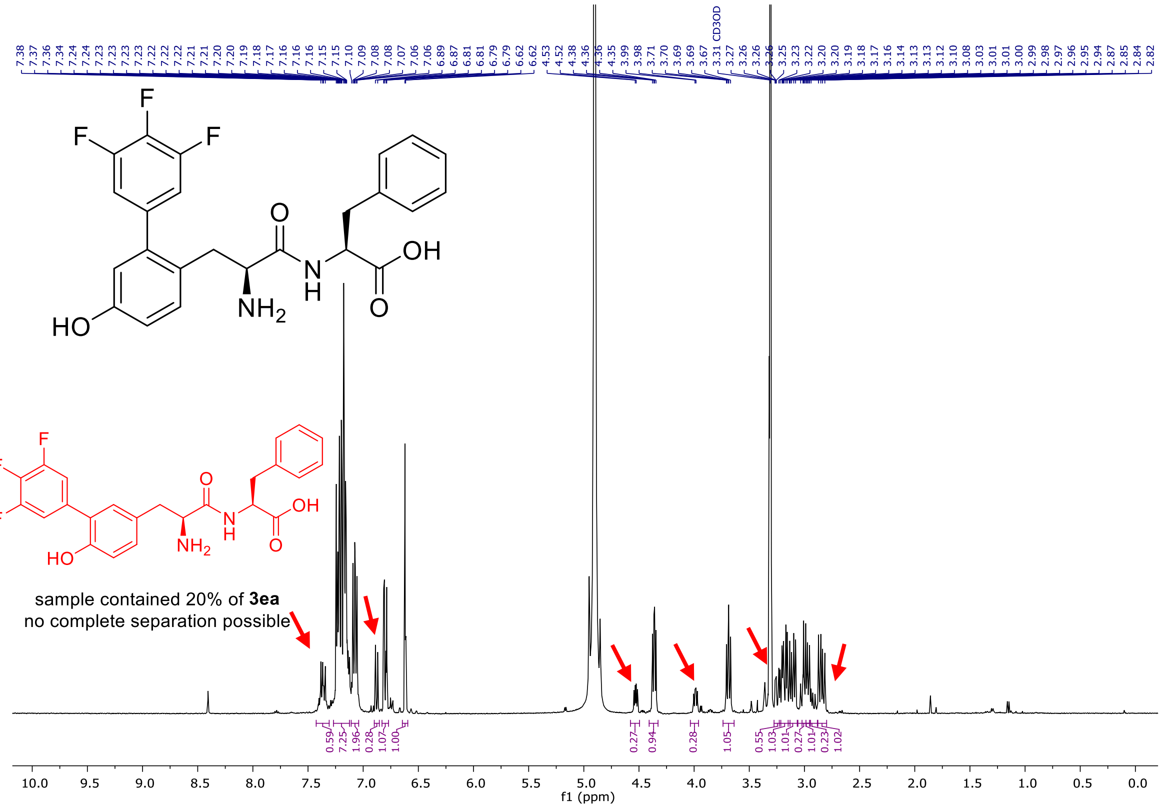

^{13}C-NMR in CD_3OD, 101 MHz

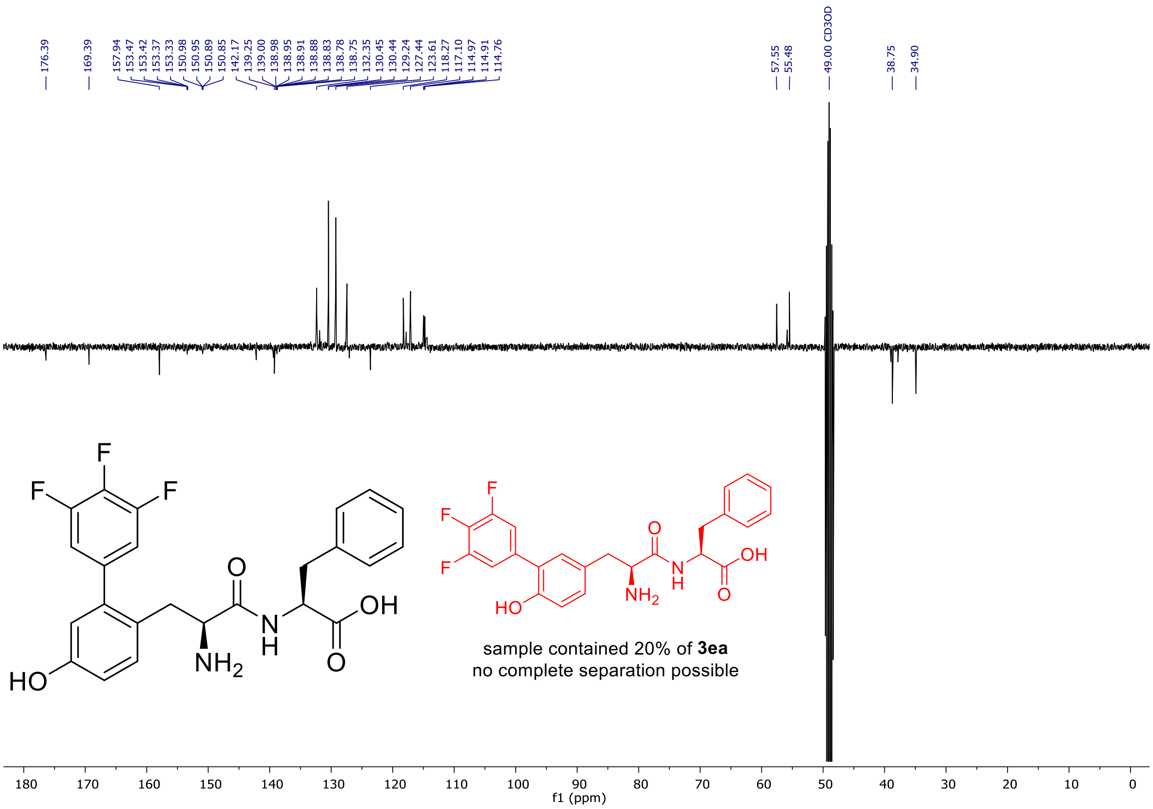

3ea‘‘

^{1}H NMR in CD_3OD, 400 MHz

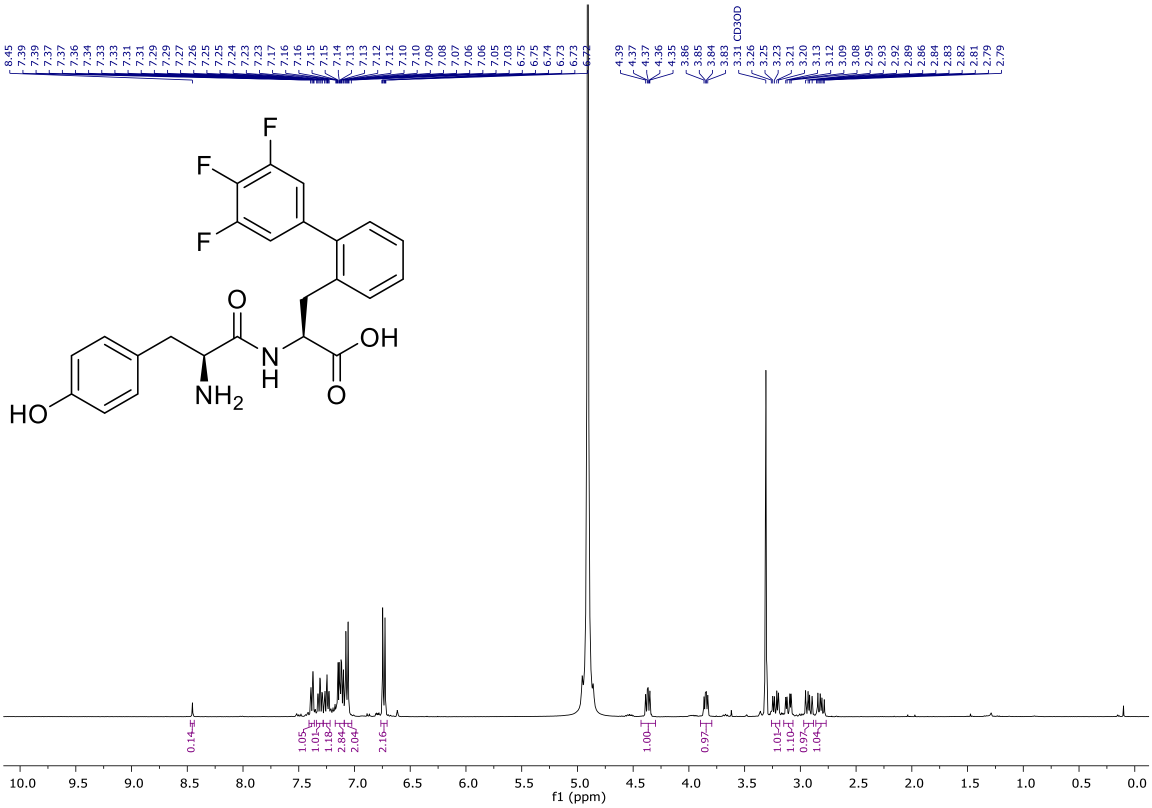

^{13}C-NMR in CD_3OD, 101 MHz

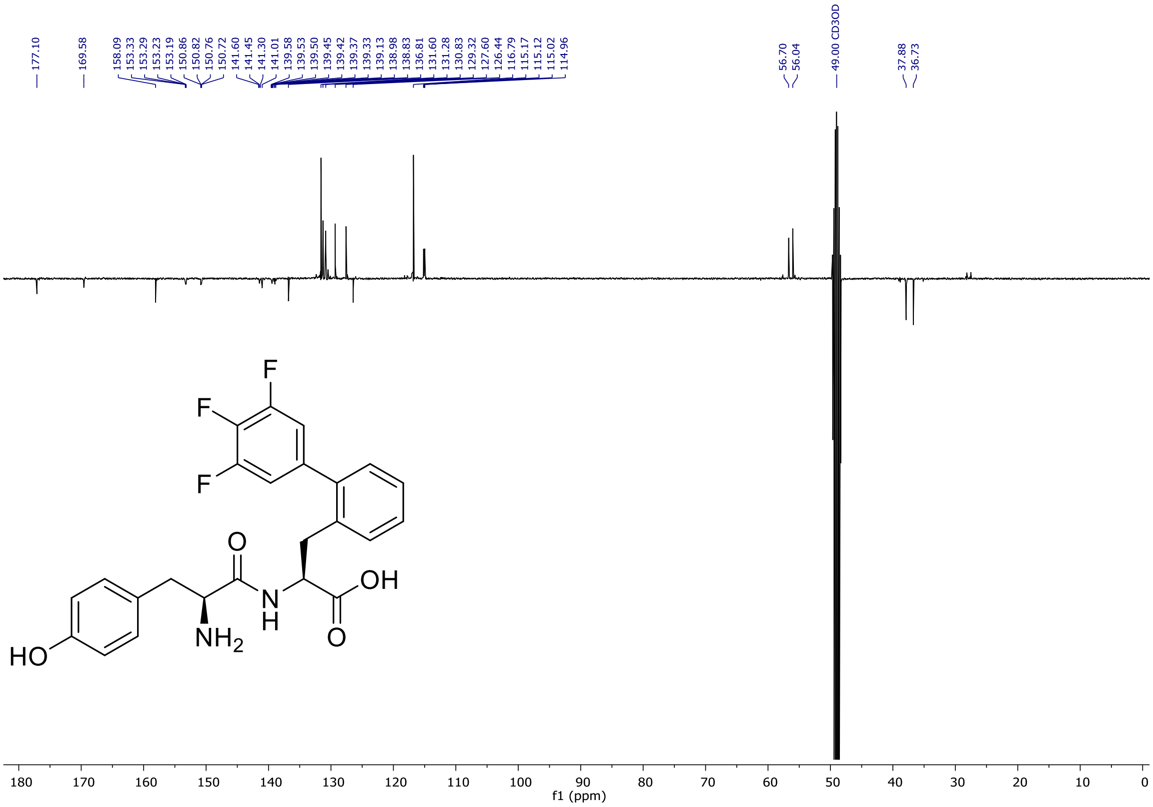

3fa

^{1}H NMR in CD_3OD, 400 MHz

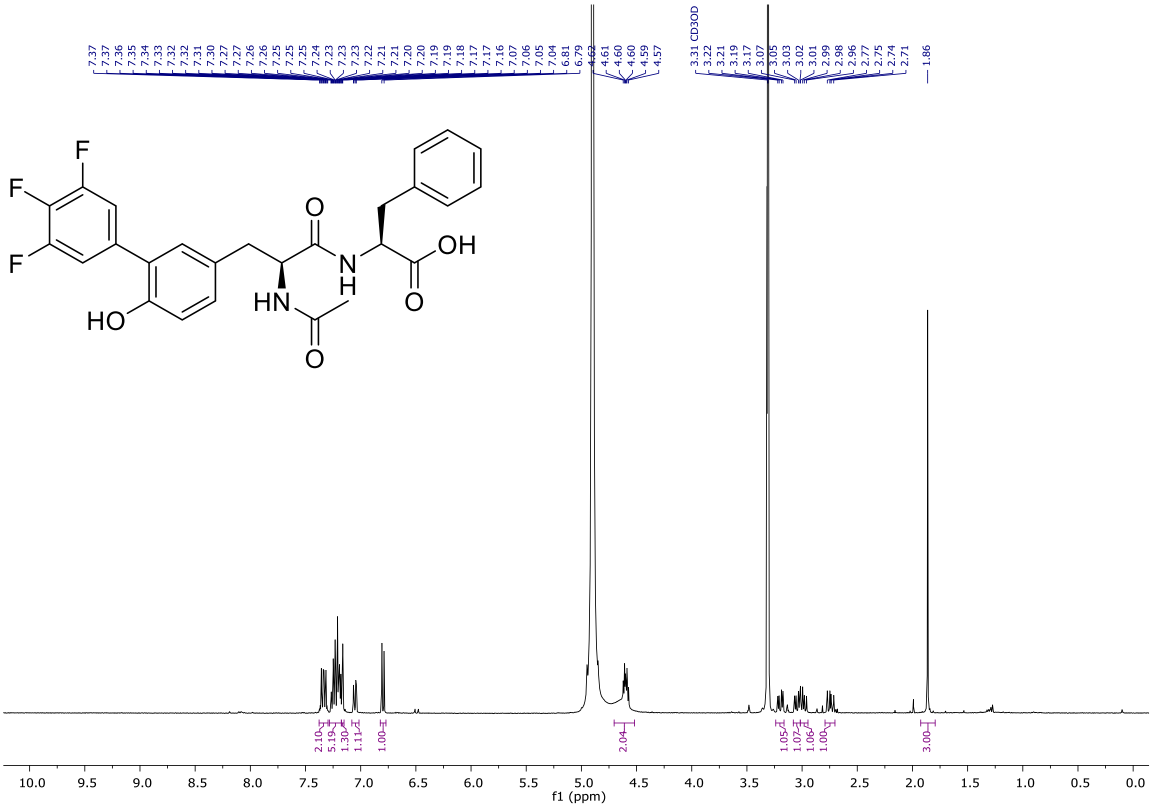

^{13}C-NMR in CD_3OD, 101 MHz

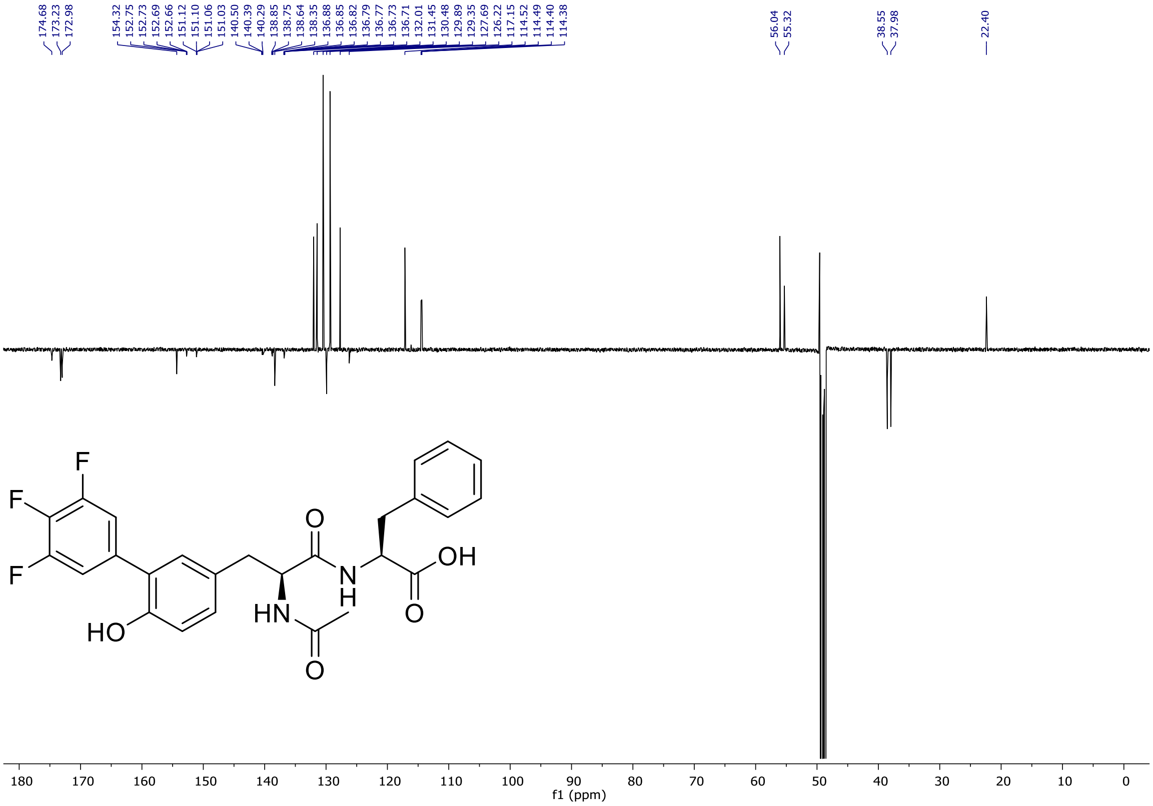

3ga

^{1}H NMR in CD_3OD, 400 MHz

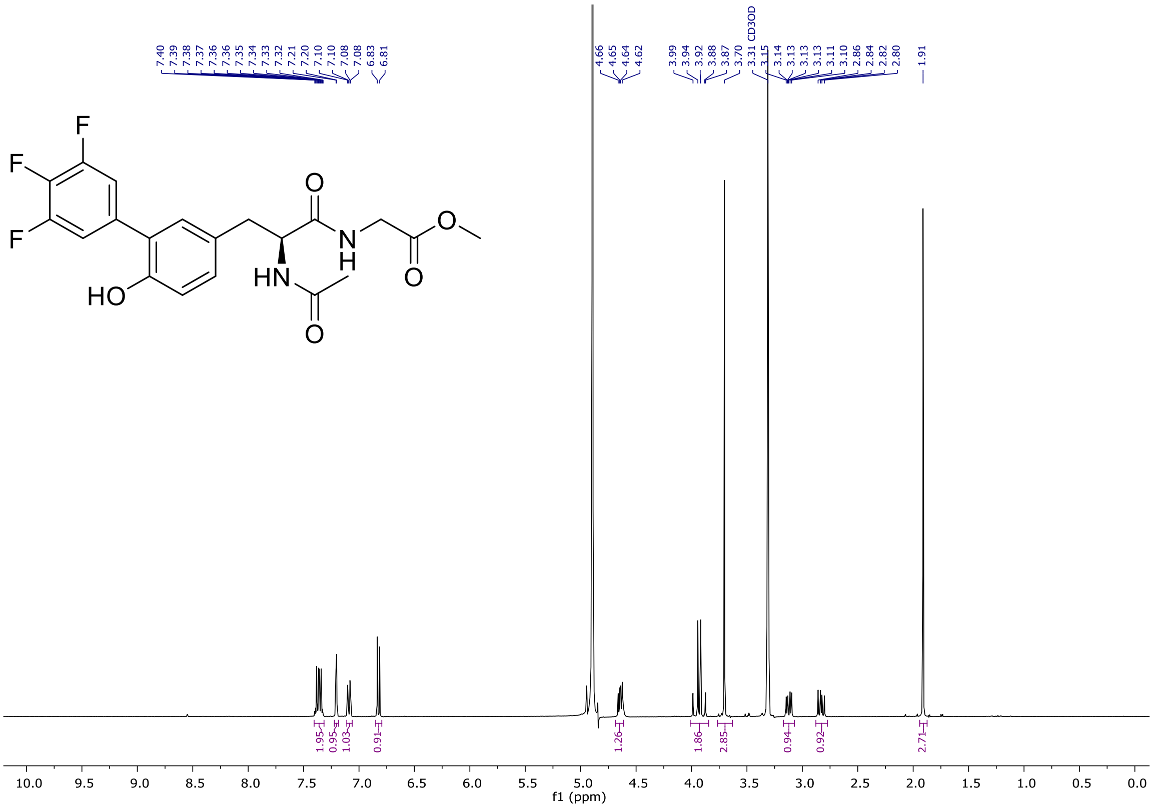

^{13}C-NMR in CD_3OD, 101 MHz

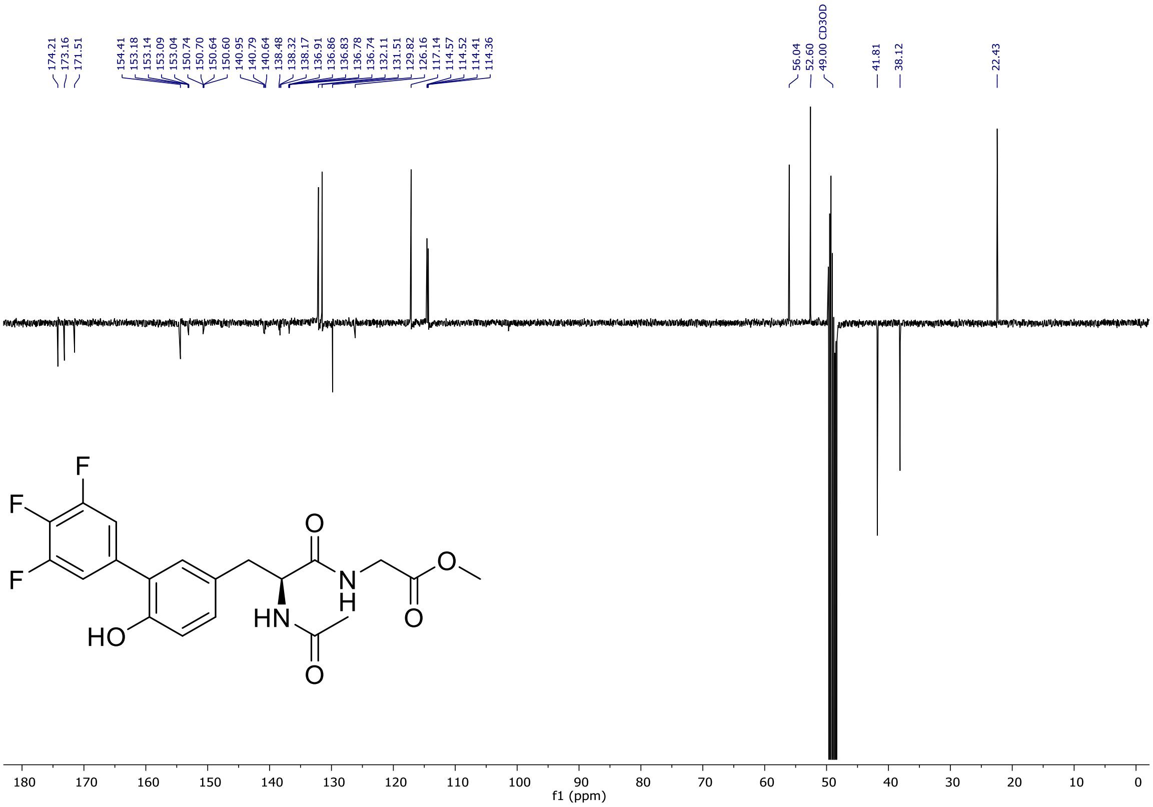

3ga'

^{1}H NMR in CD_3OD, 400 MHz

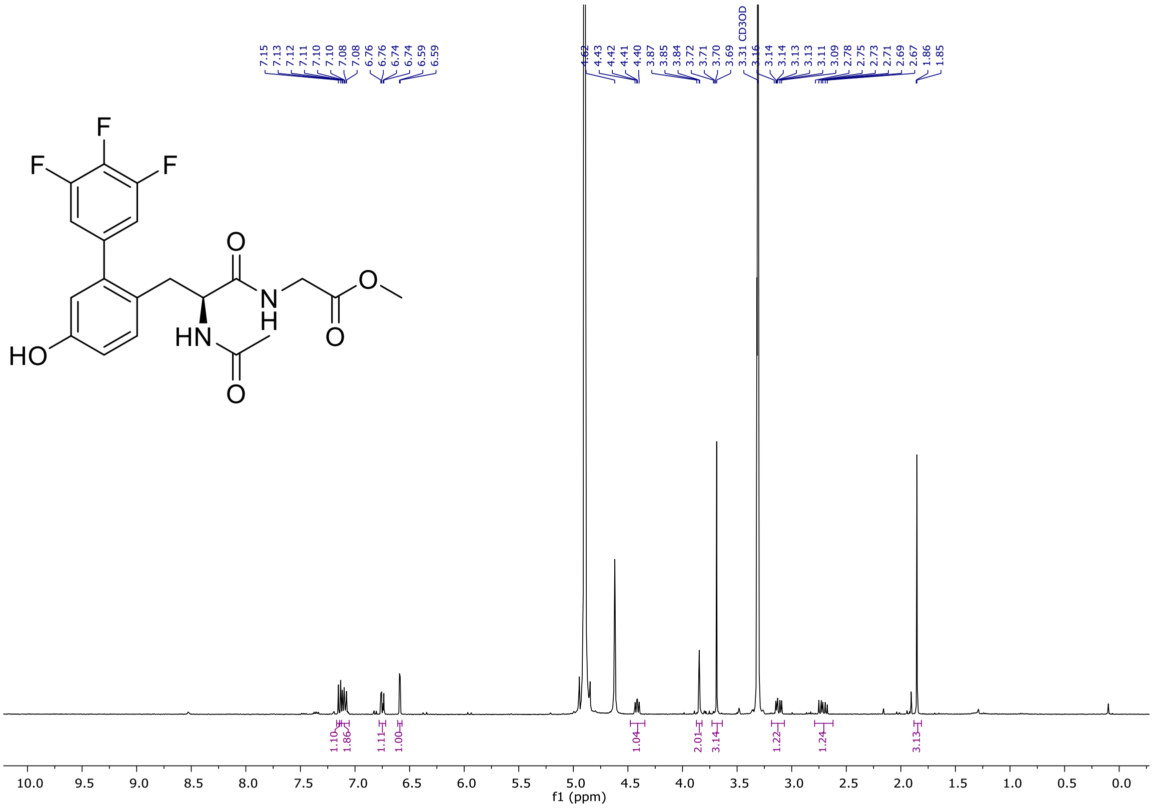

^{13}C-NMR in CD_3OD, 101 MHz

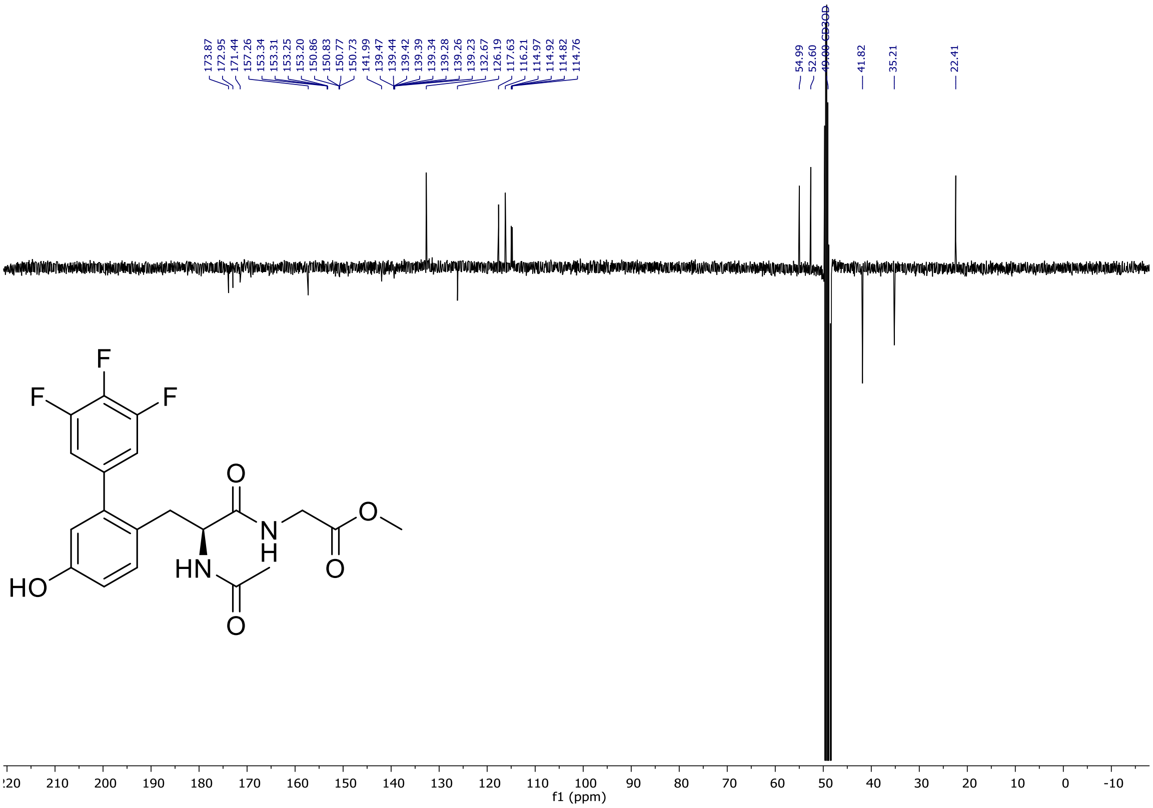

3ha

^{1}H NMR in CD_3OD, 400 MHz

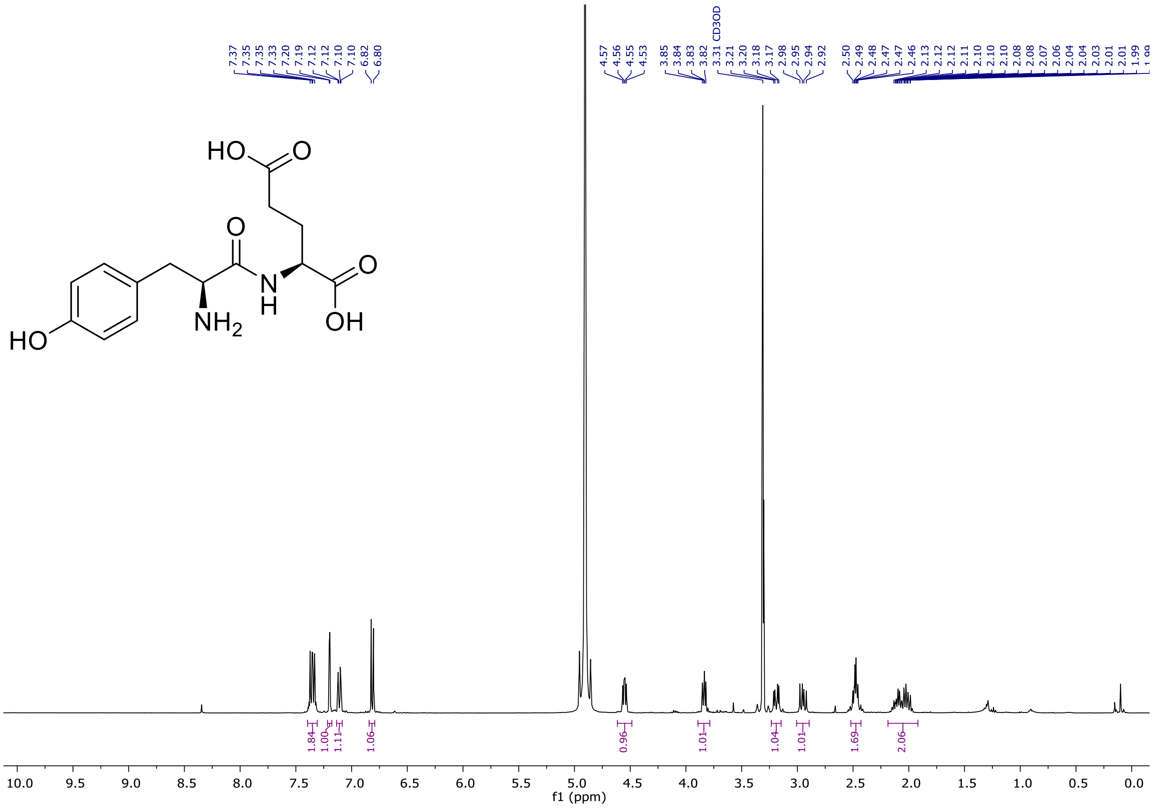

^{13}C-NMR in CD_3OD, 101 MHz

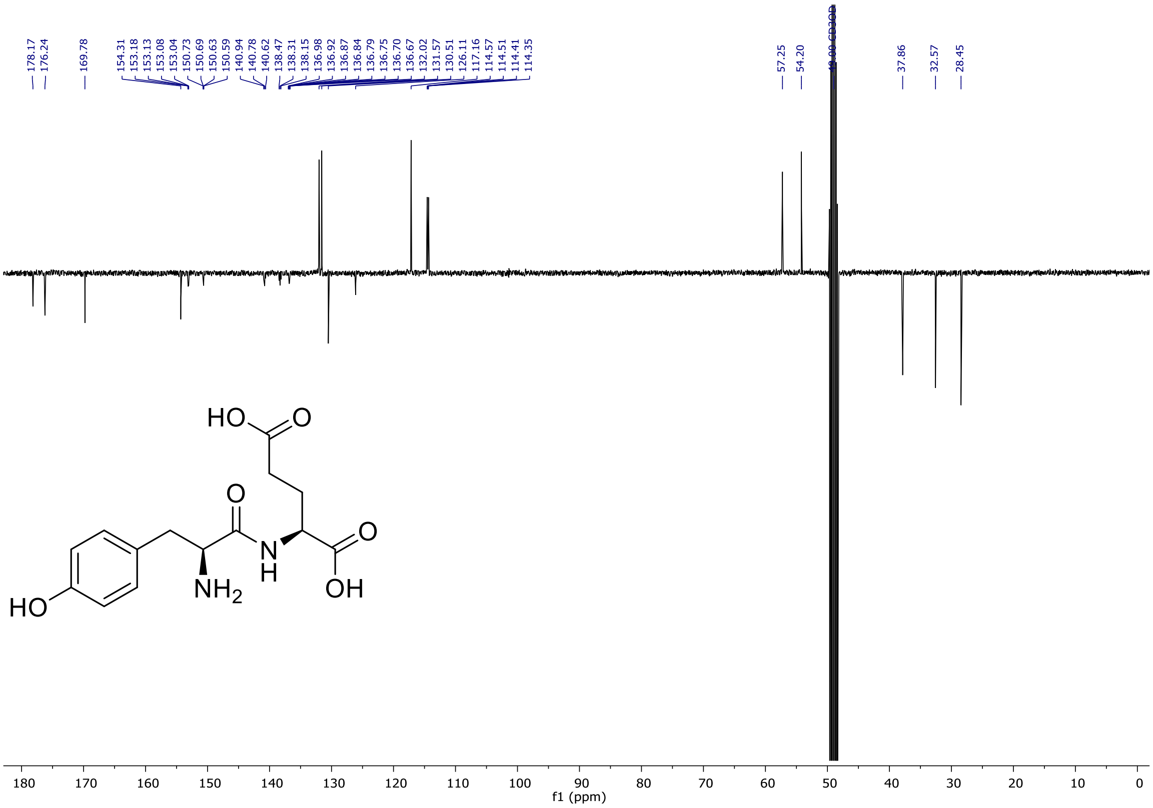

3ia

^{1}H NMR in CD_3OD, 400 MHz

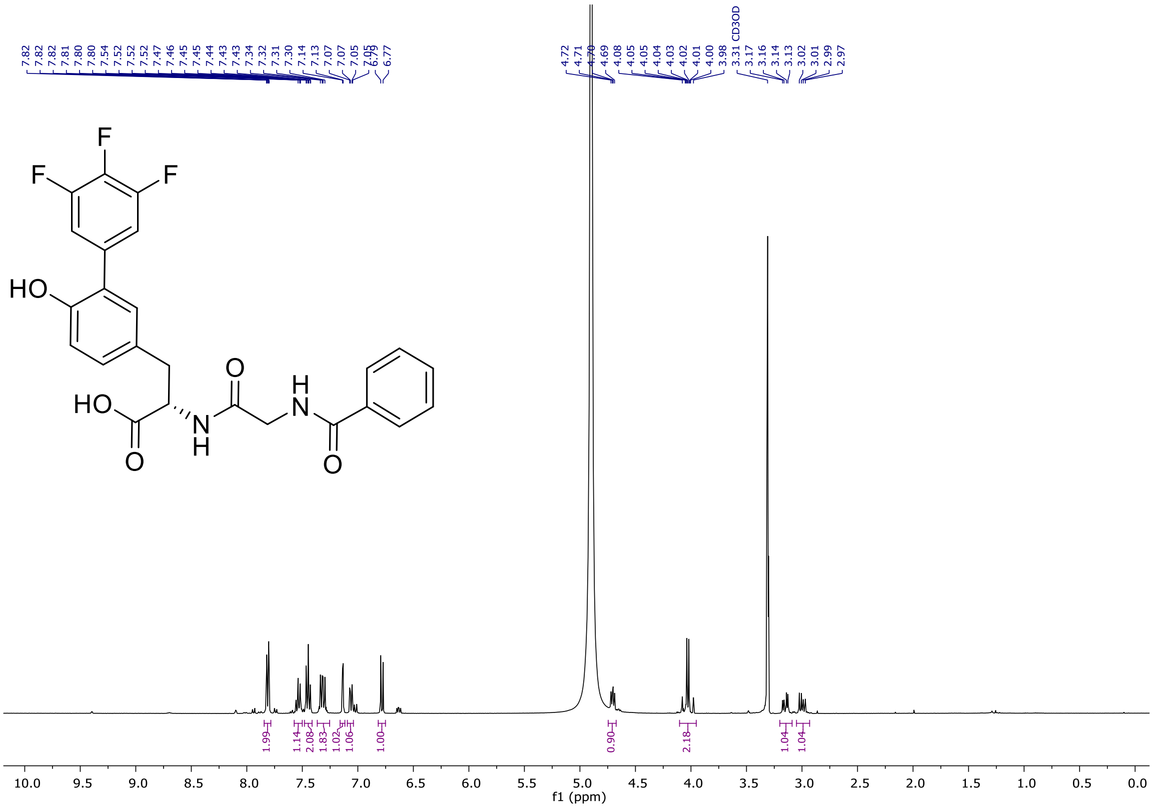

^{13}C-NMR in CD_3OD, 101 MHz

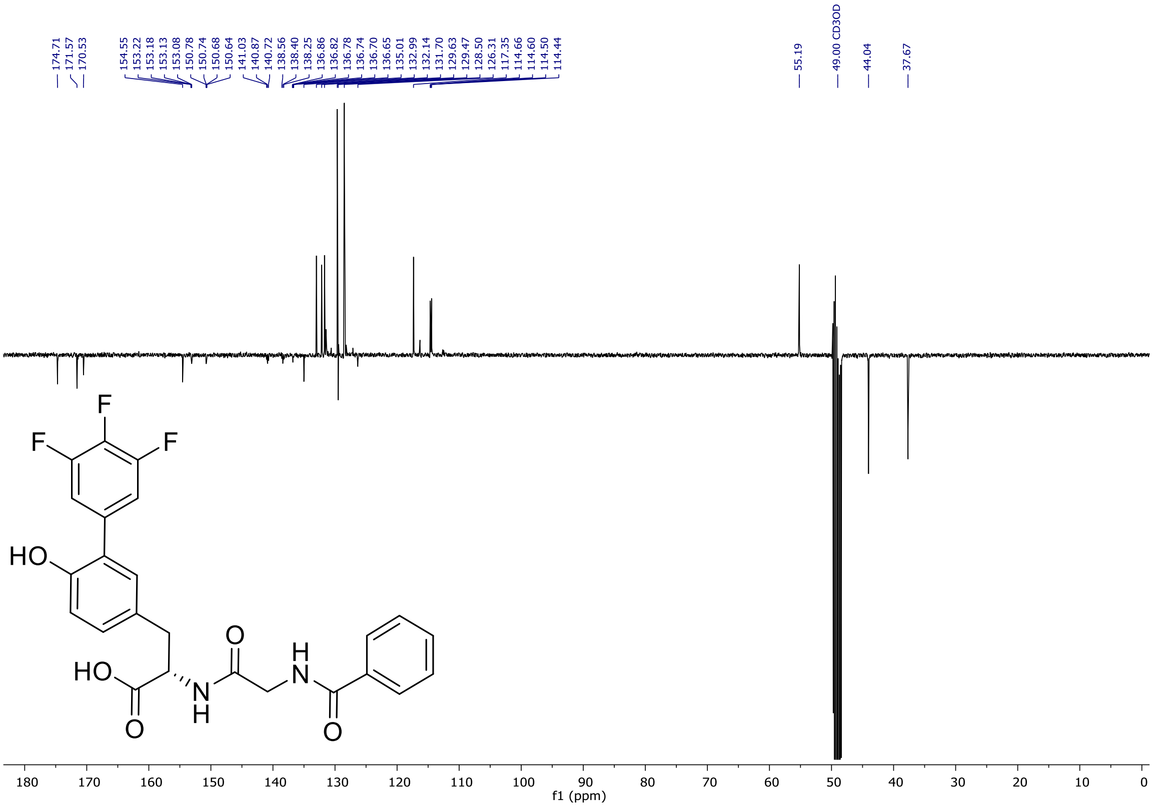

3ja

^{1}H NMR in CD_3OD, 400 MHz

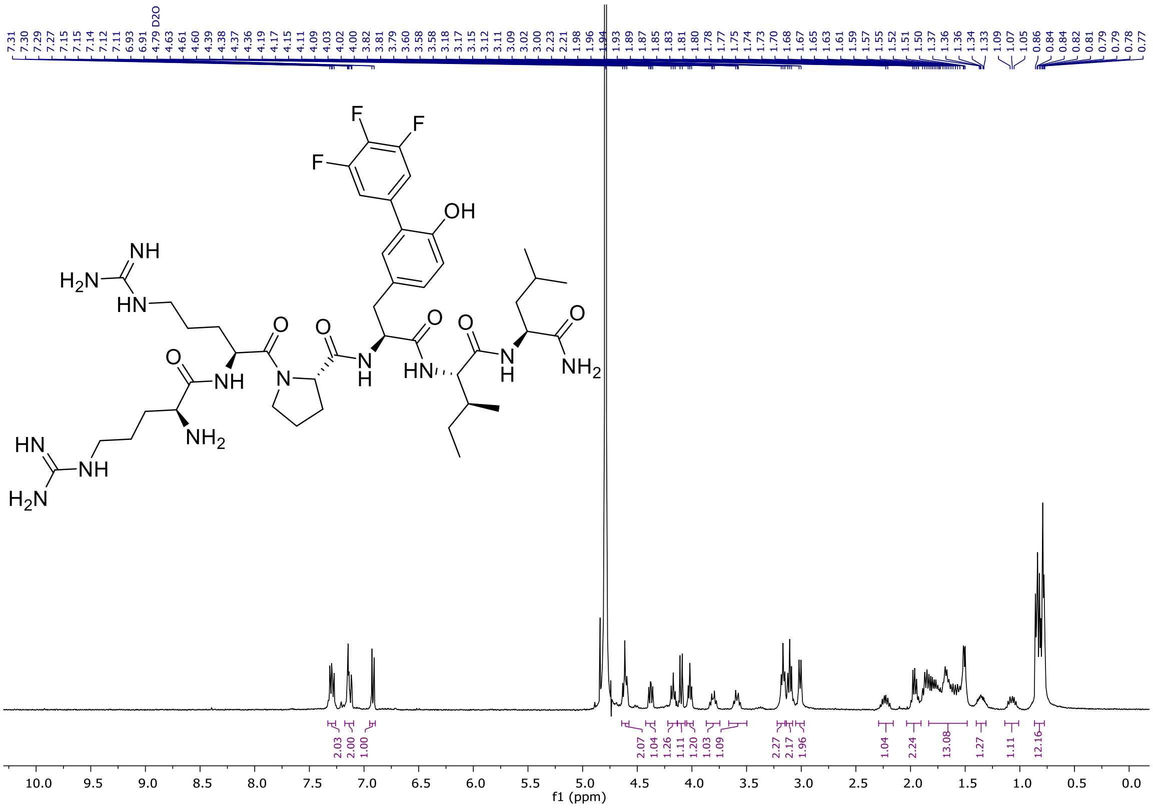

^{13}C-NMR in CD_3OD, 101 MHz

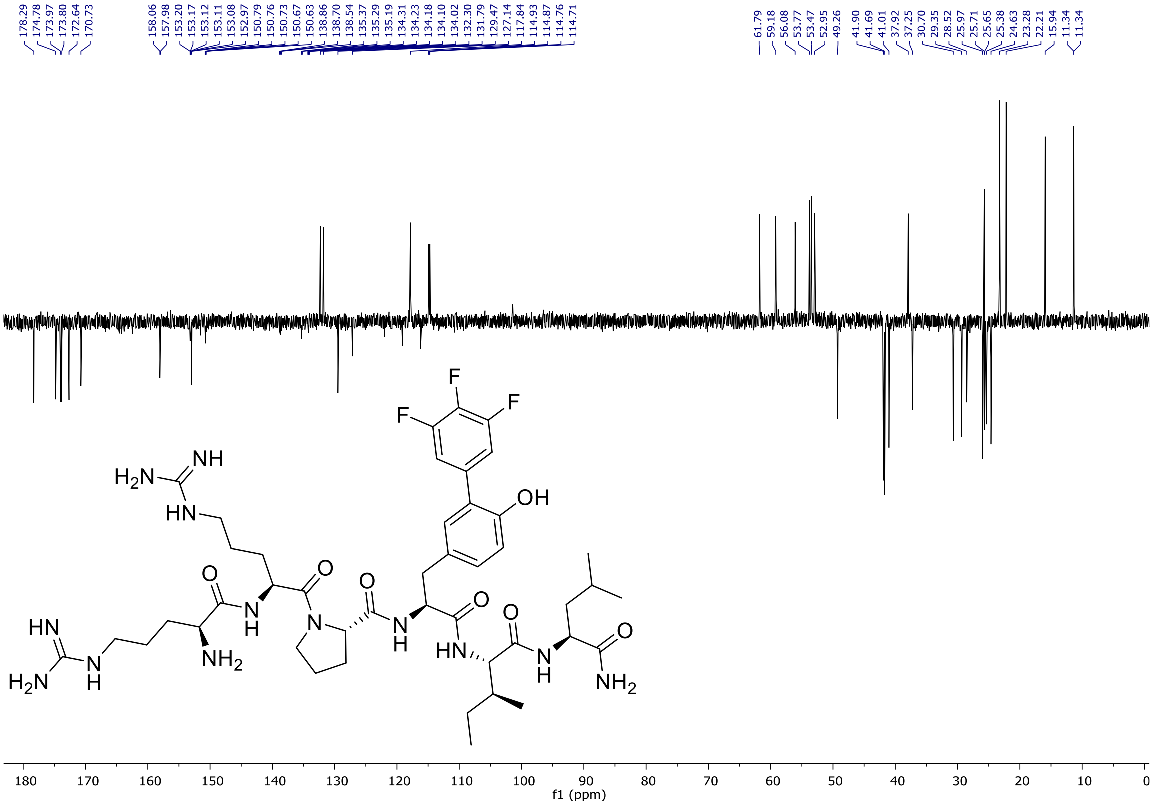

3ka

^{1}H NMR in CD_3OD, 400 MHz

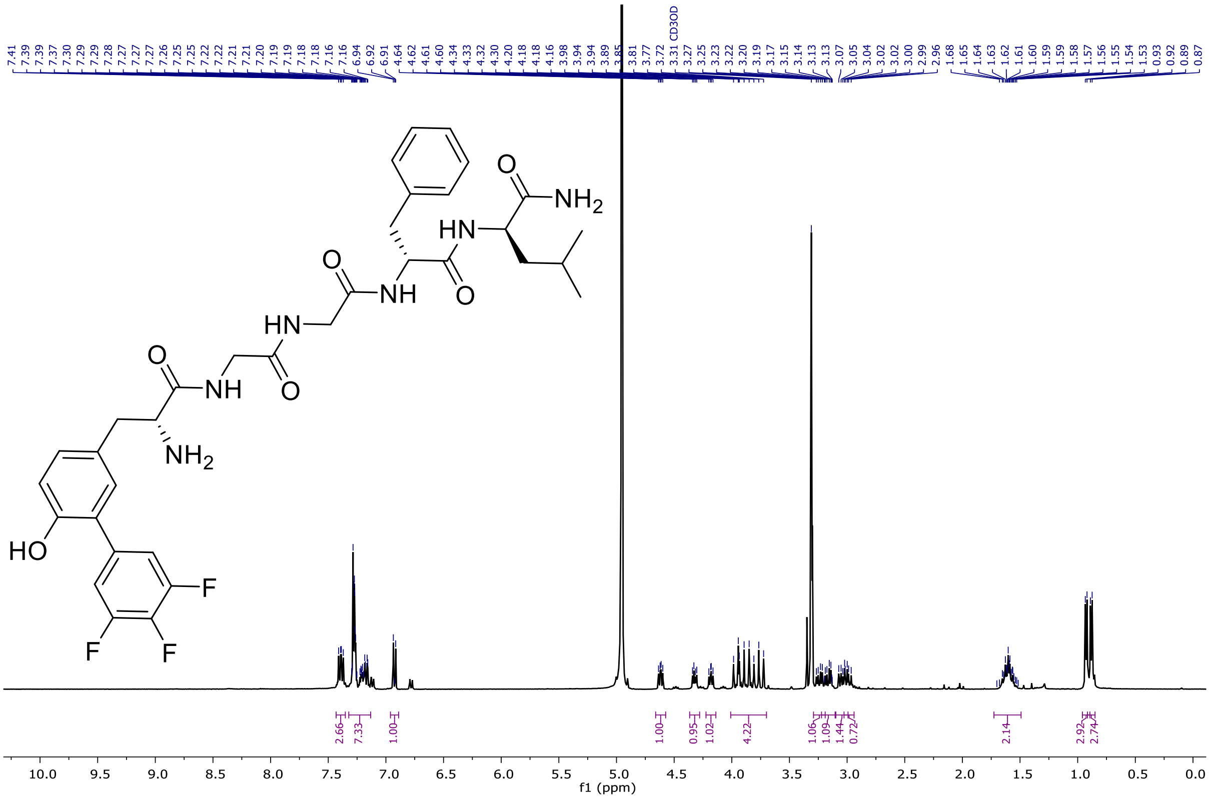

^{13}C-NMR in CD_3OD, 101 MHz

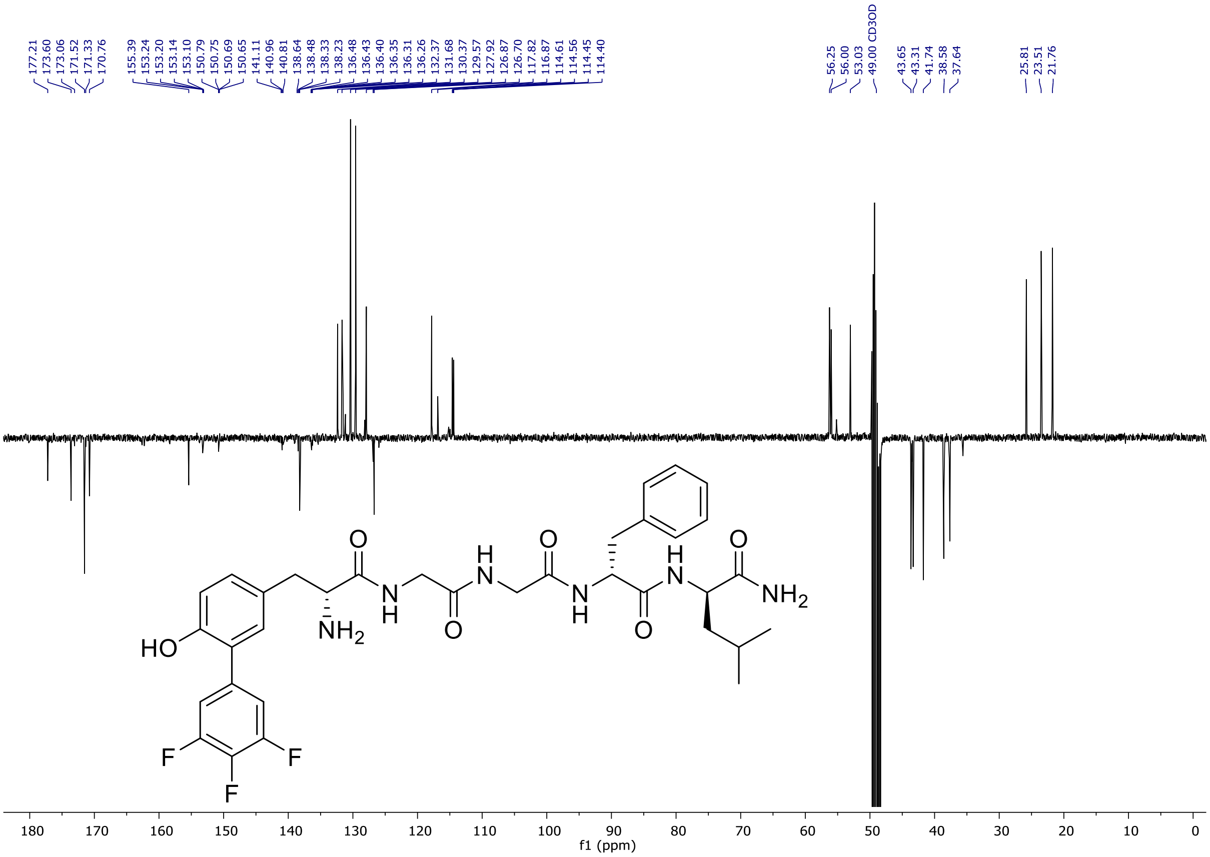

3nb

^{1}H NMR in CD_3OD, 400 MHz

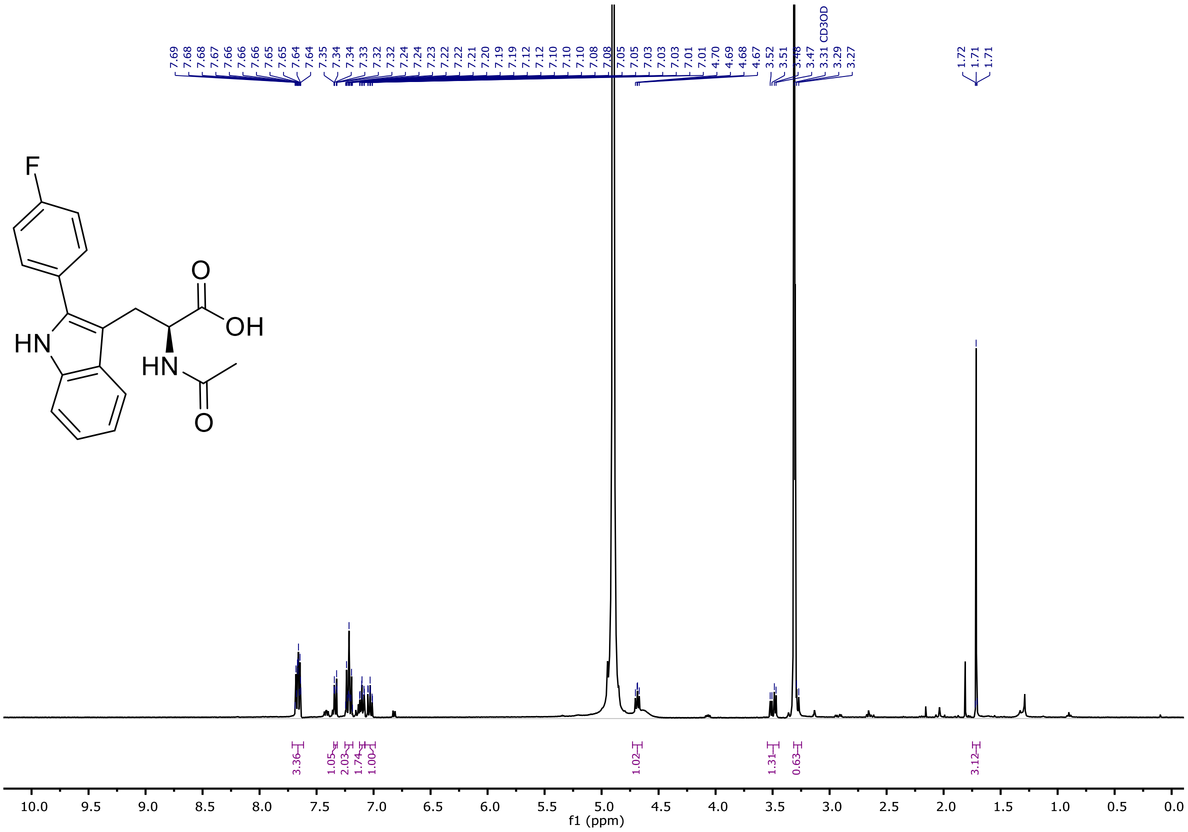

^{13}C-NMR in CD_3OD, 101 MHz

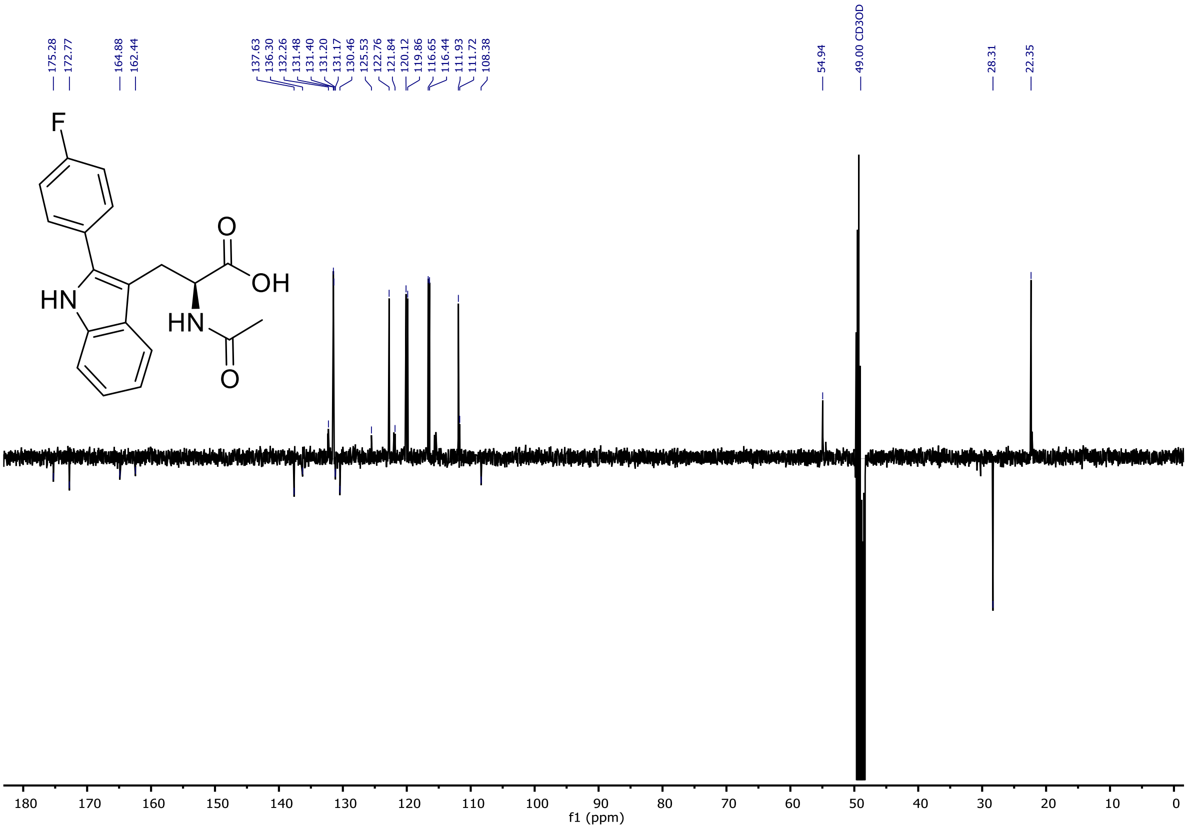

3nb'

^{1}H NMR in CD_3OD, 400 MHz

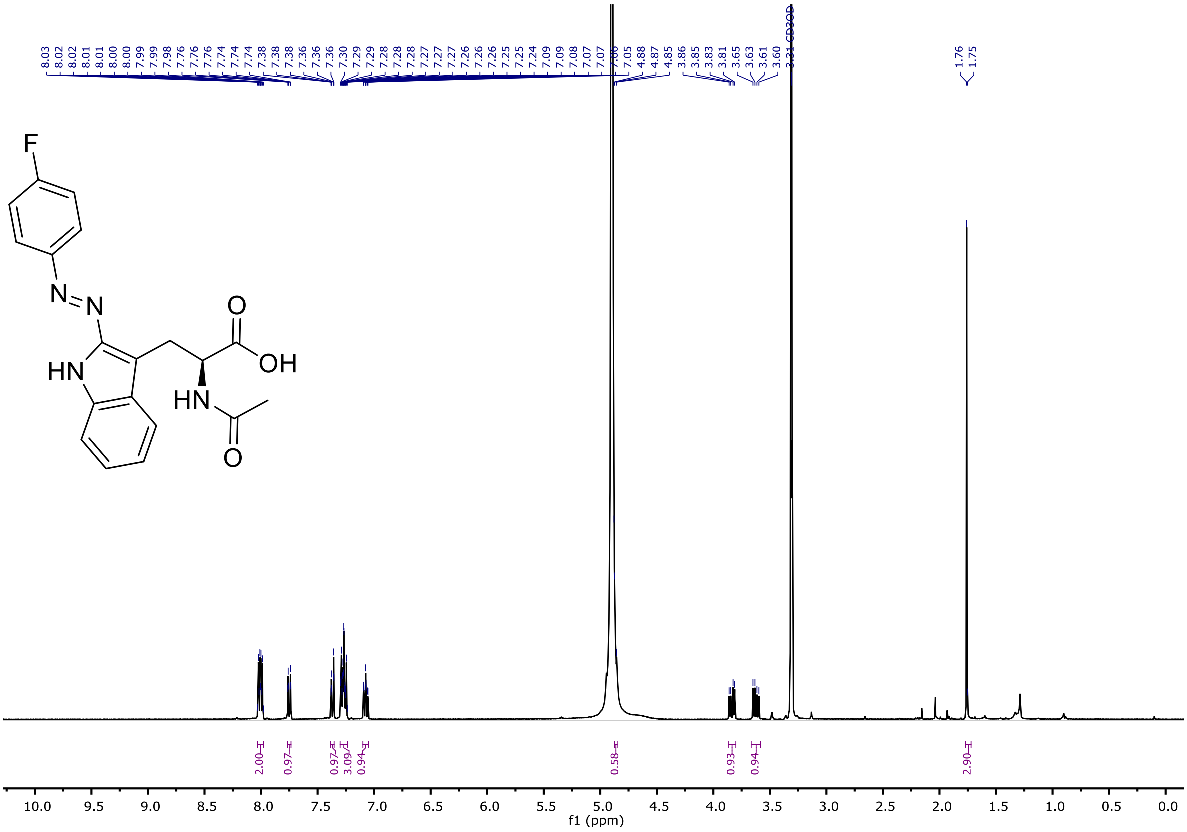

^{13}C-NMR in CD_3OD, 101 MHz

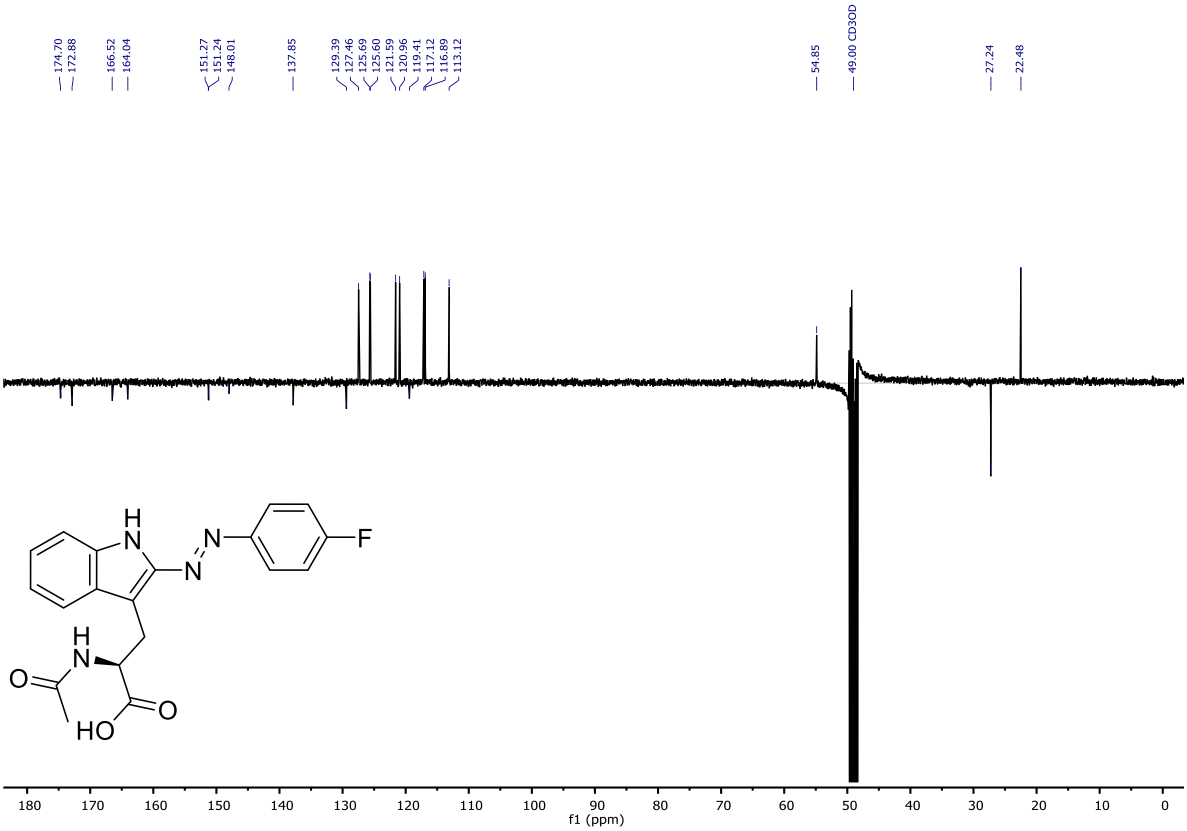

3oa’

^{1}H NMR in CD_3OD, 400 MHz

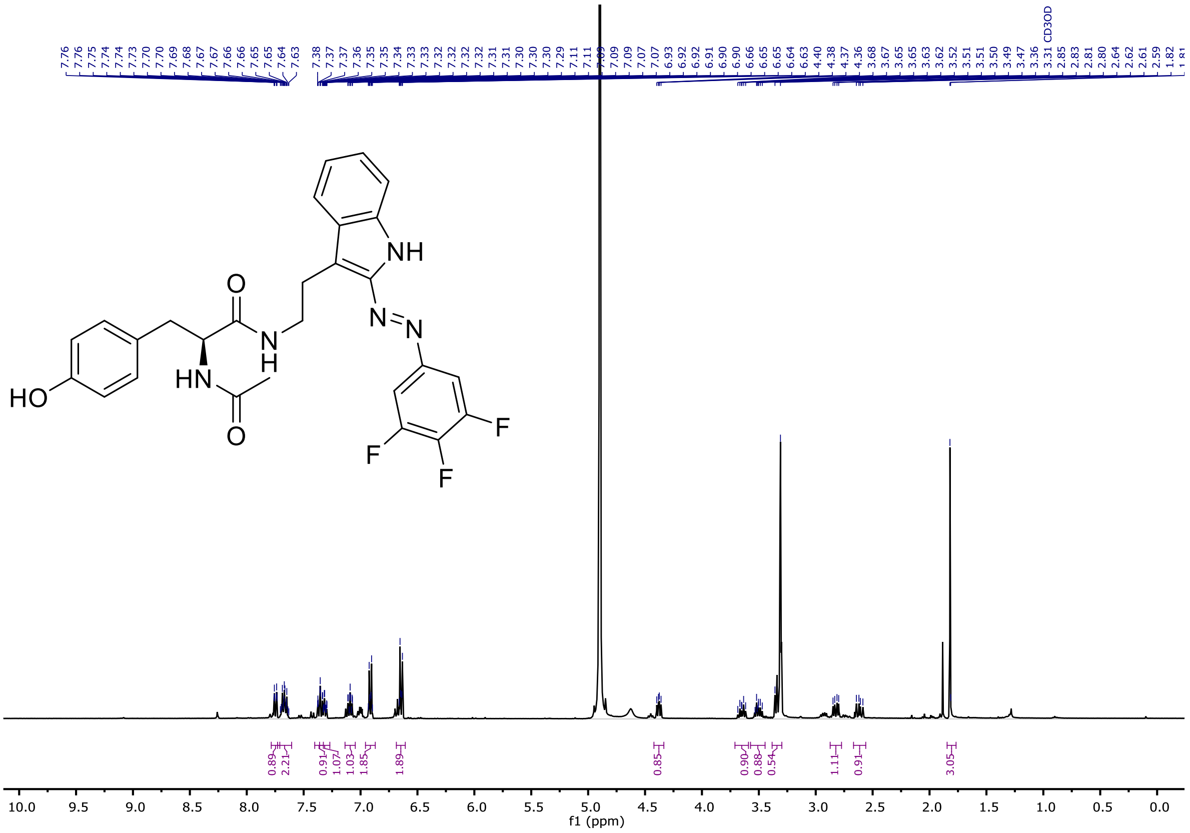

^{13}C-NMR in CD_3OD, 101 MHz

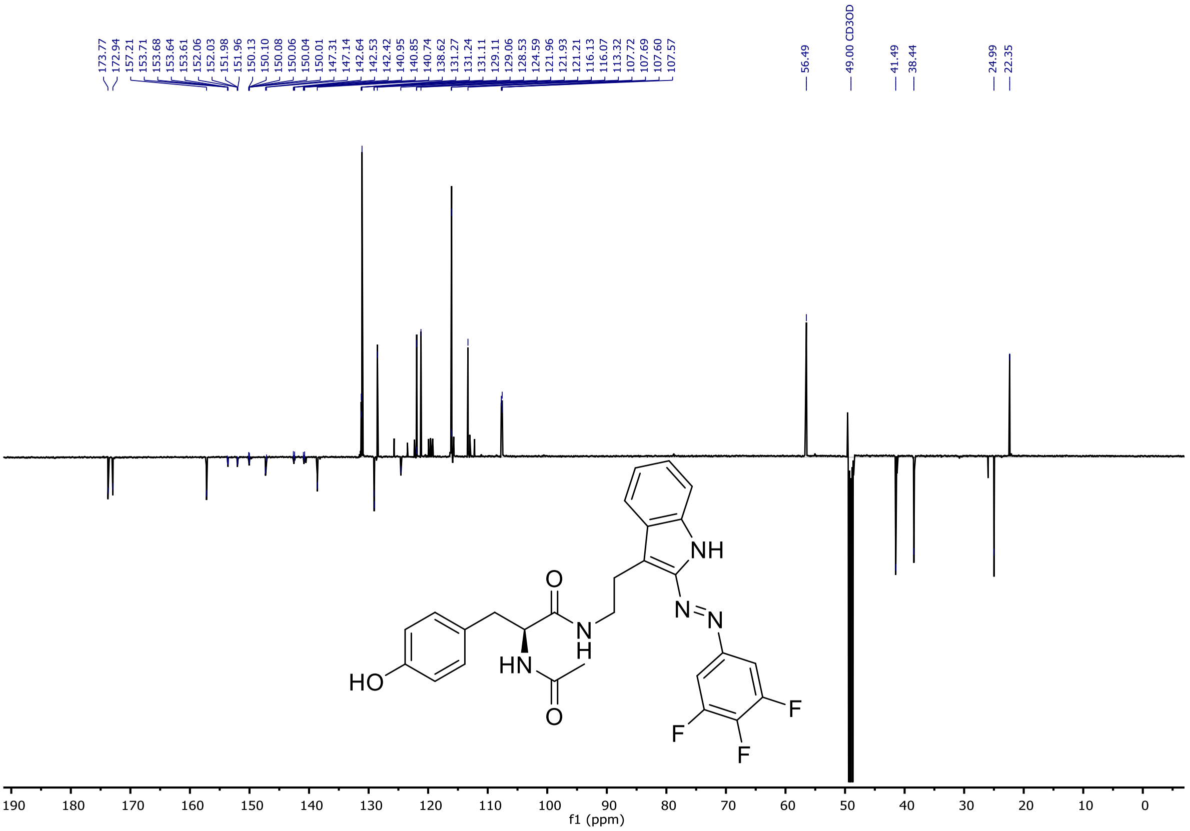

3ob

^{1}H NMR in CD_3OD, 400 MHz

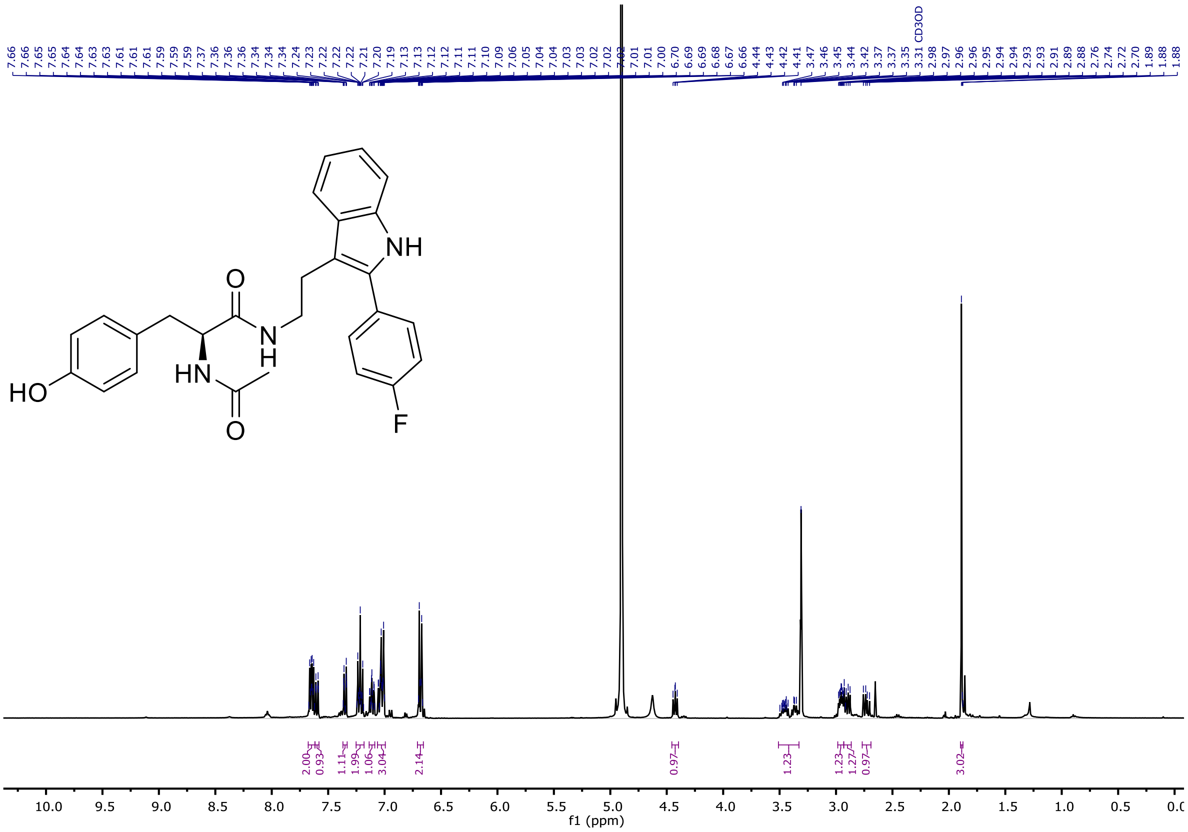

^{13}C-NMR in CD_3OD, 101 MHz

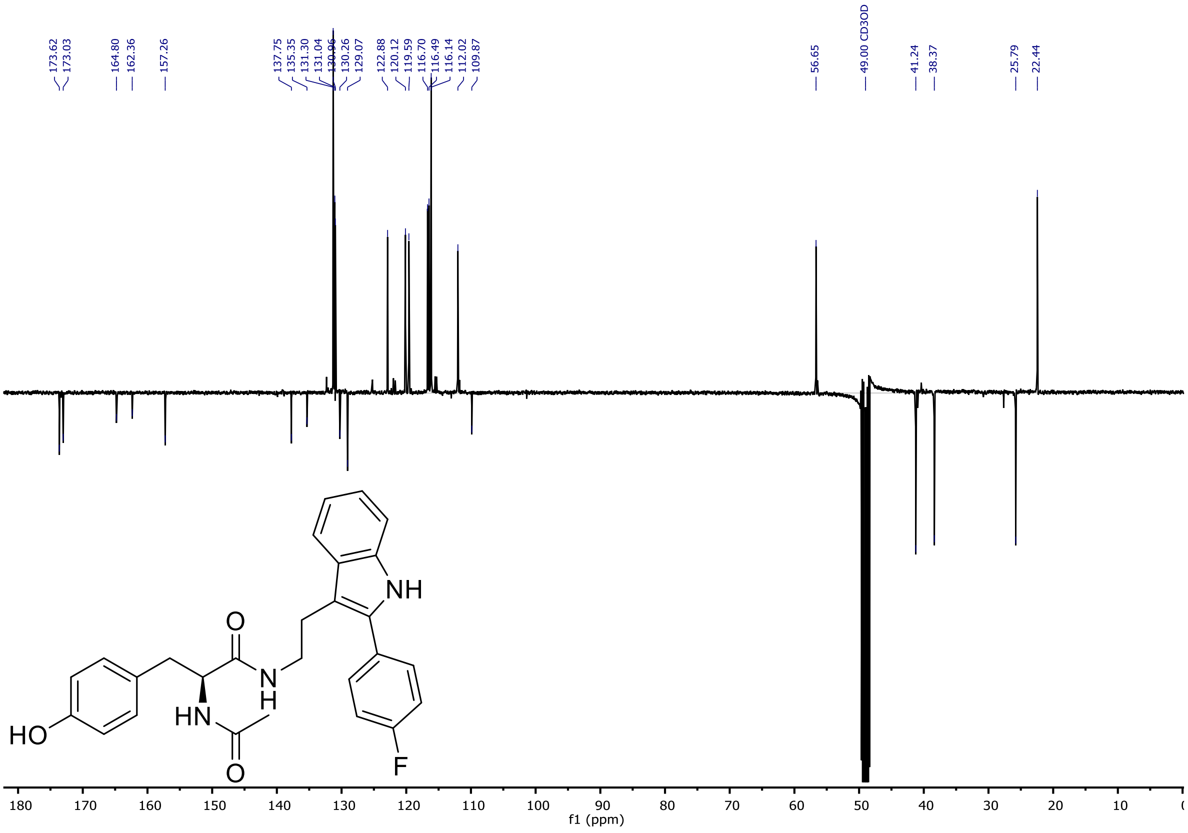

3ob’

^{1}H NMR in CD_3OD, 600 MHz

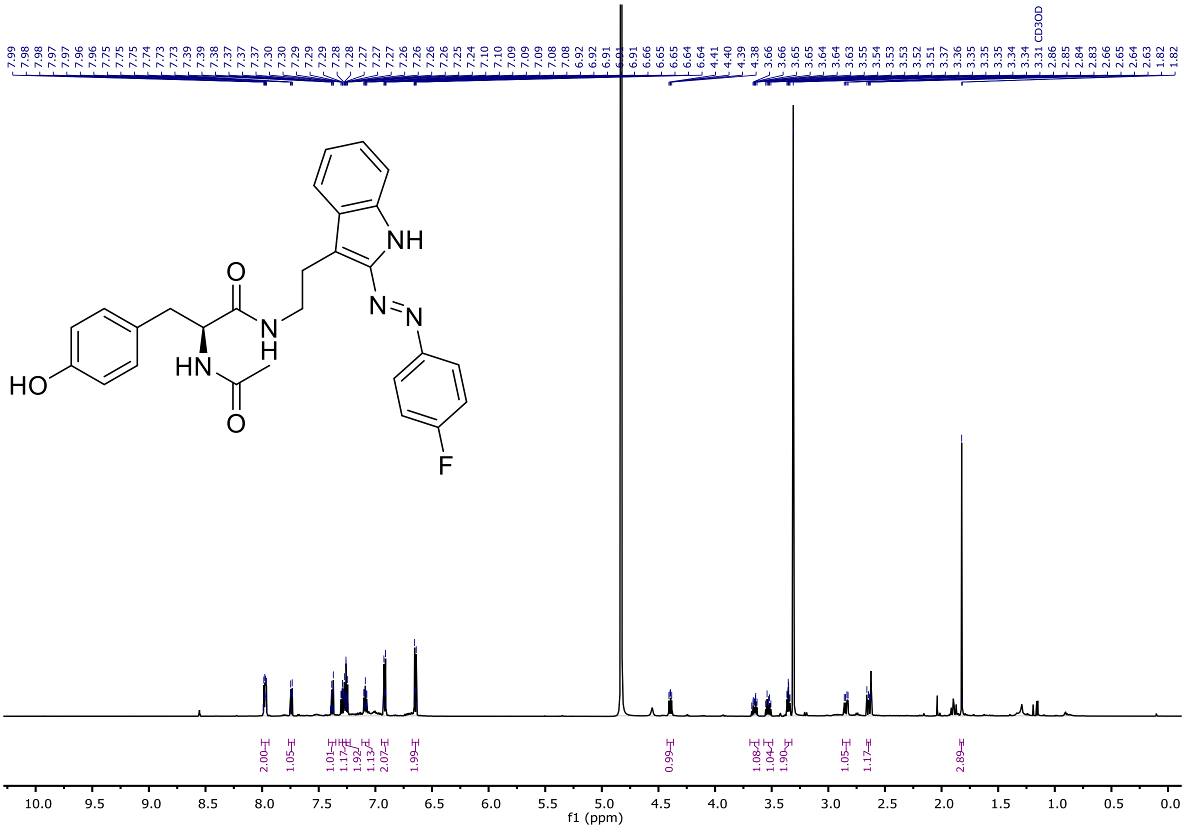

^{13}C-NMR in CD_3OD, 151 MHz

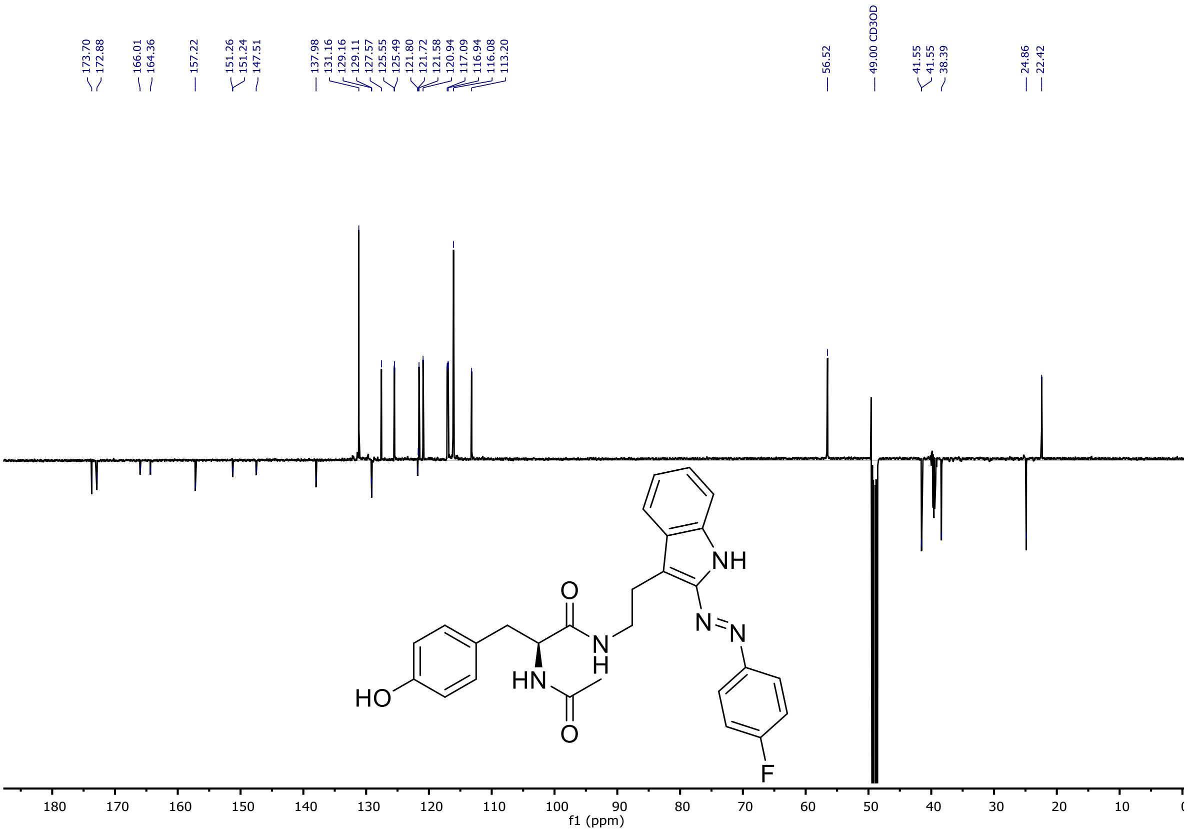

3,4,5-trifluoro-4'-nitro-1,1'-biphenyl

^{1}H NMR in CD_3OD, 400 MHz

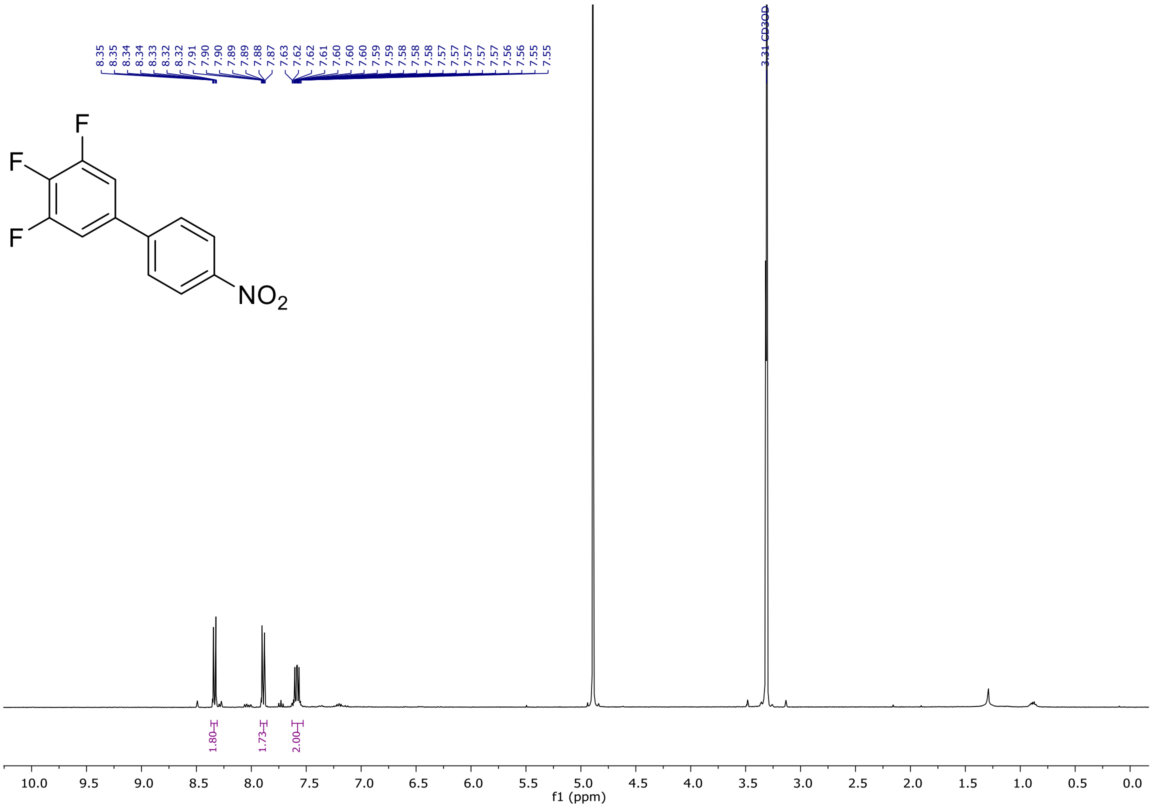

^{13}C-NMR in CD_3OD, 101 MHz

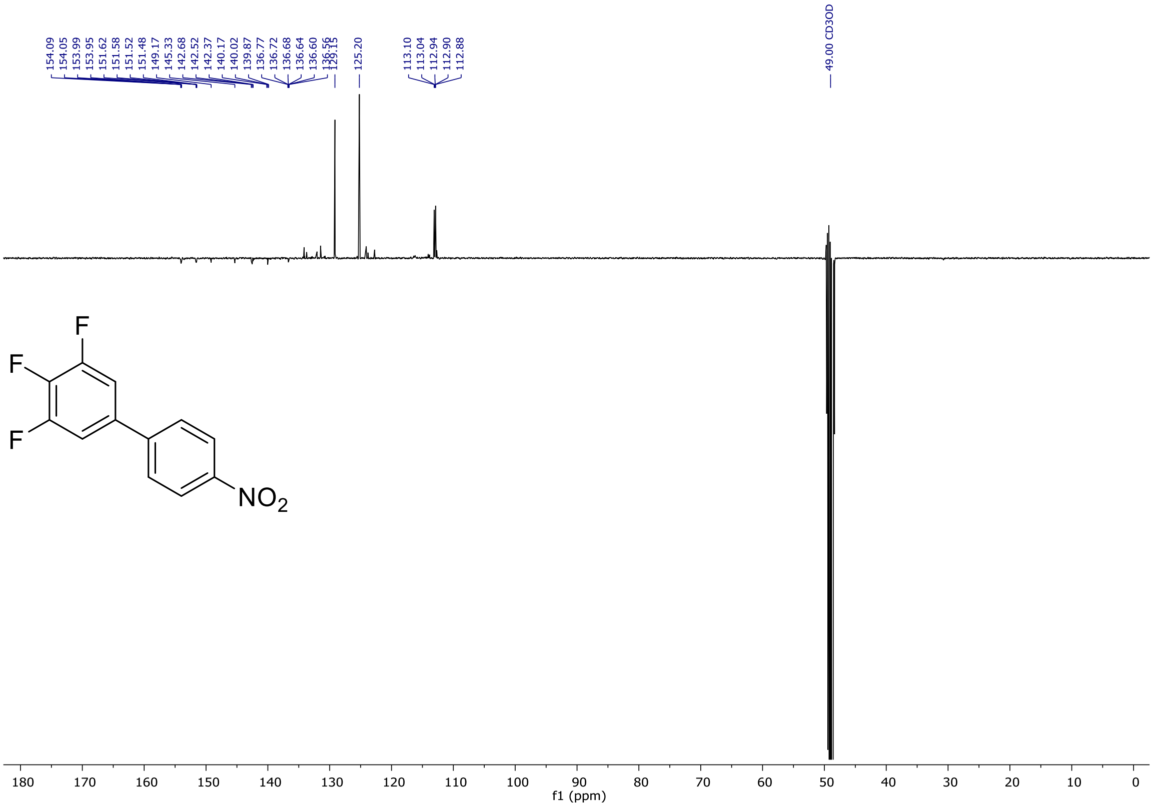

7a

^{1}H NMR in $(CD_3)_2SO$, 400 MHz

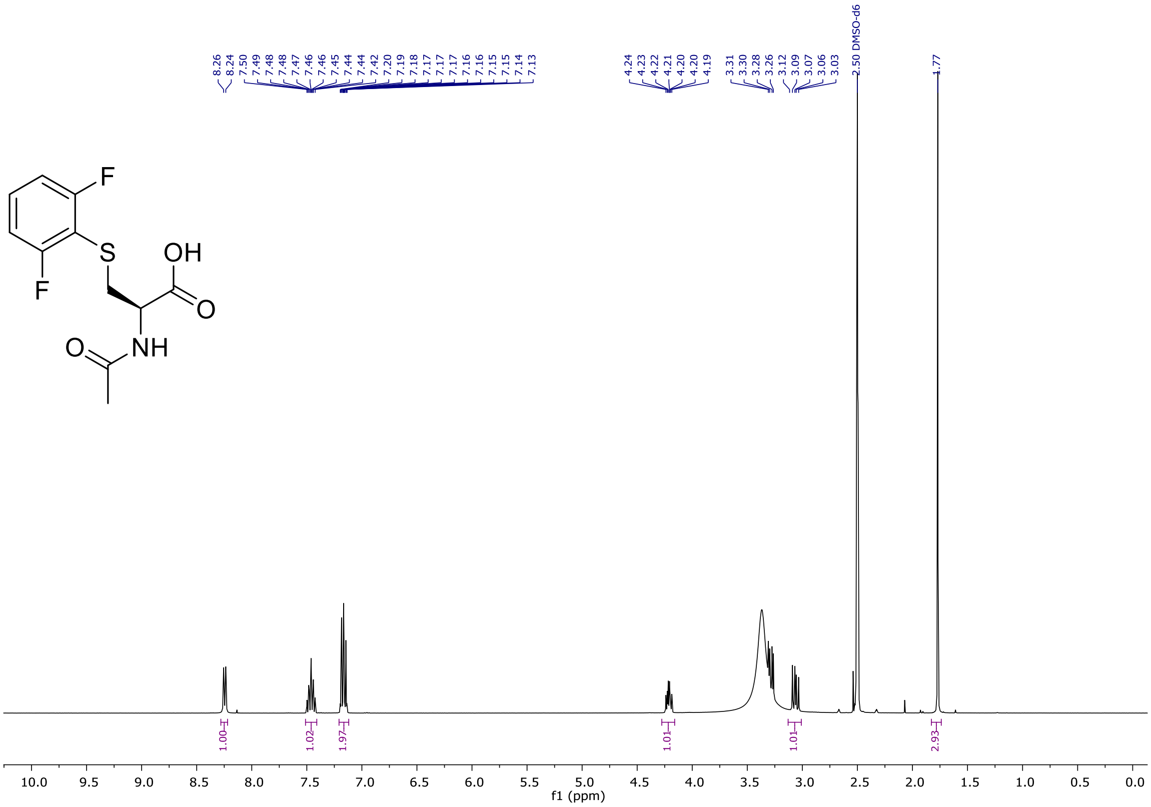

^{13}C-NMR in $(CD_3)_2SO$, 101 MHz

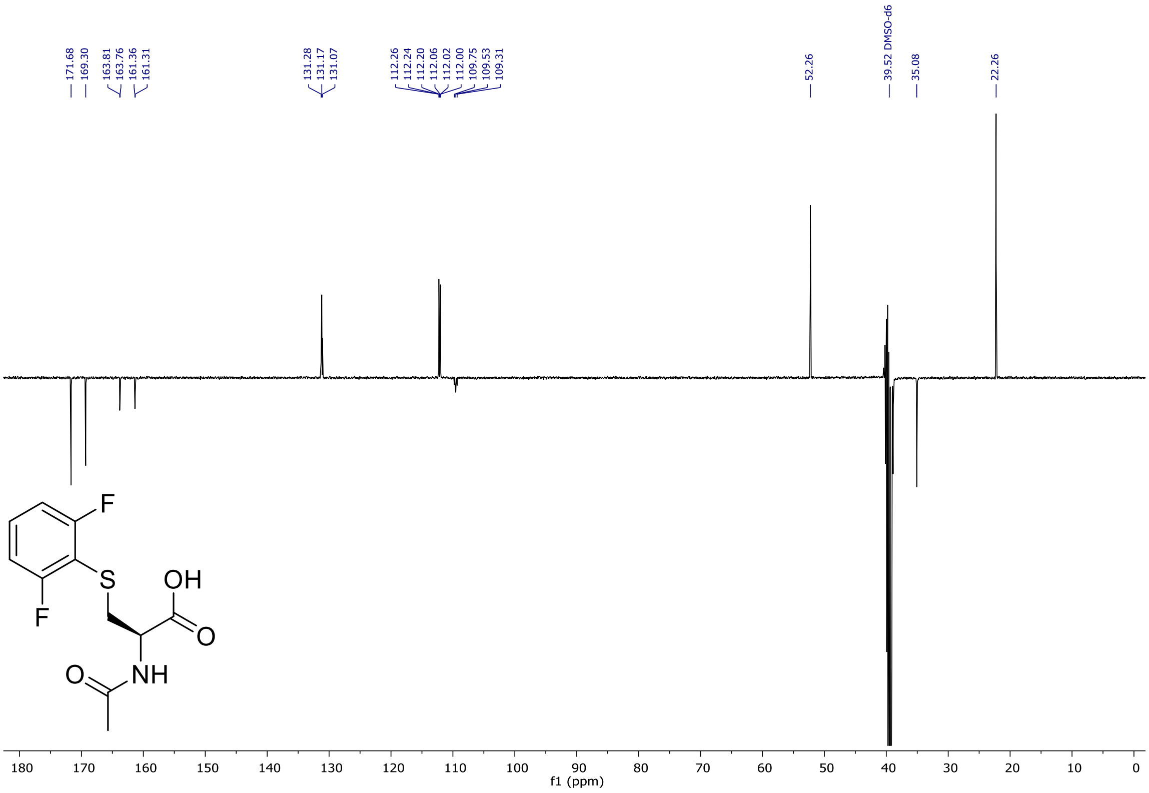

7b

^{1}H NMR in $(CD_3)_2SO$, 400 MHz

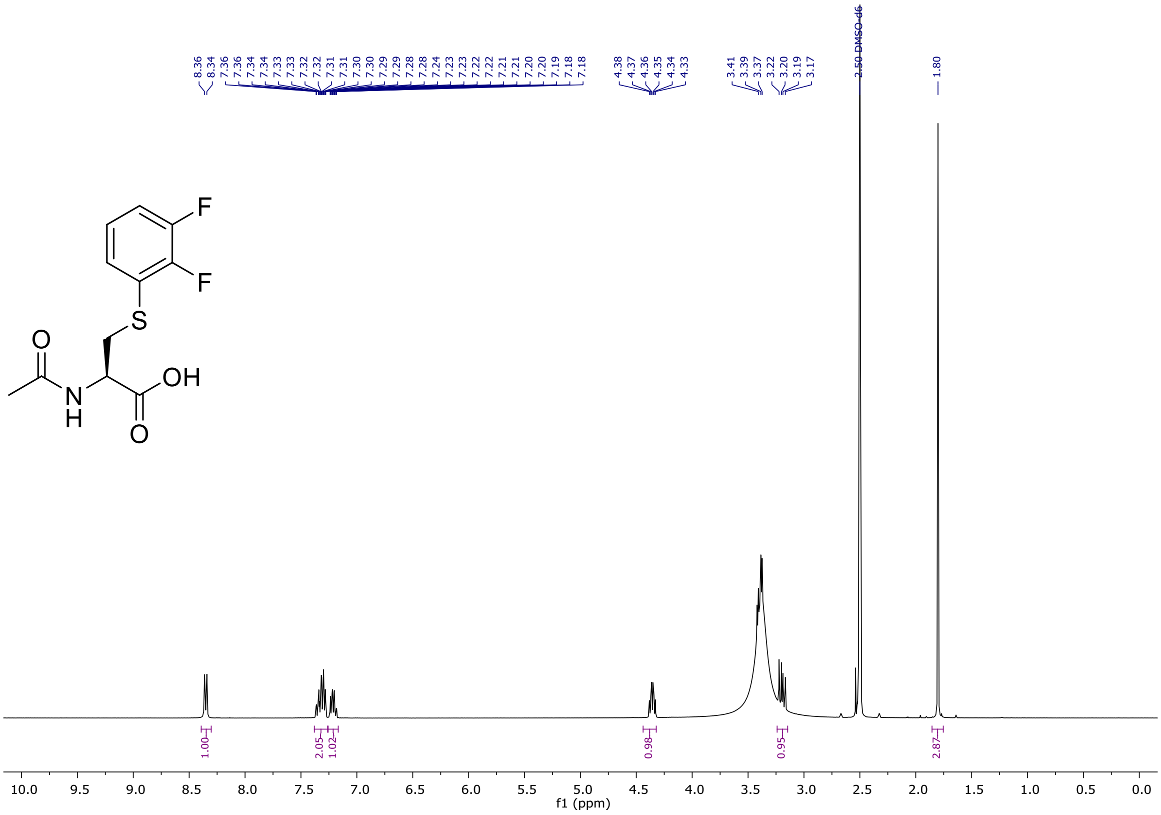

^{13}C-NMR in $(CD_3)_2SO$, 101 MHz

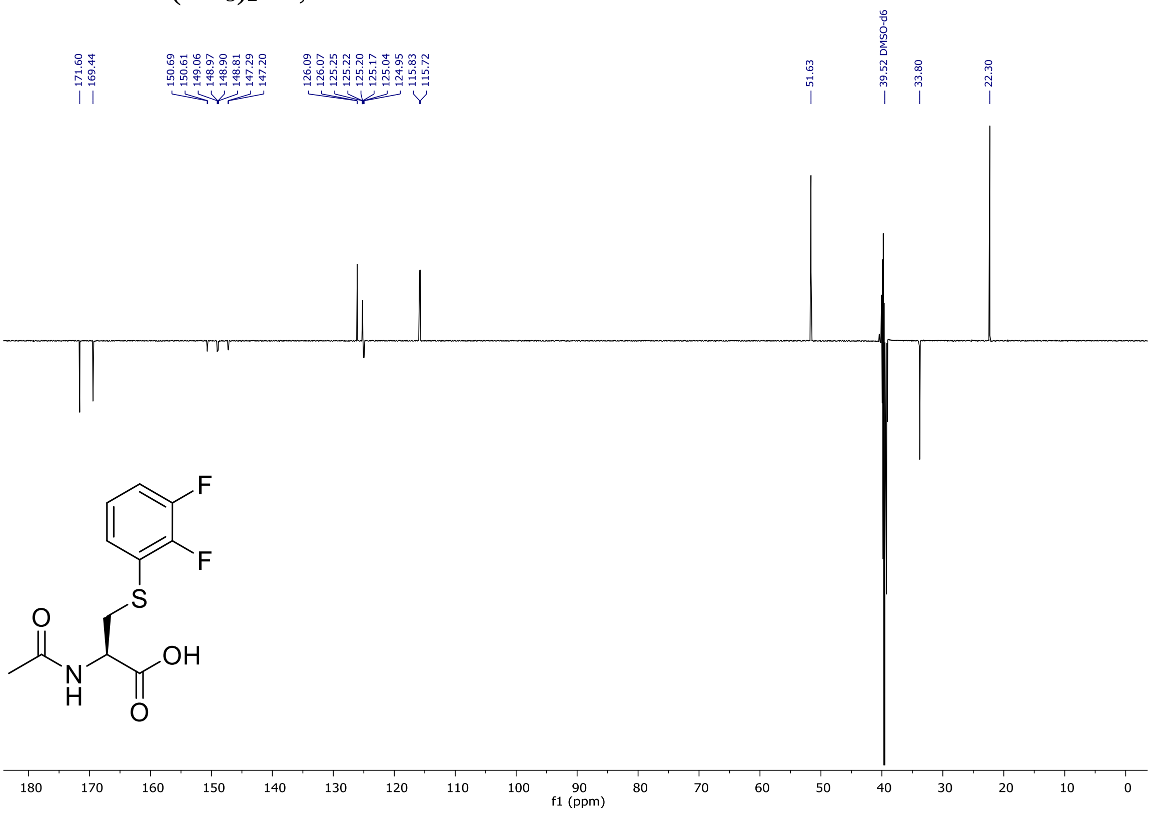

8

^{1}H NMR in $(CD_3)_2SO$, 400 MHz

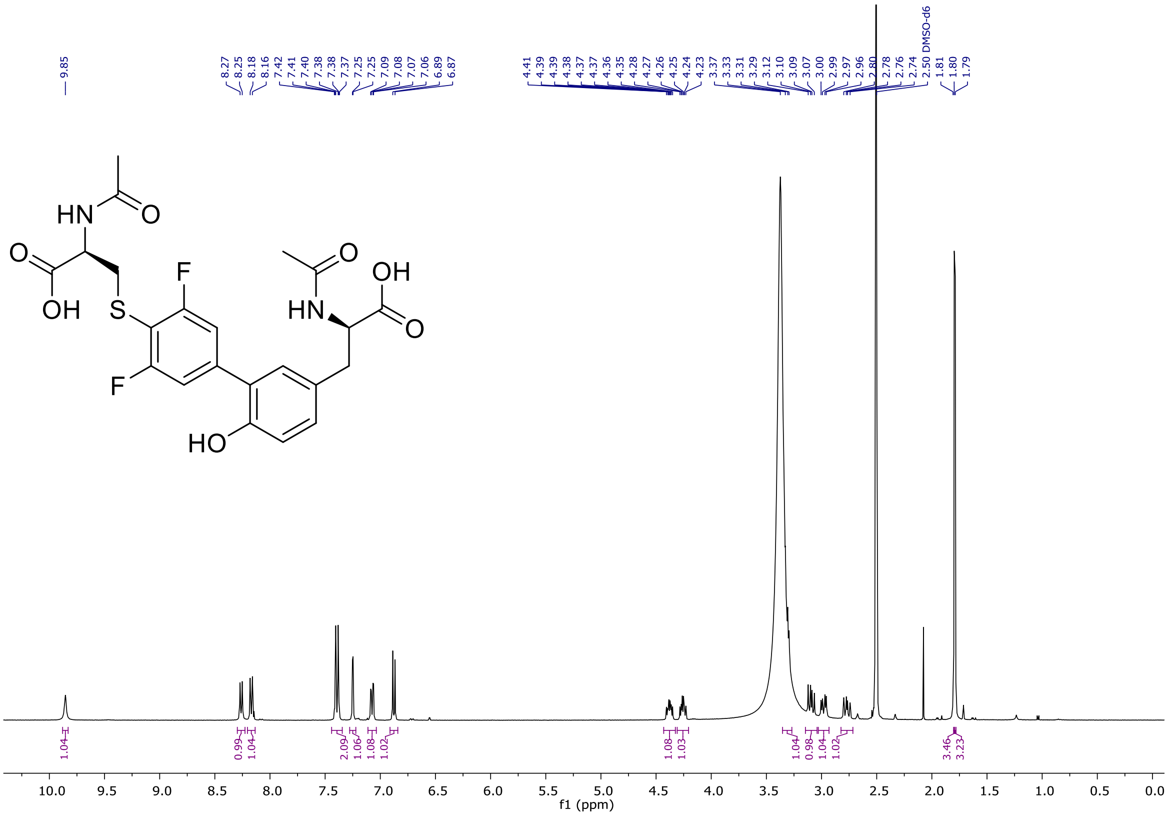

^{13}C-NMR in $(CD_3)_2SO$, 101 MHz

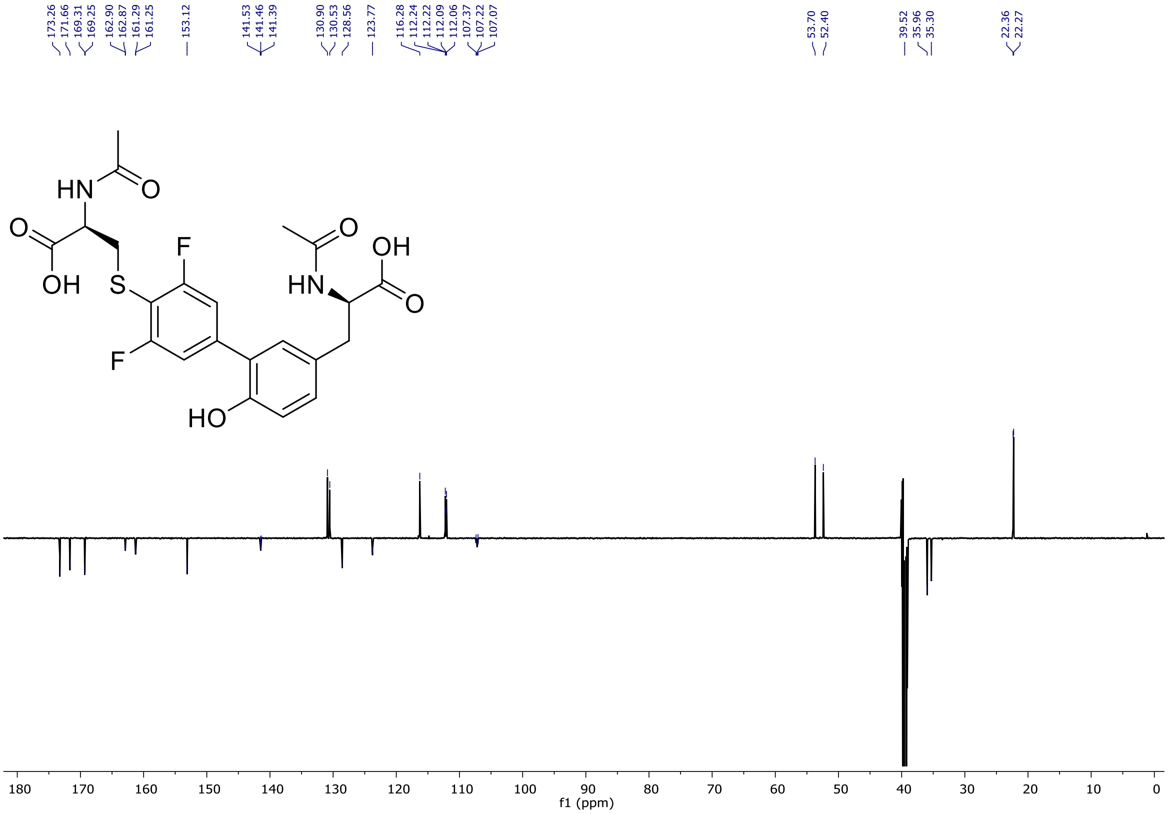

8. References

[1] M. C. D. Fürst, E. Gans, M. J. Böck, M. R. Heinrich, *Chem. Eur. J.* **2017**, *23*, 15312–15315.

[2] S. Rajput, K. J. McLean, H. Poddar, I. R. Selvam, G. Nagalingam, J. A. Triccas, C. W. Levy, A. W. Munro, C. A. Hutton, *J. Med. Chem.* **2019**, *62*, 9792–9805.

[3] L. Weißenborn, E. Richel, H. Hüseman, J. Welzer, S. Beck, S. Schäfer, H. Sticht, K. Überla, J. Eichler, *Int. J. Mol. Sci.* **2022**, *23,* 6309.

[4] M. Bremerich, C. M. Conrads, T. Langletz, C. Bolm, *Angew. Chem. Int. Ed.* **2019**, *58,* 19014–19020.

[5] M. P. Stewart, F. Maya, D. V. Kosynkin, S. M. Dirk, J. J. Stapleton, C. L. McGuiness, D. L. Allara, J. M. Tour, *J. Am. Chem. Soc.* **2004**, *126*, 370–378.

[6] S. Andrejčák, P. Kisszékelyi, M. Májek, R. Šebesta, *Eur. J. Org. Chem.* **2023**, *26*, e202201399.

[7] D. Kosynkin, T. M. Bockman, and J. K. Kochi, *J. Am. Chem. Soc.* **1997**, *119*, 4846–4855.

[8] S. K. Fehler, G. Pratsch, C. Östreicher, M. C.D. Fürst, M. Pischetsrieder, M. R. Heinrich, *Tetrahedron* **2016**, *72*, 7888–7893.

[9] E. R. M. Habraken, L. J. C. van der Zee, K.n N. A. van de Vrande, A. R. Jupp, M. Nieger, A. W. Ehlers, J. C. Slootweg, *Eur. J. Inorg. Chem.* **2019**, 1594–1603.

[10] B. Vergani, G. Sandrone, M. Marchini, C. Ripamonti, E. Cellupica, E. Galbiati, G. Caprini, G. Pavich, G. Porro, I. Rocchio, M. Lattanzio, M. Pezzuto, M. Skorupska, P. Cordella, P. Pagani, P. Pozzi, R. Pomarico, D. Modena, F. Leoni, R. Perego, G. Fossati, C. Steinkühler, A. Stevenazzi, *J. Med. Chem.* **2019**, *62*, 10711–10739.

[11] S. K. Fehler, G. Pratsch, W. Huber, A. Gast, R. Hochstrasser, M. Hennig, M. R. Heinrich, *Tetrahedron* **2012**, *53*, 2189-2194
